# Atom-economic catalytic amide synthesis from amines and carboxylic acids activated *in situ* with acetylenes

**DOI:** 10.1038/ncomms11732

**Published:** 2016-06-10

**Authors:** Thilo Krause, Sabrina Baader, Benjamin Erb, Lukas J. Gooßen

**Affiliations:** 1 FB Chemie-Organische Chemie, Technische Universität Kaiserslautern, Erwin Schrödinger Strasse Geb. 54, 67663 Kaiserslautern, Germany

## Abstract

Amide bond-forming reactions are of tremendous significance in synthetic chemistry. Methodological research has, in the past, focused on efficiency and selectivity, and these have reached impressive levels. However, the unacceptable amounts of waste produced have led the ACS GCI Roundtable to label ‘amide bond formation avoiding poor atom economy' as the most pressing target for sustainable synthetic method development. In response to this acute demand, we herein disclose an efficient one-pot amide coupling protocol that is based on simple alkynes as coupling reagents: in the presence of a dichloro[(2,6,10-dodecatriene)-1,12-diyl]ruthenium catalyst, carboxylate salts of primary or secondary amines react with acetylene or ethoxyacetylene to vinyl ester intermediates, which undergo aminolysis to give the corresponding amides along only with volatile acetaldehyde or ethyl acetate, respectively. The new amide synthesis is broadly applicable to the synthesis of structurally diverse amides, including dipeptides.

## Abstract (web summary)

Amide formation is a ubiquitous reaction in organic chemistry, but suffers from the problem of generating large amounts of waste. Here, the authors report a catalytic amide synthesis by the ruthenium catalysed addition of carboxylic acids to acetylenes, followed by reaction with primary or secondary amines.

## Graphical abstract

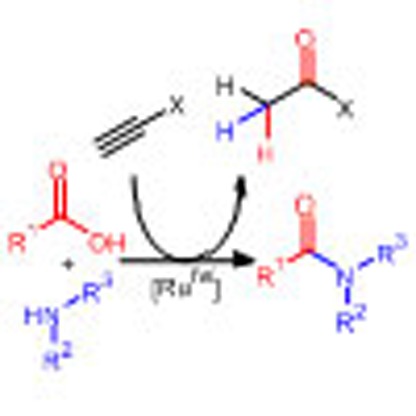

Amide bond formation is one of the most frequently used transformations in organic chemistry^1^,^2^,^3^,^4^. The most desirable amide synthesis, a direct condensation of carboxylic acids with amines, is hindered by the intrinsic acid–base reactivity of the starting materials. The thermal amide bond formation from the ammonium carboxylate salts requires high temperatures^5^,^6^,^7^, which can be lowered by Lewis acids or boronic acid derivatives. However, even the best known systems are limited to a narrow range of amines and require scavenging the reaction water, for example, by large amounts of molecular sieves. (Fig. 1, left)^8^,^9^,^10^,^11^,^12^,^13^. Therefore, amides are usually synthesized by aminolysis of activated carboxylic acid derivatives, such as halides, anhydrides, azides, or activated esters, that are mostly generated in an extra step with aggressive, expensive or waste-intensive reagents^14^,^15^,^16^,^17^,^18^,^19^,^20^. The other main strategy for amide bond formation involves the *in situ* activation of carboxylic acids by peptide coupling reagents, such as carbodiimides or phosphonium salts^21^,^22^,^23^,^24^,^25^,^26^,^27^,^28^,^29^,^30^,^31^. Such amide syntheses are highly optimized and provide access to almost any amide structure in near quantitative yields. In modern protein synthesis, they are complemented by efficient chemical and enzymatic peptide ligation methods^32^,^33^,^34^,^35^,^36^,^37^. However, the atom economy of all these processes is low, and the cumulative waste generated during amide synthesis is unacceptable. As a result, the ACS GCI Roundtable has identified ‘amide bond formation avoiding poor atom economy' as the most pressing target for sustainable synthetic method development^38^.

Over the last years, some elegant strategies for waste-minimized amide synthesis have been devised (Fig. 1), for example, dehydrogenative couplings of alcohols, aldehydes or alkynes with amines, or additions of alcohols to nitriles^39^,^40^,^41^,^42^,^43^,^44^,^45^,^46^,^47^,^48^,^49^,^50^,^51^. However, for most synthetic organic chemists, carboxylic acids and amines are still the optimal substrate base for amide synthesis.

To address the central issue of atom economy in the synthesis of amides from ammonium carboxylates, we looked for an activator with minimal molecular weight and low intrinsic reactivity that would scavenge the reaction water in a catalytic condensation process. We envisioned that a hydroacyloxylation catalyst with unprecedented activity might enable the generation of vinyl esters from ammonium carboxylates and gaseous acetylene. Aminolysis of these intermediates would furnish the desired amides along with volatile acetaldehyde.

Ru^II^, Ag^I^ and Au^I^ complexes efficiently promote the addition of carboxylic acids to alkynes under mild conditions, as reported by Mitsudo, Dixneuf, Bruneau and others^52^,^53^,^54^,^55^,^56^,^57^,^58^,^59^. The aminolysis of enol esters takes place under similarly mild conditions^60^,^61^,^62^,^63^. However, for all known catalysts, the two reaction steps are incompatible. As a result, this technology appeared limited to two-step procedures with isolation of sensitive enol esters. For example, Kita *et al*. reported an amide synthesis via isolated ketene acetal intermediates^64^, and Breinbauer *et al*. synthesized polypeptides via a Ru-catalysed hydroacyloxylation of alkynes followed by enzymatic aminolysis^65^. These reactions demonstrate the potential of this concept, giving access to amides in high yields under mild conditions, as demanded especially by peptide chemists. However, this approach can reach synthetic maturity only through a catalytic one-pot process that overcomes all its associated problems, for example, carboxylate salt formation with basic amines which hinders catalytic hydroacyloxylation, the control of hydroamination as a side reaction, and the challenging activation of gaseous acetylene, which state-of-the-art catalysts have not been extending to^66^.

We disclose herein an amidation protocol which allows the use of low-molecular acetylene and its more activated homologue ethoxyacetylene as a sustainable alternative for state-of-the-art coupling agents. These procedures are convincing in terms of the amount, toxicity and separation of the formed byproducts, yet, broadly applicable, convenient and comparable cheap.

## Results

### Development of a one-pot amide synthesis

Evaluation of state-of-the-art catalysts, for example, [Ru(methallyl)_2_dppb] or [RuCl_2_(PPh_3_)(*p*-cymene)]^58^,^67^,^68^,^69^, in the reaction between benzoic acid (**1a)** and 1-hexyne confirmed that they give high yields only in the absence of benzylamine. None of them catalysed the reaction of **1a** with acetylene to give vinyl benzoate (**3a**; Supplementary Tables 4 and 5).

However, we were pleased to find that simple RuCl_3_ catalyses the conversion of benzylammonium benzoate (**6aa**) to the desired *N*-benzyl benzamide in up to 75% yield at 80 °C under acetylene at 1.7 bar, which is its usual tank pressure (Table 1, entry 1). Systematic evaluation of Ru^III^ and Ru^IV^ precursors revealed that **Ru-1** was most effective (entries 2 and 3). Phosphine and nitrogen ligands adversely affected the yield (Supplementary Tables 1 and 2). This is surprising, because the only known Ru^IV^ hydroacyloxylation catalyst is the triphenylphosphine complex reported by Cadierno *et al*.^70^

Dioxane was found to be the best solvent, but the reaction also works well in toluene, THF and ethyl acetate (entries 4−8). The reaction is surprisingly tolerant to oxygen and water up to a certain threshold (Supplementary Table 1).

Under optimal conditions, that is, 2 mol% **Ru-1** or RuCl_3_ in dioxane at 80 °C, the amide forms in near quantitative yield within 6 h with acetylene as the carboxylate activator (Table 1, entry 9, Supplementary Table 1). Higher alkynes are inactive as activators, but with ethoxyacetylene and **Ru-1** as catalyst, full conversion was observed already at 40 °C within 4 h (entries 13−15). Under identical conditions, RuCl_3_ gives only unsatisfactory yields for this activator (Supplementary Table 2). The advantages of the somewhat less atom-economic ethoxyacetylene are that it is more easily handled on small scales than gaseous acetylene, and that inert ethyl acetate rather than acetaldehyde is released.

Both new protocols were compared with two-step procedures using established catalysts^64^, in which the enol esters are formed in a separate step, with consecutive addition of the amine either in the same solvent or after solvent exchange. With acetylene as the activator, no conversion could be achieved, and with ethoxyacetylene, the yields obtained in these two-step syntheses were much lower than those obtained with our convenient one-step protocols (Supplementary Tables 1 and 2).

### Applicability of the developed processes

The scope of the ecologically and economically beneficial acetylene protocol is illustrated in Table 2. Aliphatic, aromatic and heteroaromatic carboxylates were successfully coupled with primary amines. Unfortunately, the substrate scope of this protocol is limited by the solubility of the alkylammonium carboxylates in dioxane, the optimal solvent for acetylene gas.

Such restrictions do not apply to the ethoxyacetylene protocol in the solvent *N*-methyl-2-pyrrolidone, which is applicable to a remarkably wide range of substrates (Table 3). Aromatic, heteroaromatic and aliphatic carboxylic acids reacted with benzylamine to give high yields of the corresponding amides. Diverse functionalities including halo, ether, amide, aldehyde, ester and even-free OH groups were tolerated. Other primary and secondary amines were successfully converted to the corresponding benzamides in good to excellent yields when increasing the temperature to 80 °C to ensure full conversion (Supplementary Table 3). Remarkably, the coupling of less nucleophilic compounds such as amides, aniline and diethylamine with benzoic acid also gave the desired products, albeit in low yields. Other oxygen- or sulfur-based nucleophiles could not be converted.

The synthetic concept may also be used for peptide couplings. Various *N*-protected amino acids were successfully coupled with amino acid esters. Without additives, racemization could not fully be suppressed but remained below 10%, which is a good basis for dedicated optimization.

### Mechanistic considerations

**Figure 2:**
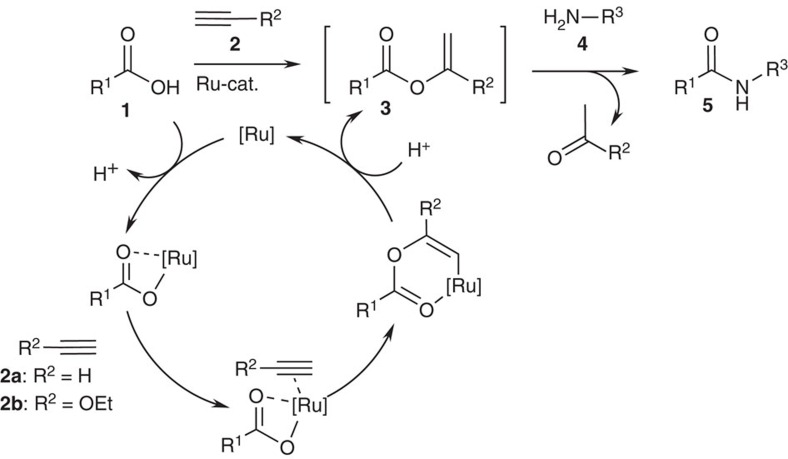
Catalytic amide condensation via enol esters. The proposed catalytic cycle starts with the coordination of a carboxylate and an alkyne to the ruthenium catalyst, followed by an addition of the carboxylate to the alkyne. After protonolysis, the enol ester intermediate is released, which then acts as an acylating agent for the amine, yielding the desired amide along with the carbonyl-byproduct.

The reaction mechanism was investigated by *in situ* nuclear magnetic resonance spectroscopy. The experiments confirmed the intermediacy of enol esters, which formed within minutes and were consumed in the course of the reaction (see Supplementary Table 6 and Supplementary Fig. 1 respectively). We thus conclude that as outlined in Fig. 2, the reaction proceeds via a Ru-catalysed hydroacyloxylation via a standard catalytic cycle^67^,^71^ followed by  aminolysis. In ESI MS investigations of the reaction mixture, species with *m*/*z* values of 754 and 647 were dominant. These were identified as [RuCl_2_(benzyl amine)_3_(ethoxyacetylene)_2_(benzoate)]^+^ and [RuCl_2_(benzyl amine)_2_(ethoxyacetylene)_2_(benzoate)]^+^. In tandem mass spectrometry (MS) experiments, these adducts fragmented with loss of benzyl amine ligands and formation of the six-coordinate [RuCl_2_(benzyl amine)_1_(ethoxyacetylene)_2_(benzoate)]^+^ complex, which we believe to be the catalyst resting state. It is reasonable to assume that it is a Ru(IV)-complex, since it bears three anions and is still positively charged. These investigations suggest the intermediacy of high-valent Ru-species, which explains why Ru^IV^ pecursors have a higher activity than the Ru^II^ and Ru^0^ precursors employed in other catalytic additions. For the details of the spectroscopic investigation, see Supplementary Figs 2–5. In-depth, studies are required to clarify whether the carboxylate addition proceeds via Ru-complexes with η^2^-coordinated alkynes or via Ru-alkylidene complexes.

In conclusion, the feasibility of catalytic amidation reactions with minimal waste production has been demonstrated. Even though extensive optimization is still required, this reaction concept could become an important factor in meeting one of the key challenges of Green Chemistry.

## Methods

For analytical data and preparation methods of the compounds in this article, see Supplementary Figs 6–111 and Supplementary Methods.

### General techniques

All reactions were performed in oven-dried glassware containing a Teflon-coated stirring bar and dry septum under a nitrogen atmosphere. For the exclusion of atmospheric oxygen from the reaction media, solvents were degassed by argon sparge and purified by standard procedures before use. Non-aqueous amines were distilled before use. All reactions were monitored by gas chromatography (GC) using *n*-tetradecane as an internal standard or by high-performance liquid chromatography using anisole as an internal standard. Response factors of the products with regard to *n*-tetradecane/anisole were obtained experimentally by analysing known quantities of the substances. GC analyses were carried out using an HP-5 capillary column (Phenyl Methyl Siloxane 30 m × 320 × 0.25, 100/2.3-30-300/3) and a temperature programme beginning with 2 min at 60 °C followed by 30 °C/min ramp to 300 °C, then 3 min at this temp. High-performance liquid chromatography analyses were carried out using a Shimadzu LC-2010A. The stationary phase was a reversed phase column LiChroCart PAH C-18 from *Merck KGaA* with acetonitrile and water as eluents at 60 °C and the following solvent programme: starting from 10 vol% acetonitrile for 1 min, followed by increasing acetonitrile to 70 vol% during 23 min, then decreasing again to 10 vol% rapidly and maintaining this value for the next 2 min. Column chromatography was performed using a Combi Flash Companion-Chromatography-System (Isco-Systems) and Redi*Sep* packed columns (12 g). nuclear magnetic resonance spectra were obtained on Bruker AMX 400 or on Bruker Avance 600 systems using DMSO-*d*_*6*_, Chloroform-*d*_*3*_ or Toluene-*d*_*8*_ as solvent, with proton and carbon resonances at 400/600 MHz and 101/151 MHz, respectively. Mass spectral data were acquired on a GC-MS Saturn 2,100 T (Varian). Infrared spectra were recorded on Perkin Elmer Spectrum 100 FT-IR Spectrometer with Universal ATR Sampling Accessory. Melting points are uncorrected and were measured on a Mettler FP 61. ESI MS data were acquired on a Bruker Esquire 6,000 and evaluated with mMass software. Sample solutions at concentrations of ∼1 × 10^−4^ M were continuously infused into the ESI chamber at a flow rate of 2 μl min^−1^ using a syringe pump. We use nitrogen as drying gas at a flow rate of 3.0–4.0 l min^−1^ at 300 °C and spray the solutions at a nebulizer pressure of 4 psi with the electrospray needle held at 4.5 kV. CHN-elemental analyses were performed with a Hanau Elemental Analyzer vario Micro cube and HRMS with a Waters GCT Premier.

### Synthesis of amides using acetylene as activator

An oven-dried headspace vial with Teflon-coated stirring bar was charged with the corresponding ammonium carboxylate (1 mmol) and dichloro[(2,6,10-dodecatriene)-1,12-diyl]ruthenium (5.06 mg, 20 μmol). The atmosphere was changed three times with nitrogen, then *N*-methylpyrrolidone (1 ml) and the corresponding amine (0.5 mmol) were added. The vial was placed in an autoclave reactor, the atmosphere was changed twice with acetylene, and a pressure of 1.7 bar was set. The mixture was then heated to 80 °C for 6 h. After cooling down to room temperature, the mixture was diluted with 20 ml of ethyl acetate and washed with each 20 ml of saturated NaHCO_3_ solution, water and brine. The organic layer was dried with MgSO_4_, the solvent removed under reduced pressure and the residue purified by column chromatography (SiOH, ethyl acetate/cyclohexane gradient).

### Synthesis of amides using ethoxyacetylene as activator

An oven-dried headspace vial with Teflon-coated stirring bar was charged with the corresponding carboxylic acid (1 mmol) and dichloro[(2,6,10-dodecatriene)-1,12-diyl]ruthenium (5.06 mg, 15.0 μmol). The atmosphere was changed three times with nitrogen, then *N*-methylpyrrolidone (2 ml), benzyl amine (164 mg, 167 μl, 1.5 mmol) and ethoxyacetylene (40 wt%-solution in hexane; 210 mg, 299 μl and 1.5 mmol) were added in this order. The mixture was then heated to 40 °C for 4 h. After cooling down to room temperature, the mixture was diluted with 20 ml of ethyl acetate and washed with each 20 ml of sat. NaHCO_3_ solution, water and brine. The organic layer was dried with MgSO_4_, the solvent removed under reduced pressure and the residue purified by column chromatography (SiOH, ethyl acetate/cyclohexane gradient).

### Data availability

The authors declare that the data supporting the findings of this study are available within the article and its supplementary information files.

## Additional information

**How to cite this article:** Krause, T. *et al*. Atom-economic catalytic amide synthesis from amines and carboxylic acids activated *in situ* with acetylenes. *Nat. Commun.* 7:11732 doi: 10.1038/ncomms11732 (2016).

## Supplementary Material

Supplementary Information Supplementary Figures 1-111, Supplementary Tables 1-6, Supplementary Methods and Supplementary References

## Figures and Tables

**Figure 1:**
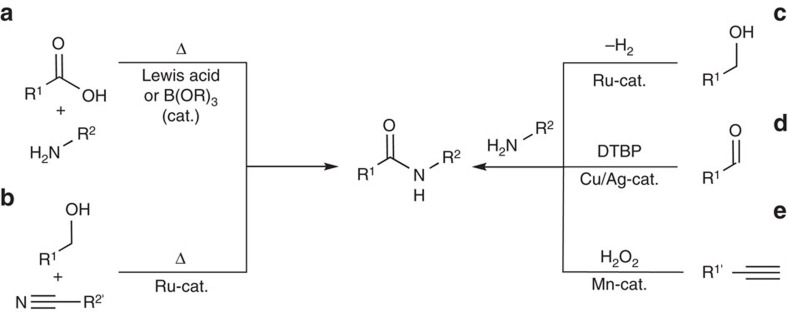
Atom-efficient approaches to amide bond formation. (**a**) Thermal or Lewis acid-mediated dehydration of ammonium carboxylates. (**b**) Catalytic addition of alcohols to nitriles. (**c**) Dehydrogenative coupling of alcohols with amines. (**d**) Oxidative coupling of aldehydes and amines. (**e**) Oxidative coupling of alkynes and amines.

**Table 1:**
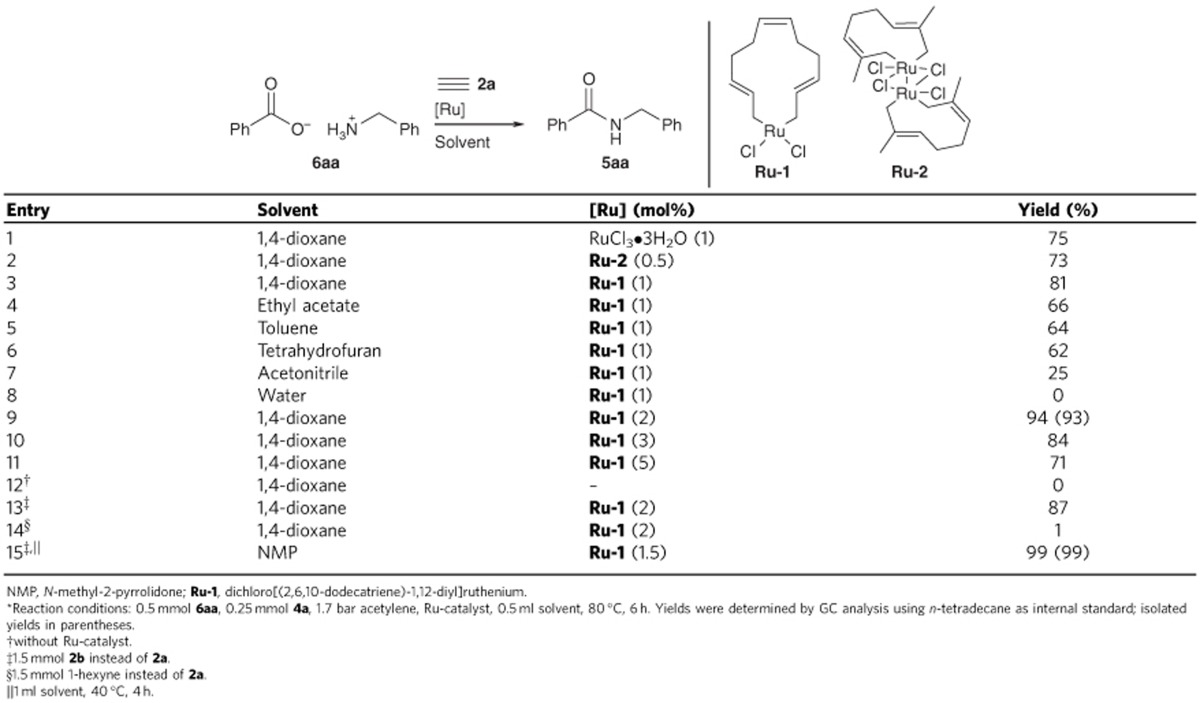
One-pot activation and amidation of carboxylic acids with acetylene^*^.

**Table 2:**
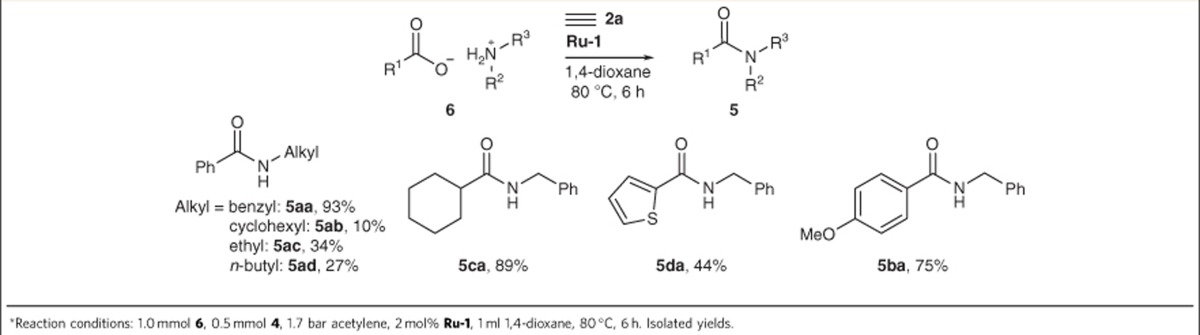
Scope of the amidation with acetylene as the activating agent^*^.

**Table 3:**
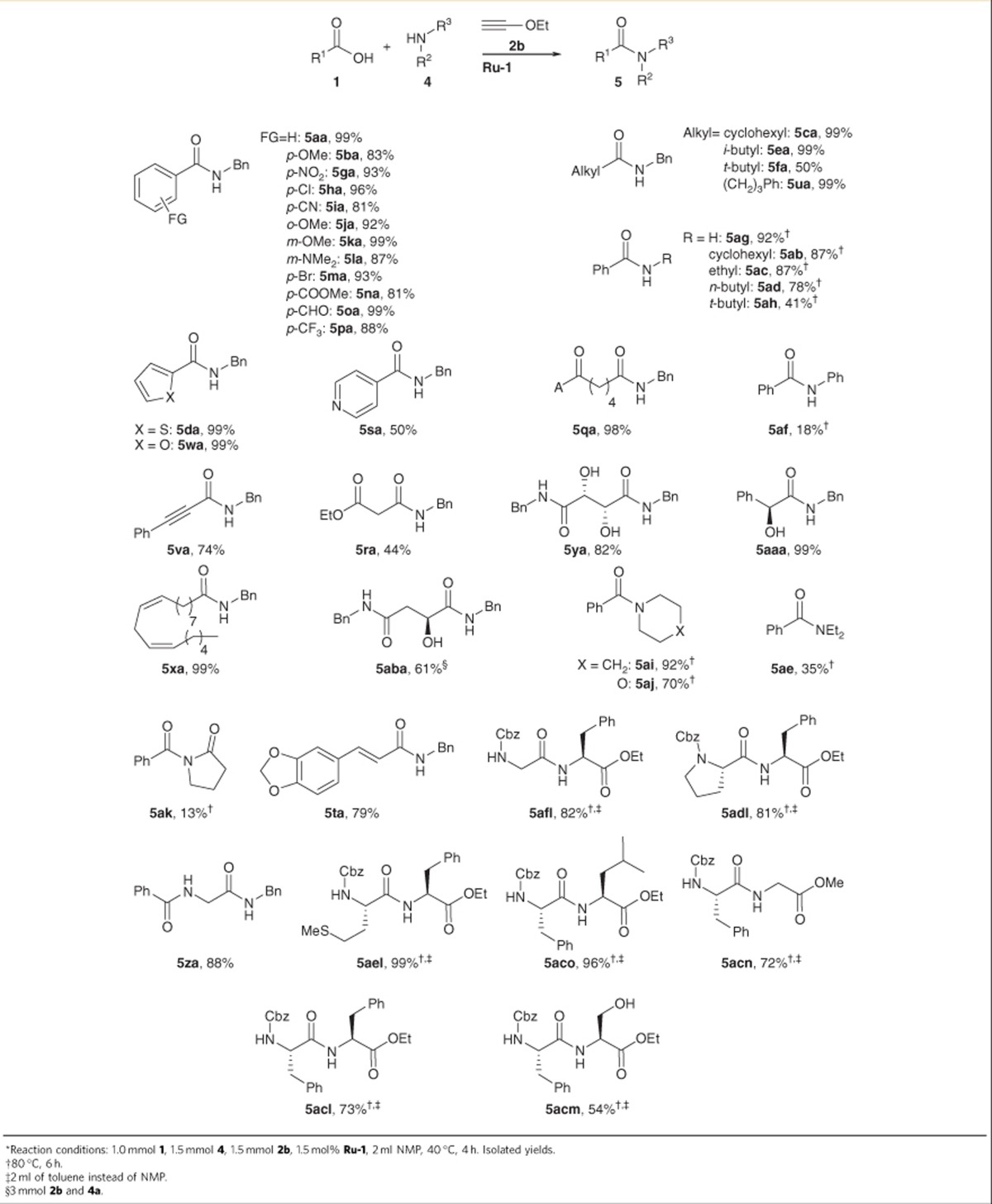
Scope of the amidation with ethoxyacetylene as activating agent^*^.
