# Supplementary material for: Atom-economic catalytic amide synthesis from amines and carboxylic acids activated in situ with acetylenes
Source: Nat Commun. 2016 Jun 10;7:11732. doi: 10.1038/ncomms11732 (PMC4906407; doi:10.1038/ncomms11732)
Supplement: Supplementary Information — Supplementary Figures 1-111, Supplementary Tables 1-6, Supplementary Methods and Supplementary References [file ncomms11732-s1.pdf]

Supplementary Figures

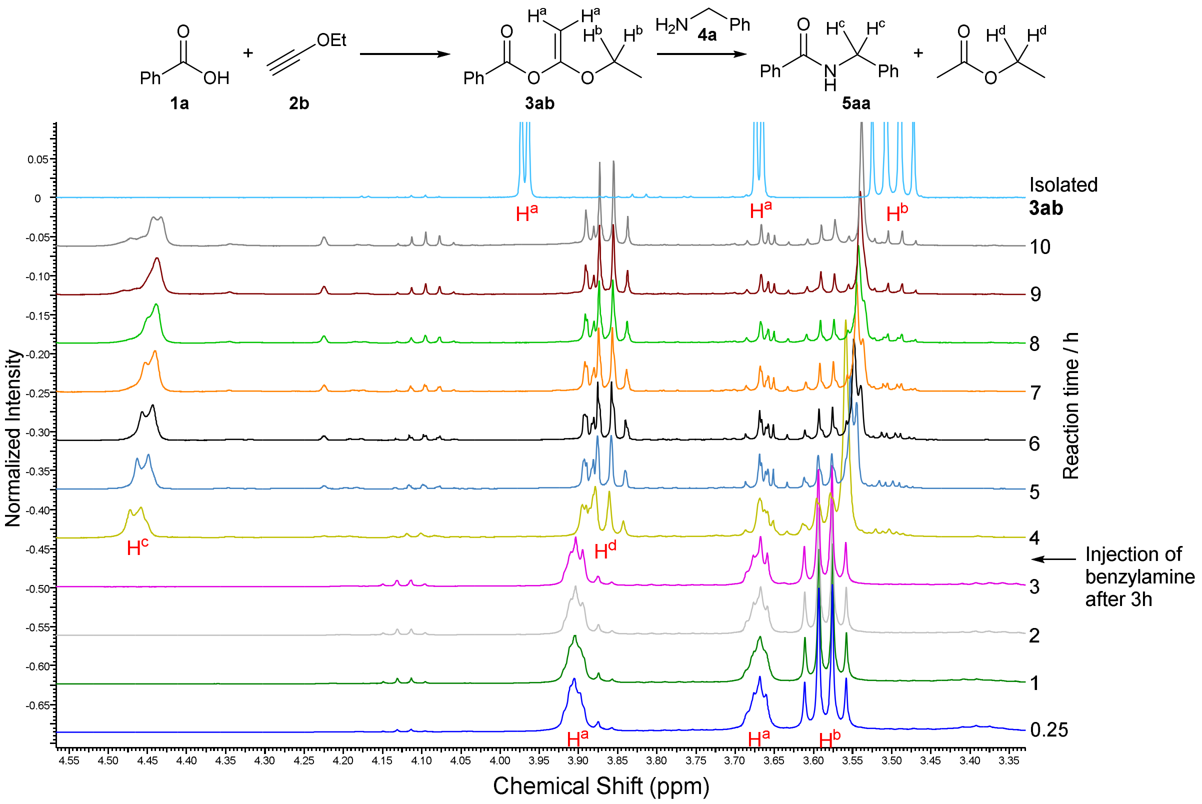

Supplementary Figure 1. Time dependent NMR measurements: An oven-dried headspace vial was charged with benzoic acid (61.7 mg, 0.5 mmol) and dichloro[(2,6,10-dodecatriene)-1,12-diyl]ruthenium (1.70 mg, 5.00 µmol). The atmosphere was changed three times with nitrogen, then toluene-d_8 (0.5 ml), triethylamine (72.8 mg, 0.71 mmol, 100 µl) and ethoxyacetylene (40 wt.%-solution in hexane) (105 mg, 150 µl, 0.75 mmol) were added via syringe in this order. The mixture was transferred into an inerted NMR tube which was placed in a 40 °C tempered NMR spectrometer. After 3 h, benzylamine (81.2 mg, 1.5 mmol, 82.8 µl) was added via syringe. The formation of the enol ester proceeds within 15 minutes. After addition of the benzylamine, the signals of intermediate **3ab** diminish while those of the product N-benzyl benzamide **5aa** appear.

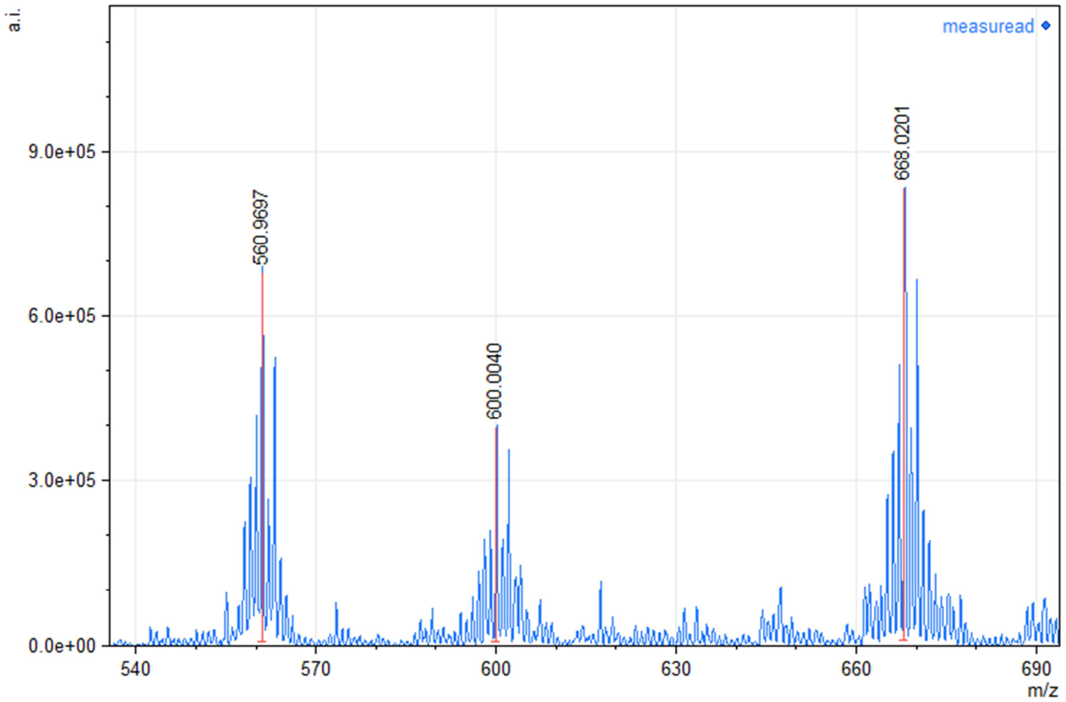

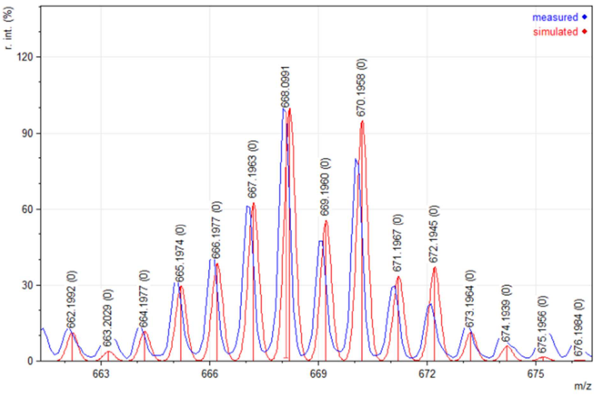

[Ru^{IV}Cl$_2$(C$_{12}$H$_{18}$)(C$_7$H$_9$N)(C$_4$H$_8$O)$_2$(C$_2$H$_3$N)$_2$H]$^+$

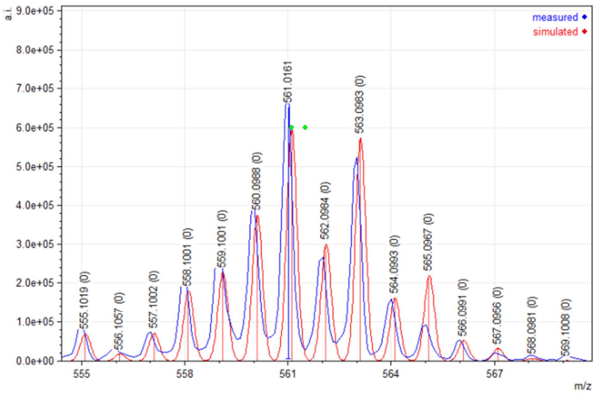

[Ru^{IV}Cl$_2$(C$_{12}$H$_{18}$)(C$_4$H$_8$O)$_2$(C$_2$H$_3$N)$_2$H]$^+$

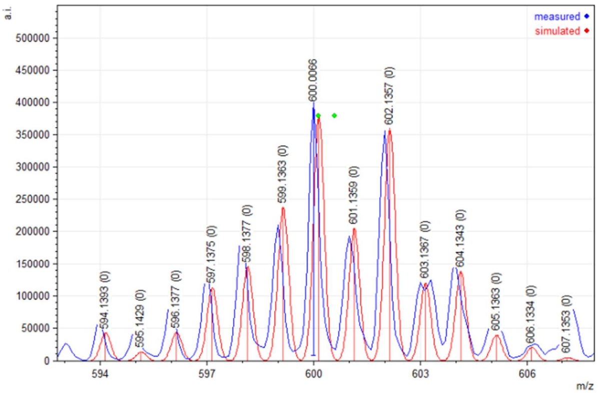

[Ru^{III}Cl$_2$(C$_7$H$_9$N)$_4$]$^+$

Supplementary Figure 2. To a solution of dichloro[(2,6,10-dodecatriene)-1,12-diyl]ruthenium (2.5 mg, 7.5 µmol) in NMP was added benzyl amine (10 µl, 90.5 µmol). The resulting solution was stirred for 5 min at 40 °C, then a 10 µl sample was taken, diluted with tetrahydrofuran and injected into ESI MS.

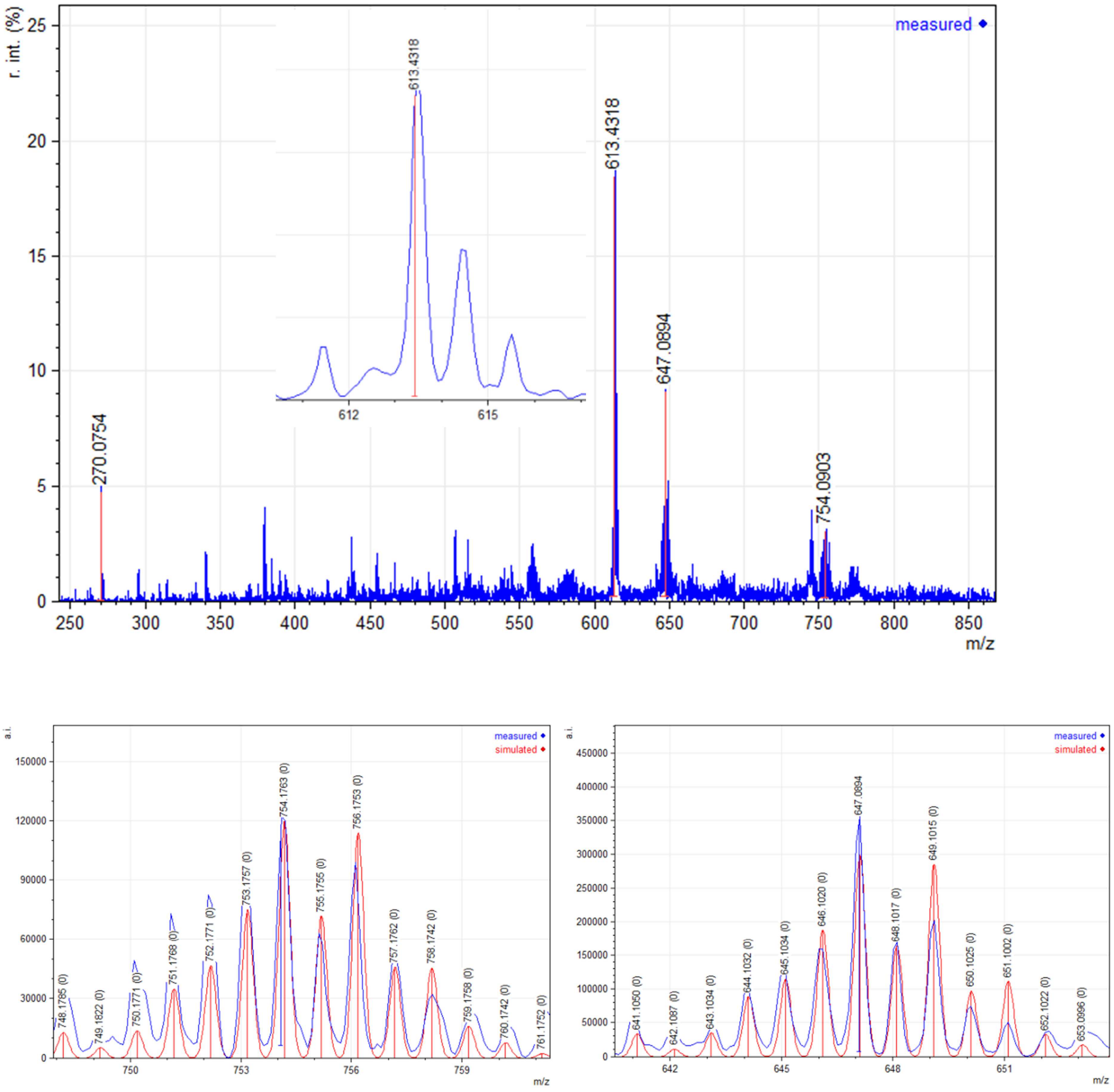

$[Ru^{IV}Cl_2(C_7H_9N)_3(C_4H_6O)_2(C_7H_5O_2)]^+$ $[Ru^{IV}Cl_2(C_7H_9N)_2(C_4H_6O)_2(C_7H_5O_2)]^+$

Supplementary Figure 3. A reaction mixture was prepared analogous to general procedure B. The resulting solution was stirred for 10 min at 40 °C. Then a 10 µl sample was taken, diluted with acetonitrile and injected into ESI MS.

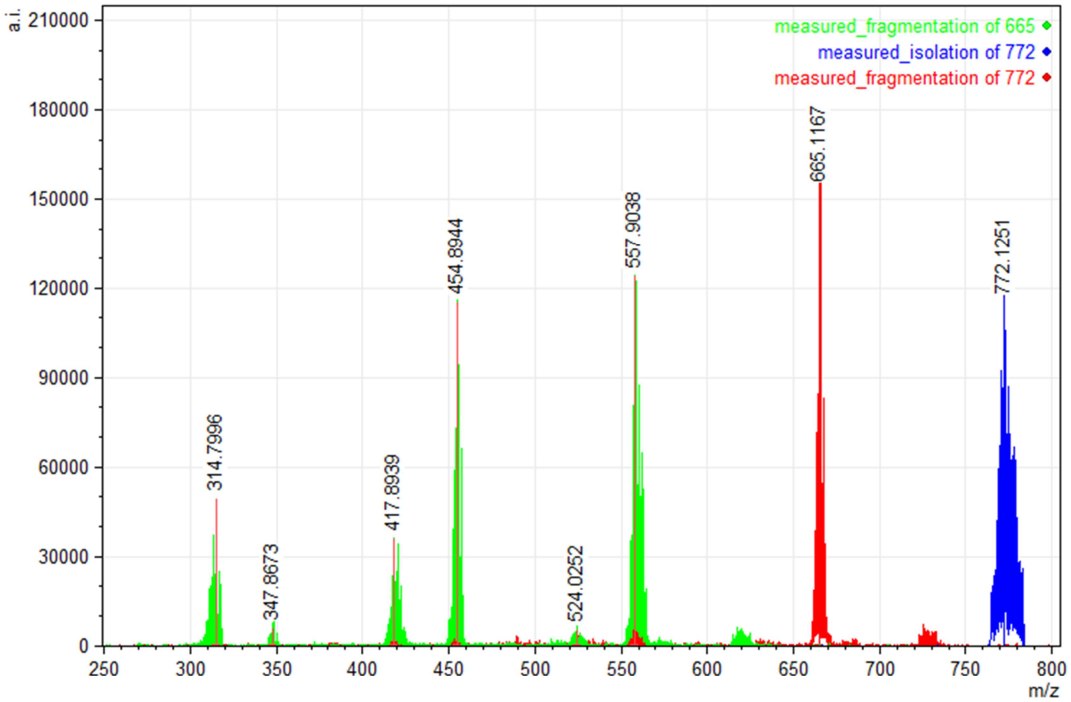

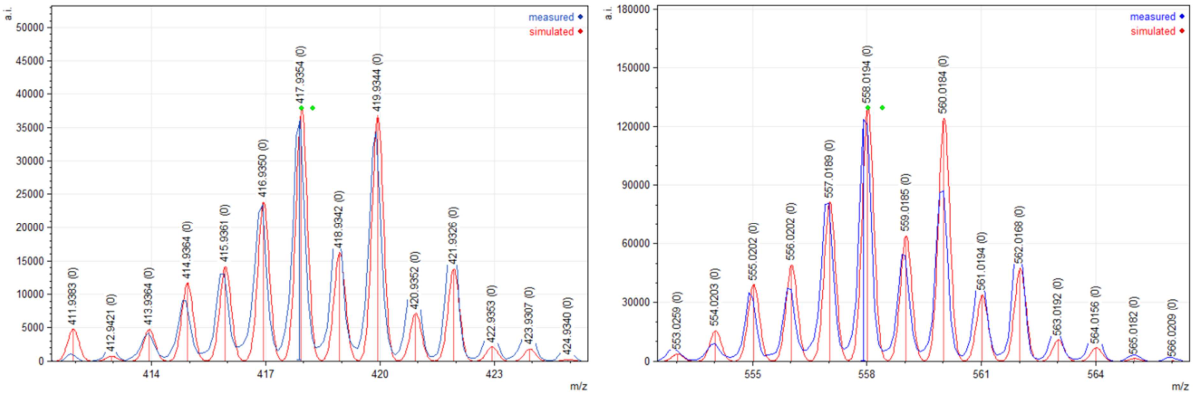

[Ru^{IV}Cl$_2$(C$_7$H$_9$N)(C$_7$H$_4$FO$_2$)]$^+$

[Ru^{IV}Cl$_2$(C$_7$H$_9$N)(C$_4$H$_6$O)$_2$(C$_7$H$_4$FO$_2$)]$^+$

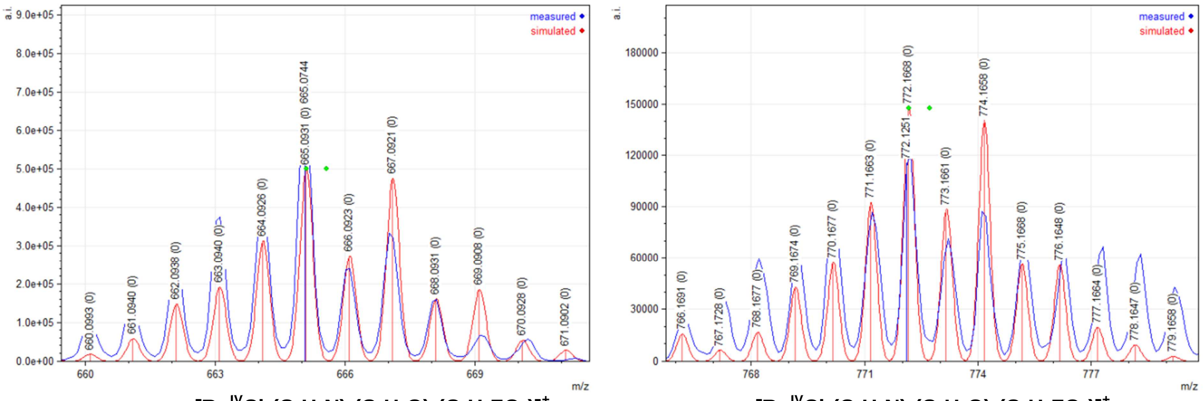

[Ru^{IV}Cl$_2$(C$_7$H$_9$N)$_2$(C$_4$H$_6$O)$_2$(C$_7$H$_4$FO$_2$)]$^+$

[Ru^{IV}Cl$_2$(C$_7$H$_9$N)$_3$(C$_4$H$_6$O)$_2$(C$_7$H$_4$FO$_2$)]$^+$

Supplementary Figure 4. A reaction mixture was prepared analogous to general procedure B with 4-fluoro benzoic acid instead of benzoic acid. The resulting solution

was stirred for 10 min at 40 °C. Then a 10 µl sample was taken, diluted with acetonitrile and injected into ESI MS.

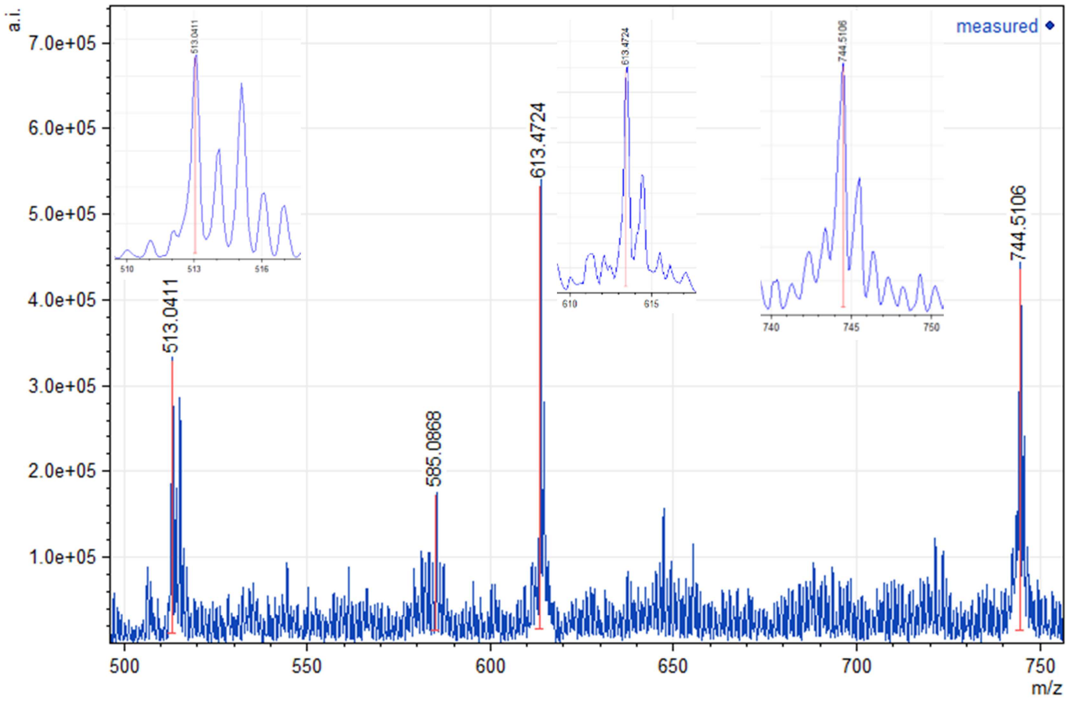

Supplementary Figure 5. To a solution prepared analogous to general procedure B. The resulting solution was stirred for 4 h at 40 °C. Then a 10 µl sample was taken, diluted with acetonitrile (C_2H_3N) and injected into ESI MS.

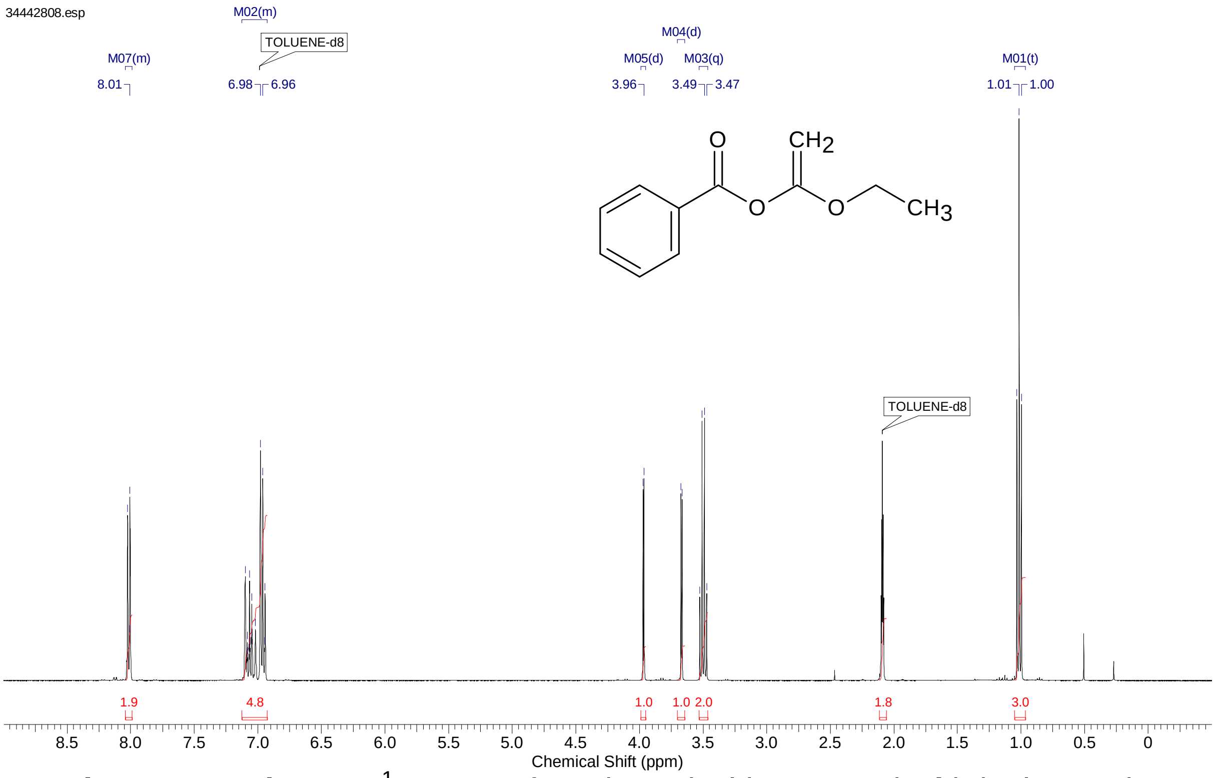

Supplementary Figure 6. ^{1}H NMR of 1-Ethoxyvinyl benzoate (**3ab**) (Toluene-d_8. 400 MHz).

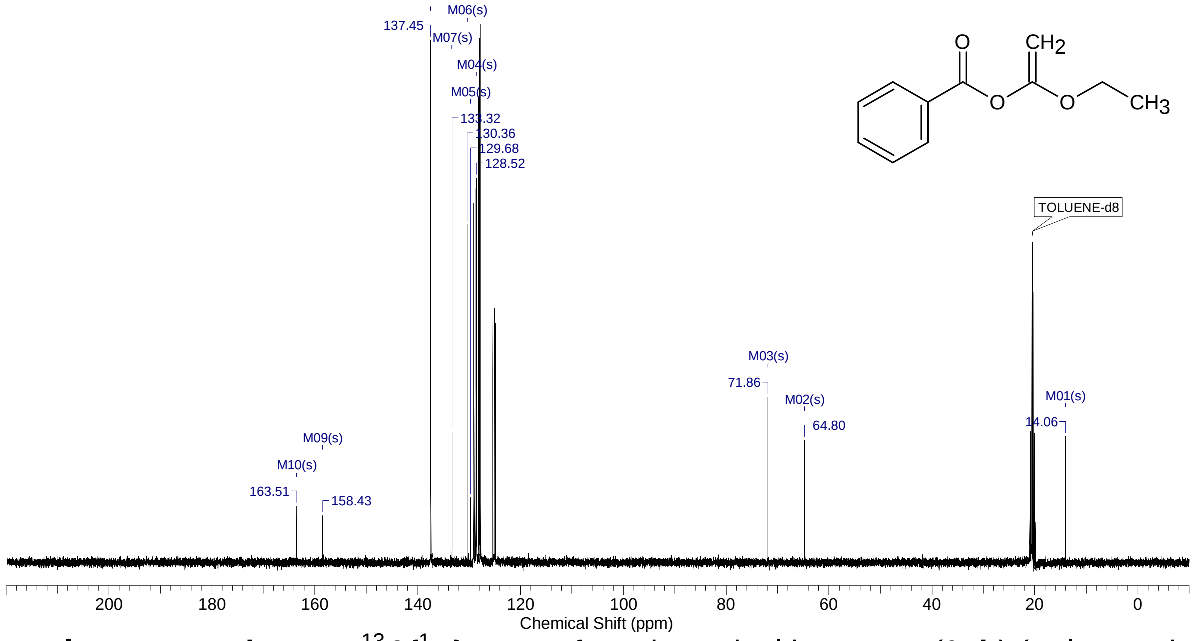

Supplementary Figure 7. ^{13}C{^{1}H} NMR of 1-Ethoxyvinyl benzoate (**3ab**) (Toluene-d_8. 400 MHz).

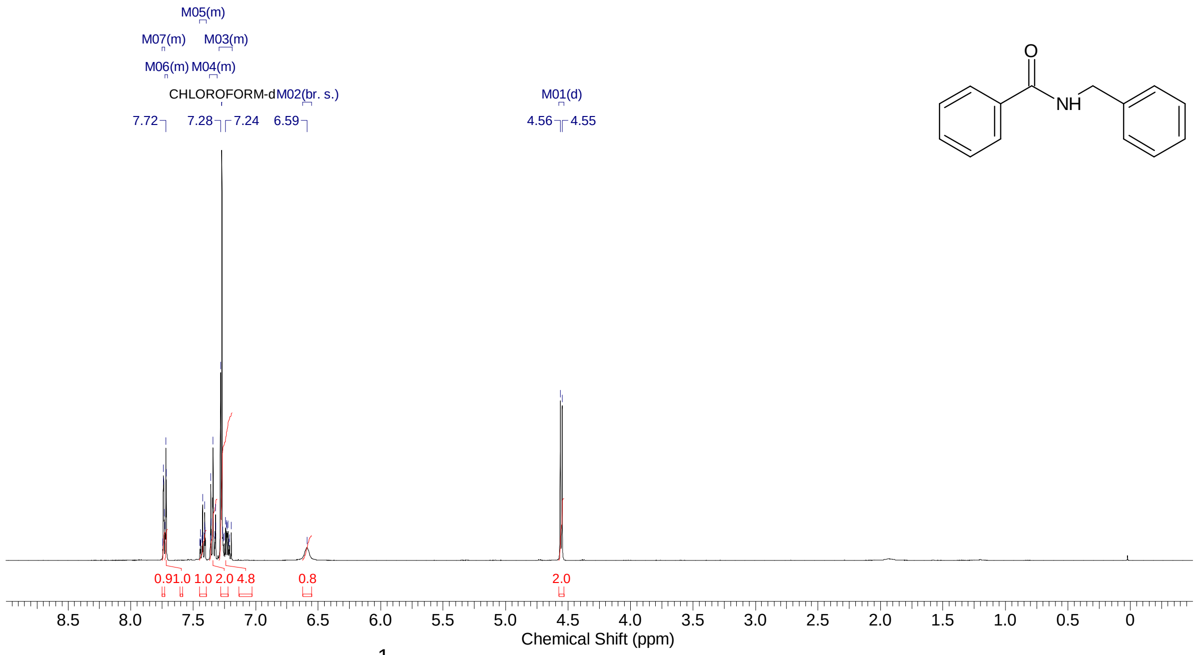

Supplementary Figure 8. ^{1}H NMR of *N*-benzylbenzamide (**5aa**) (Chloroform-d_3, 400 MHz).

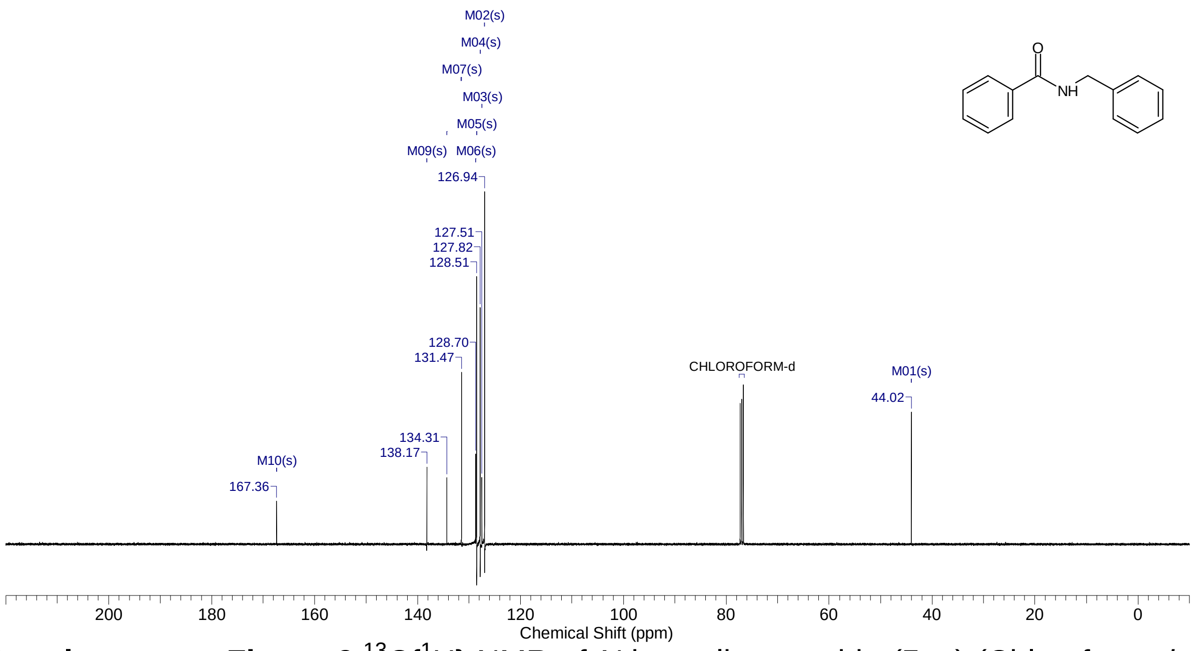

Supplementary Figure 9. $^{13}C\{^{1}H\}$ NMR of *N*-benzylbenzamide (**5aa**) (Chloroform-d_3. 400 MHz).

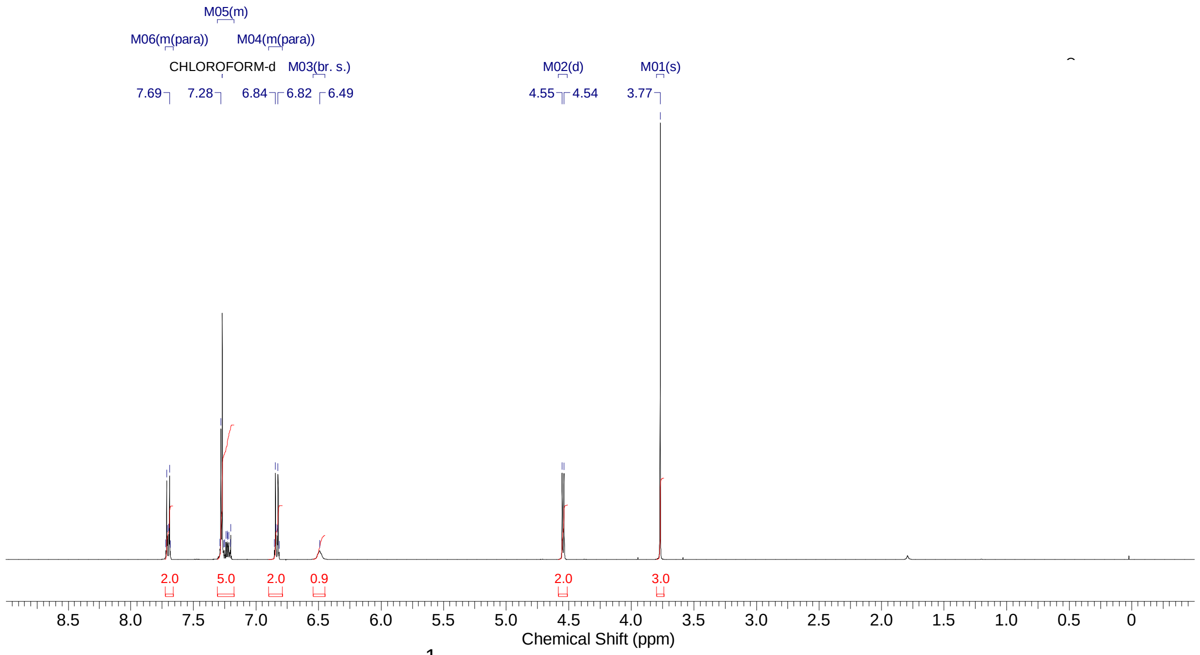

Supplementary Figure 10.^{1}H NMR of *N*-benzyl-4-methoxybenzamide (**5ba**) (Chloroform-d_3, 400 MHz).

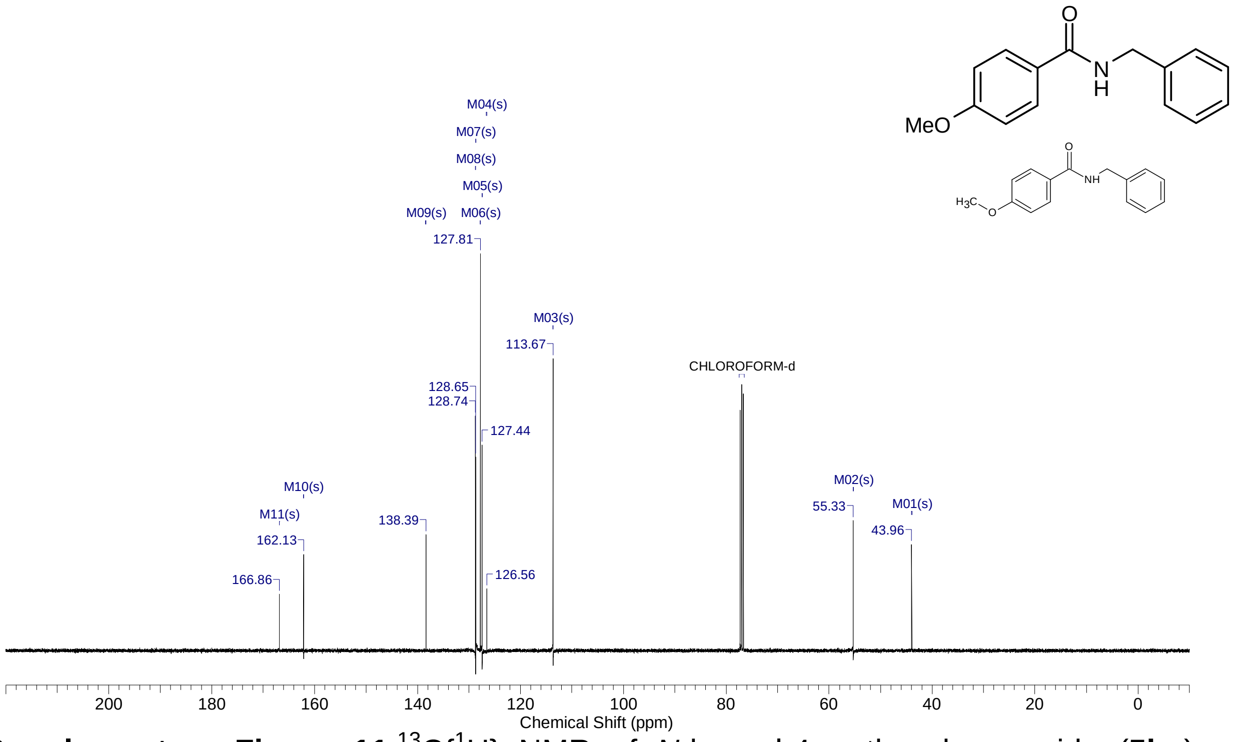

Supplementary Figure 11.^{13}C{^{1}H} NMR of *N*-benzyl-4-methoxybenzamide (**5ba**) (Chloroform-d_3. 400 MHz).

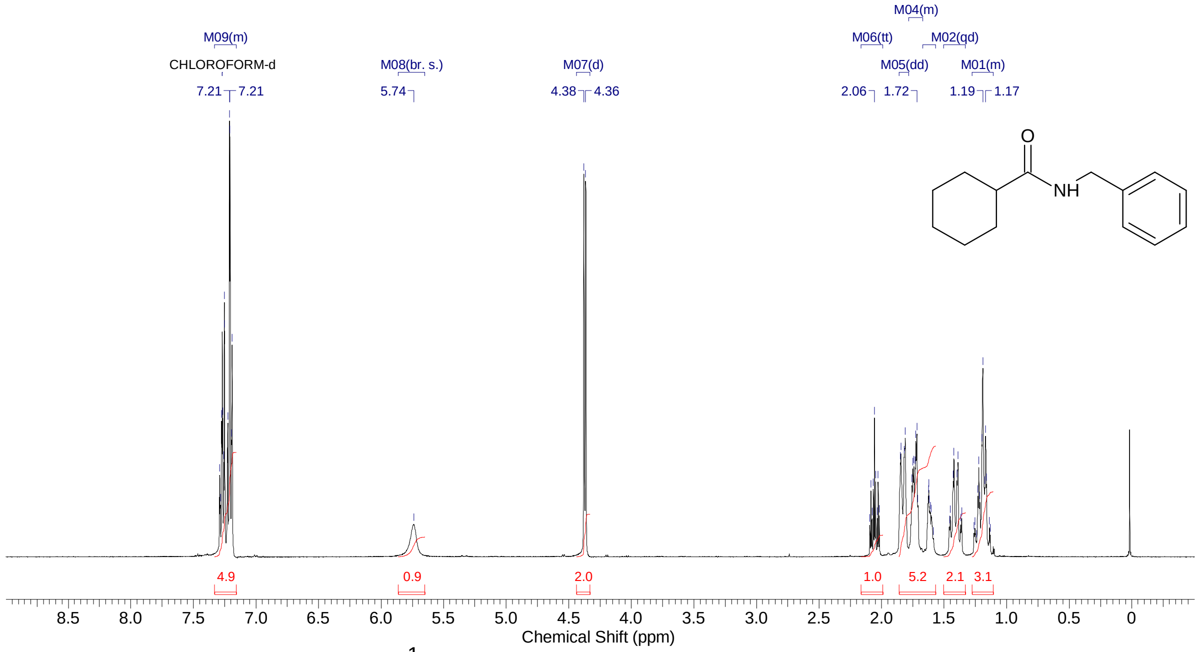

Supplementary Figure 12. ^{1}H NMR of *N*-(phenylmethyl)-cyclohexanecarboxamide (**5ca**) (Chloroform-d_3, 400 MHz).

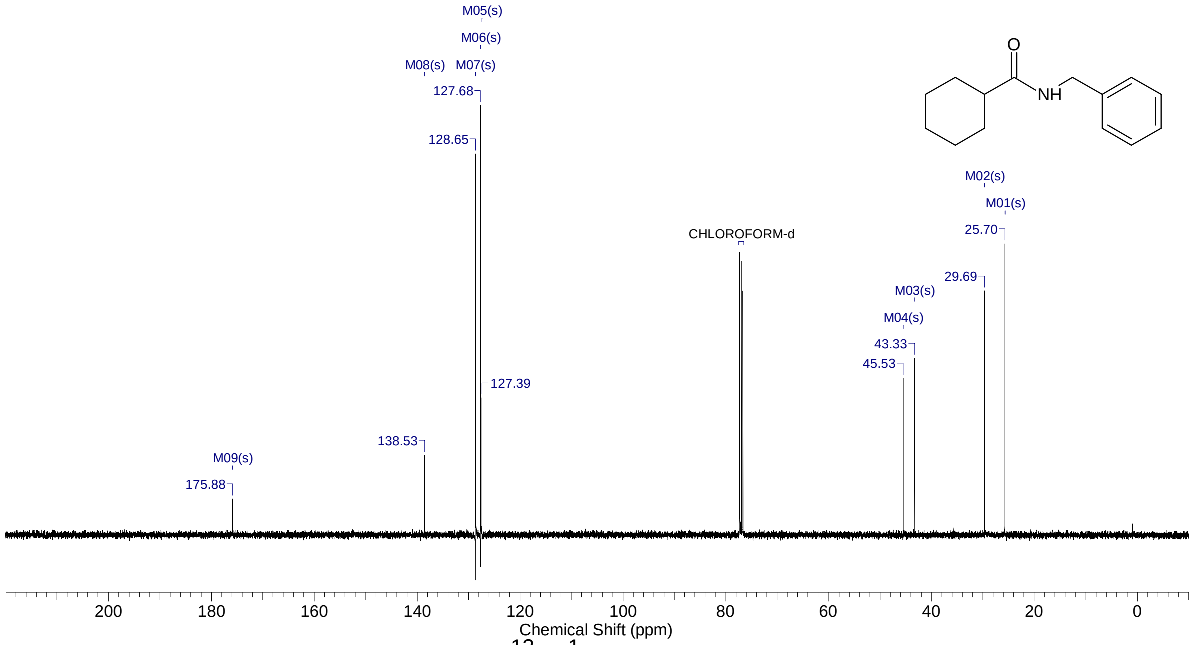

Supplementary Figure 13. $^{13}C\{^{1}H\}$ NMR of *N*-(phenylmethyl)-cyclohexanecarboxamide (**5ca**) (Chloroform-d_3. 400 MHz).

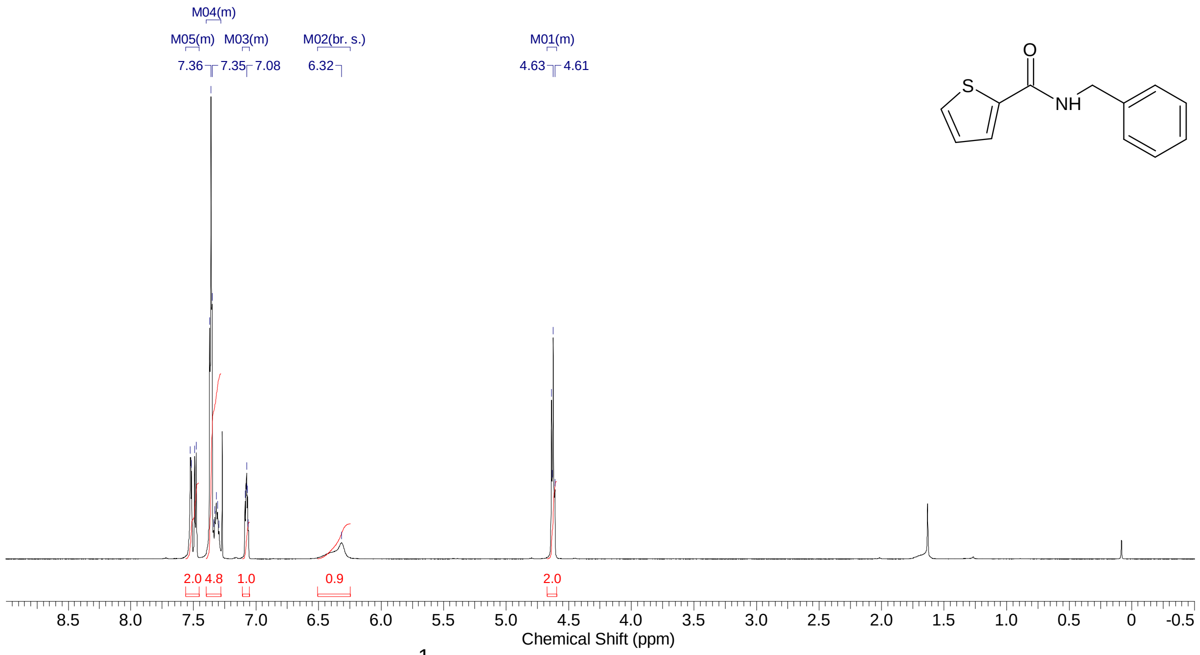

Supplementary Figure 14. ^{1}H NMR of thiophene-2-carboxylic acid benzylamide (**5da**) (Chloroform-d_3, 400 MHz).

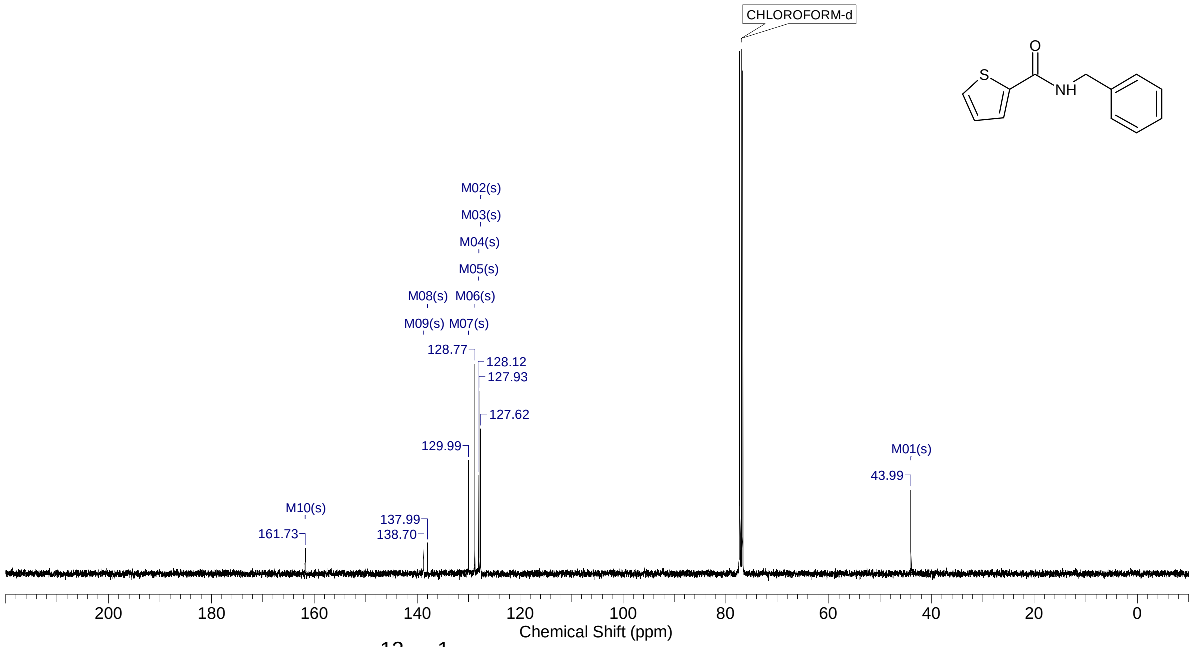

Supplementary Figure 15.$^{13}C\{^{1}H\}$ NMR of thiophene-2-carboxylic acid benzylamide (**5da**) (Chloroform-d_3. 400 MHz).

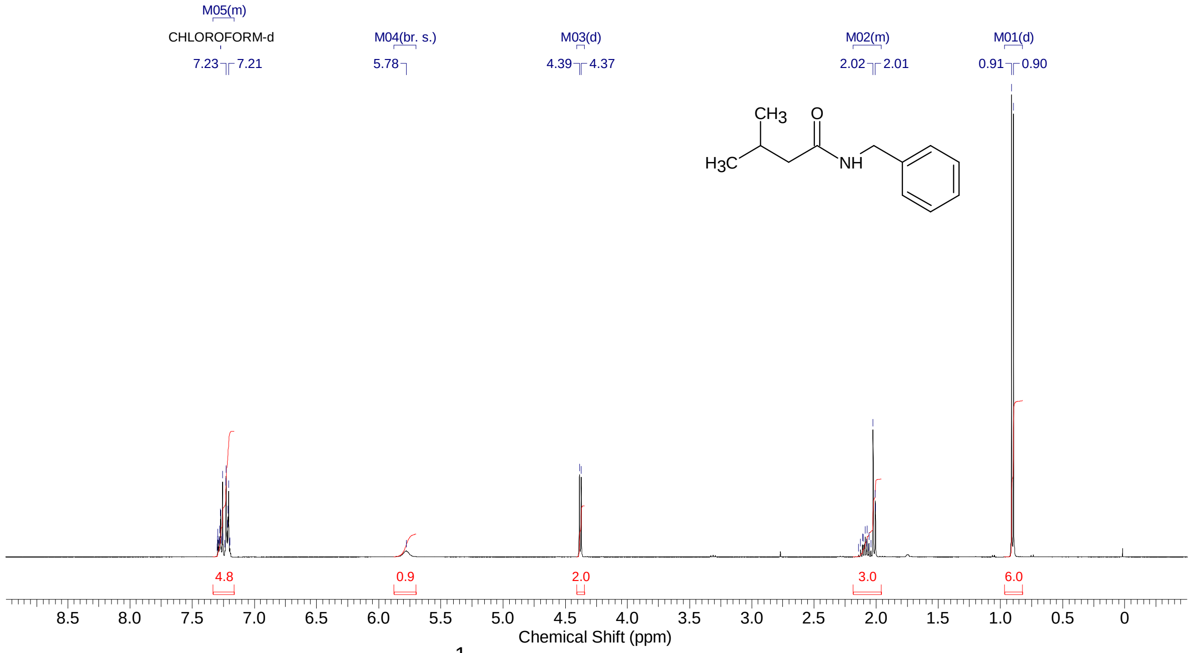

Supplementary Figure 16. ^{1}H NMR of 3-methyl-*N*-benzylbutyramide (**5ea**) (Chloroform-d_3, 400 MHz).

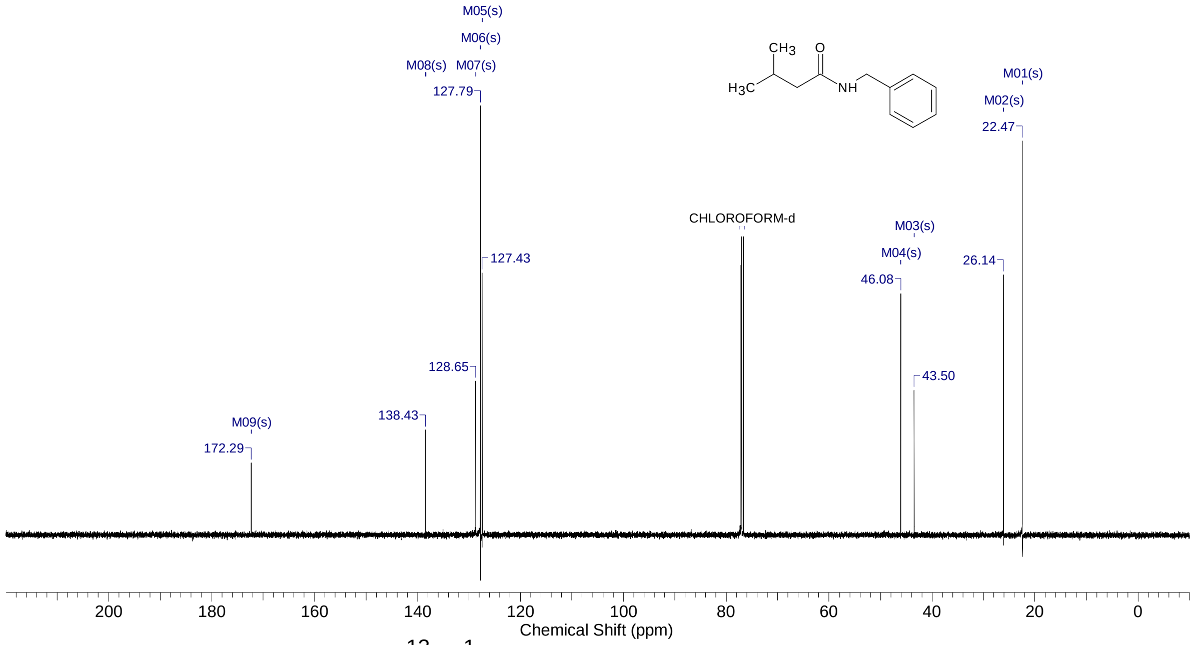

Supplementary Figure 17. $^{13}C\{^{1}H\}$ NMR of 3-methyl-*N*-benzylbutyramide (**5ea**) (Chloroform-d_3. 400 MHz).

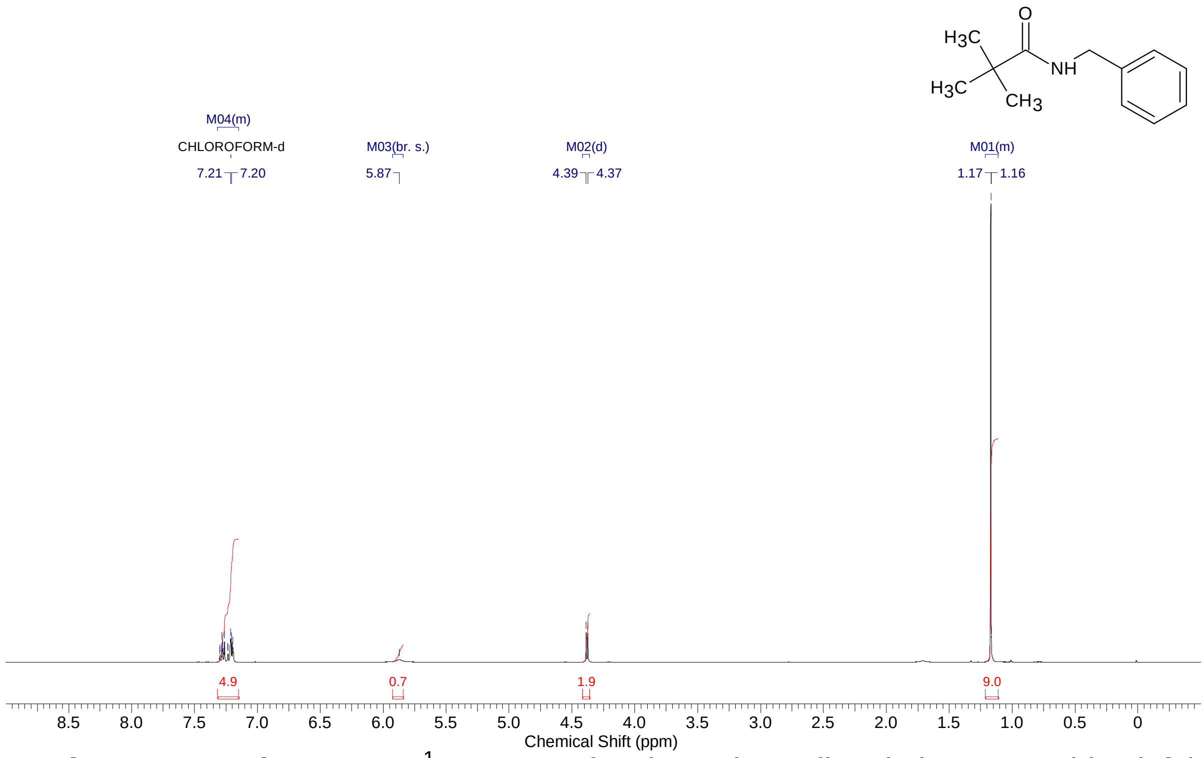

Supplementary Figure 18. ^{1}H NMR of *N*-benzyl-2,2-dimethylpropanamide (**5fa**) (Chloroform-d_3, 400 MHz).

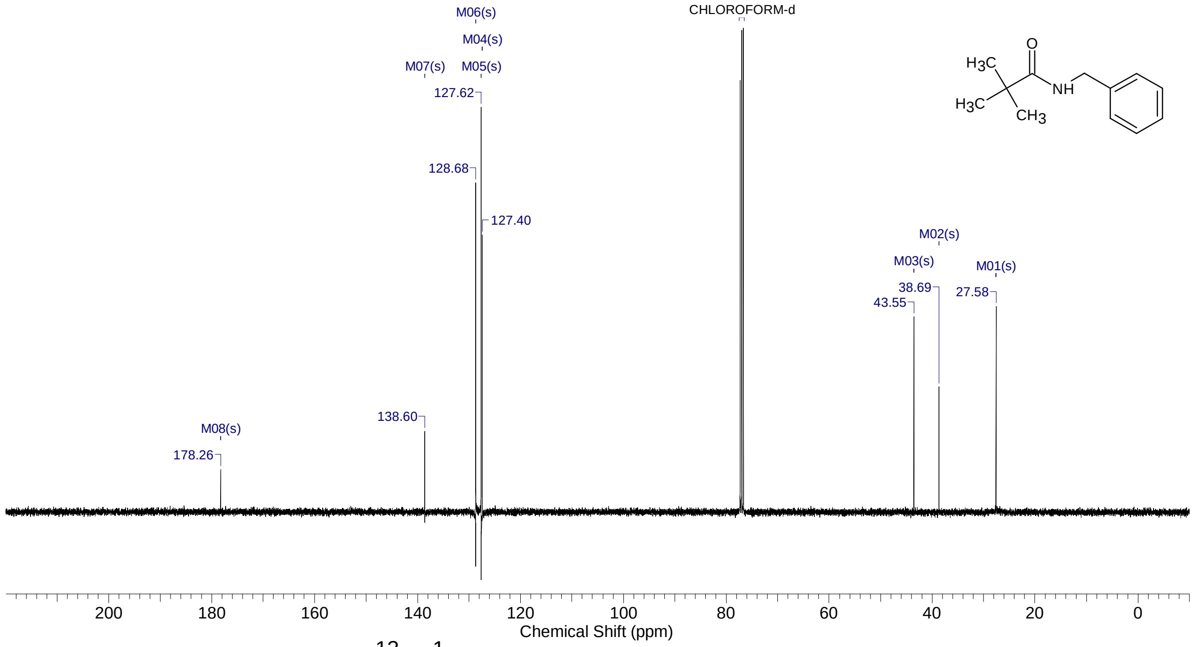

Supplementary Figure 19. $^{13}C\{^{1}H\}$ NMR of (Chloroform-d_3. 400 MHz).

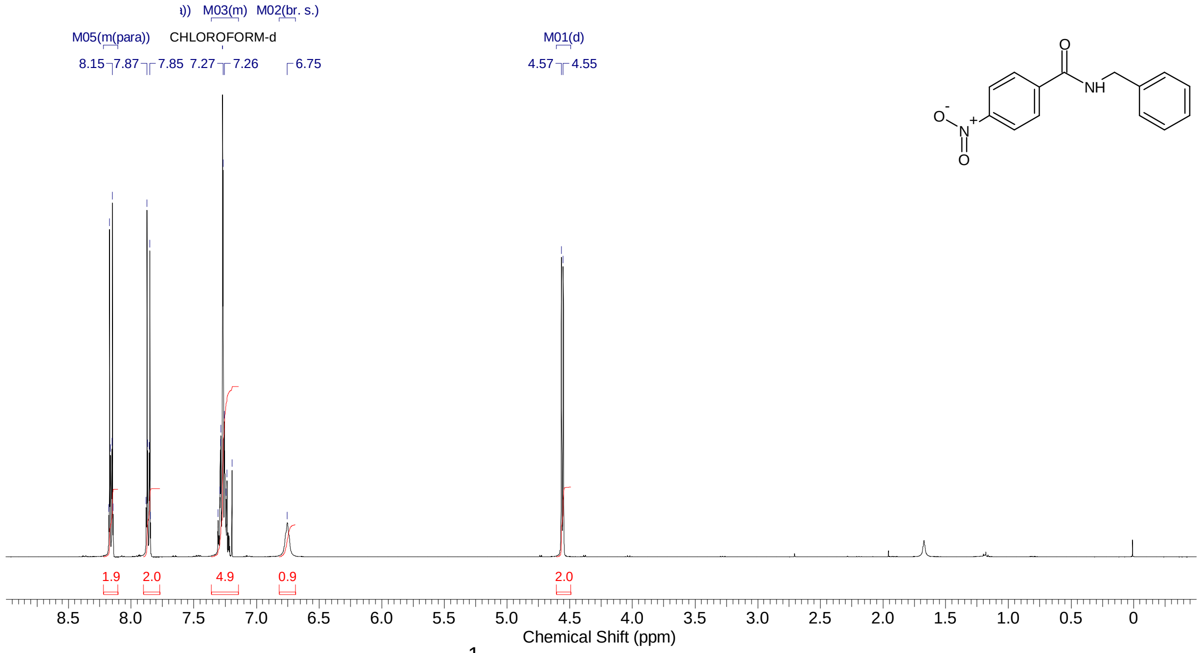

Supplementary Figure 20. ^1H NMR of *N*-benzyl-4-nitrobenzamide (**5ga**) (Chloroform-d_3, 400 MHz).

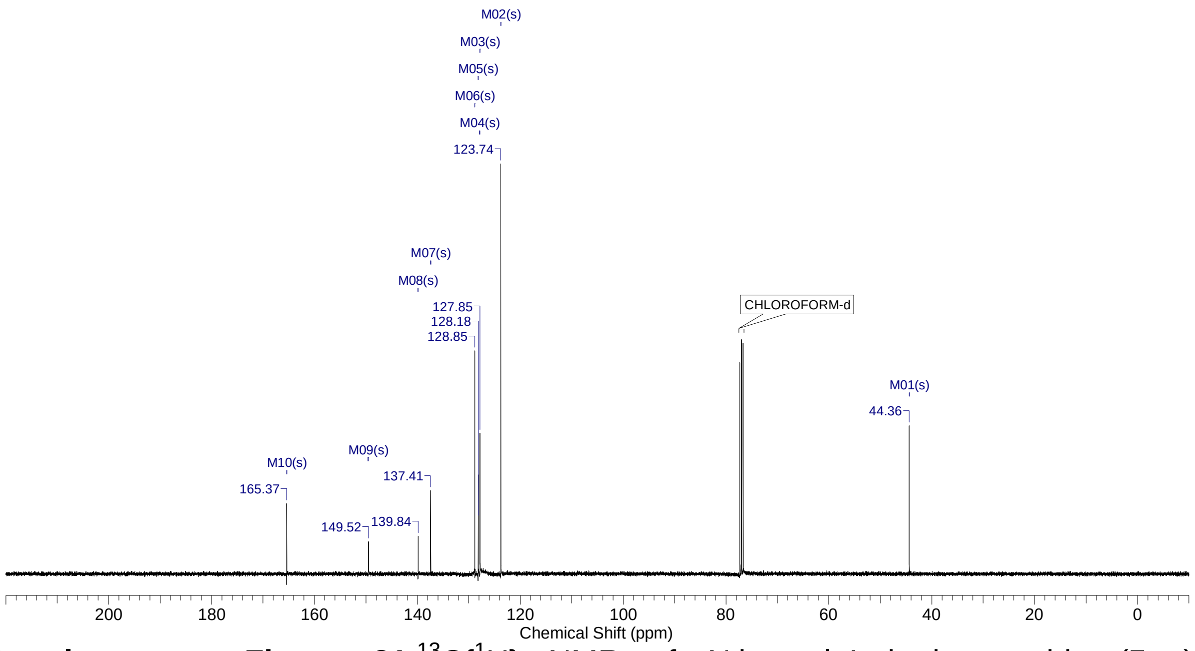

Supplementary Figure 21.$^{13}C\{^1H\}$ NMR of *N*-benzyl-4-nitrobenzamide (**5ga**) (Chloroform-d_3. 400 MHz).

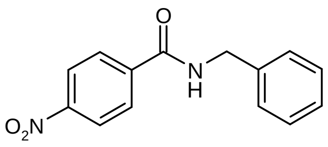

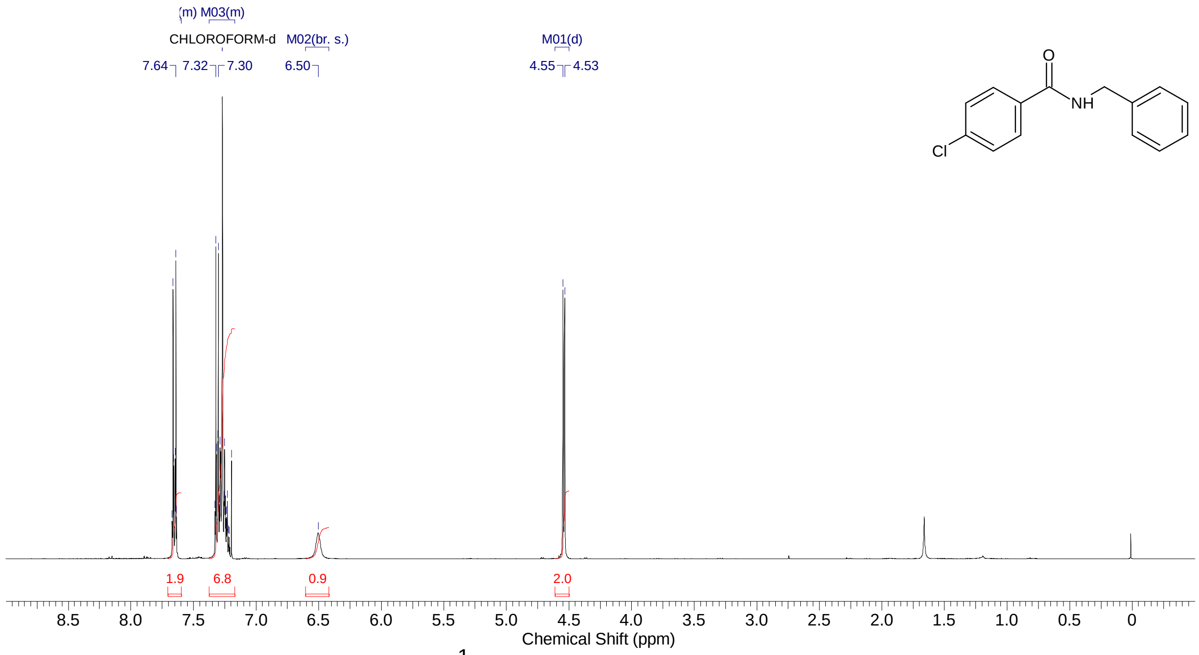

Supplementary Figure 22. ^{1}H NMR of *N*-benzyl-4-chlorobenzamide (**5ha**) (Chloroform-d_3, 400 MHz).

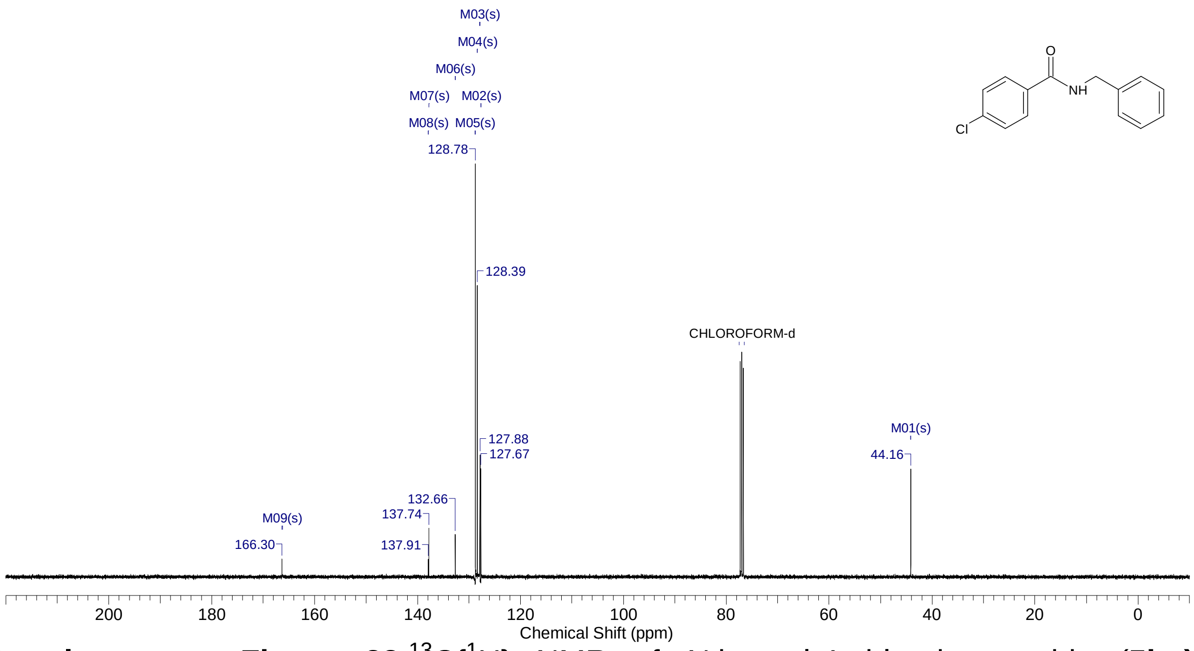

Supplementary Figure 23.^{13}C{^{1}H} NMR of *N*-benzyl-4-chlorobenzamide (**5ha**) (Chloroform-d_3. 400 MHz).

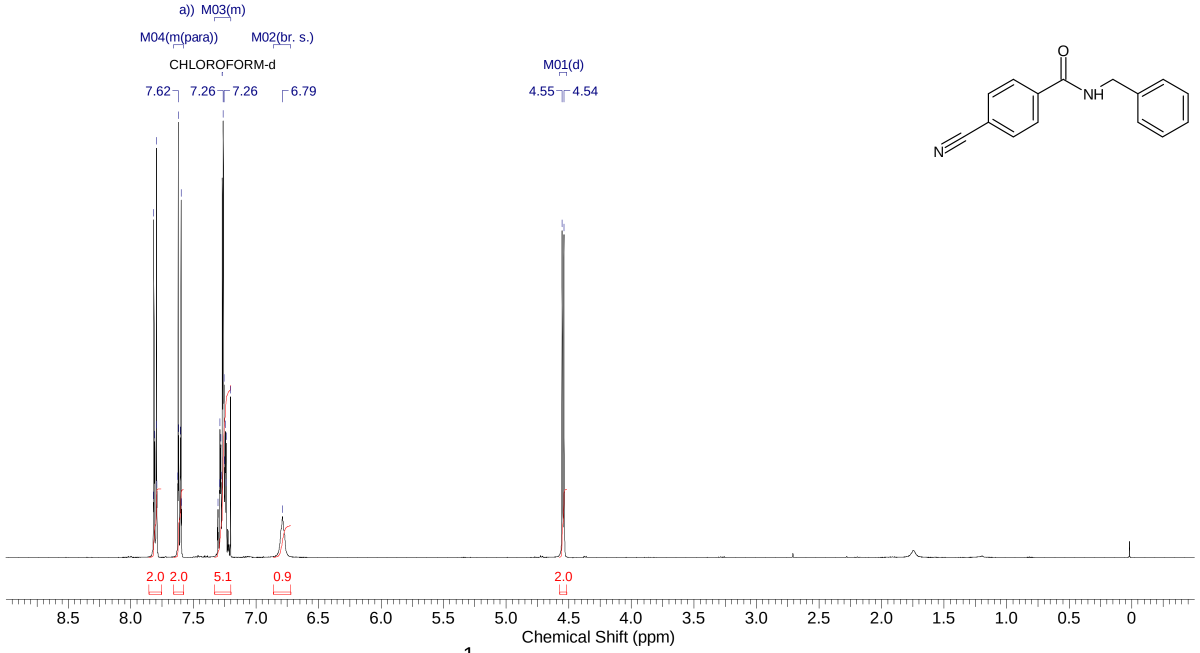

Supplementary Figure 24. ^{1}H NMR of *N*-benzyl-4-cyanobenzamide (**5ia**) (Chloroform-d_3, 400 MHz).

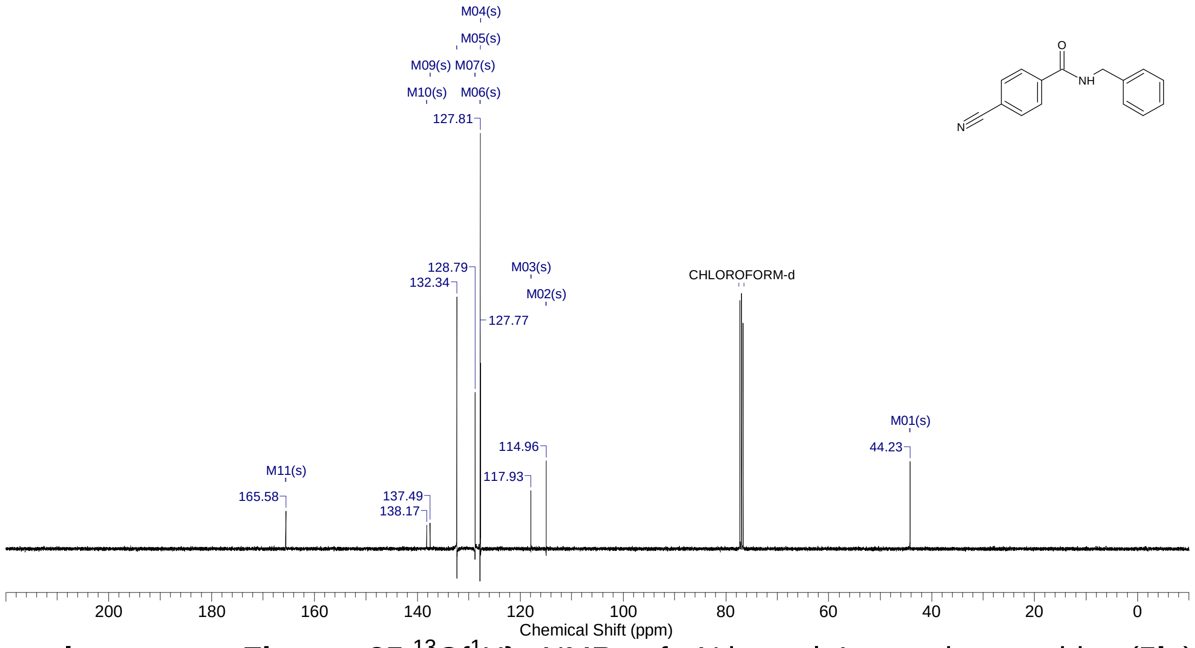

Supplementary Figure 25.$^{13}C\{^{1}H\}$ NMR of *N*-benzyl-4-cyanobenzamide (**5ia**) (Chloroform-d_3. 400 MHz).

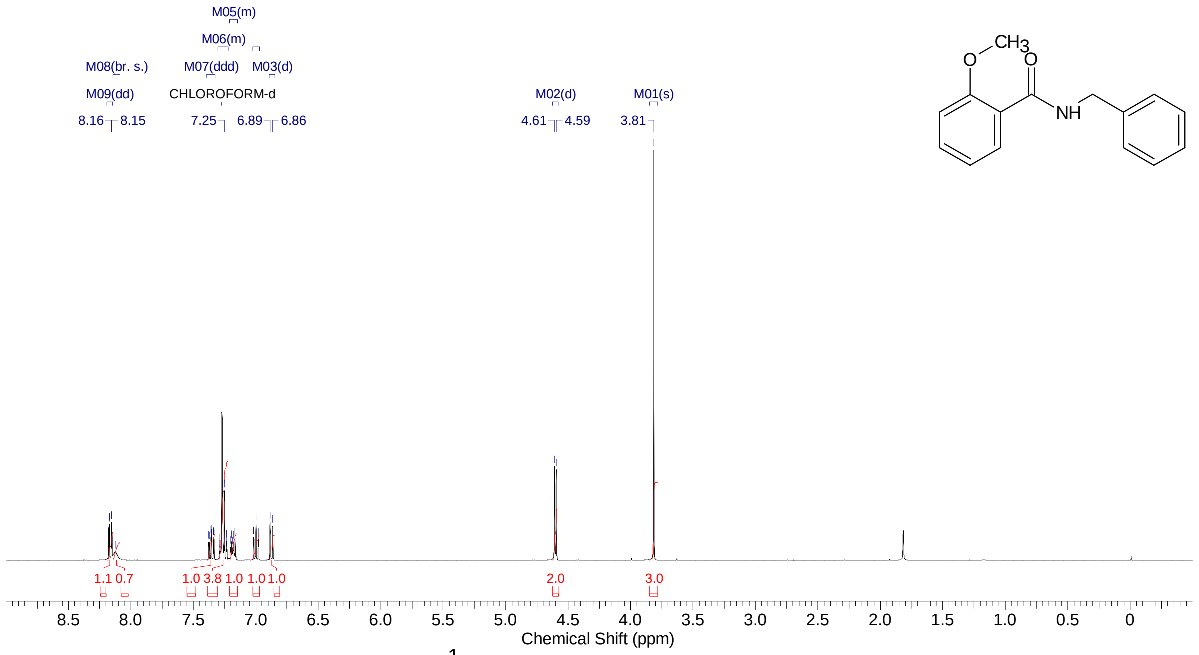

Supplementary Figure 26. ^{1}H NMR of *N*-benzyl-2-methoxybenzamide (**5ja**) (Chloroform-d_3, 400 MHz).

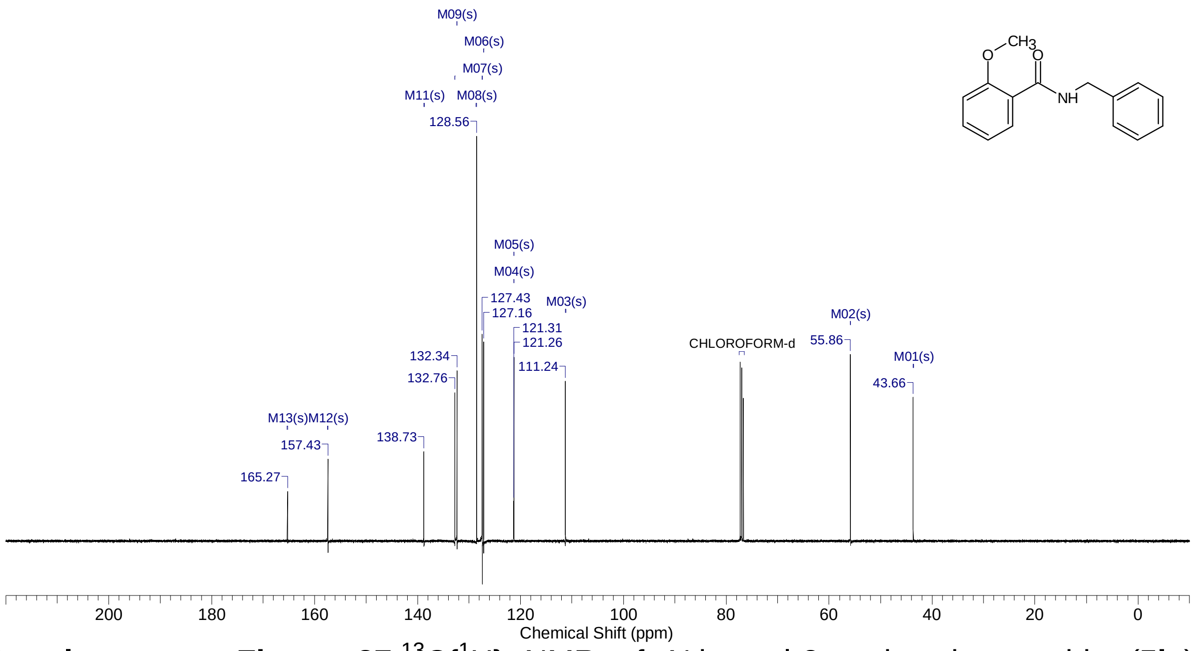

Supplementary Figure 27. $^{13}C\{^{1}H\}$ NMR of *N*-benzyl-2-methoxybenzamide (**5ja**) (Chloroform-d_3. 400 MHz).

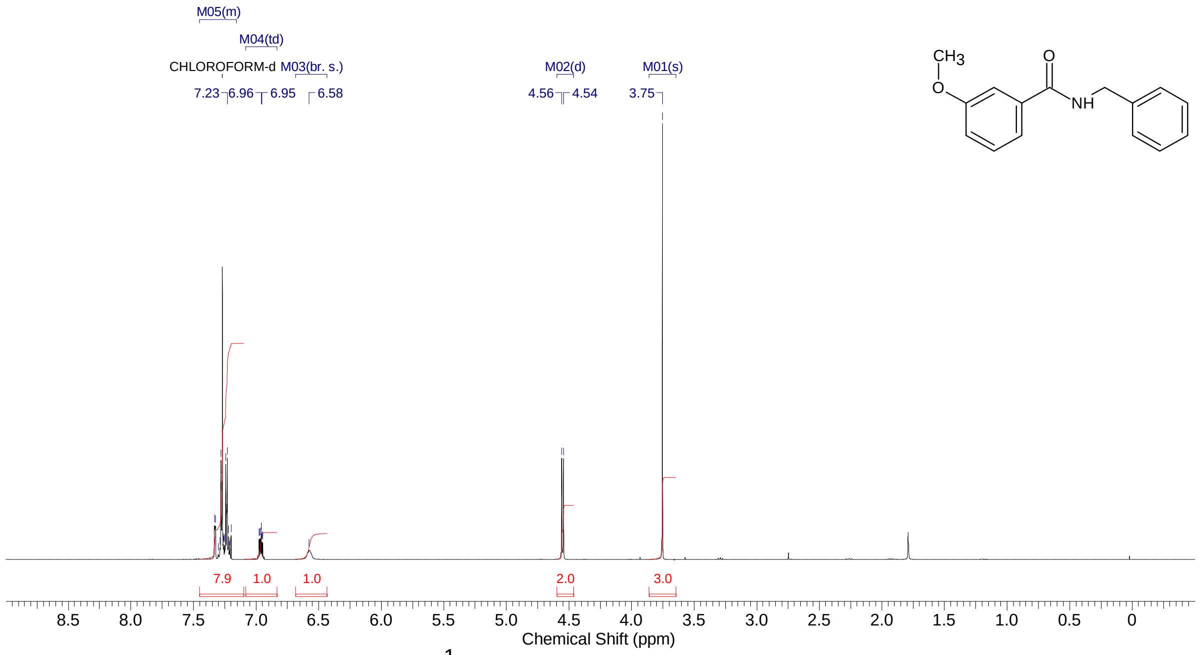

Supplementary Figure 28. ^{1}H NMR of *N*-benzyl-3-methoxybenzamide (**5ka**) (Chloroform-d_3, 400 MHz).

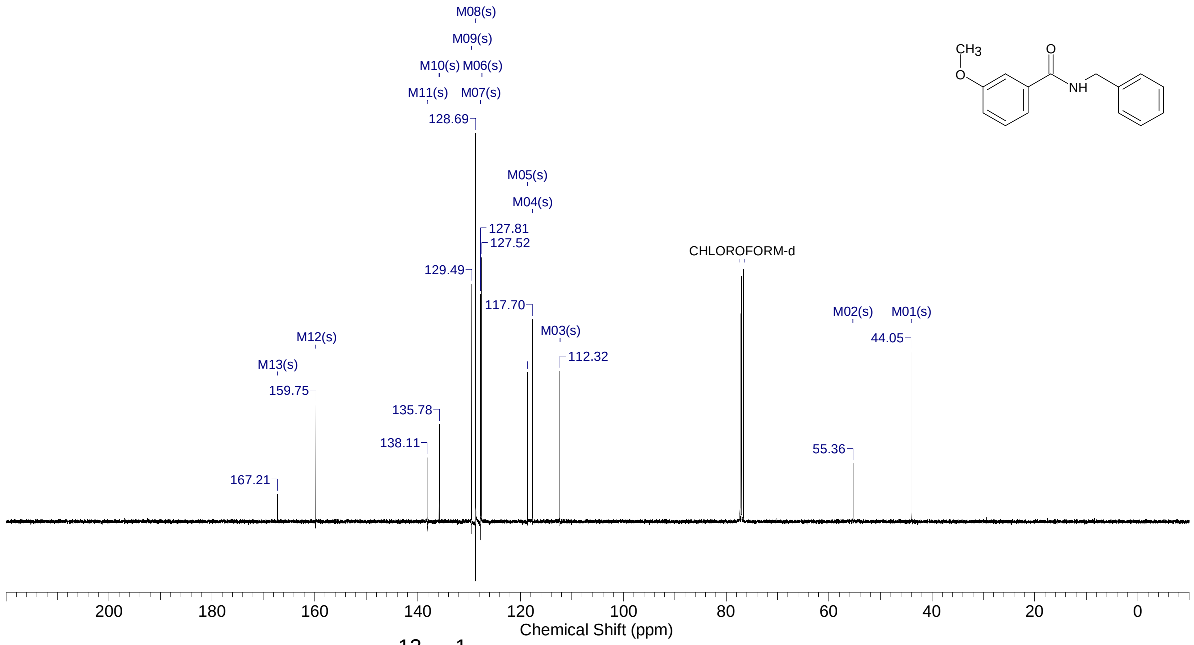

Supplementary Figure 29. $^{13}C\{^{1}H\}$ NMR of *N*-benzyl-3-methoxybenzamide (**5ka**) (Chloroform-d_3. 400 MHz).

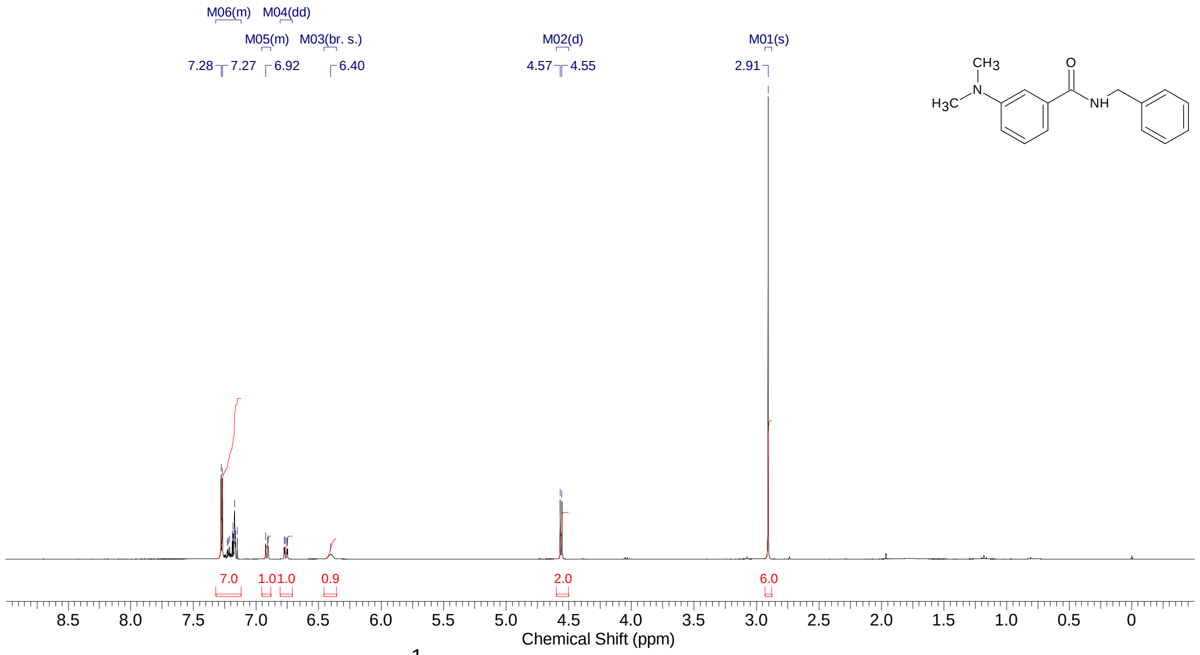

Supplementary Figure 30. ^{1}H NMR of 3-dimethylamino-*N*-benzylbenzamide (**5la**) (Chloroform-d_3, 400 MHz).

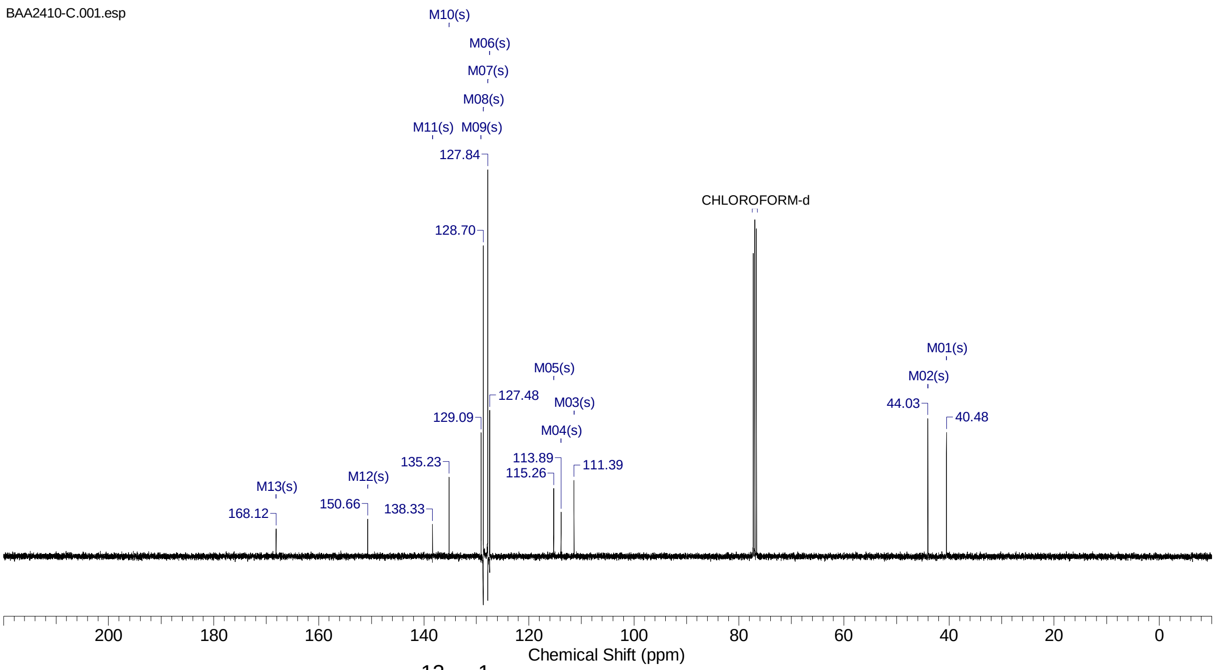

Supplementary Figure 31. $^{13}C\{^{1}H\}$ NMR of 3-dimethylamino-*N*-benzylbenzamide (**5la**) (Chloroform-d_3. 400 MHz).

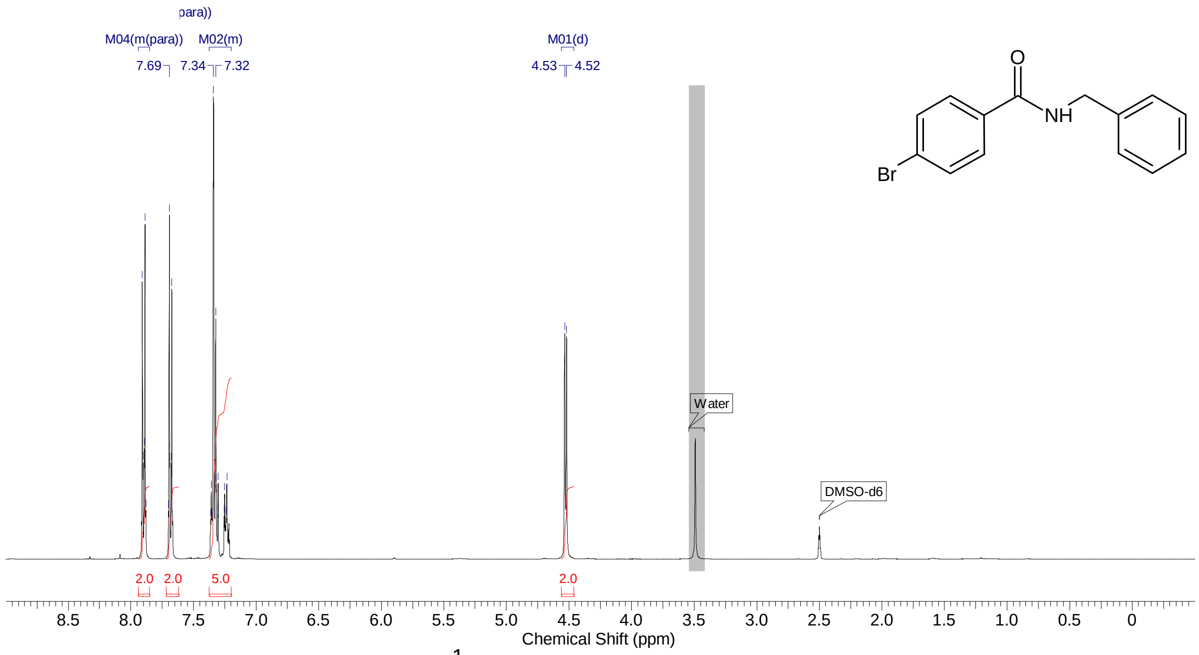

Supplementary Figure 32. ^{1}H NMR of *N*-benzyl-4-bromobenzamide (**5ma**) (Chloroform-d_3, 400 MHz).

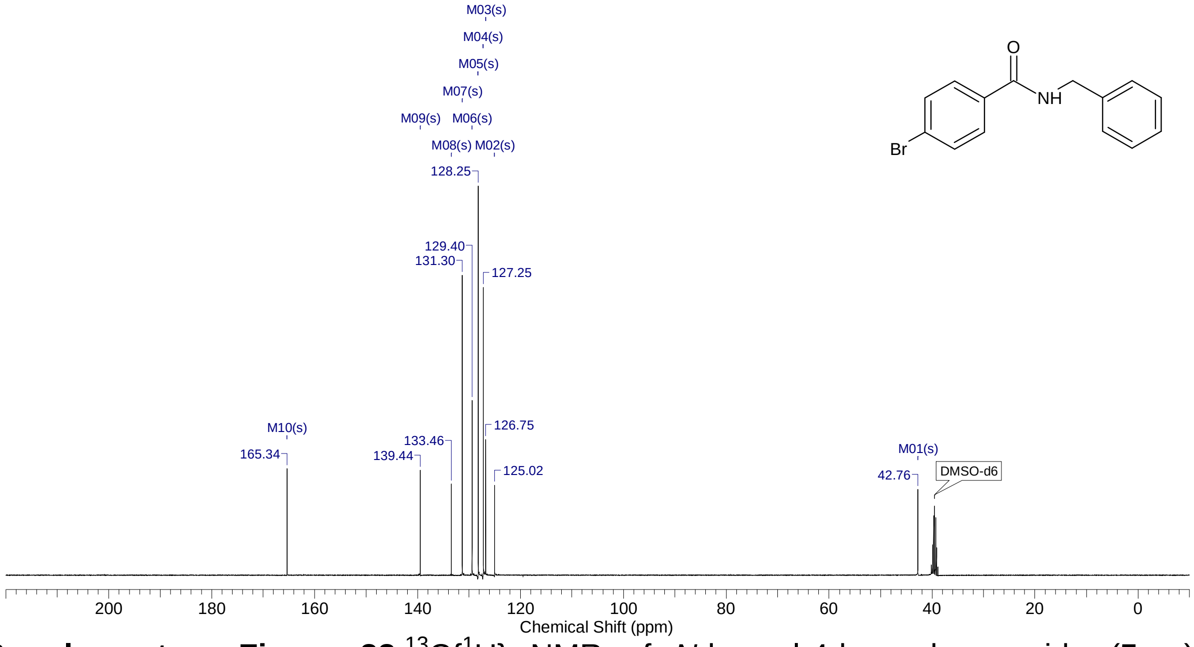

Supplementary Figure 33.$^{13}C\{^{1}H\}$ NMR of *N*-benzyl-4-bromobenzamide (**5ma**) (Chloroform-d_3. 400 MHz).

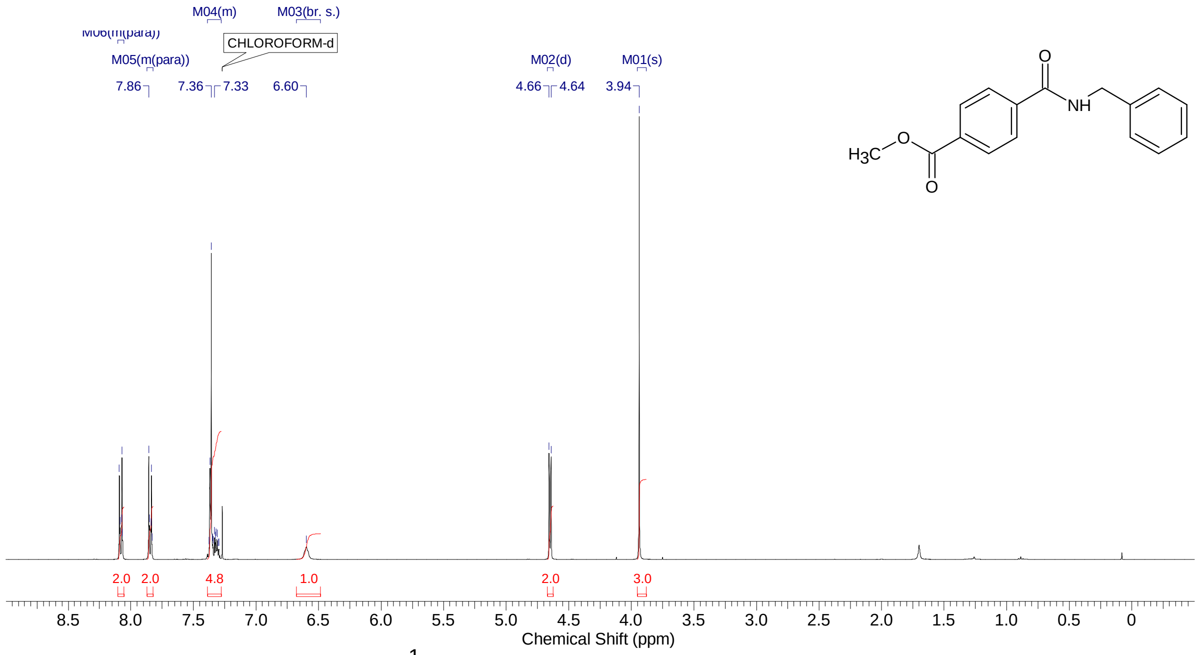

Supplementary Figure 34. ^{1}H NMR of *N*-benzyl-terephthalamic acid methyl ester (**5na**) (Chloroform-d_3, 400 MHz).

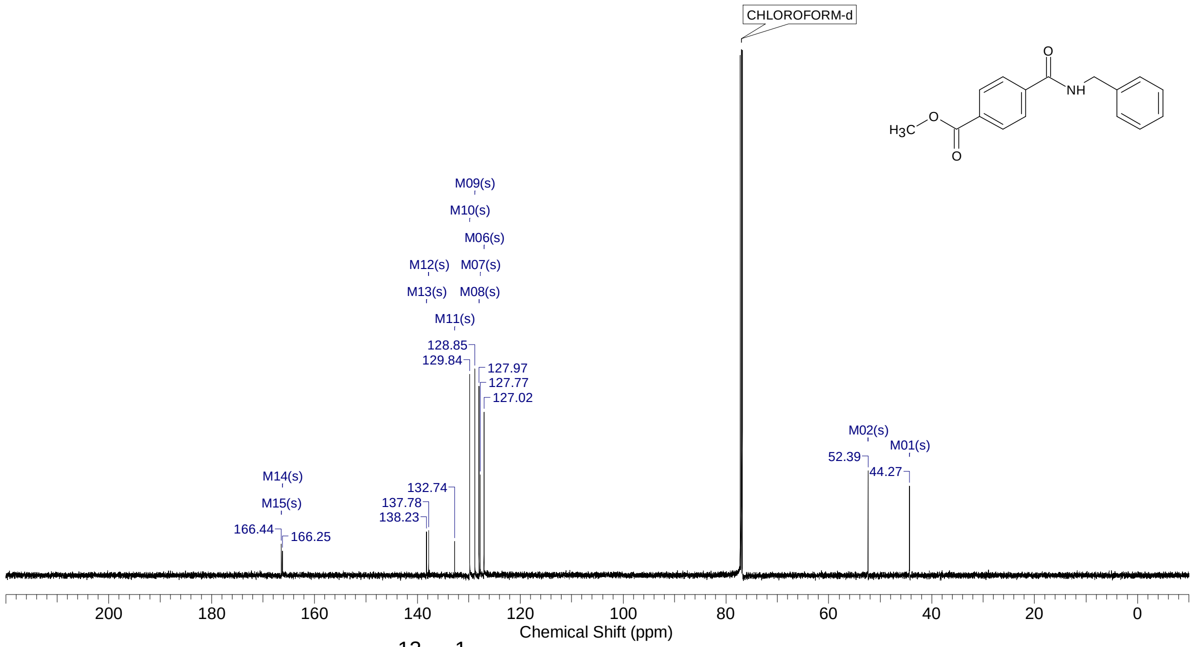

Supplementary Figure 35. $^{13}C\{^{1}H\}$ NMR of *N*-benzyl-terephthalamic acid methyl ester (**5na**) (Chloroform-d_3. 400 MHz).

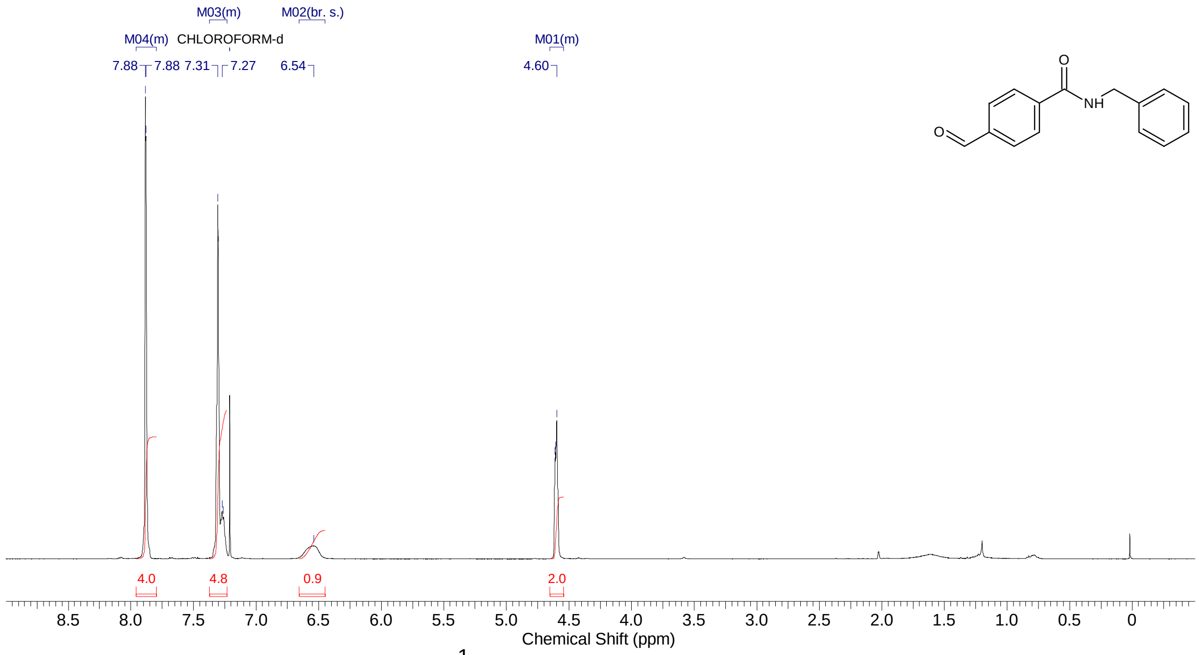

Supplementary Figure 36. ^{1}H NMR of *N*-benzyl-4-formylbenzamide (**5oa**) (Chloroform-d_3, 400 MHz).

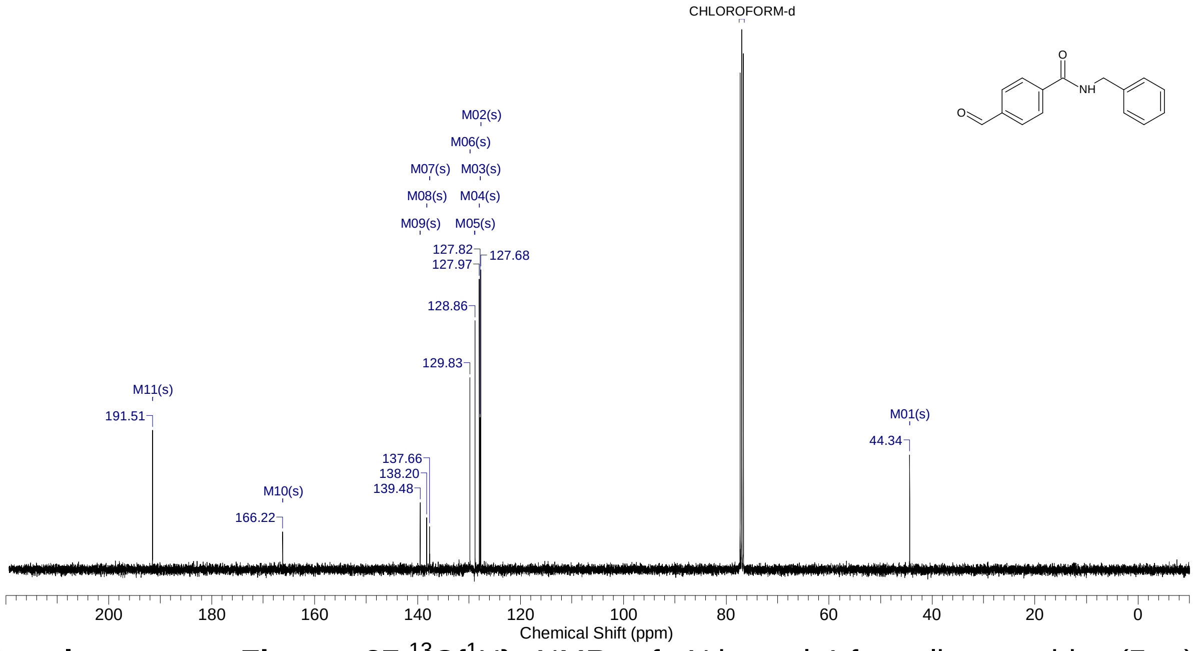

Supplementary Figure 37.^{13}C{^{1}H} NMR of *N*-benzyl-4-formylbenzamide (**5oa**) (Chloroform-d_3. 400 MHz).

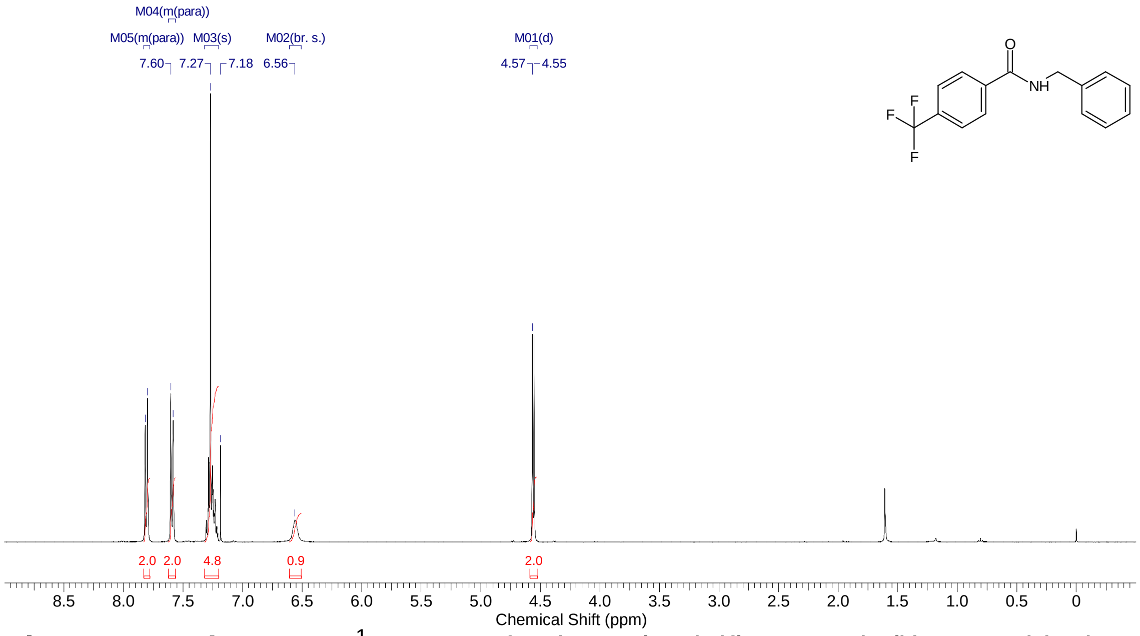

Supplementary Figure 38. ^{1}H NMR of *N*-benzyl-4-(trifluoromethyl)benzamide (**5pa**) (Chloroform-d_3, 400 MHz).

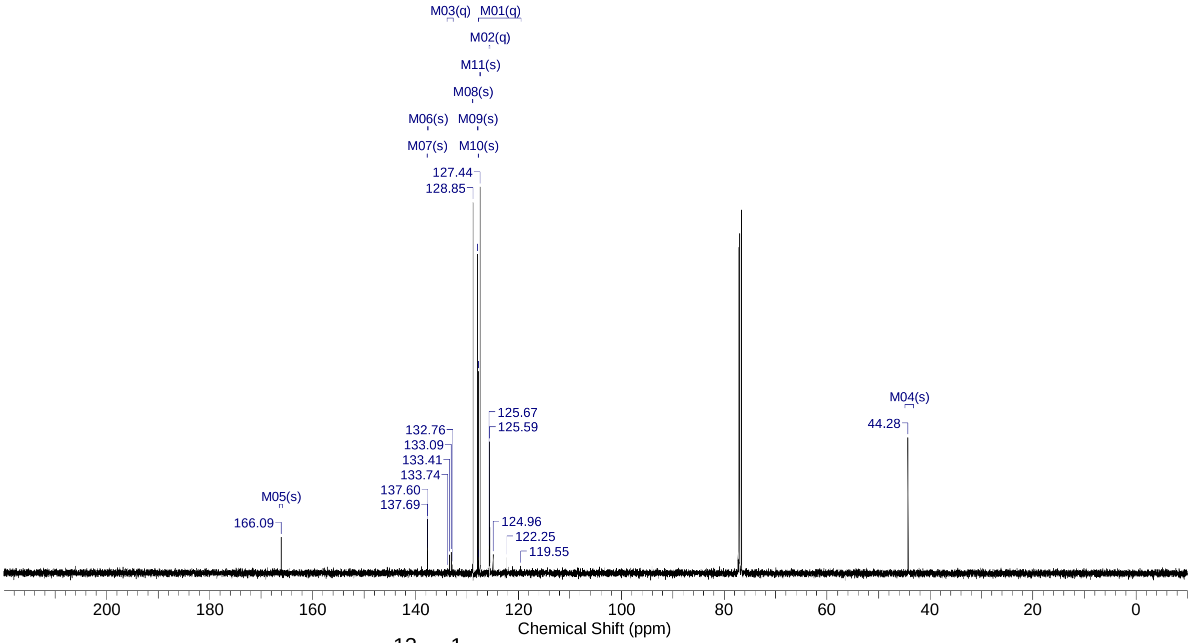

Supplementary Figure 39.^{13}C{^{1}H} NMR of *N*-benzyl-4-(trifluoromethyl)benzamide (**5pa**) (Chloroform-d_3. 400 MHz).

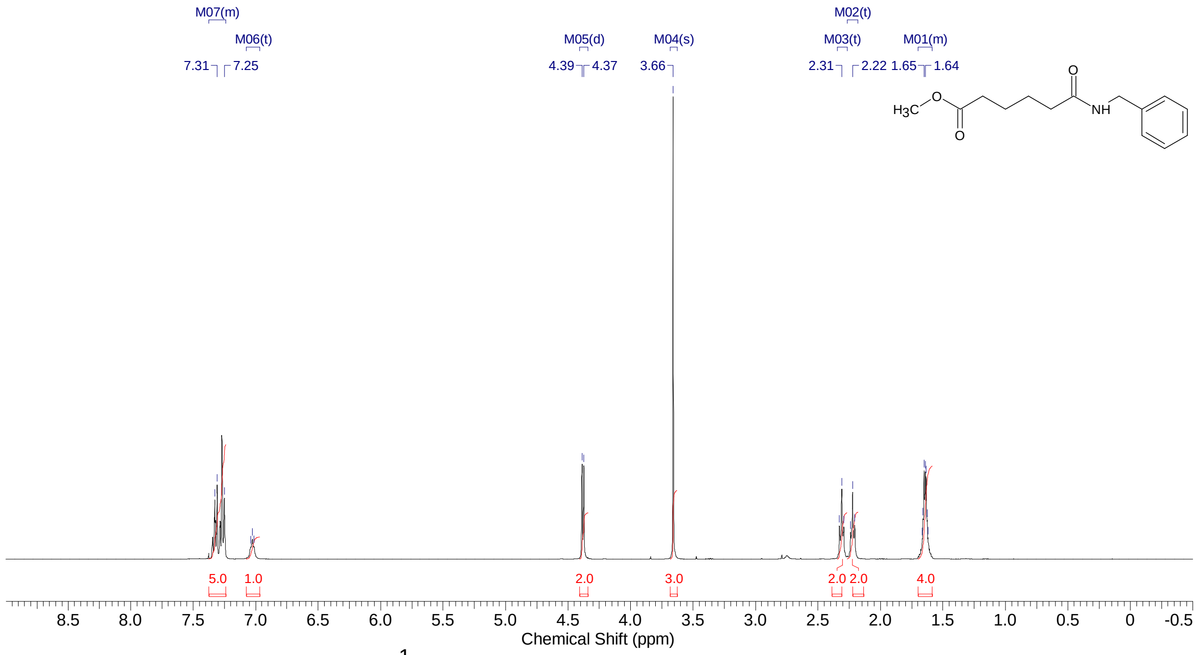

Supplementary Figure 40. ^{1}H NMR of 6-oxo-6-(benzylamino)-hexanoic acid methyl ester (**5qa**) (Chloroform-d_3, 400 MHz).

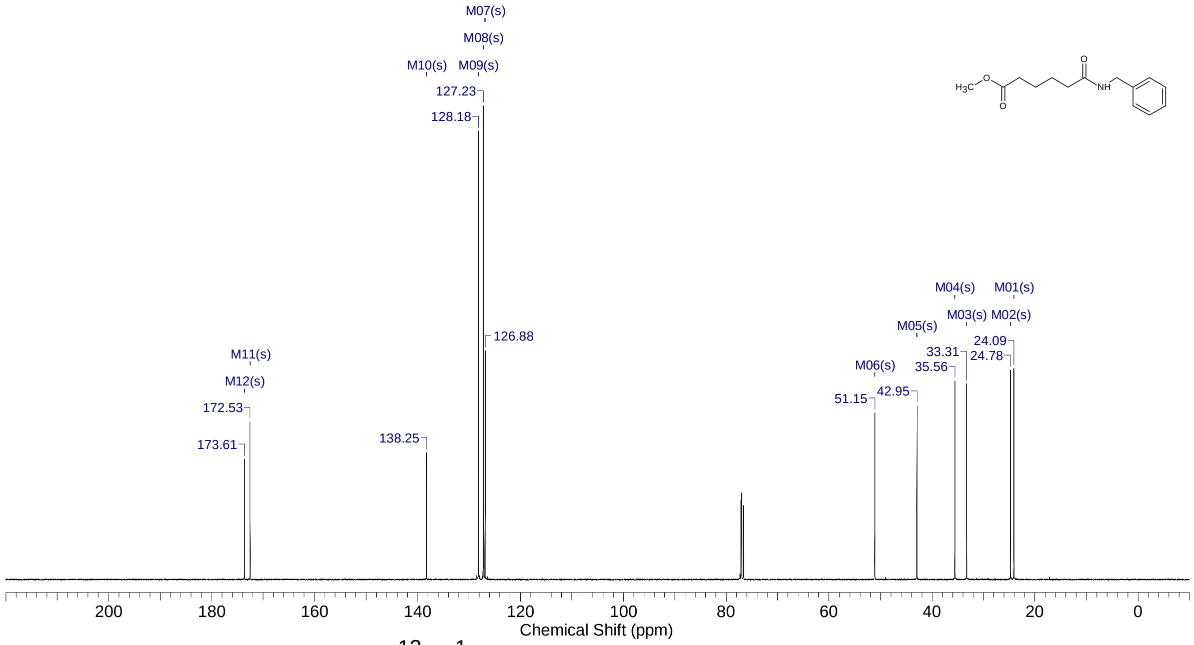

Supplementary Figure 41. $^{13}C\{^{1}H\}$ NMR of 6-oxo-6-(benzylamino)-hexanoic acid methyl ester (**5qa**) (Chloroform-d_3. 400 MHz).

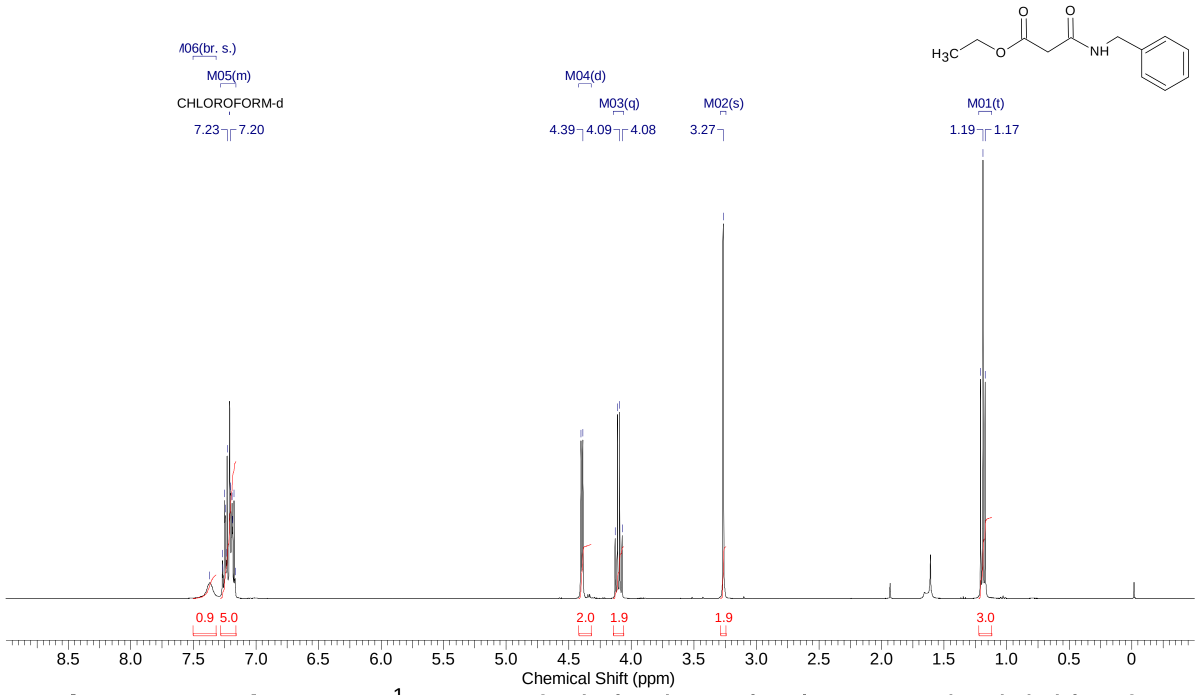

Supplementary Figure 42. ^{1}H NMR of ethyl *N*-benzylmalonamate (**5ra**) (Chloroform-*d*$_3$, 400 MHz).

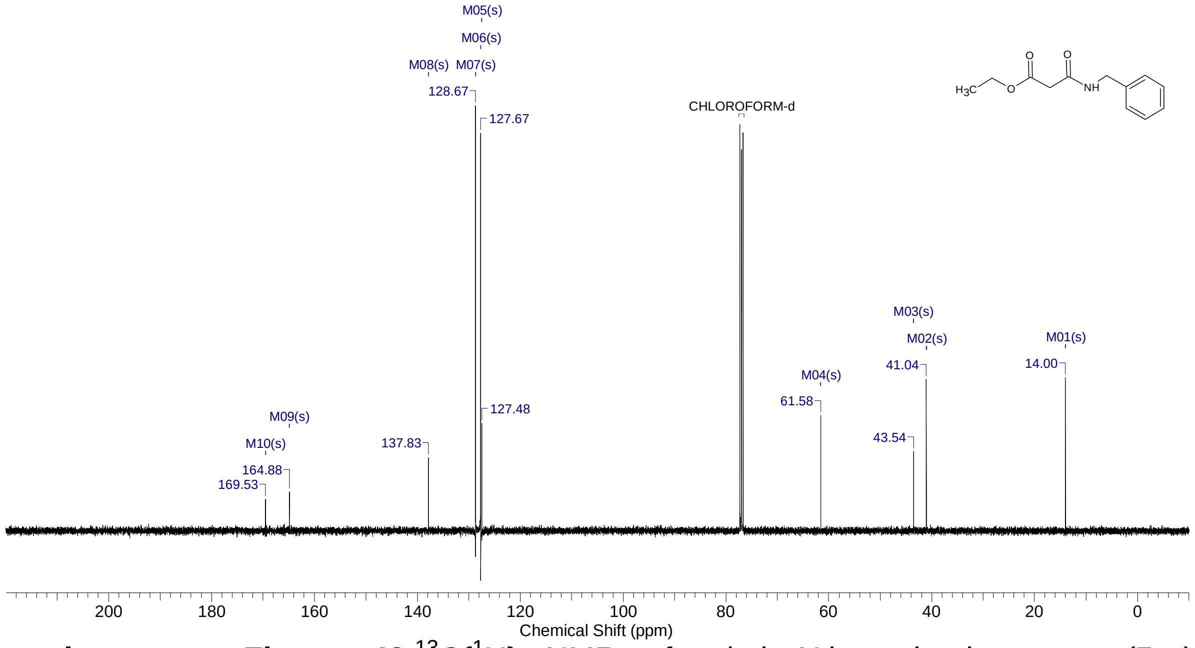

Supplementary Figure 43. ^{13}C{^{1}H} NMR of ethyl *N*-benzylmalonamate (**5ra**) (Chloroform-*d*$_3$. 400 MHz).

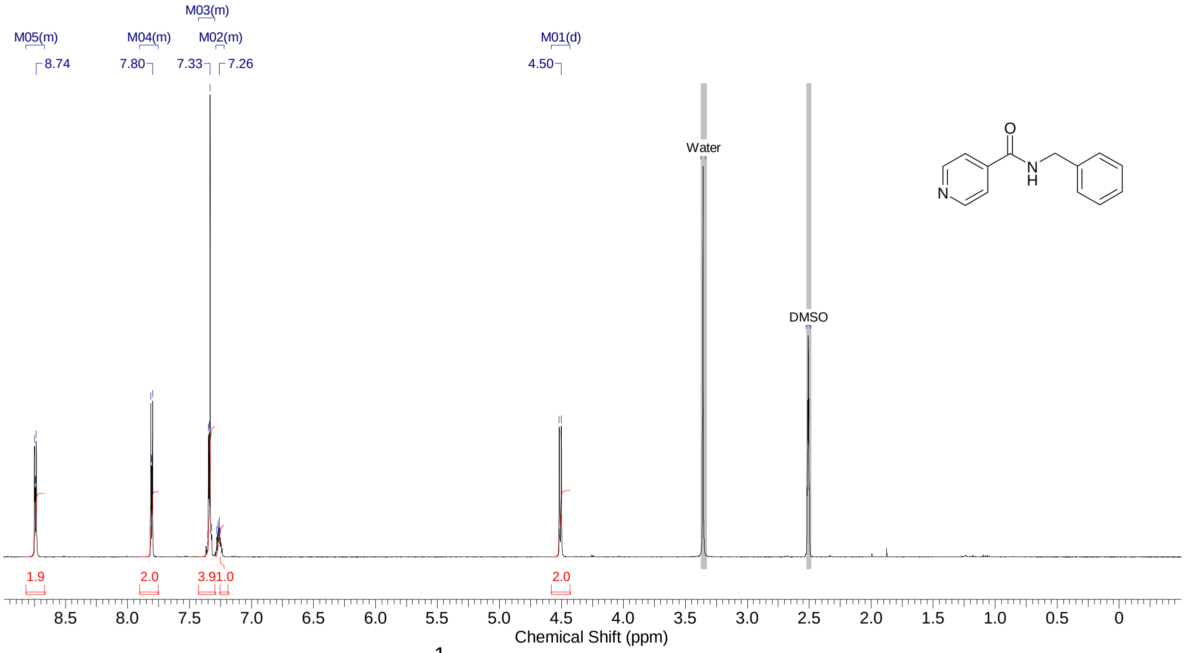

Supplementary Figure 44. ^{1}H NMR of *N*-benzylpyridine-4-carboxamide (**5sa**) (DMSO-d_6, 400 MHz).

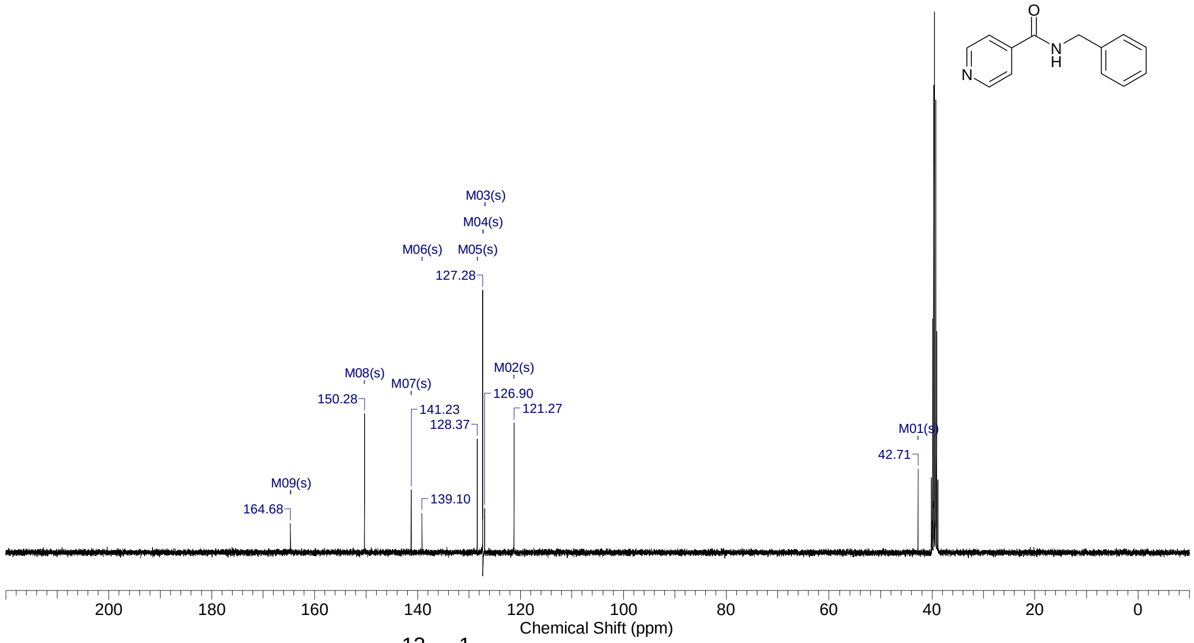

Supplementary Figure 45. ^{13}C{^{1}H} NMR of *N*-benzylpyridine-4-carboxamide (**5sa**) (DMSO-d_6. 400 MHz).

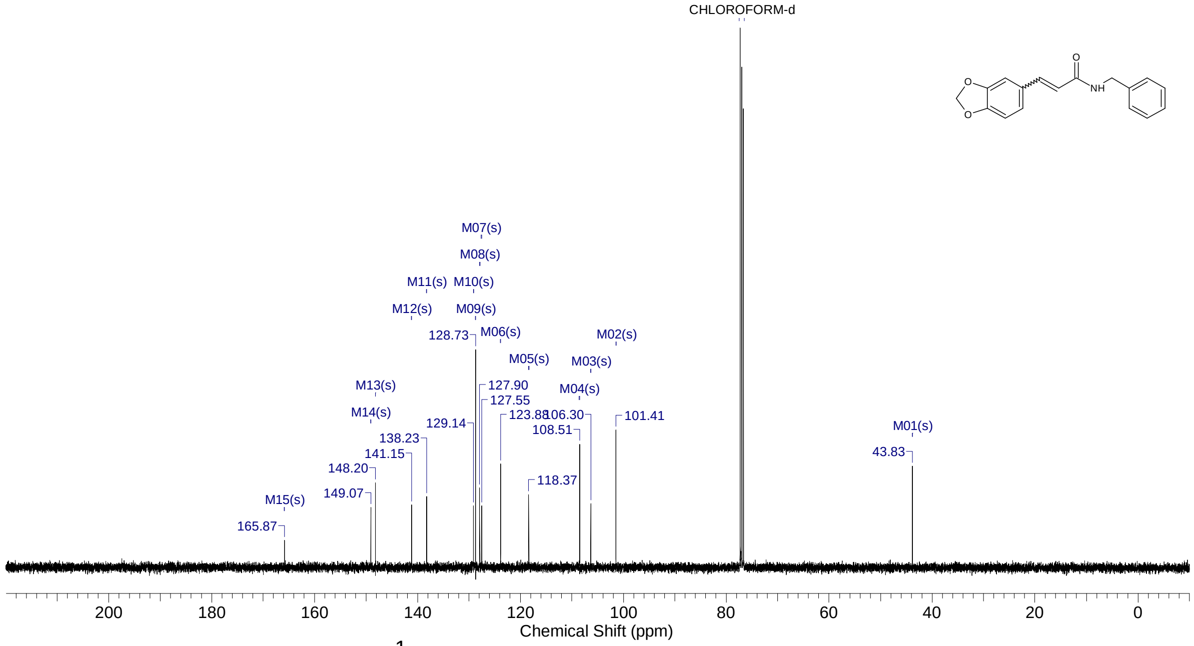

Supplementary Figure 46. ^{1}H NMR of 3-(1,3-benzodioxol-5-yl)-*N*-(phenylmethyl)-2-propenamide (**5ta**) (Chloroform-d_3, 400 MHz).

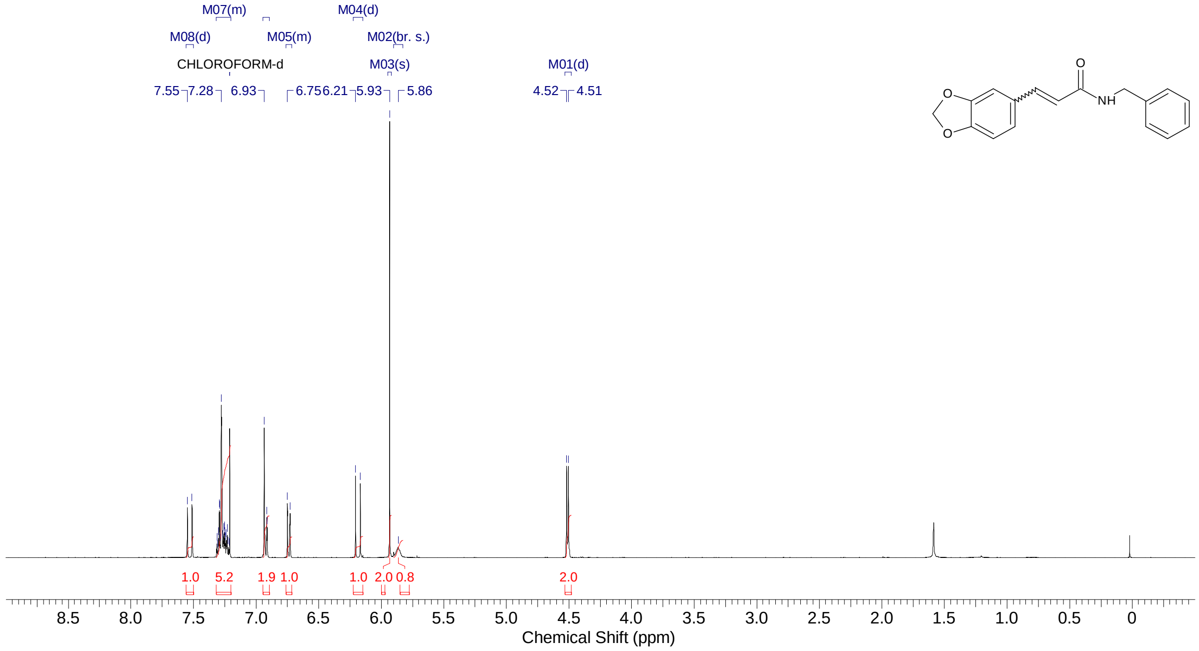

Supplementary Figure 47.^{13}C{^{1}H} NMR of 3-(1,3-benzodioxol-5-yl)-*N*-(phenylmethyl)-2-propenamide (**5ta**) (Chloroform-d_3. 400 MHz).

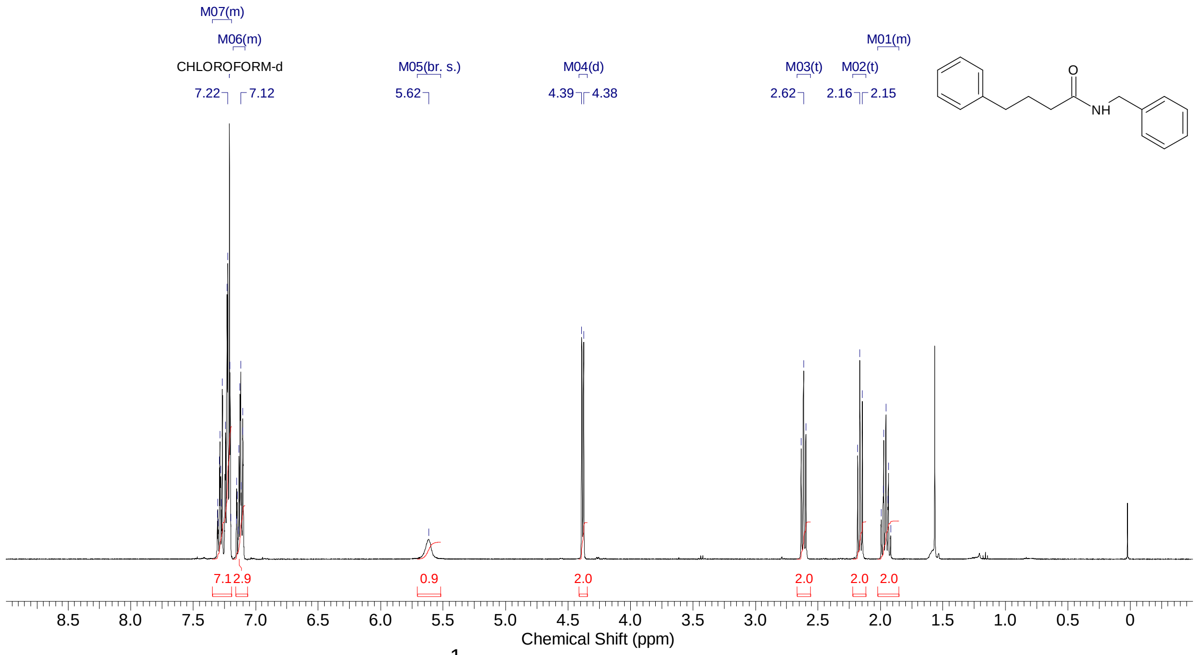

Supplementary Figure 48. ^{1}H NMR of *N*-benzyl-4-phenylbutanamide (**5ua**) (Chloroform-d_3, 400 MHz).

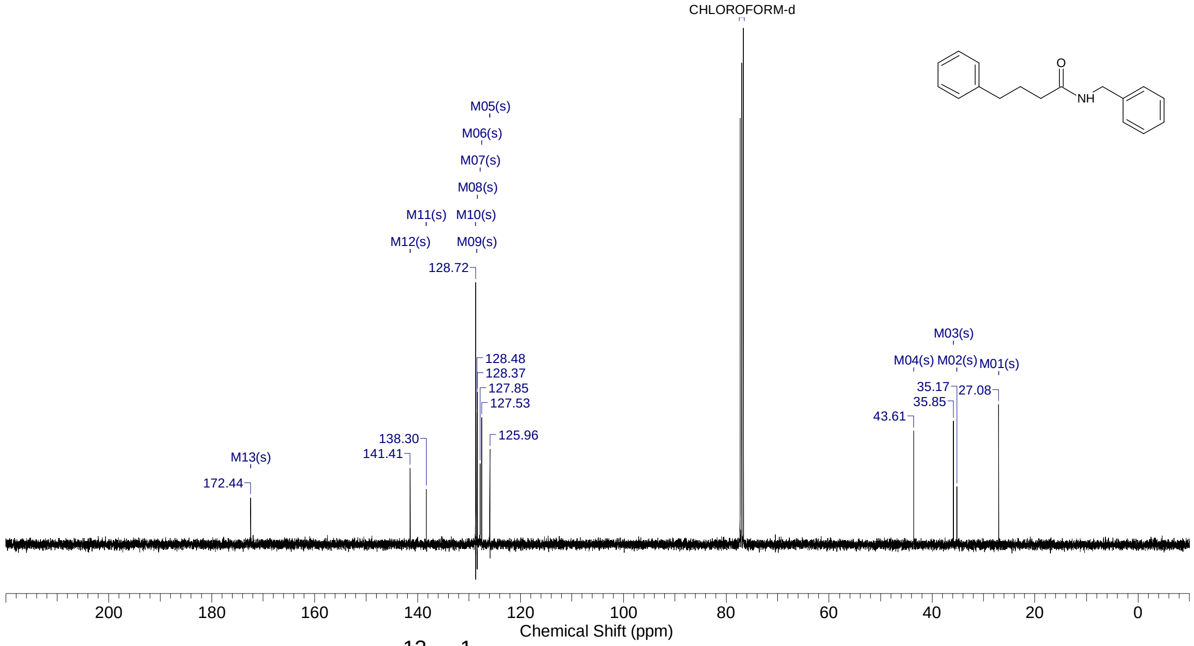

Supplementary Figure 49.$^{13}C\{^{1}H\}$ NMR of *N*-benzyl-4-phenylbutanamide (**5ua**) (Chloroform-d_3. 400 MHz).

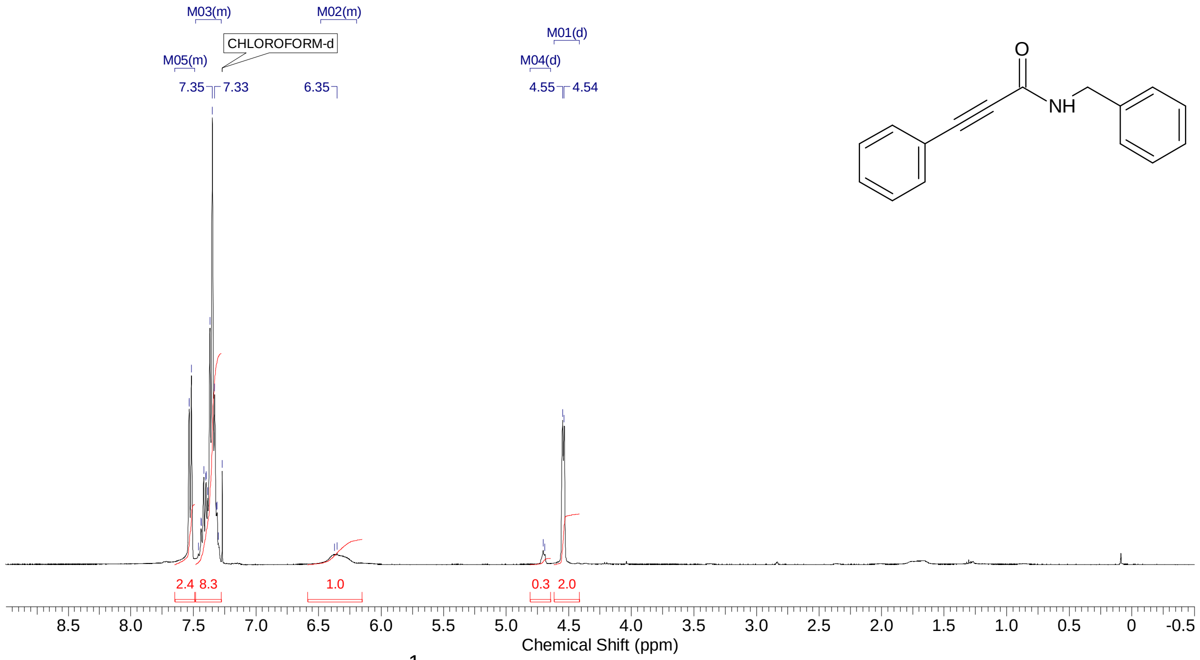

Supplementary Figure 50. ^{1}H NMR of ethyl *N*-benzyl-3-phenylpropiolamide (**5va**) (Chloroform-d_3, 400 MHz).

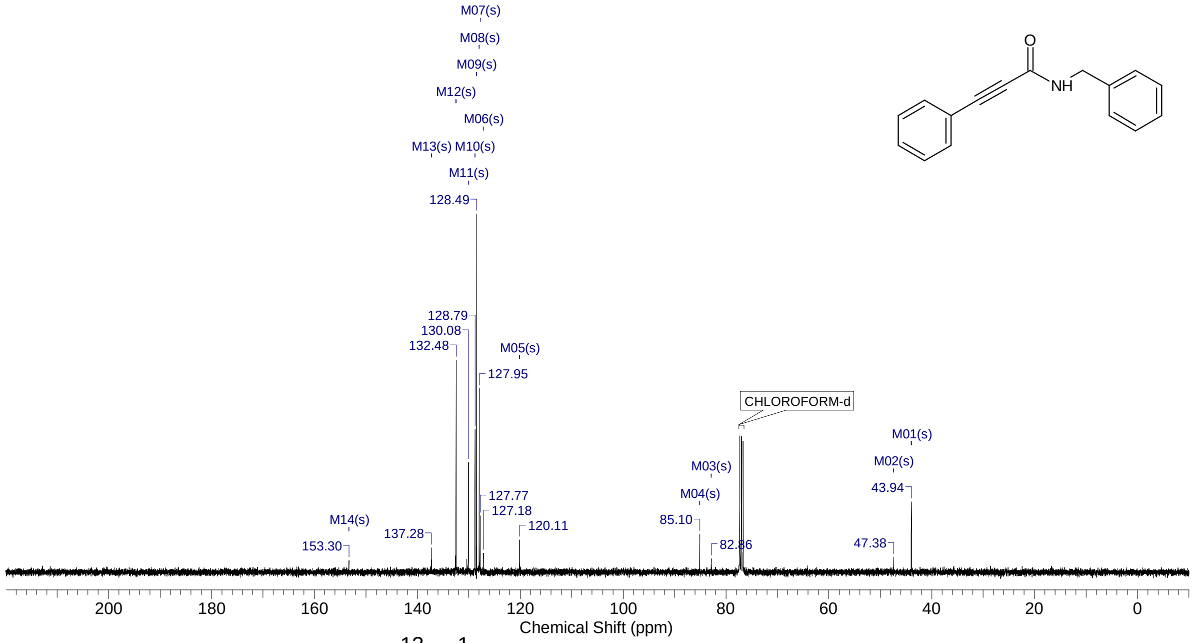

Supplementary Figure 51.$^{13}C\{^{1}H\}$ NMR of ethyl *N*-benzyl-3-phenylpropiolamide (**5va**) (Chloroform-d_3. 400 MHz).

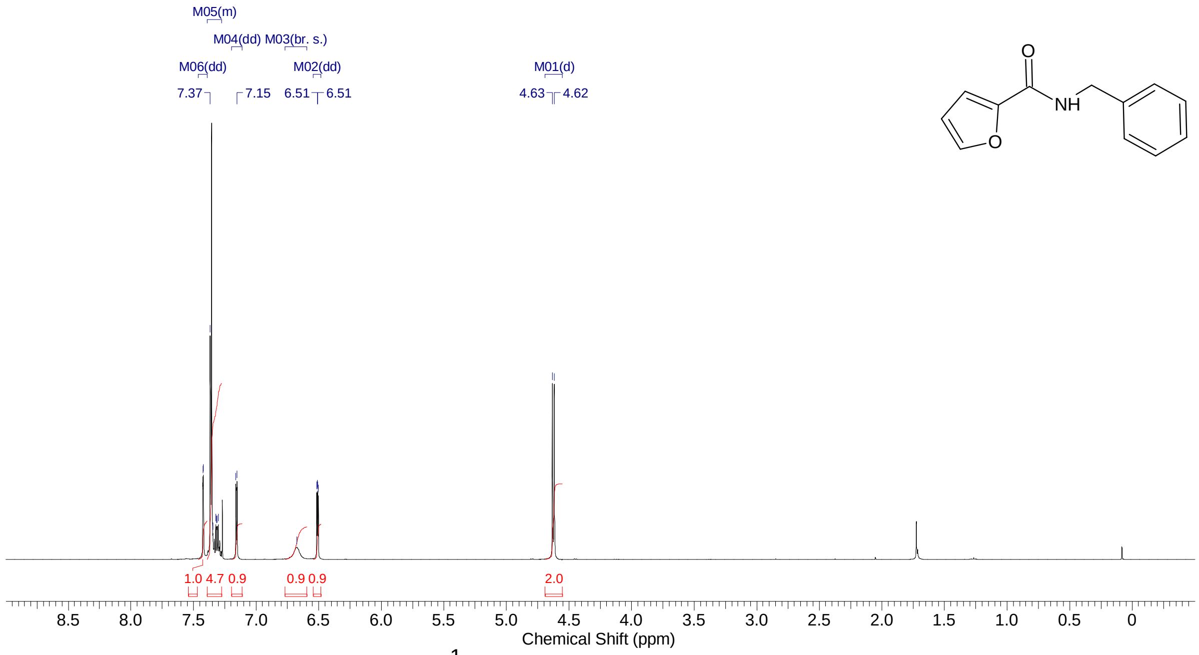

Supplementary Figure 52. ^{1}H NMR of *N*-benzylfuran-2-carboxamide (**5wa**) (Chloroform-d_3, 400 MHz).

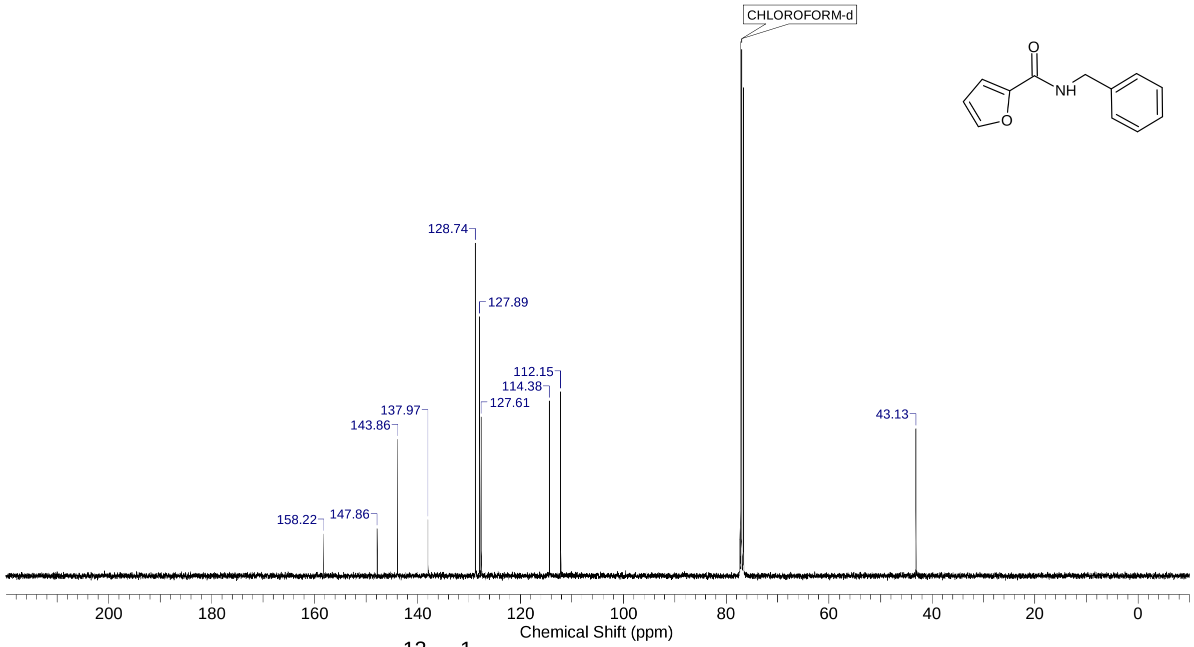

Supplementary Figure 53. $^{13}C\{^{1}H\}$ NMR of *N*-benzylfuran-2-carboxamide (**5wa**) (Chloroform-d_3. 400 MHz).

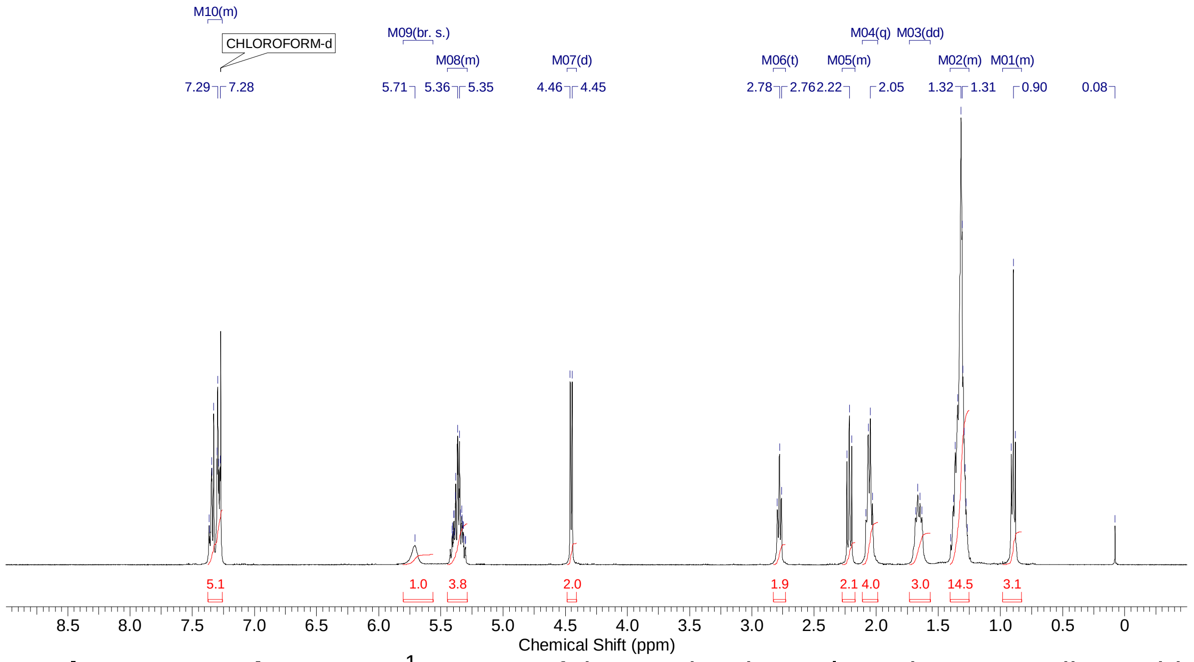

Supplementary Figure 54. ^{1}H NMR of (9Z,12Z)-*N*-benzyloctadeca-9,12-dienamide (**5xa**) (Chloroform-d_3, 400 MHz).

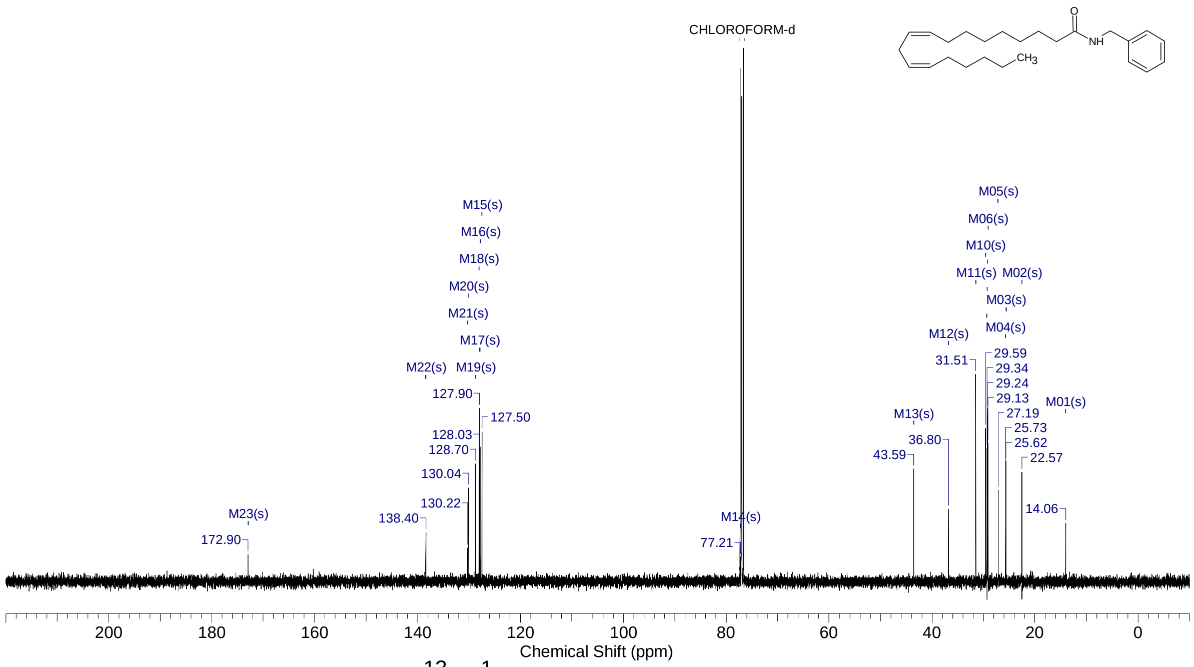

Supplementary Figure 55. $^{13}C\{^{1}H\}$ NMR of (9Z,12Z)-*N*-benzyloctadeca-9,12-dienamide (**5xa**) (Chloroform-d_3. 400 MHz).

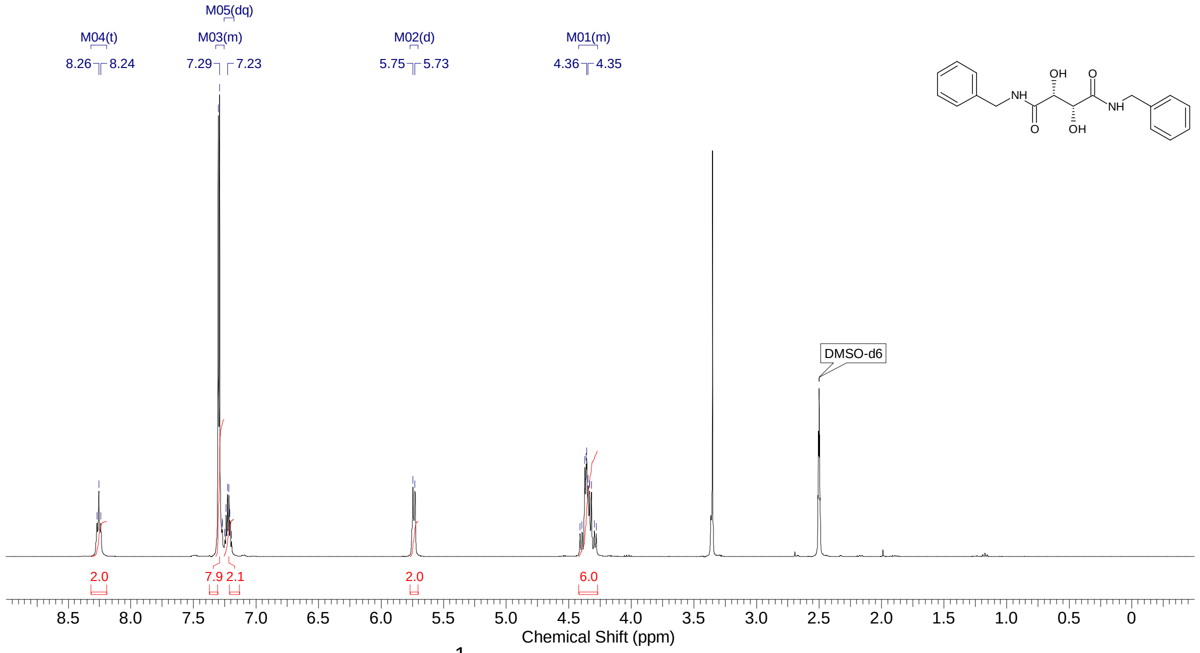

Supplementary Figure 56. ^{1}H NMR of (S,S)-*N*,*N'*-dibenzyltartramide (**5ya**) (Chloroform-d_3, 400 MHz).

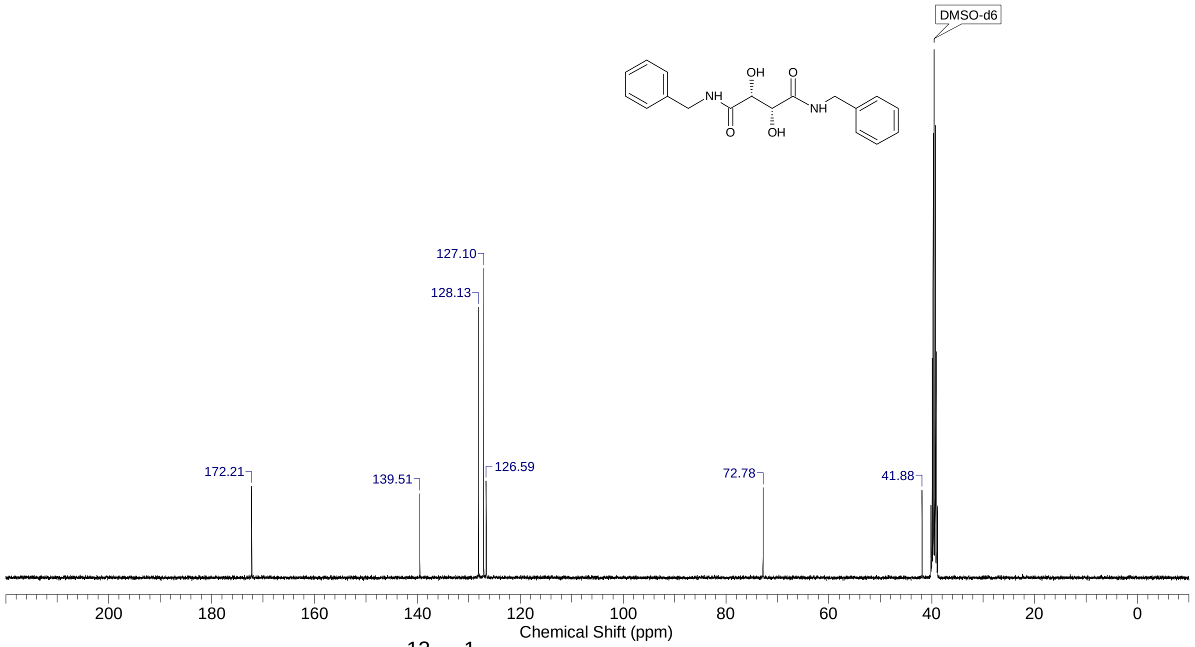

Supplementary Figure 57. ^{13}C{^{1}H} NMR of (S,S)-*N*,*N'*-dibenzyltartramide (**5ya**) (DMSO-d_6. 400 MHz).

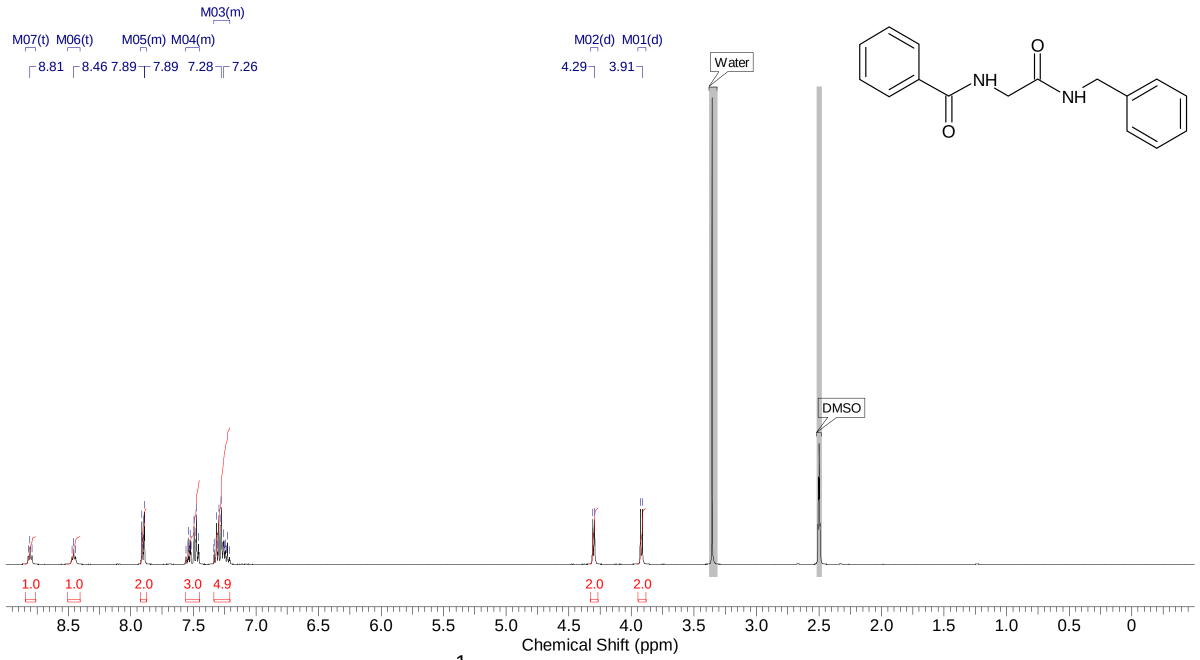

Supplementary Figure 58. ^{1}H NMR of ethyl *N*-(2-benzylamino-2-oxoethyl) benzamide (**5za**) (DMSO-d_6, 400 MHz).

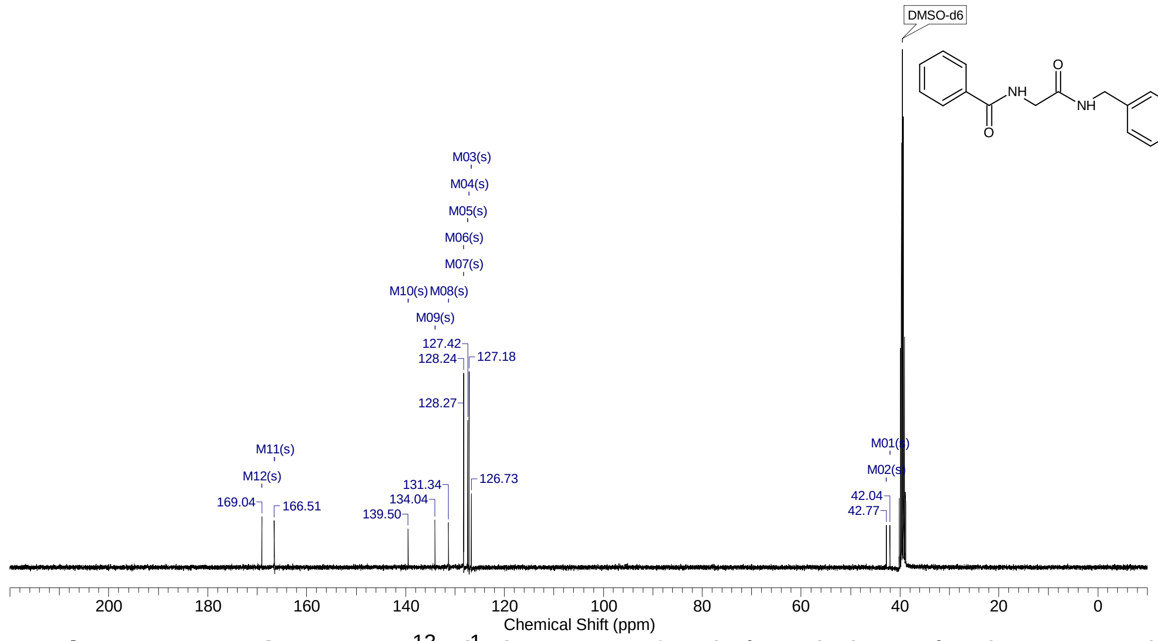

Supplementary Figure 59. ^{13}C{^{1}H} NMR of ethyl *N*-(2-benzylamino-2-oxoethyl) benzamide (**5za**) (DMSO-d_6. 400 MHz).

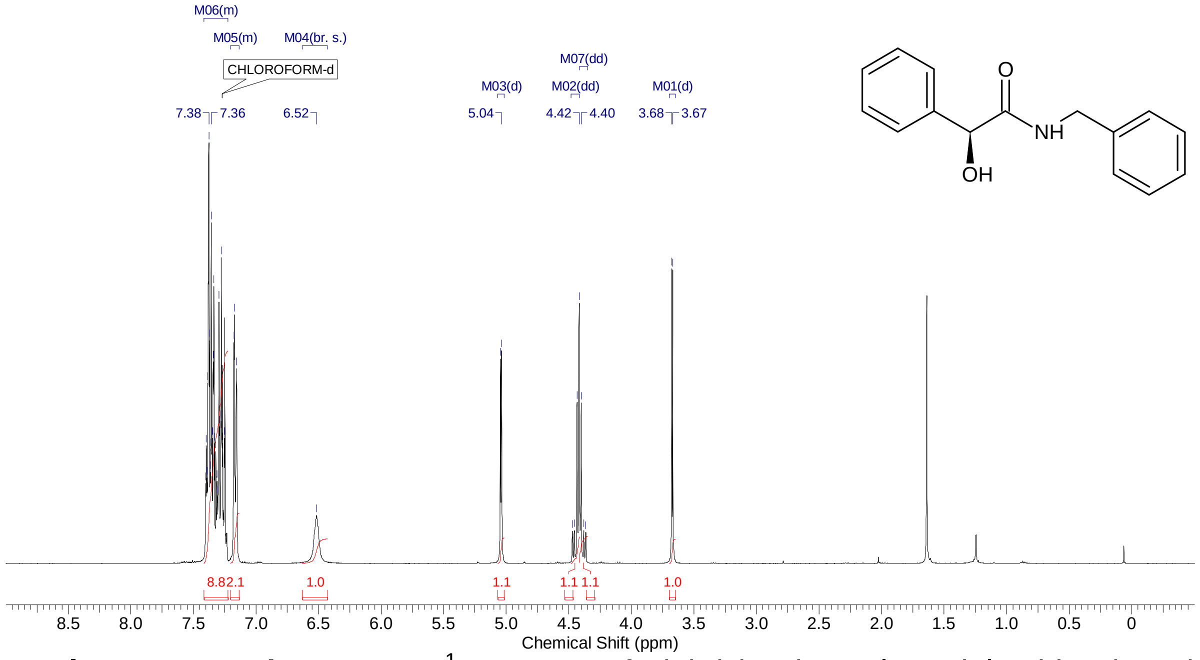

Supplementary Figure 60. ^{1}H NMR of (S)-(+)-*N*-benzylmandelamide (**5aaa**) (Chloroform-d_3, 400 MHz).

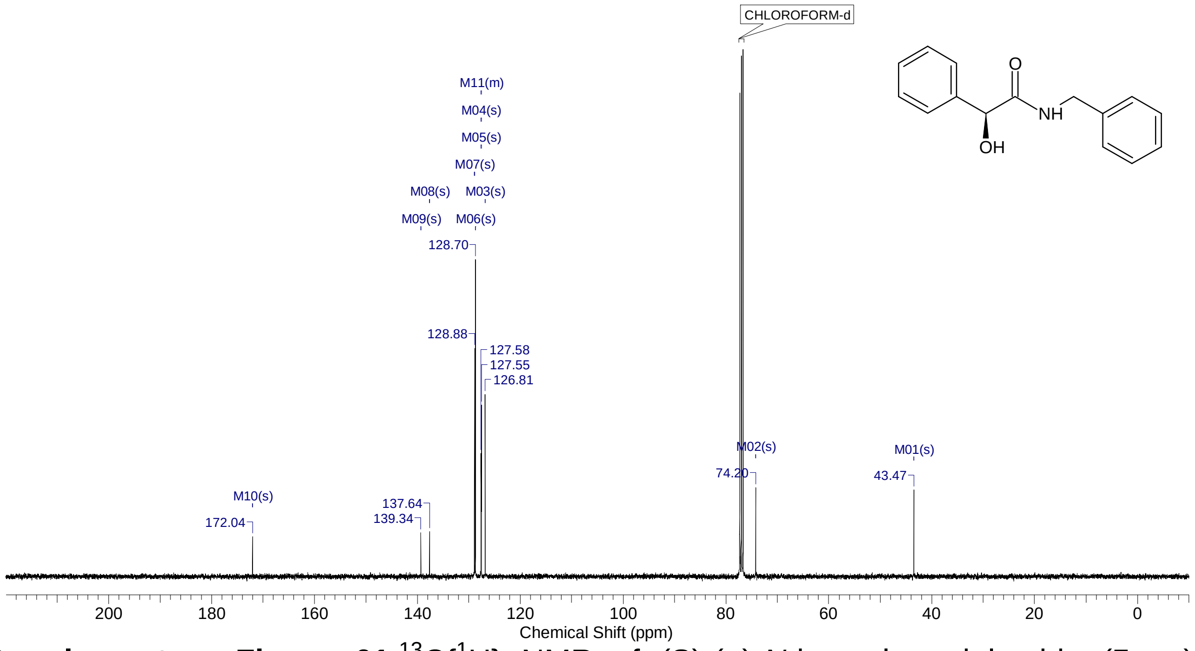

Supplementary Figure 61. $^{13}C\{^{1}H\}$ NMR of (S)-(+)-*N*-benzylmandelamide (**5aaa**) (Chloroform-d_3. 400 MHz).

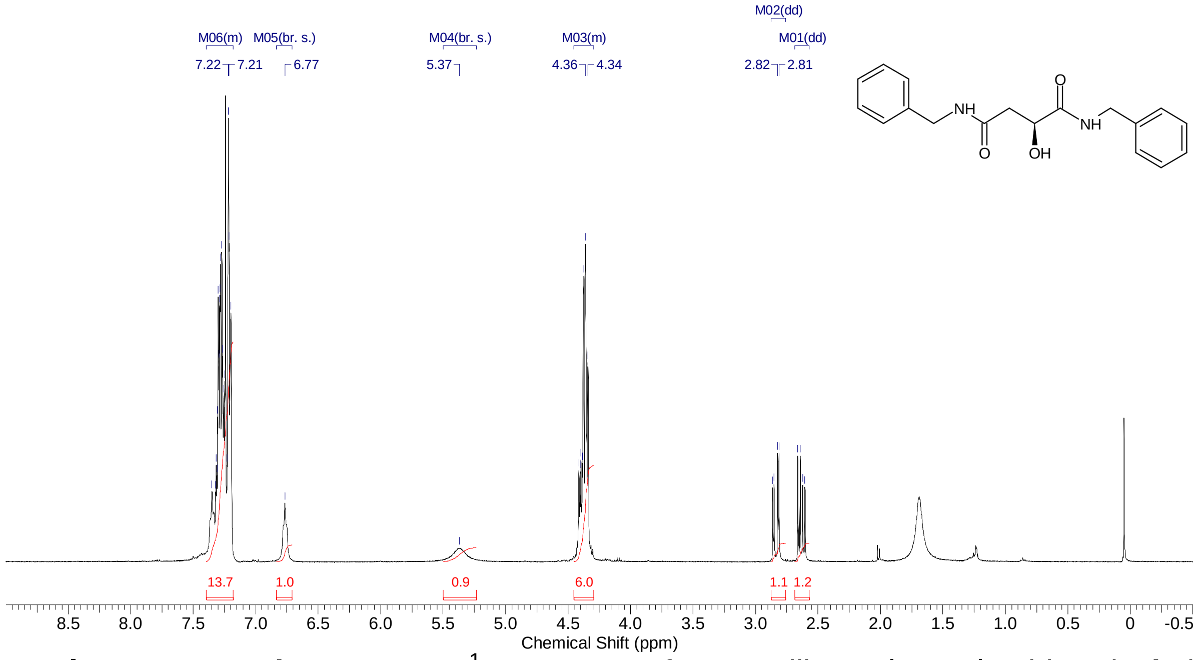

Supplementary Figure 62. ^{1}H NMR of *N*,*N*'-dibenzyl-L-malamide (**5aba**) (Chloroform-d_3, 400 MHz).

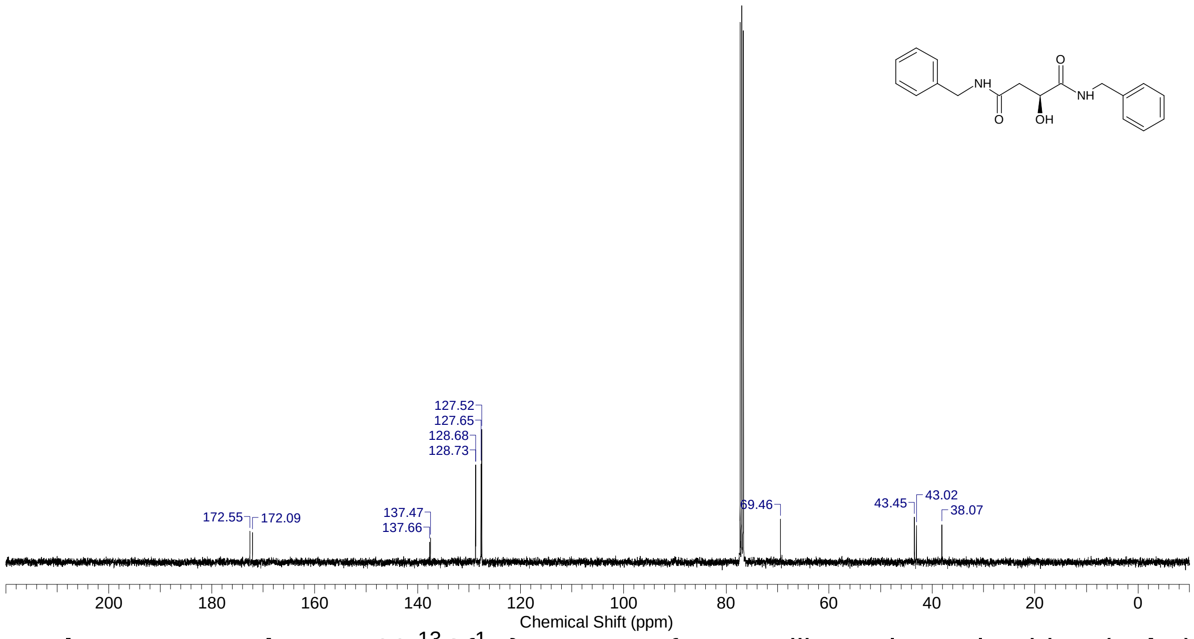

Supplementary Figure 63.^{13}C{^{1}H} NMR of *N*,*N*'-dibenzyl-L-malamide (**5aba**) (Chloroform-d_3. 400 MHz).

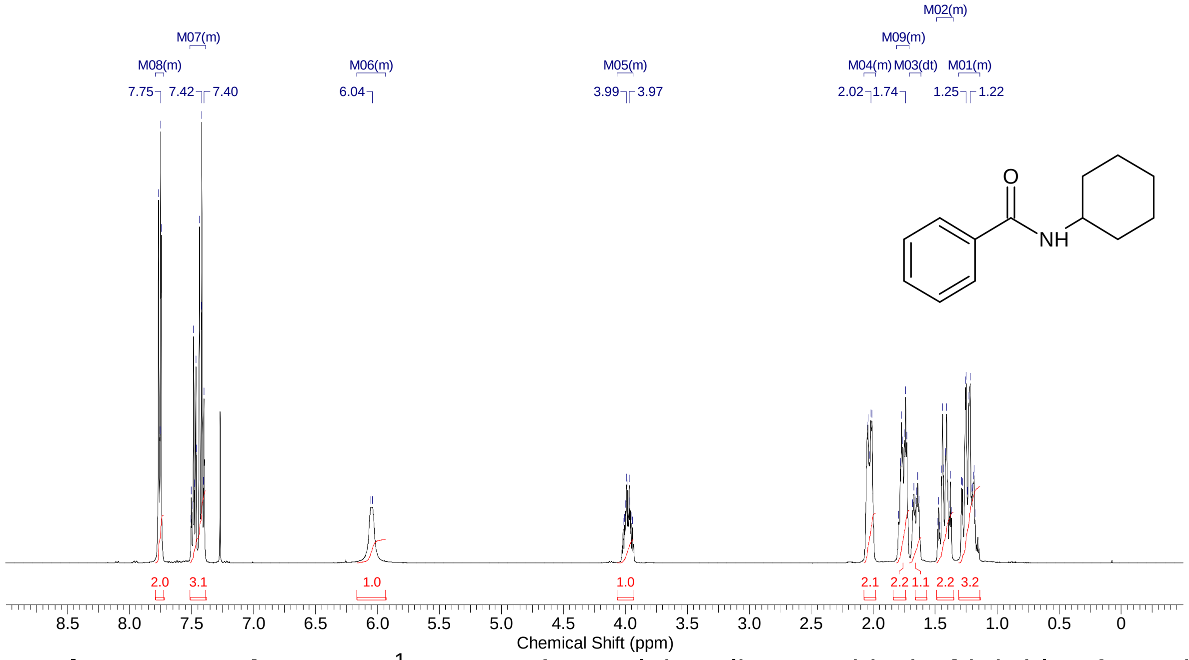

Supplementary Figure 64. ^{1}H NMR of *N*-cyclohexylbenzamide (**5ab**) (Chloroform-d_3, 400 MHz).

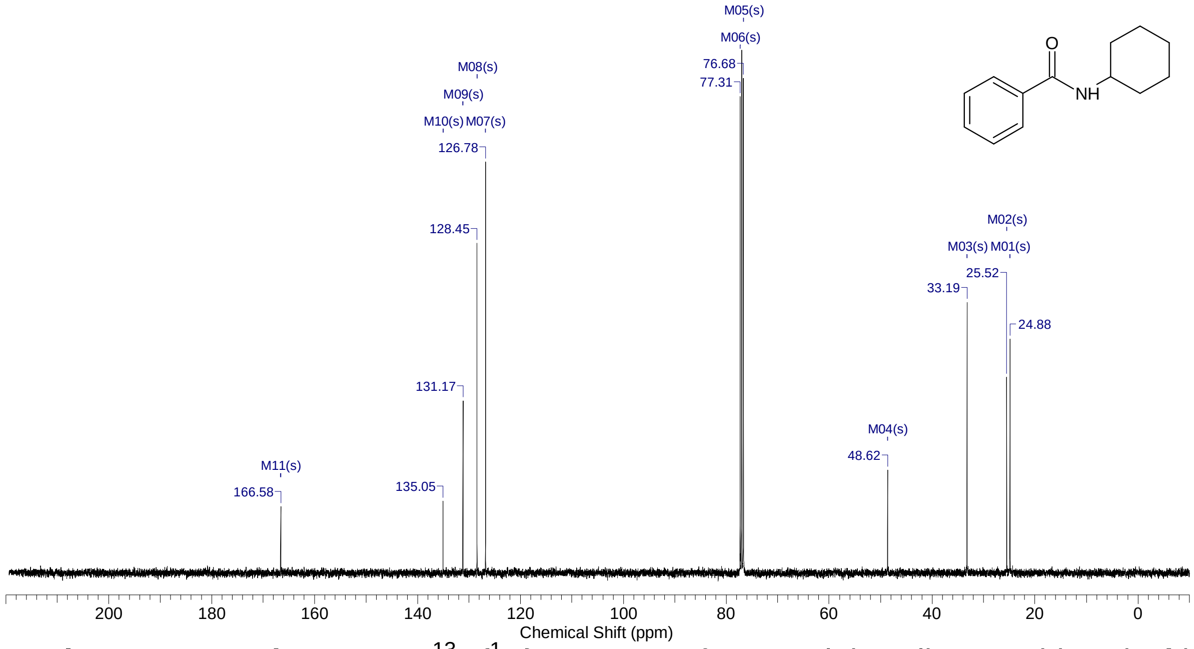

Supplementary Figure 65. ^{13}C{^{1}H} NMR of *N*-cyclohexylbenzamide (**5ab**) (Chloroform-d_3. 400 MHz).

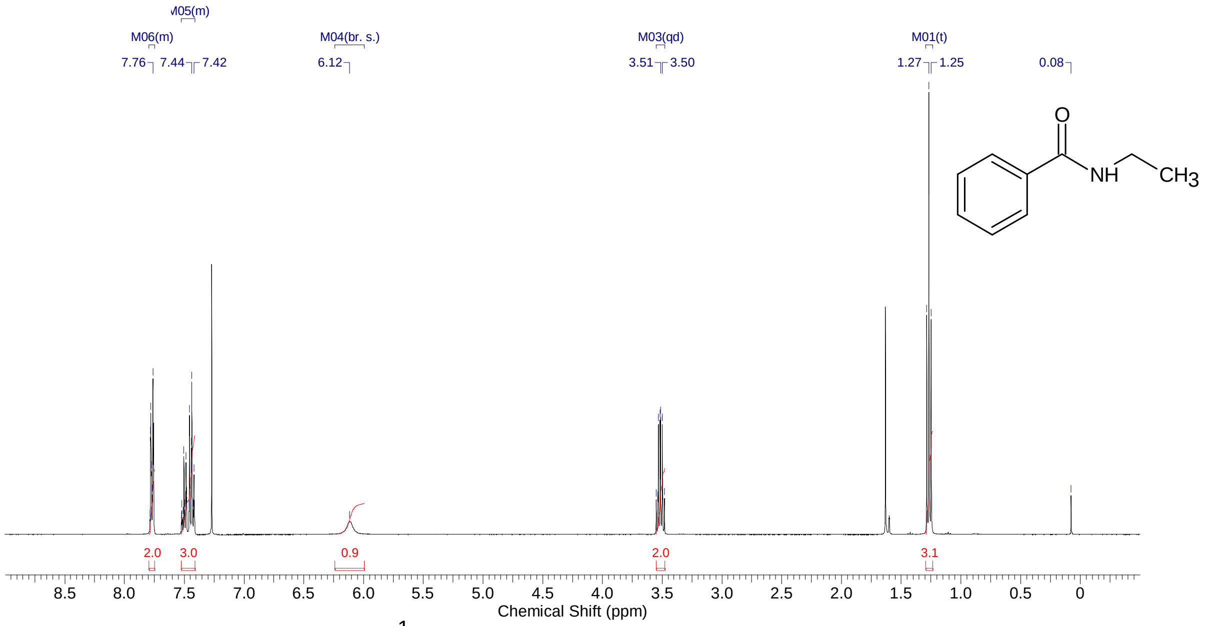

Supplementary Figure 66. ^{1}H NMR of *N*-ethylbenzamide (**5ac**) (Chloroform-d_3, 400 MHz).

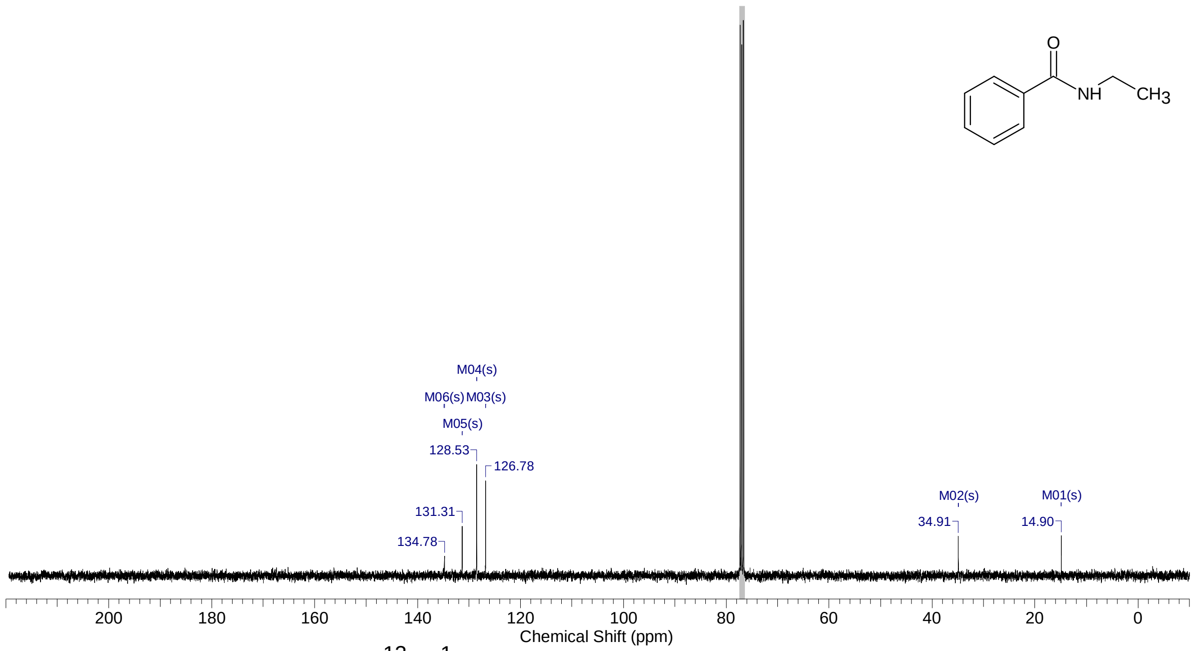

Supplementary Figure 67.^{13}C{^{1}H} NMR of *N*-ethylbenzamide (**5ac**) (Chloroform-d_3. 400 MHz).

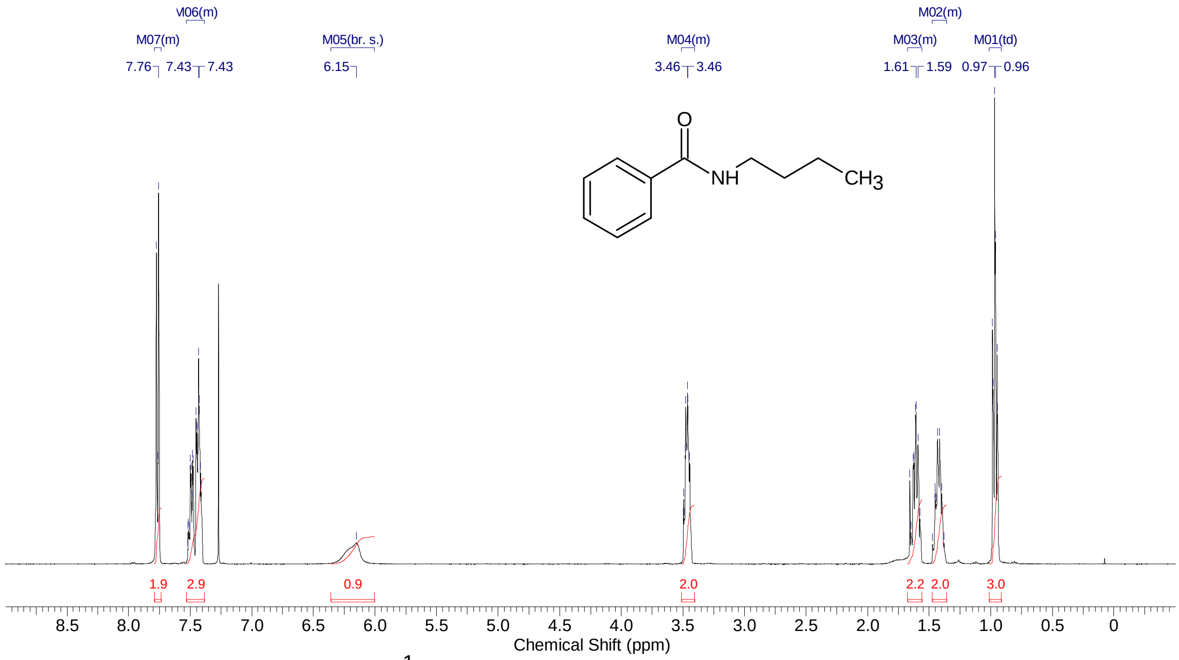

Supplementary Figure 68. ^{1}H NMR of *N*-(*n*-butyl)benzamide (**5ad**) (Chloroform-d_3, 400 MHz).

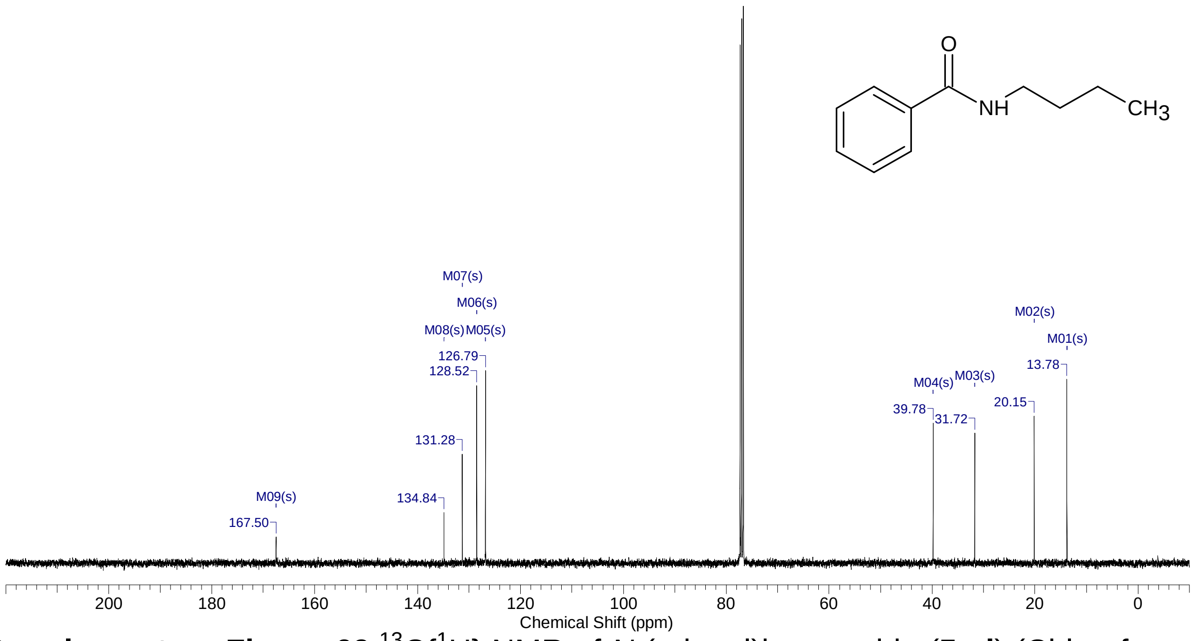

Supplementary Figure 69.^{13}C{^{1}H} NMR of *N*-(*n*-butyl)benzamide (**5ad**) (Chloroform-d_3. 400 MHz).

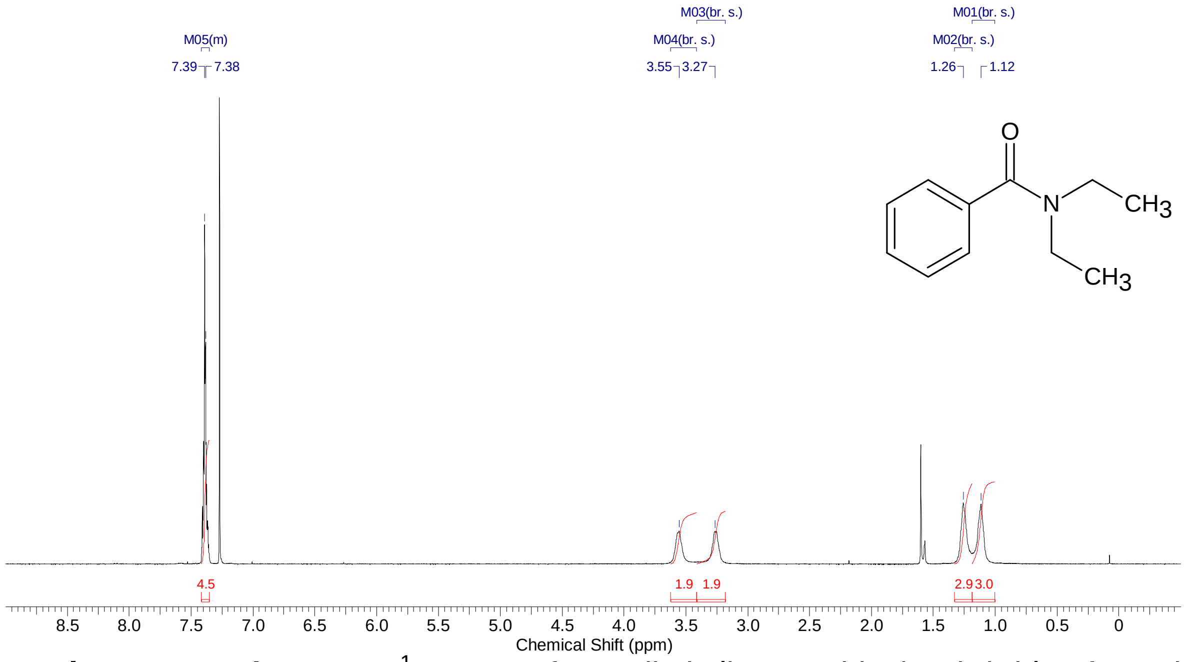

Supplementary Figure 70. ^{1}H NMR of *N,N*-diethylbenzamide (**5ae**) (Chloroform-d_3, 400 MHz).

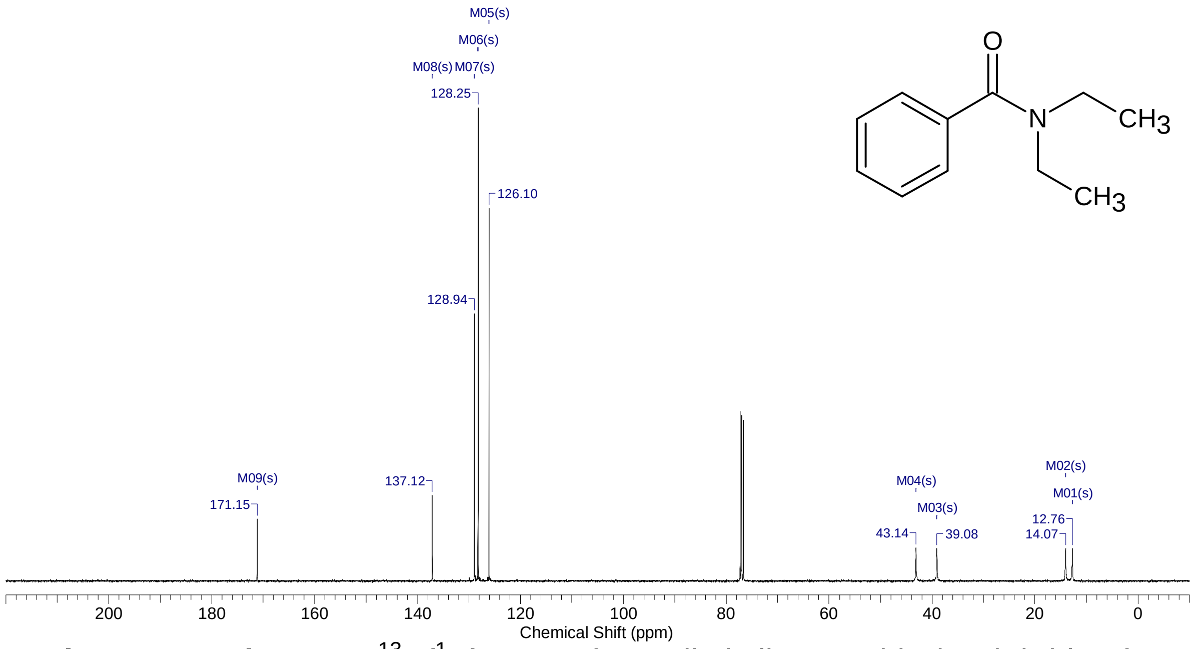

Supplementary Figure 71. ^{13}C{^{1}H} NMR of *N,N*-diethylbenzamide (**5ae**) (Chloroform-d_3. 400 MHz).

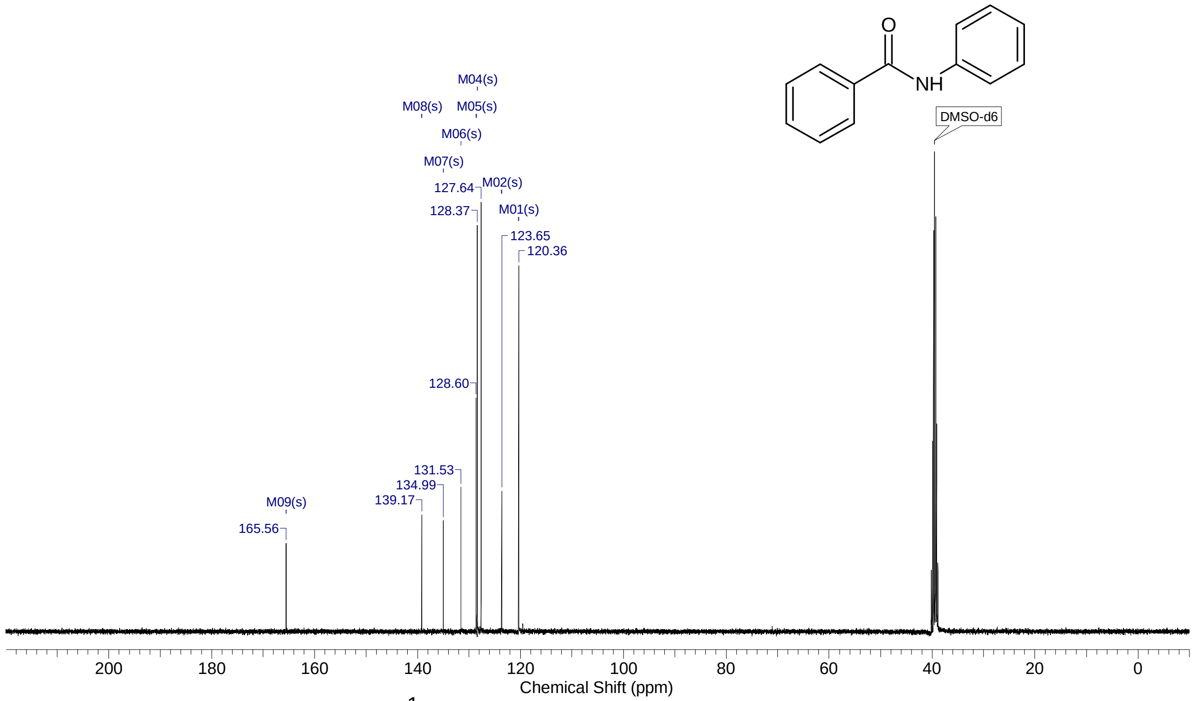

Supplementary Figure 72. ^{1}H NMR of *N*-phenylbenzamide (**5af**) (DMSO-d_6, 400 MHz).

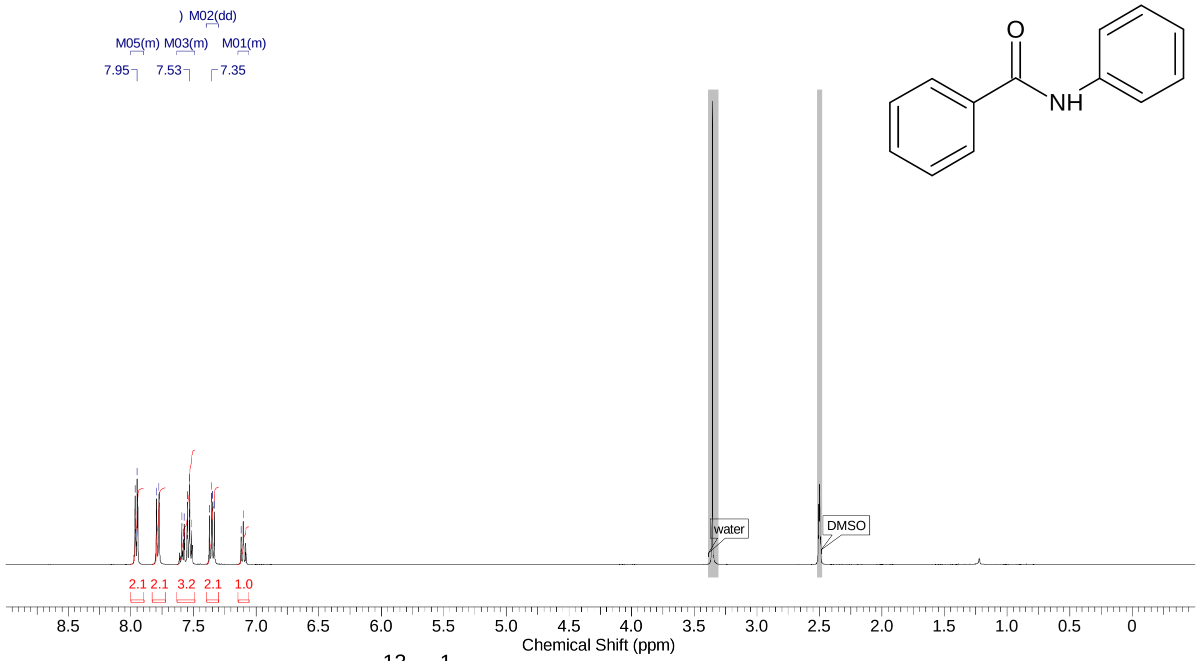

Supplementary Figure 73.^{13}C{^{1}H} NMR of *N*-phenylbenzamide (**5af**) (DMSO-d_6. 400 MHz).

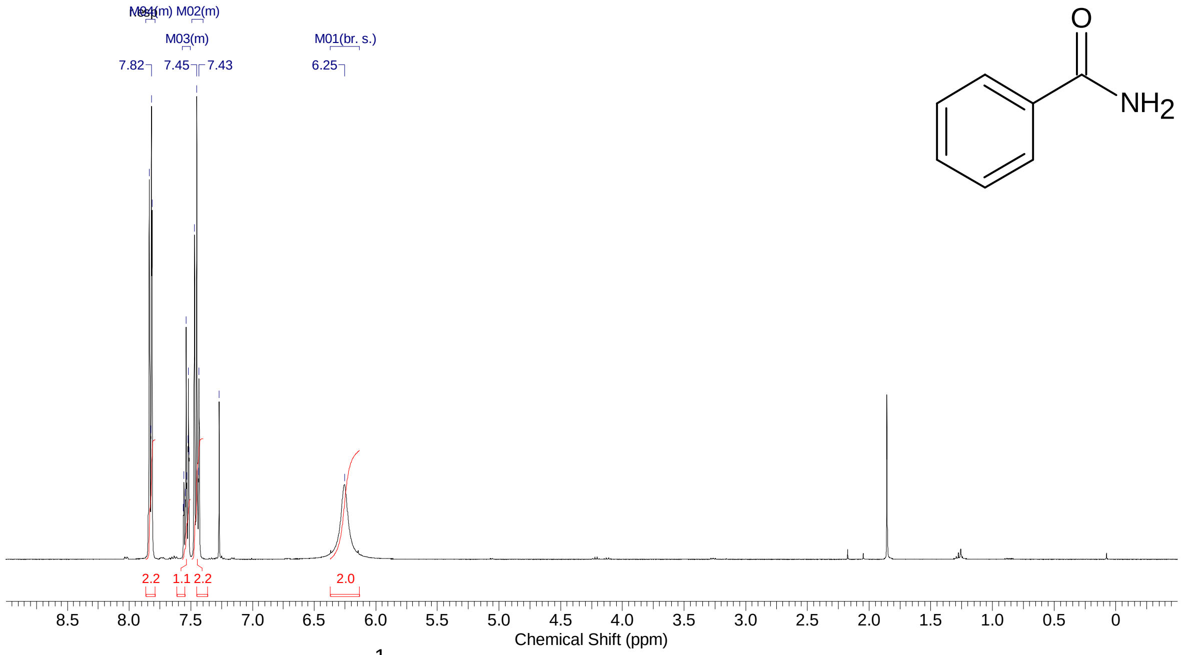

Supplementary Figure 74. ^{1}H NMR of benzamide (**5ag**) (Chloroform-d_3, 400 MHz).

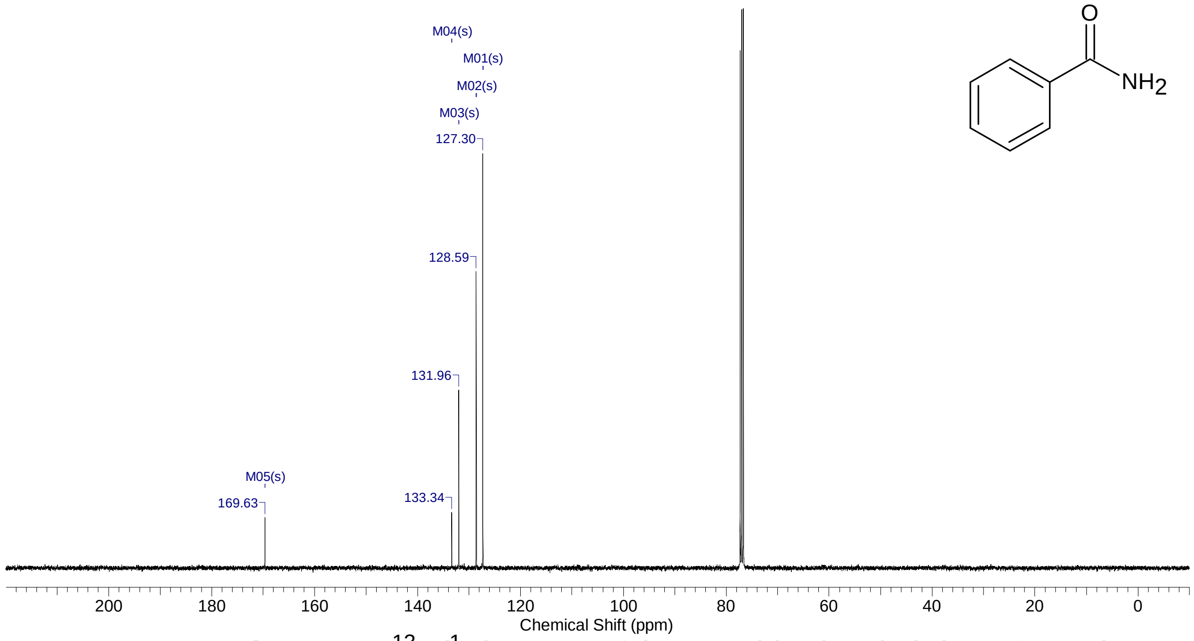

Supplementary Figure 75. ^{13}C{^{1}H} NMR of benzamide (**5ag**) (Chloroform-d_3. 400 MHz).

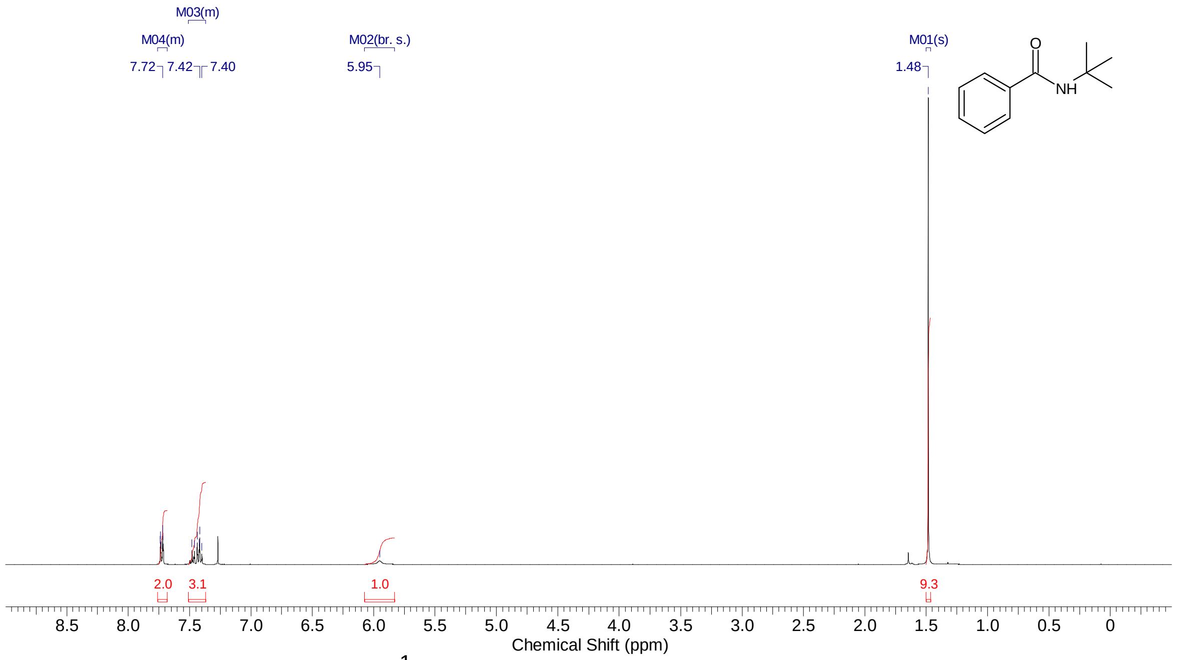

Supplementary Figure 76. ^{1}H NMR of *N*-*tert*-butylbenzamide (**5ah**) (Chloroform-d_3, 400 MHz).

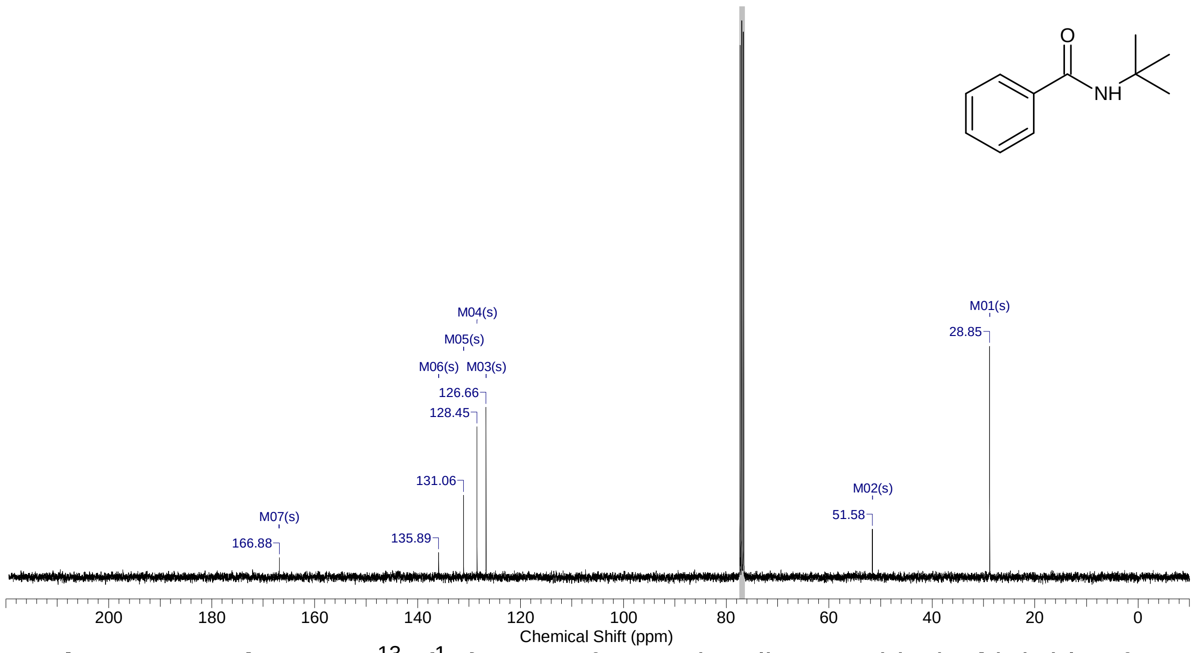

Supplementary Figure 77. ^{13}C{^{1}H} NMR of *N*-*tert*-butylbenzamide (**5ah**) (Chloroform-d_3. 400 MHz).

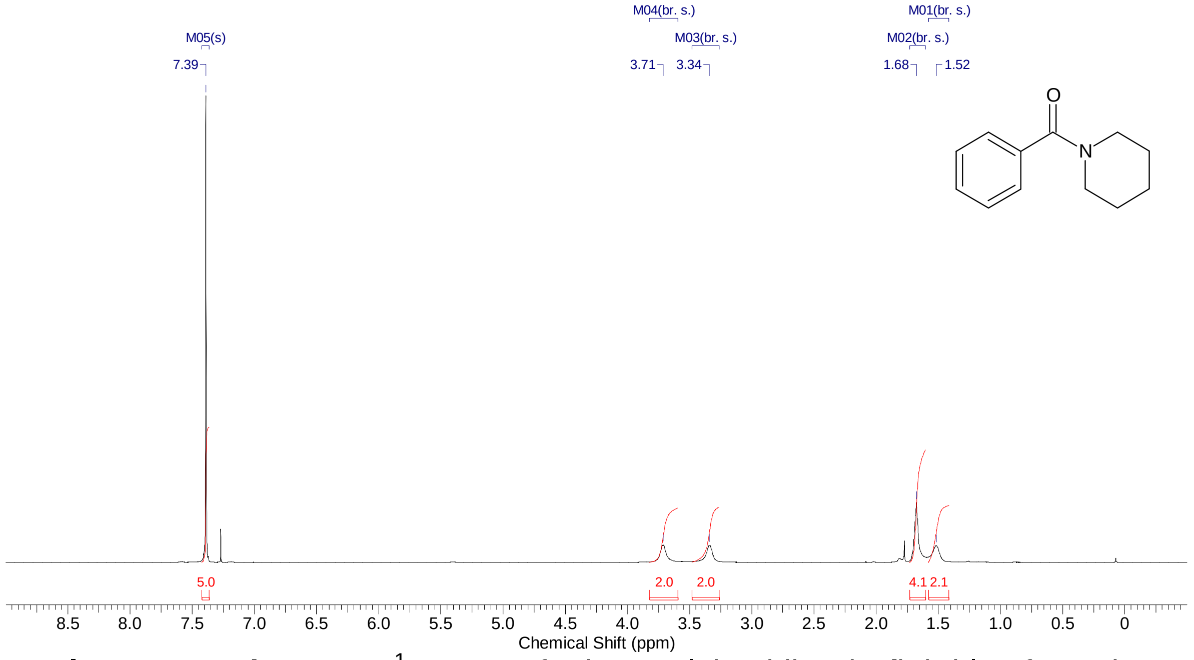

Supplementary Figure 78. ^{1}H NMR of 4-benzoylpiperidine (**5ai**) (Chloroform-d_3, 400 MHz).

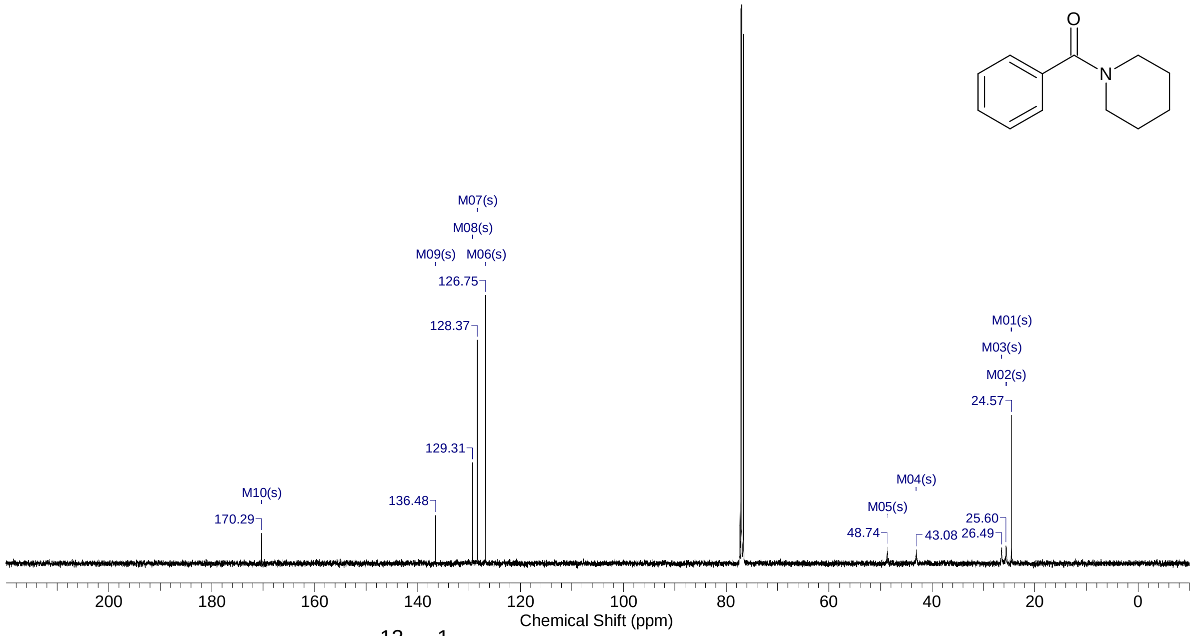

Supplementary Figure 79.^{13}C{^{1}H} NMR of 4-benzoylpiperidine (**5ai**) (Chloroform-d_3. 400 MHz).

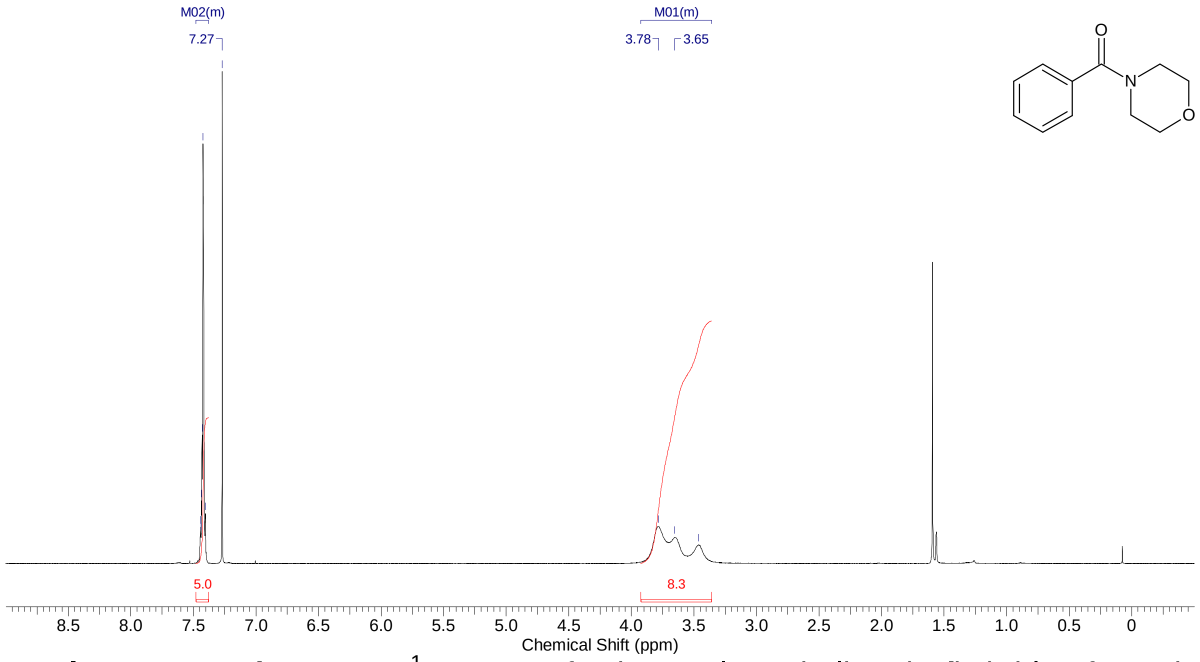

Supplementary Figure 80. ^{1}H NMR of 4-benzoylmorpholine (**5aj**) (Chloroform-d_3, 400 MHz).

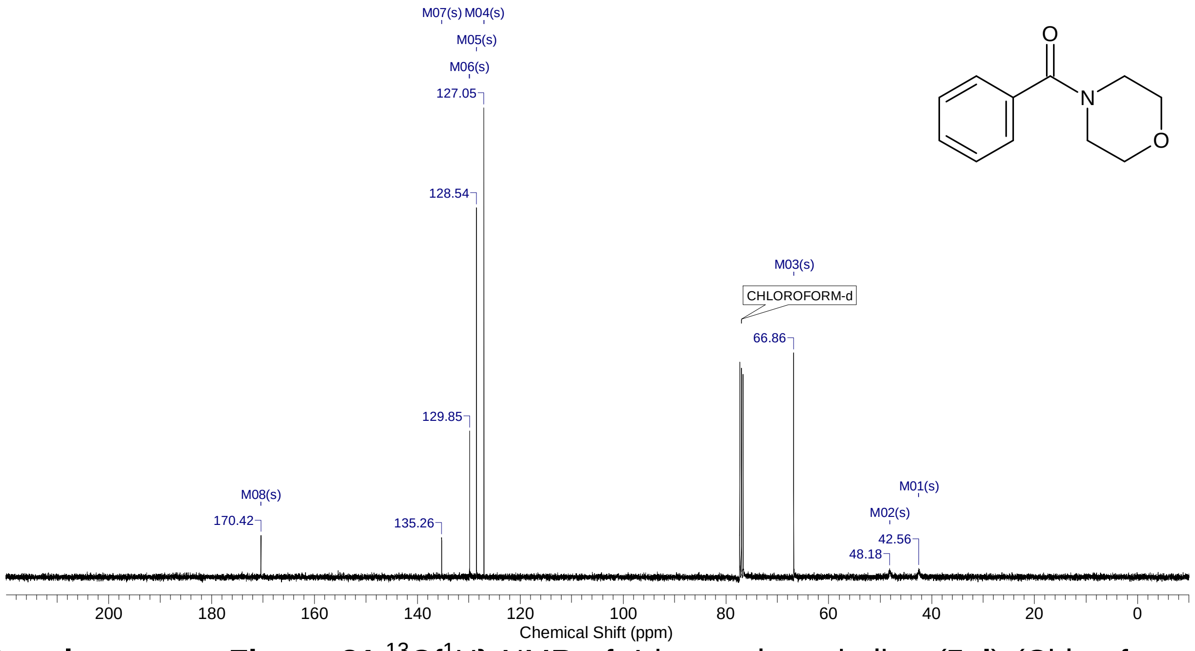

Supplementary Figure 81.$^{13}C\{^{1}H\}$ NMR of 4-benzoylmorpholine (**5aj**) (Chloroform-d_3. 400 MHz).

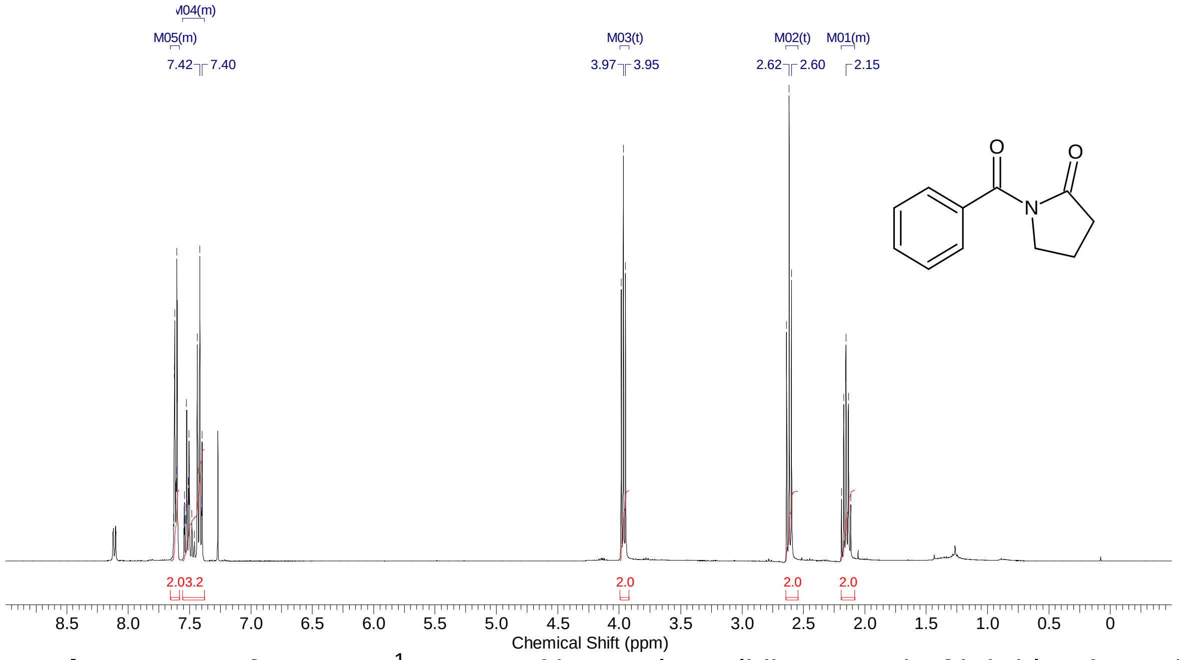

Supplementary Figure 82. ^{1}H NMR of benzoylpyrrolidin-2-one (**5ak**) (Chloroform-d_3, 400 MHz).

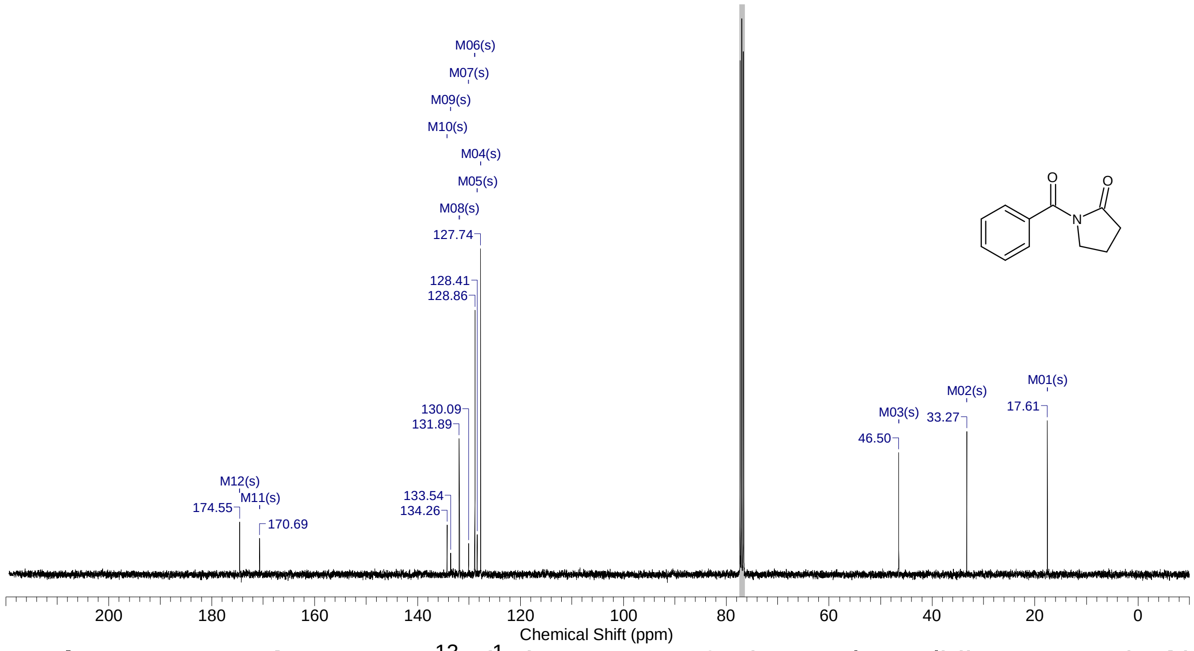

Supplementary Figure 83. ^{13}C{^{1}H} NMR of benzoylpyrrolidin-2-one (**5ak**) (Chloroform-d_3. 400 MHz).

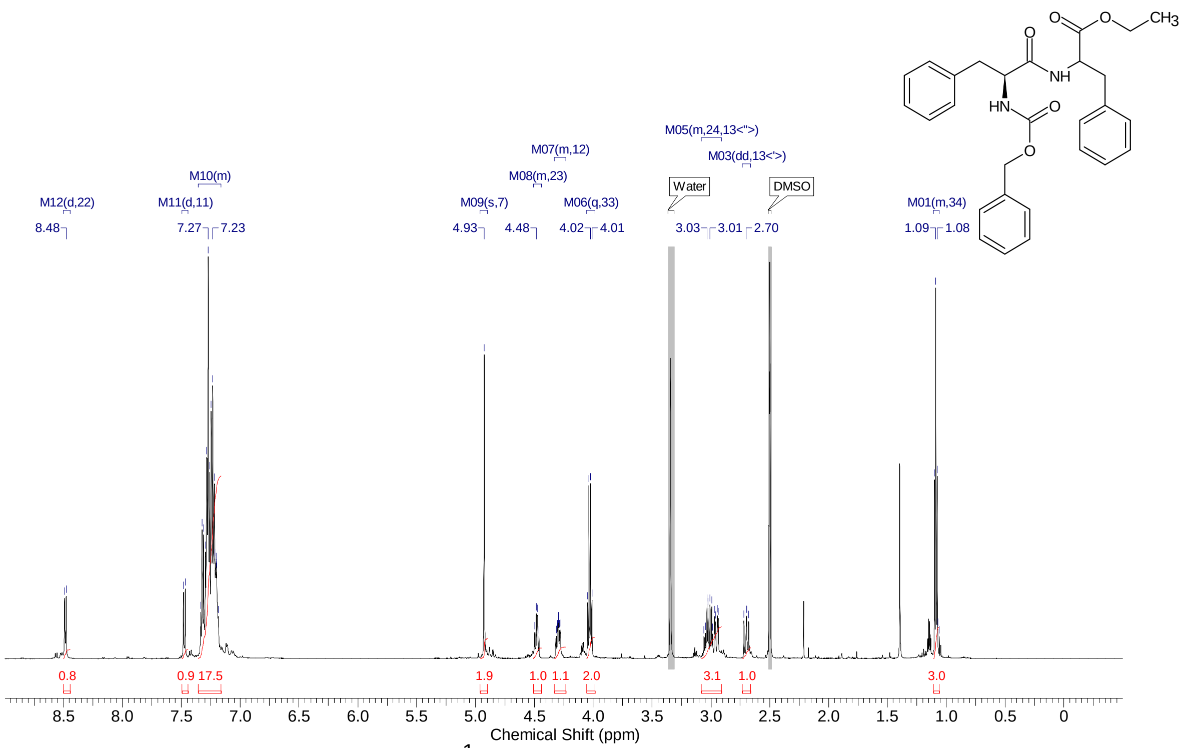

Supplementary Figure 84. ^{1}H NMR of *N*-carboxybenzoyl-L-phenylalanyl-L-phenylalanine ethyl ester (**5acl**) (DMSO-d_6, 600 MHz).

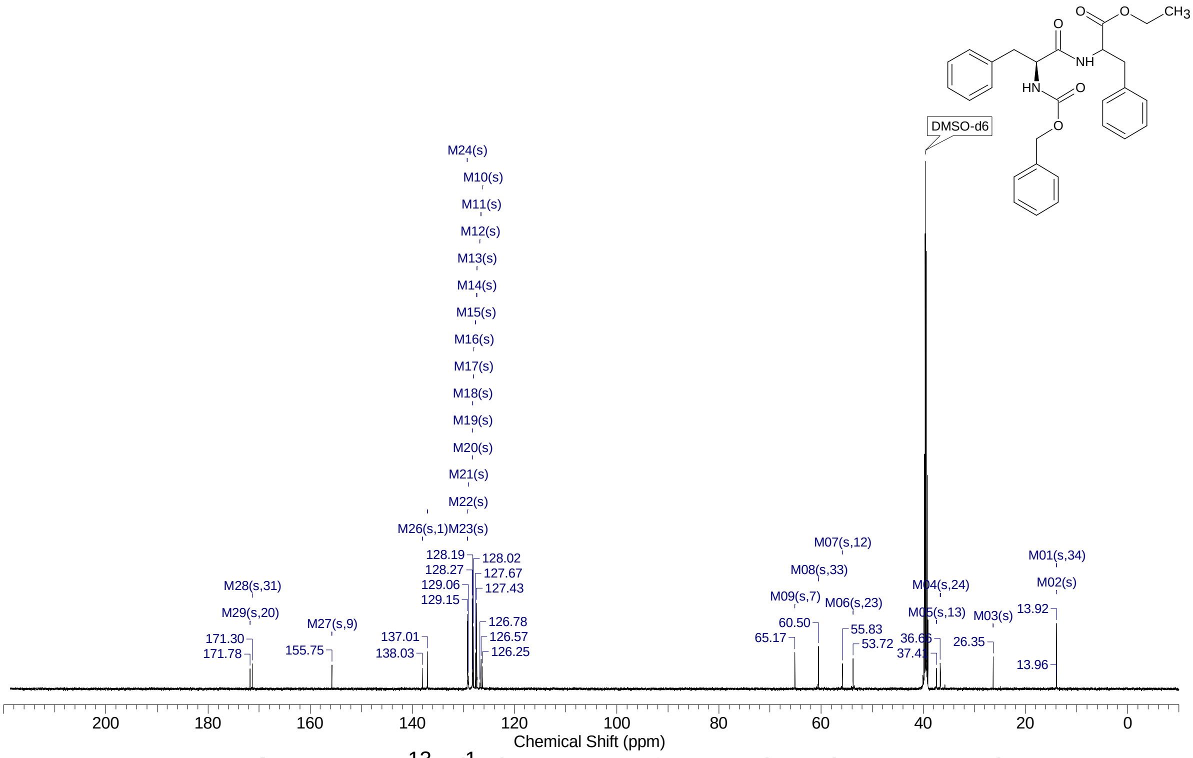

Supplementary Figure 85. $^{13}C\{^{1}H\}$ NMR of *N*-carboxybenzoyl-L-phenylalanyl-L-phenylalanine ethyl ester (**5acl**) (DMSO-d_6. 400 MHz).

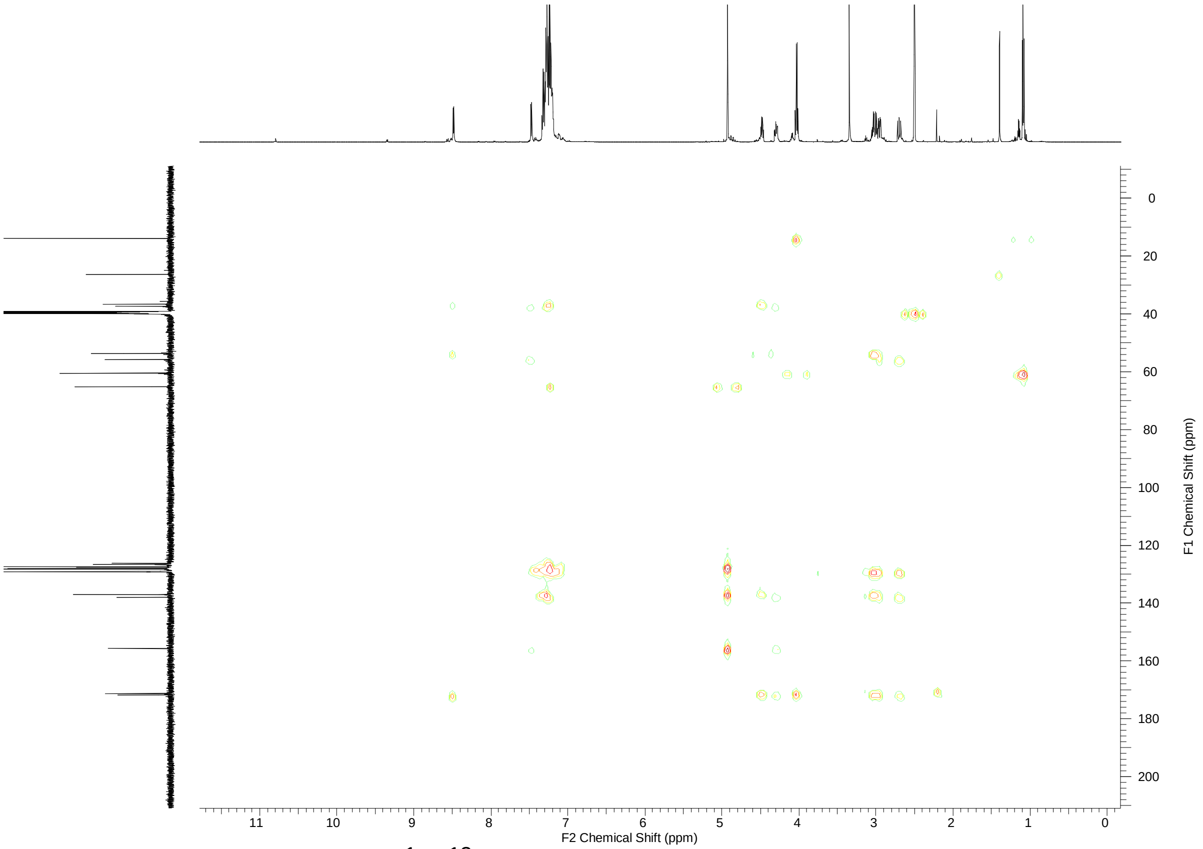

Supplementary Figure 86. $^{1}H/^{13}C$ HMBC NMR of *N*-carboxybenzoyl-L-phenylalanyl-L-phenylalanine ethyl ester (**5acl**) (DMSO-d_6, 600 MHz).

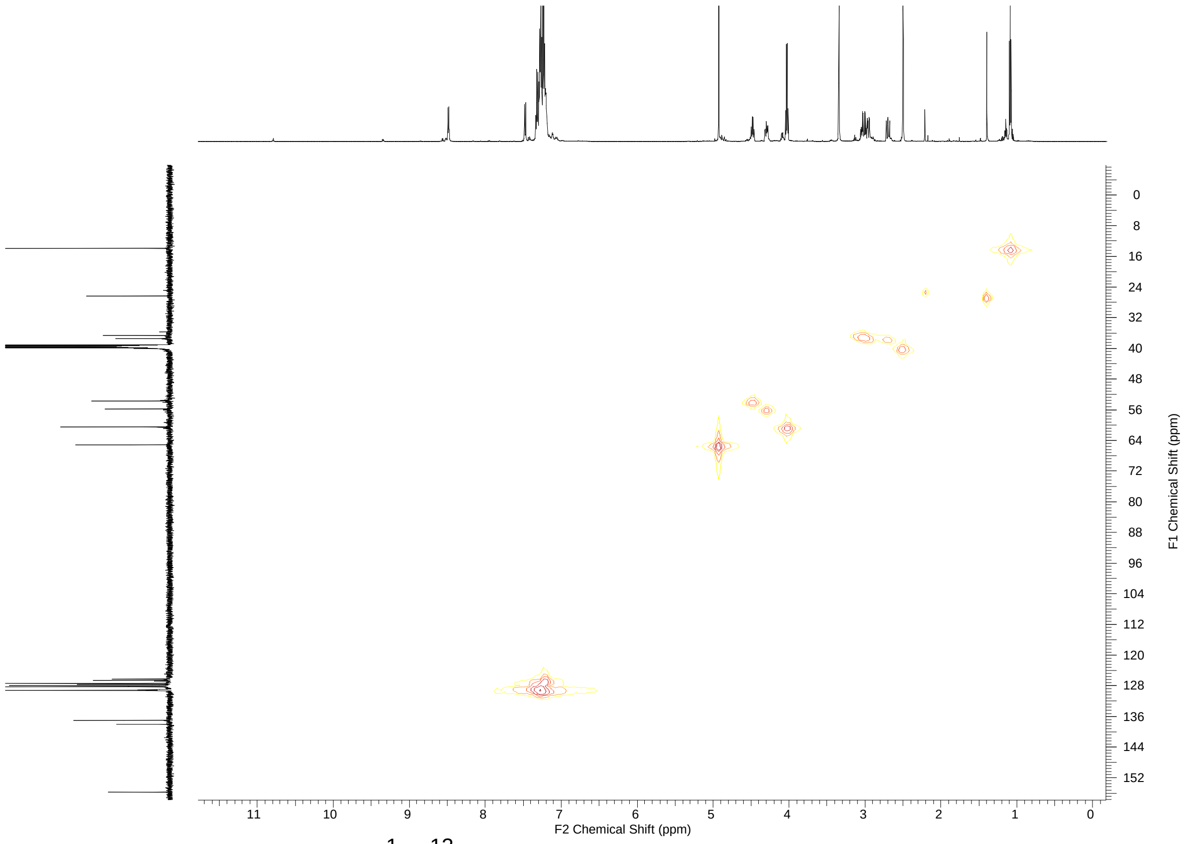

Supplementary Figure 87. $^{1}H/^{13}C$ HSQC NMR of *N*-carboxybenzoyl-L-phenylalanyl-L-phenylalanine ethyl ester (**5acl**) (DMSO-d_6, 600 MHz).

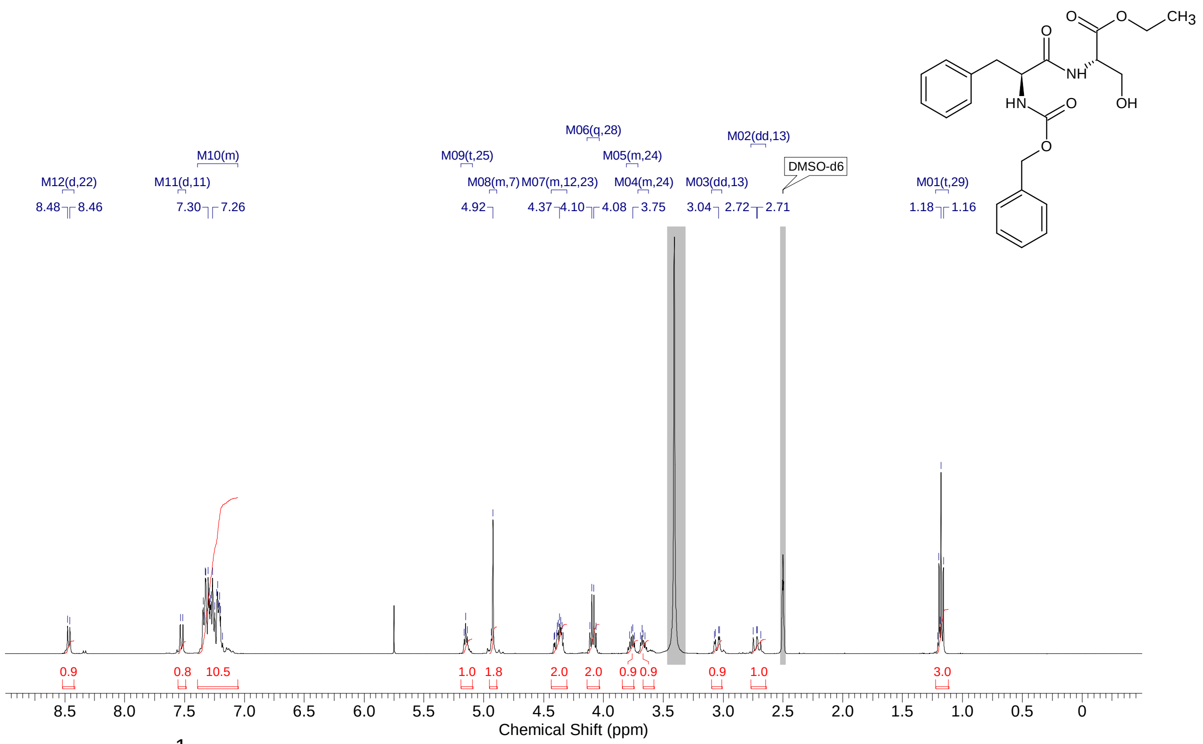

Figure 88. ^{1}H NMR of *N*-(*N*-carboxybenzoxyl-L-phenylalanyl)-L-serin ethyl ester (**5acm**) (DMSO-d_6, 600 MHz).

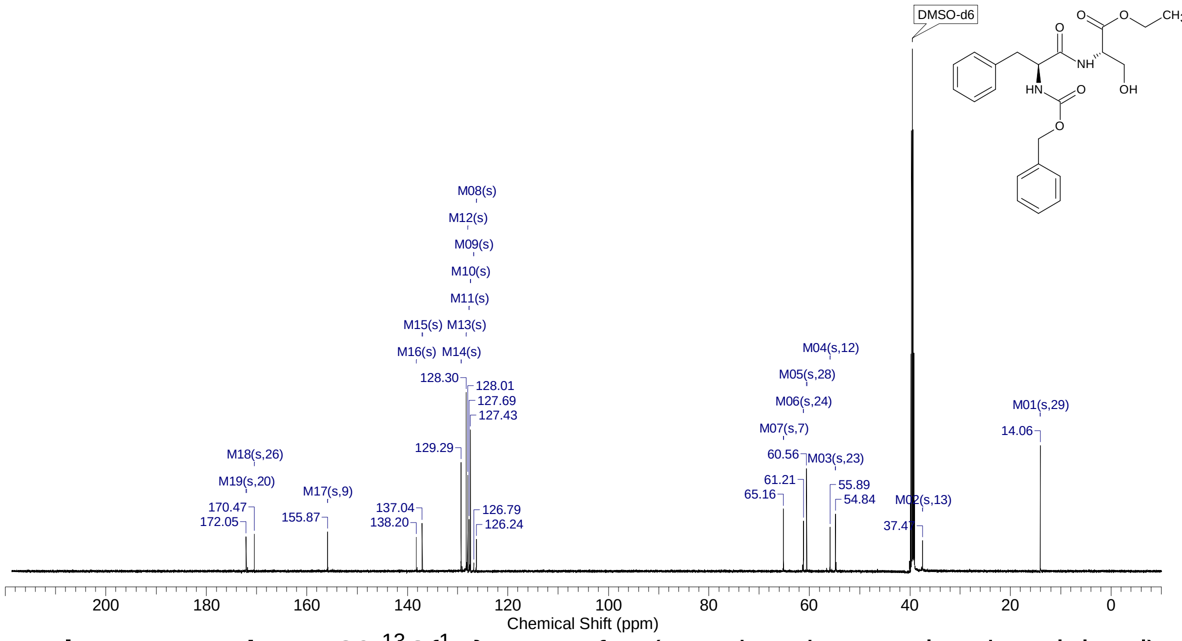

Supplementary Figure 89.^{13}C{^{1}H} NMR of *N*-(*N*-carboxybenzoxyl-L-phenylalanyl)-L-serin ethyl ester (**5acm**) (DMSO-d_6. 400 MHz).

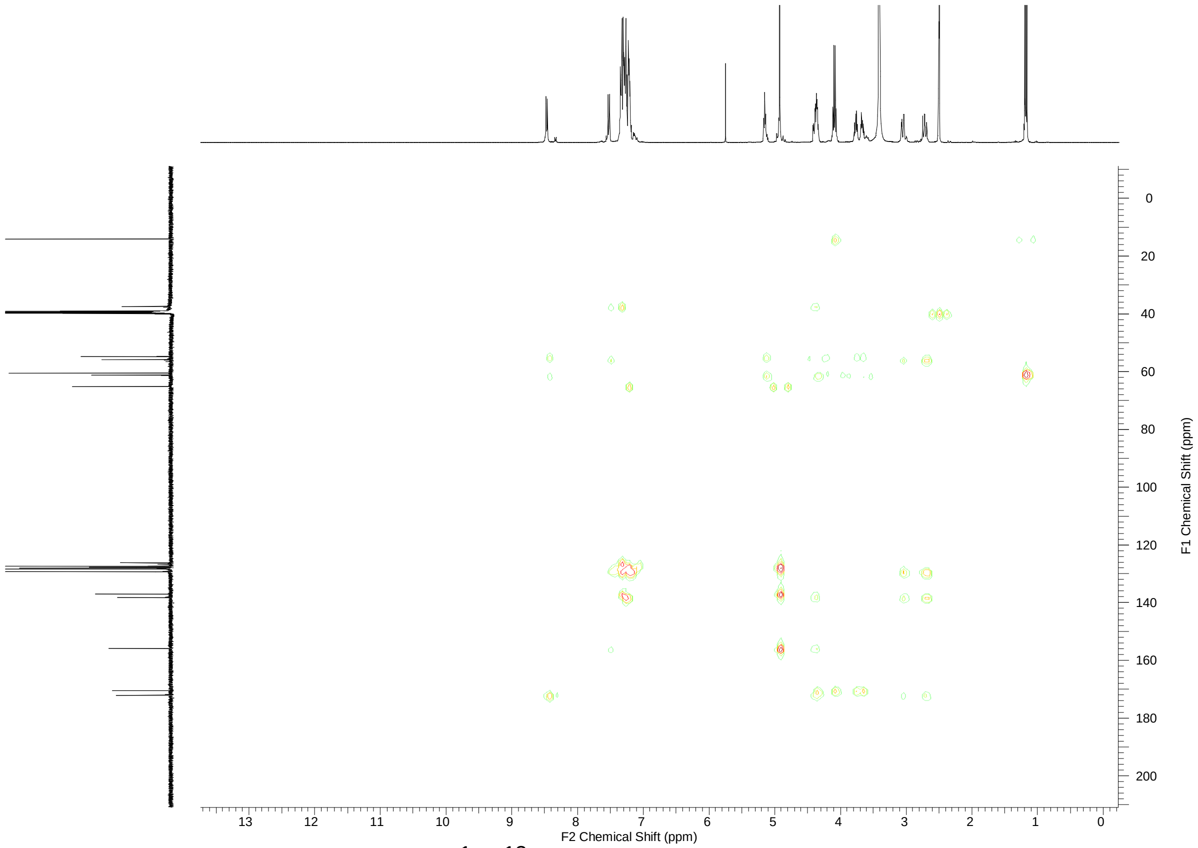

Supplementary Figure 90. $^{1}H/^{13}C$ HMBC NMR of *N*-(*N*-carboxybenzoxyl-L-phenylalanyl)-L-serin ethyl ester (**5acm**) (DMSO-d_6, 600 MHz).

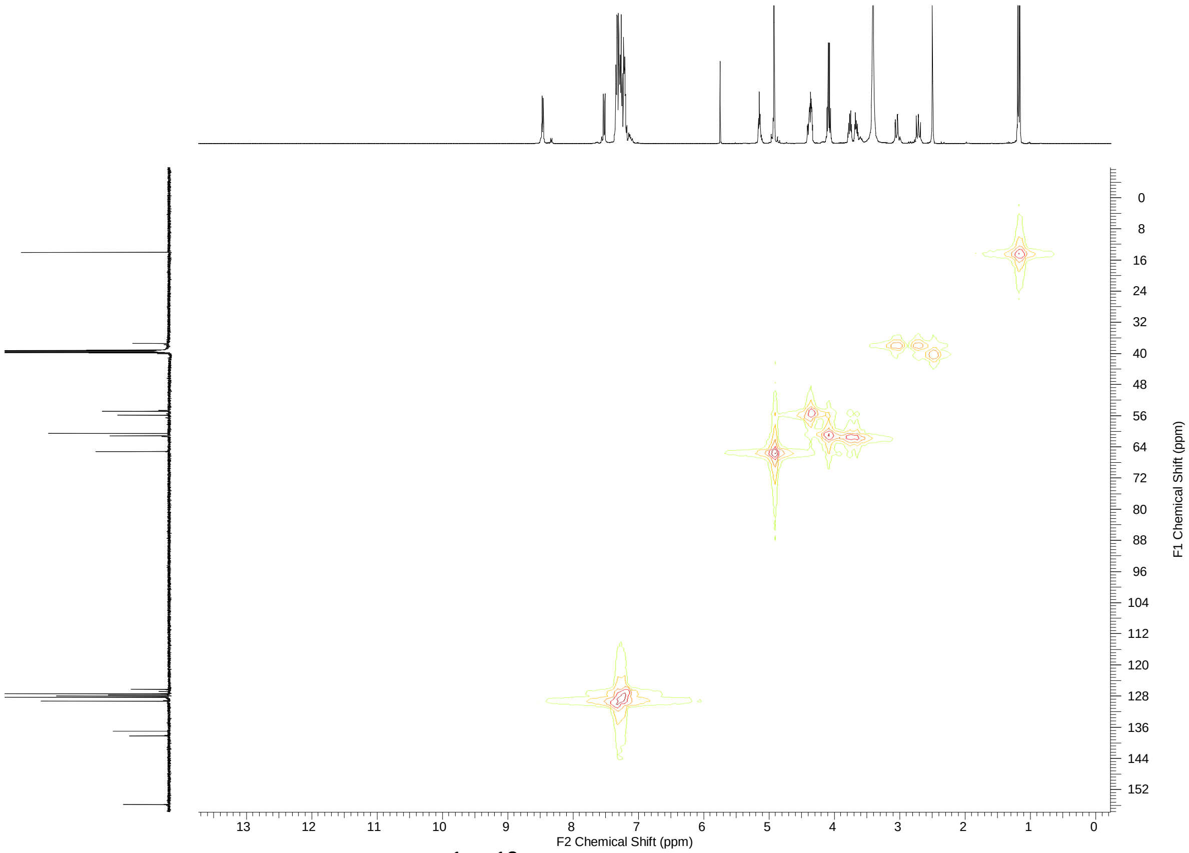

Supplementary Figure 91. $^{1}H/^{13}C$ HSQC NMR of *N*-(*N*-carboxybenzoxyl-L-phenylalanyl)-L-serin ethyl ester (**5acm**) (DMSO-d_6, 600 MHz).

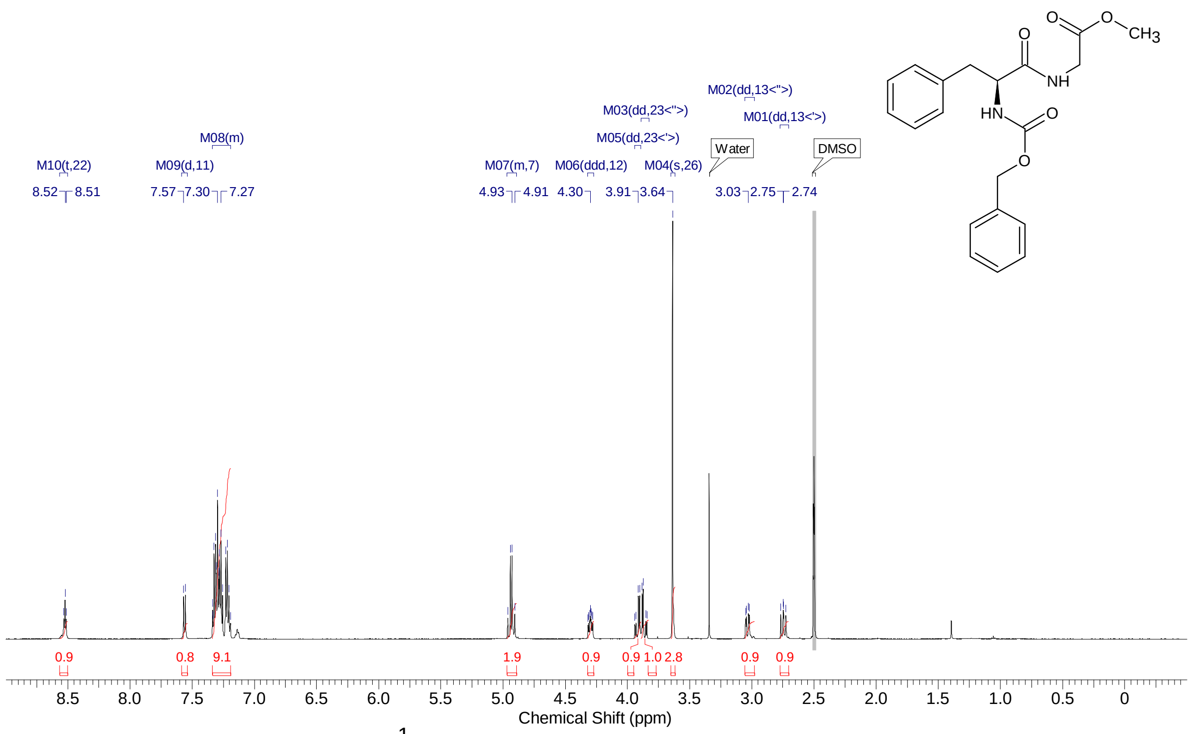

Supplementary Figure 92. ^{1}H NMR of *N*-(*N*-carboxybenzoyl-L-phenylalanyl)-glycine methyl ester (**5acn**) (DMSO-d_6, 600 MHz).

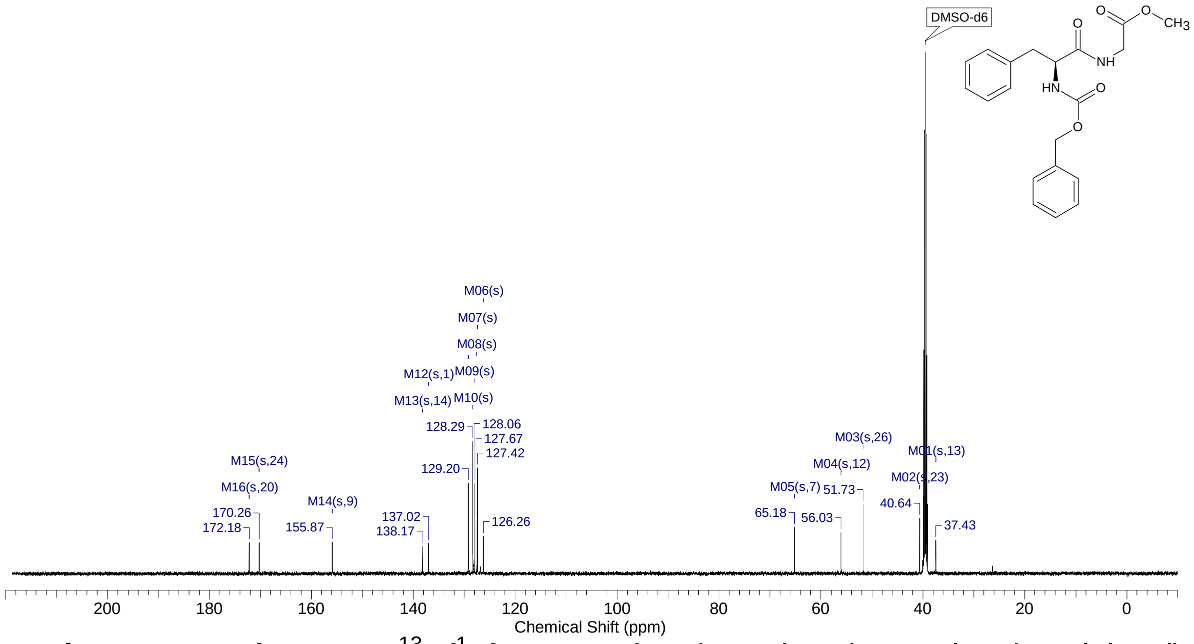

Supplementary Figure 93.^{13}C{^{1}H} NMR of *N*-(*N*-carboxybenzoyl-L-phenylalanyl)-glycine methyl ester (**5acn**) (DMSO-d_6. 400 MHz).

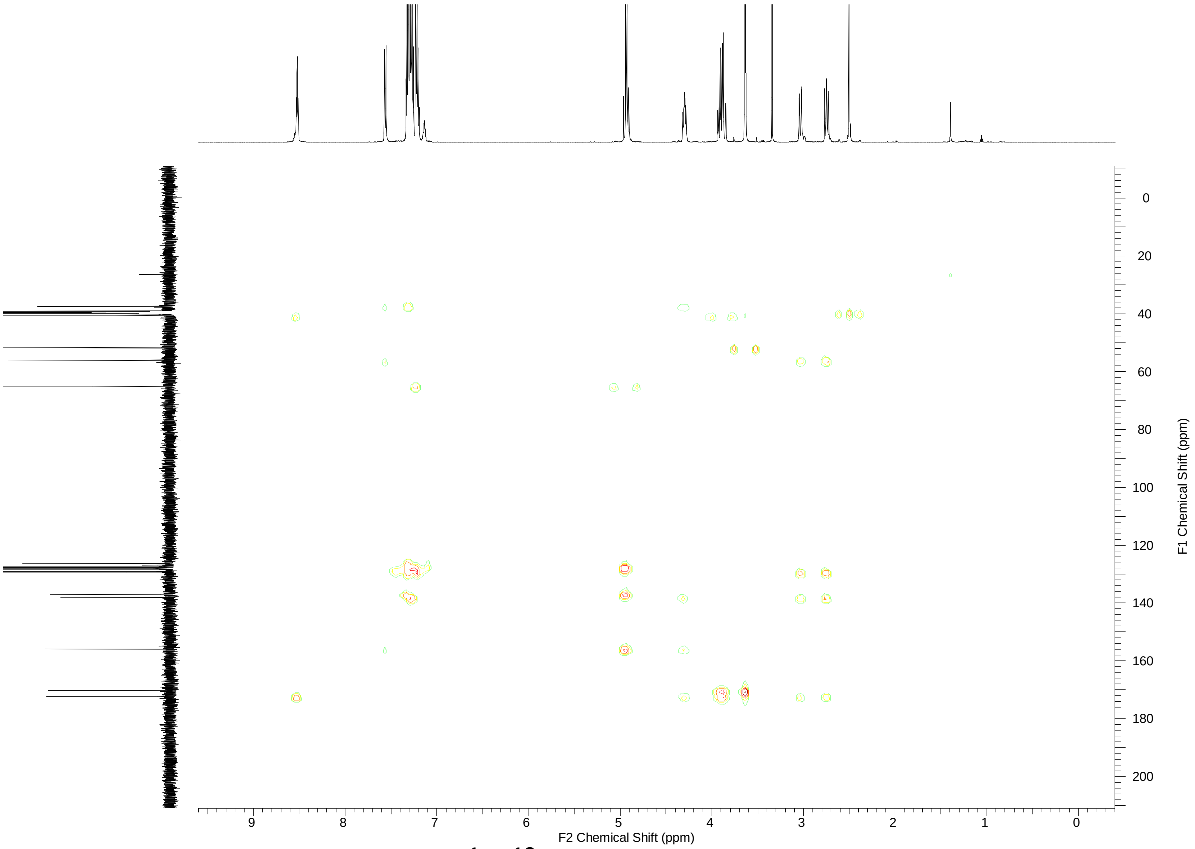

Supplementary Figure 94. $^{1}H/^{13}C$ HMBC NMR of *N*-(*N*-carboxybenzoyl-L-phenylalanyl)-glycine methyl ester (**5acn**) (DMSO-d_6, 600 MHz).

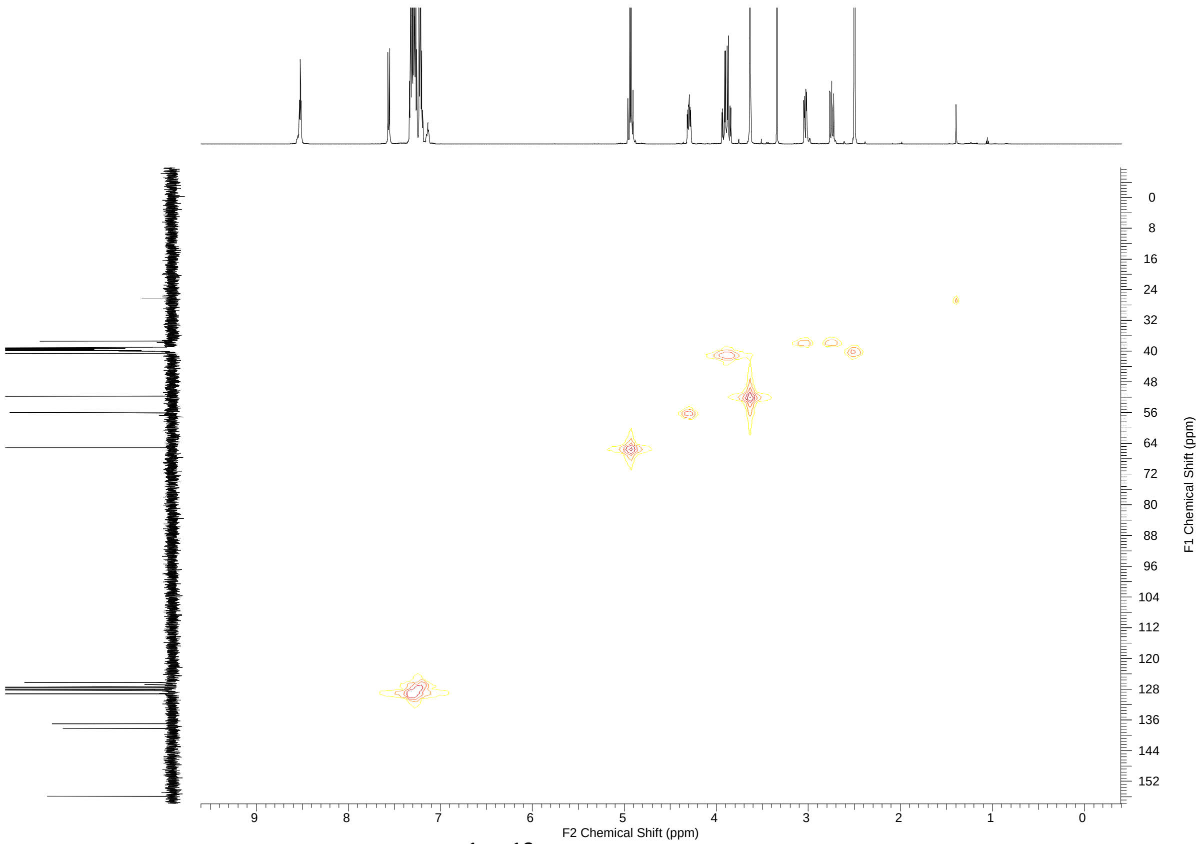

Supplementary Figure 95. $^{1}H/^{13}C$ HSQC NMR of *N*-(*N*-carboxybenzoyl-L-phenylalanyl)-glycine methyl ester (**5acn**) (DMSO-d_6, 600 MHz).

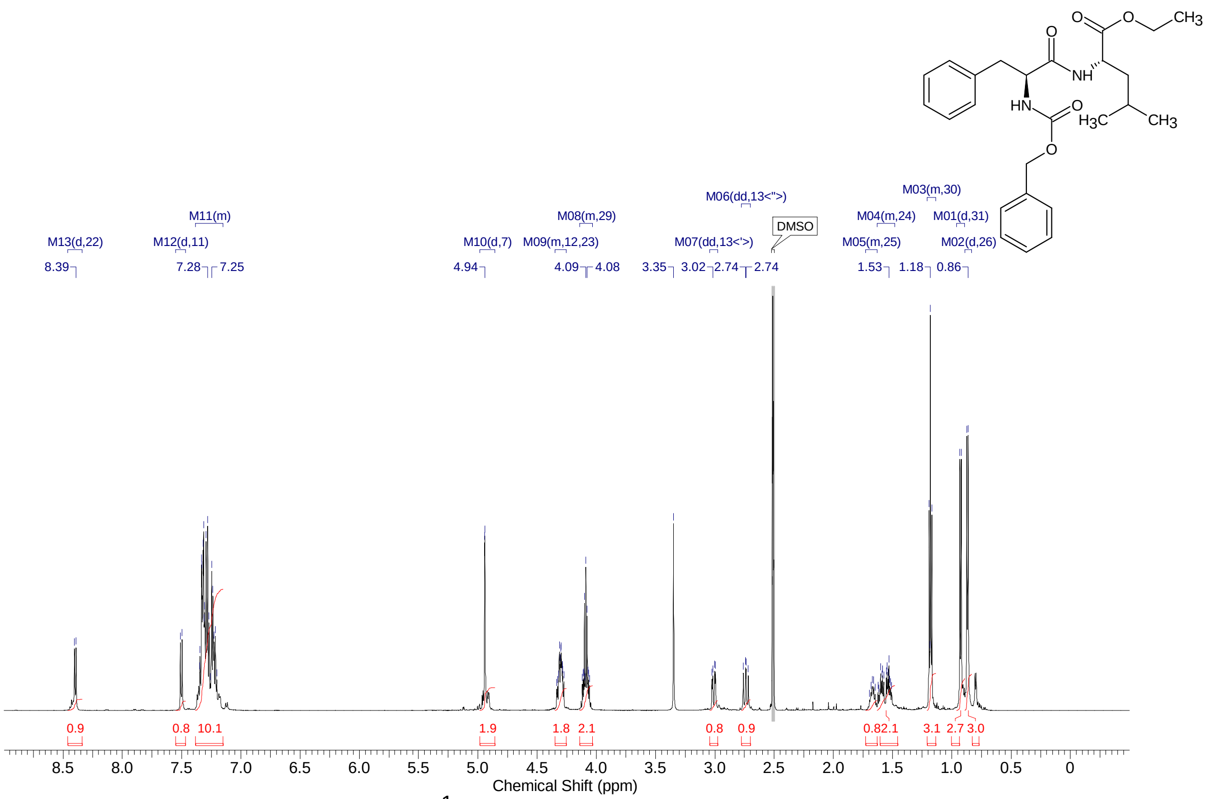

Supplementary Figure 96. ^{1}H NMR of *N*-(*N*-carboxybenzoyl-L-phenylalanyl)-L-leucine ethyl ester (**5aco**) (DMSO-d_6, 600 MHz).

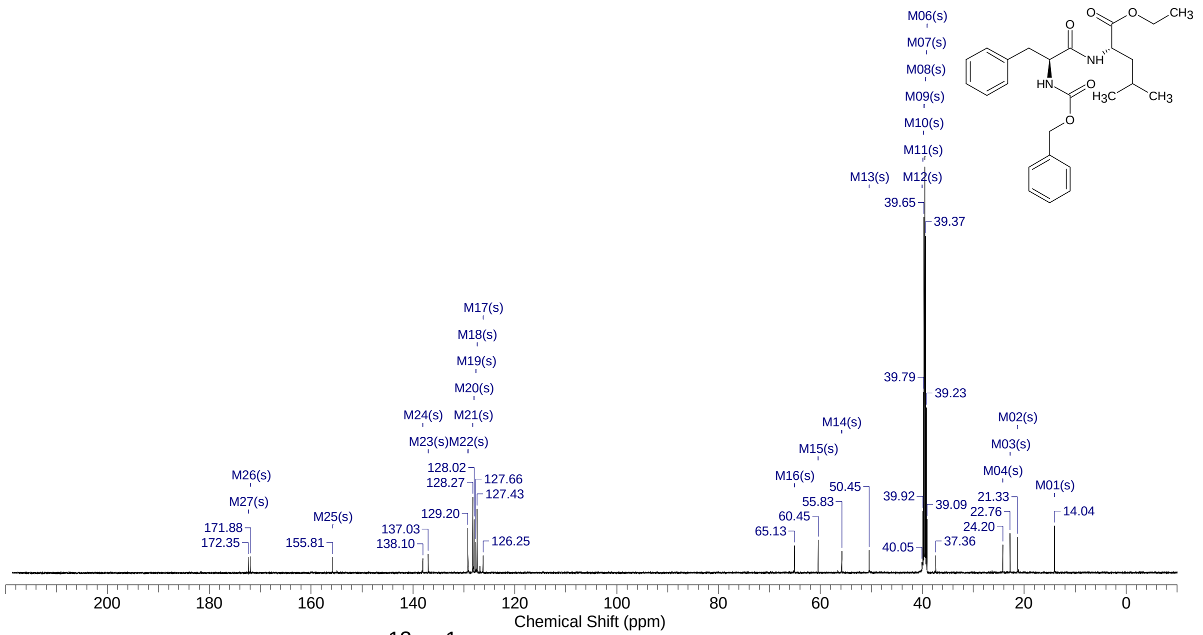

Supplementary Figure 97.$^{13}C\{^{1}H\}$ NMR of *N*-(*N*-carboxybenzoyl-L-phenylalanyl)-L-leucine ethyl ester (**5aco**) (DMSO-d_6. 400 MHz).

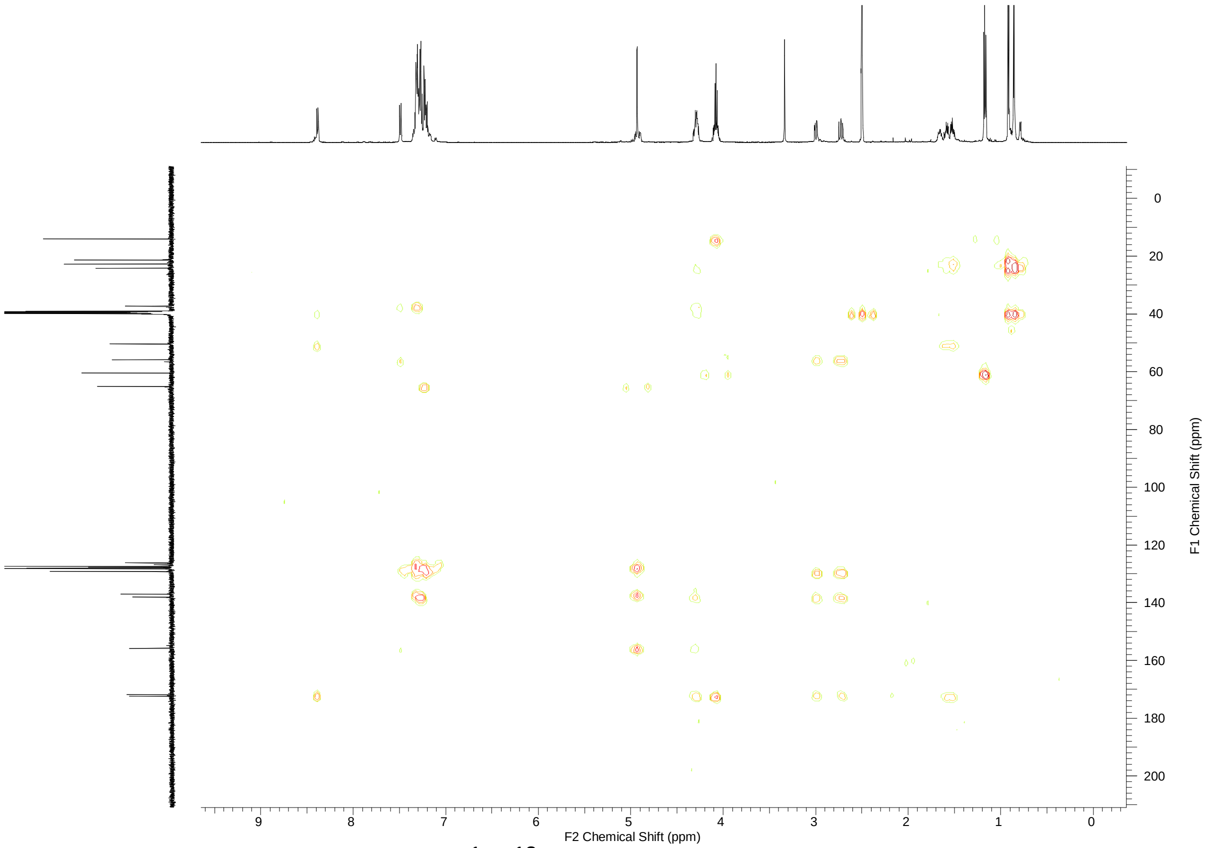

Supplementary Figure 98. $^1H/^{13}C$ HMBC NMR of *N*-(*N*-carboxybenzoyl-L-phenylalanyl)-L-leucine ethyl ester (**5aco**) (DMSO-d_6, 600 MHz).

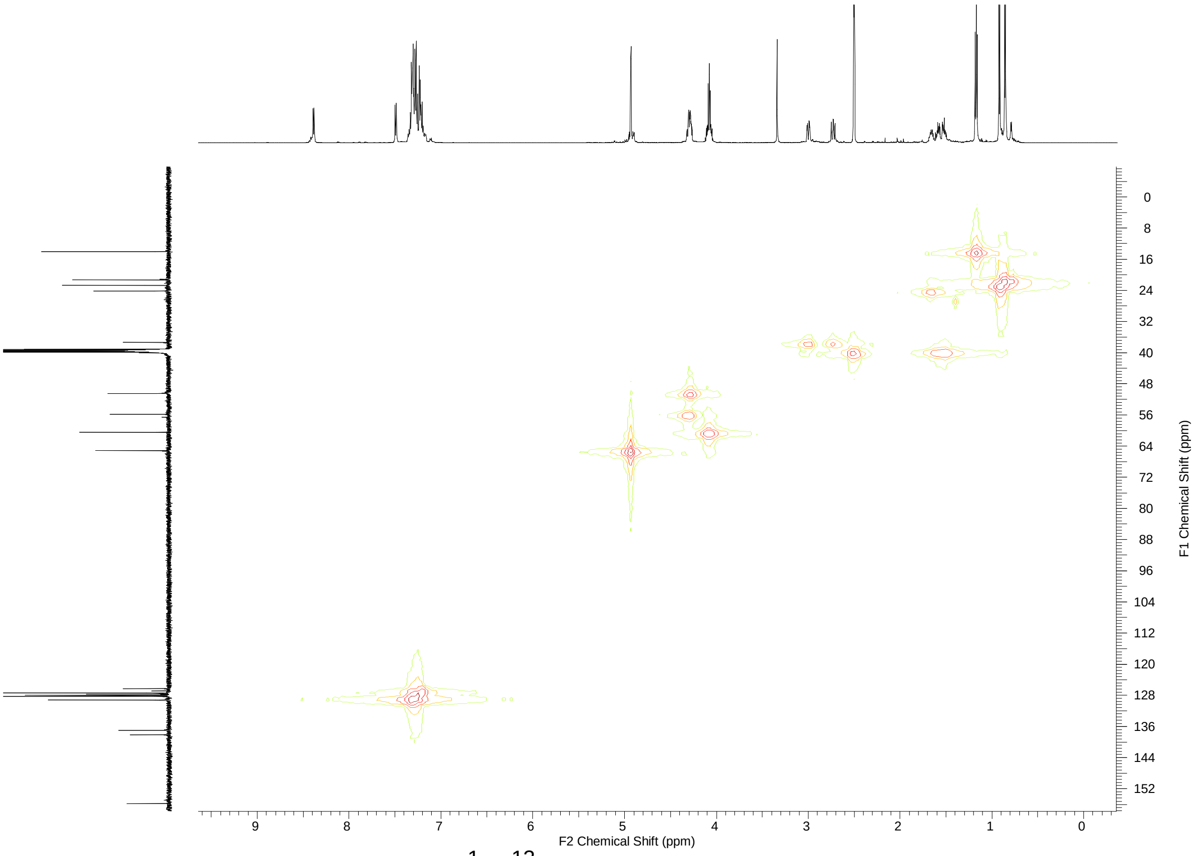

Supplementary Figure 99. $^{1}H/^{13}C$ HSQC NMR of *N*-(*N*-carboxybenzoyl-L-phenylalanyl)-L-leucine ethyl ester (**5aco**) (DMSO-d_6, 600 MHz).

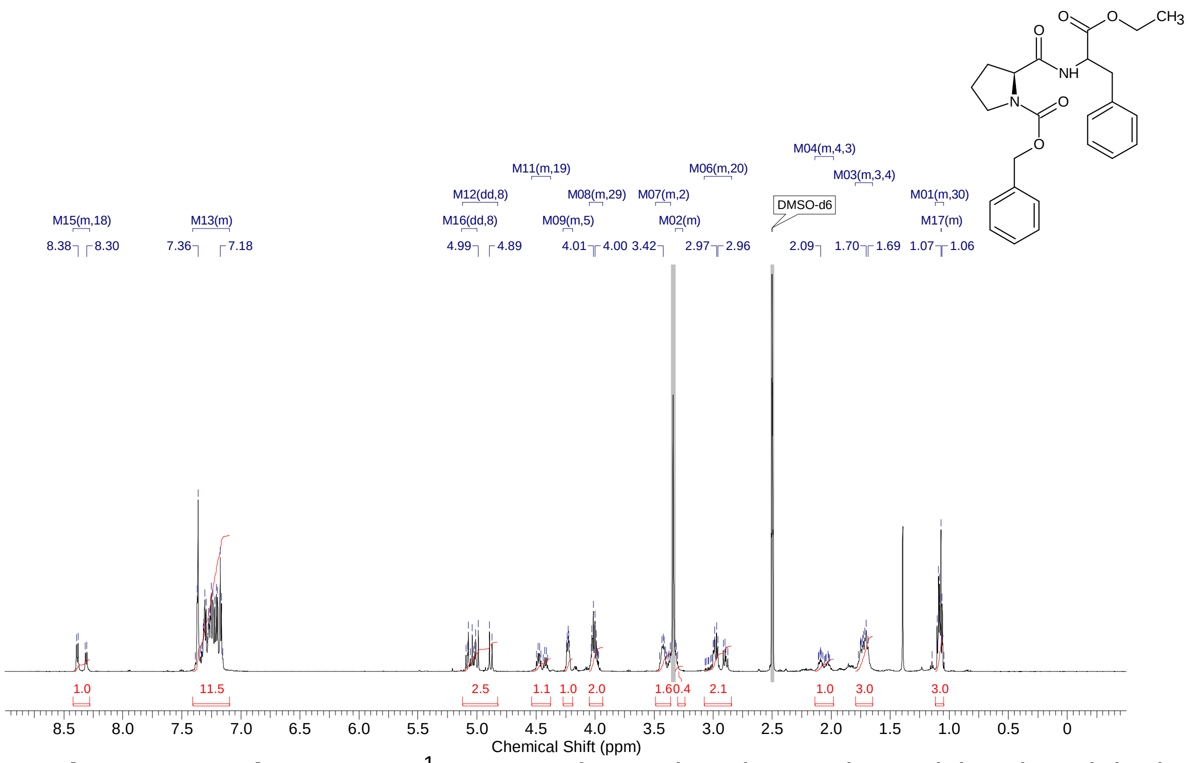

Supplementary Figure 100. ^{1}H NMR of *N*-carboxybenzoyl-L-prolyl-L-phenylalanine ethyl ester (**5adl**) (DMSO-d_6, 600 MHz).

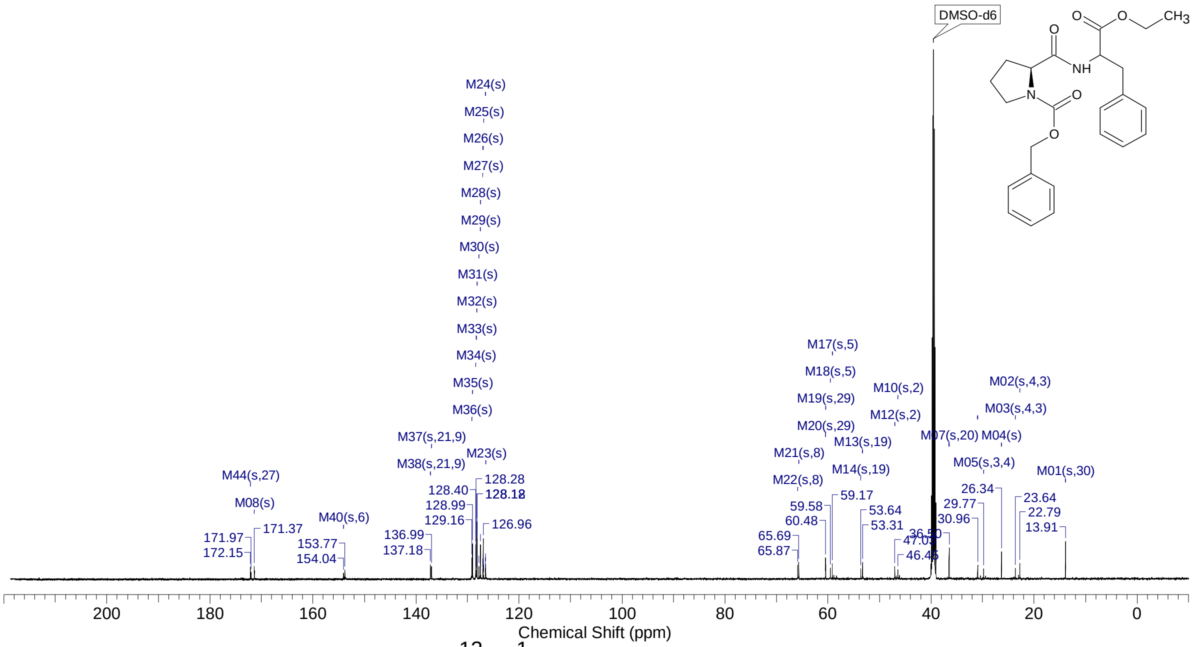

Supplementary Figure 101. ^{13}C{^{1}H} NMR of *N*-carboxybenzoyl-L-prolyl-L-phenylalanine ethyl ester (**5adl**) (DMSO-d_6. 400 MHz).

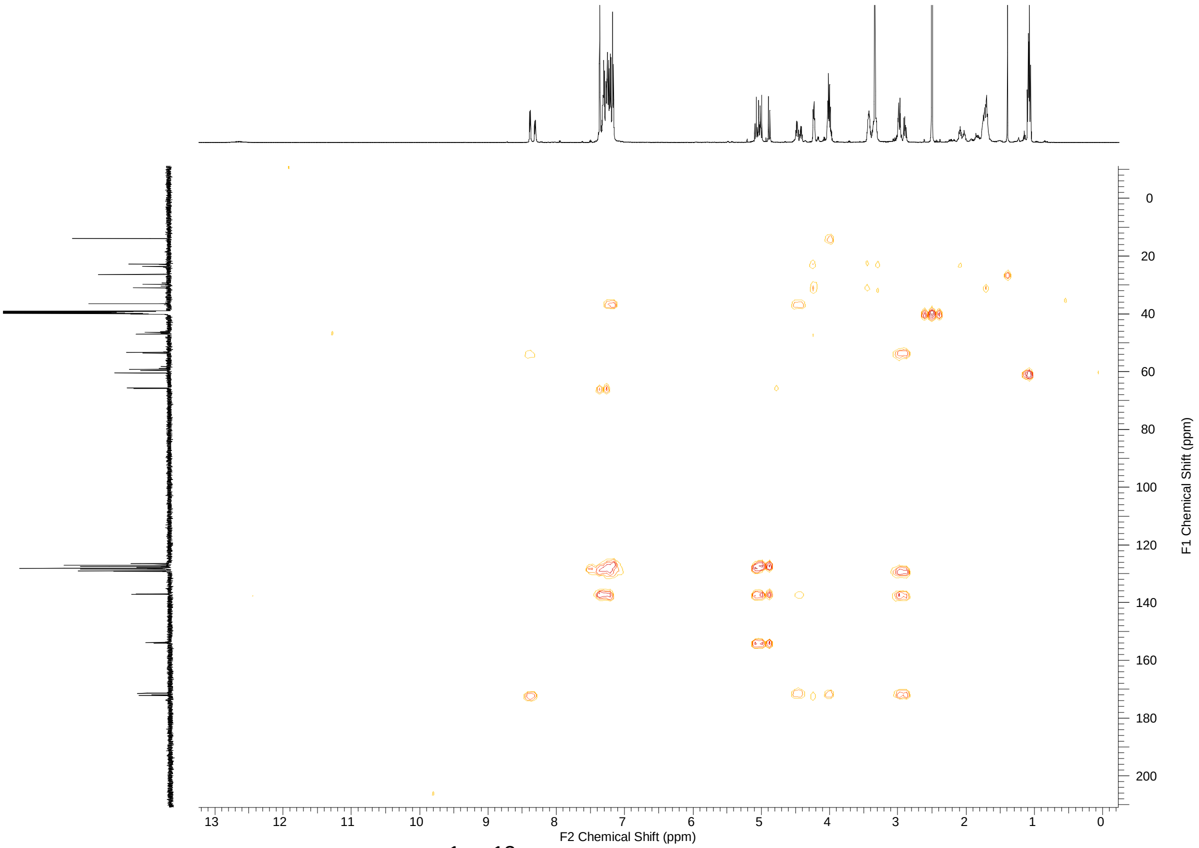

Supplementary Figure 102. $^{1}H/^{13}C$ HMBC NMR of *N*-carboxybenzoyl-L-prolyl-L-phenylalanine ethyl ester (**5adl**) (DMSO-d_6, 600 MHz).

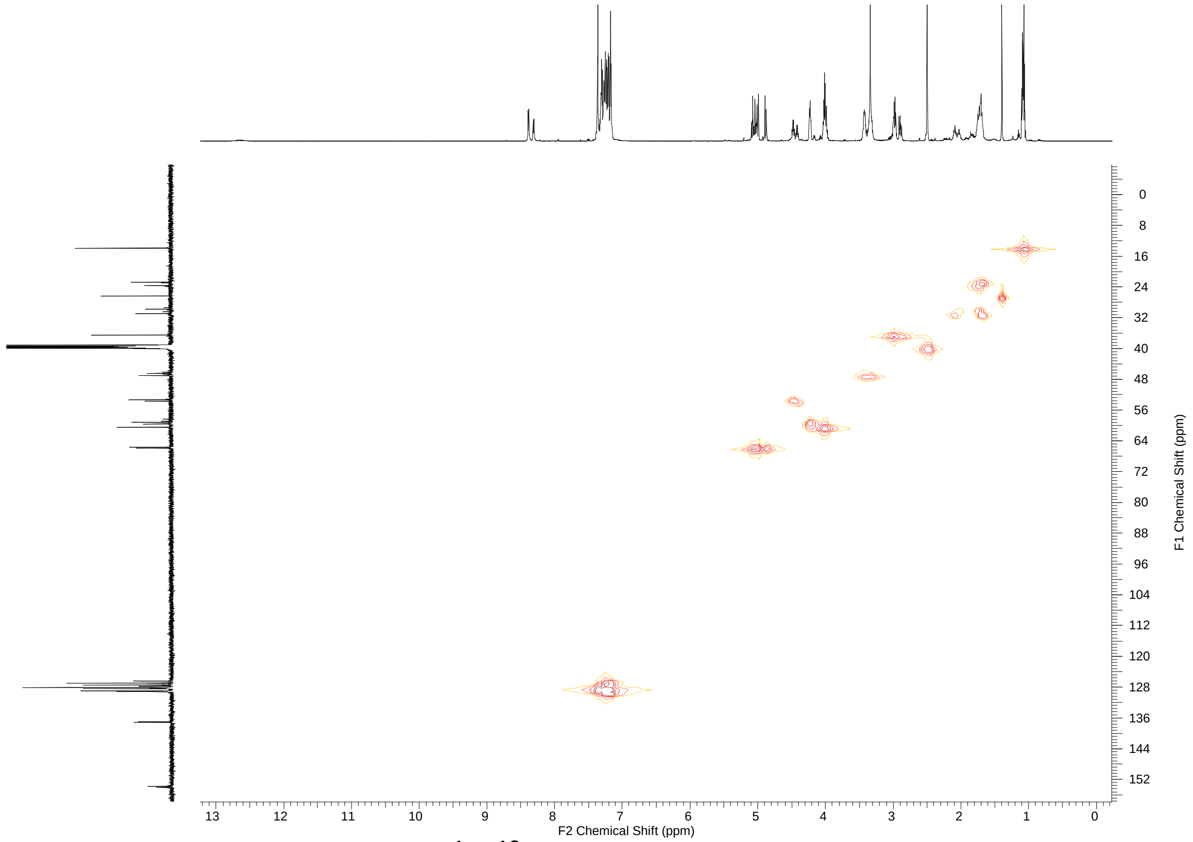

Supplementary Figure 103. $^{1}H/^{13}C$ HSQC NMR of *N*-carboxybenzoyl-L-prolyl-L-phenylalanine ethyl ester (**5adl**) (DMSO-d_6, 600 MHz).

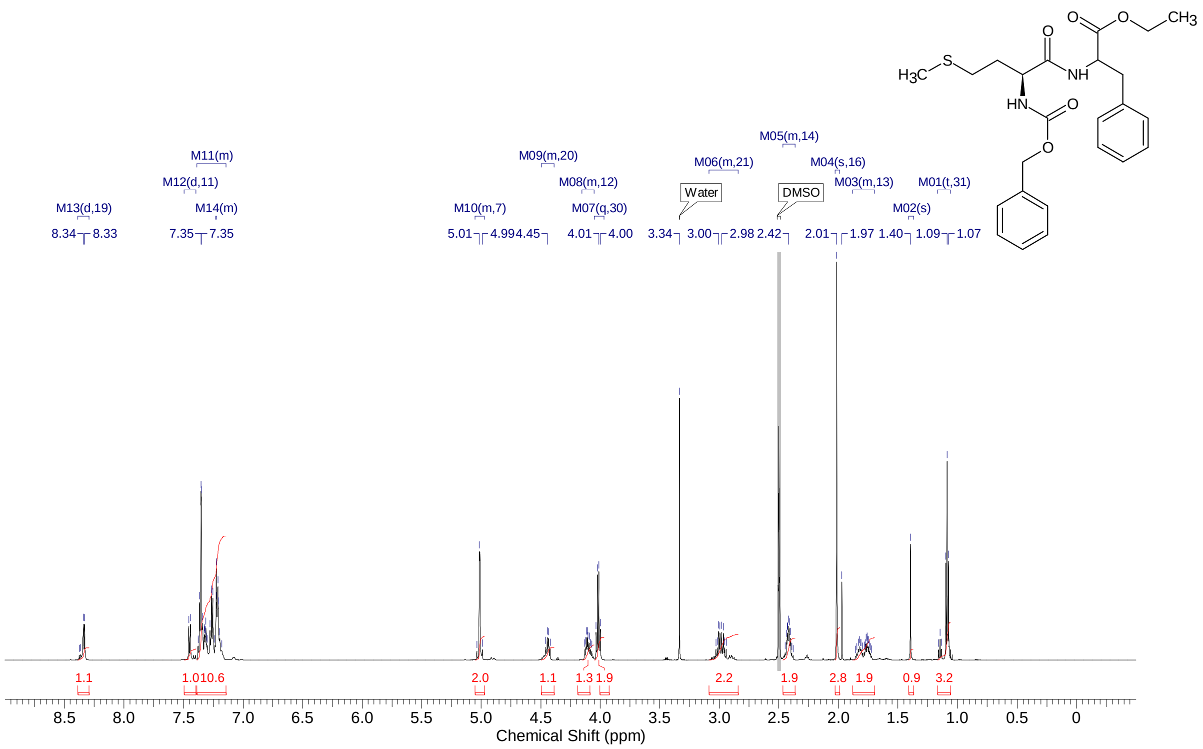

Supplementary Figure 104. ^{1}H NMR of *N*-(*N*-carboxybenzoyl-L-methionyl)-L-phenylalanine ethyl ester (**5ael**) (DMSO-d_6, 600 MHz).

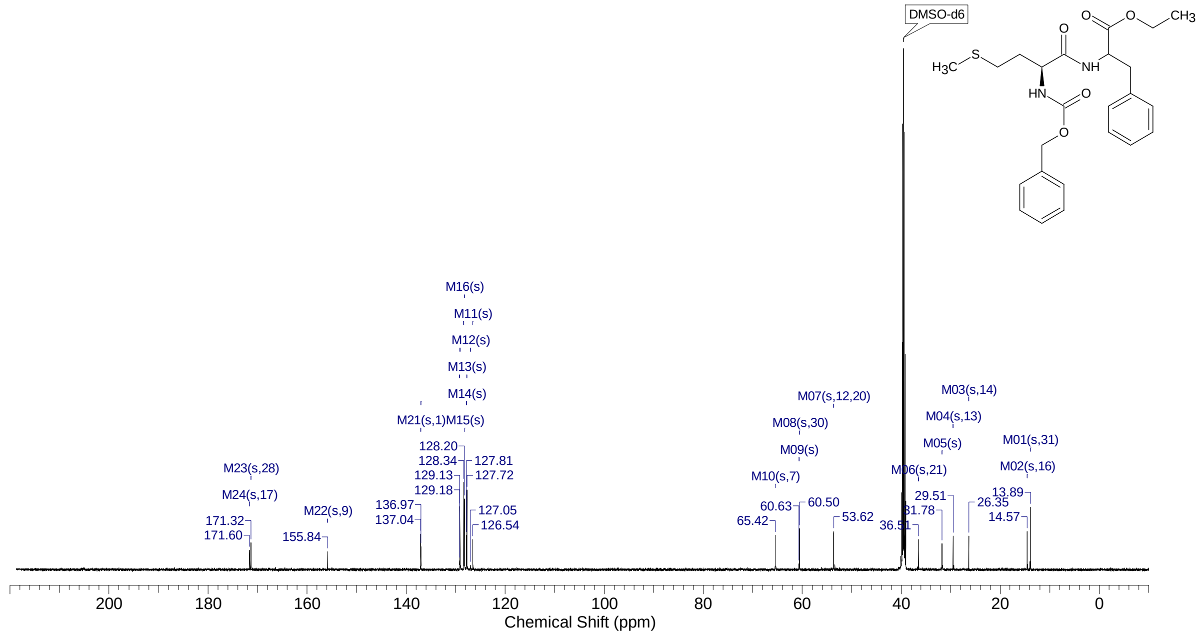

Supplementary Figure 105.$^{13}C\{^{1}H\}$ NMR of *N*-(*N*-carboxybenzoyl-L-methionyl)-L-phenylalanine ethyl ester (**5ael**) (DMSO-d_6. 400 MHz).

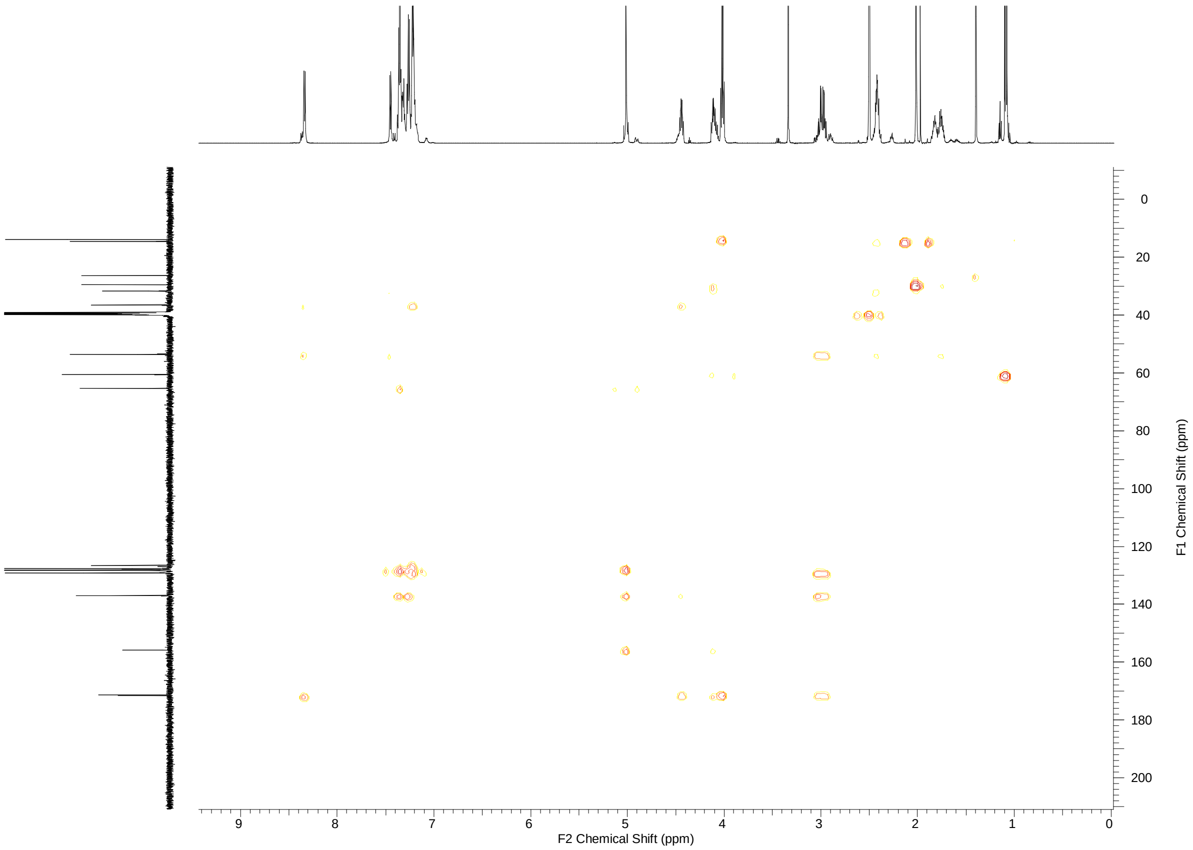

Supplementary Figure 106. $^{1}H/^{13}C$ HMBC NMR of *N*-(*N*-carboxybenzoyl-L-methionyl)-L-phenylalanine ethyl ester (**5ael**) (DMSO-d_6, 600 MHz).

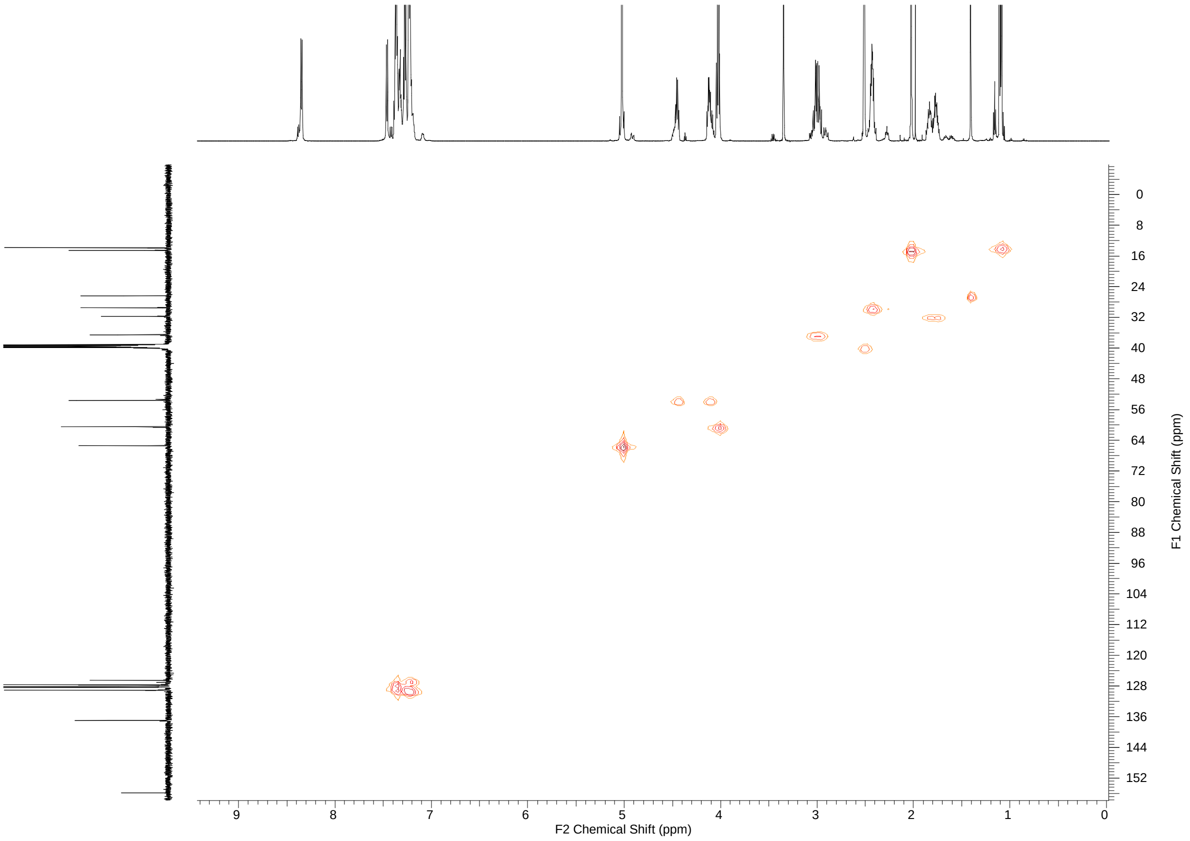

Supplementary Figure 107. $^{1}H/^{13}C$ HSQC NMR of *N*-(*N*-carboxybenzoyl-L-methionyl)-L-phenylalanine ethyl ester (**5ael**) (DMSO-d_6, 600 MHz).

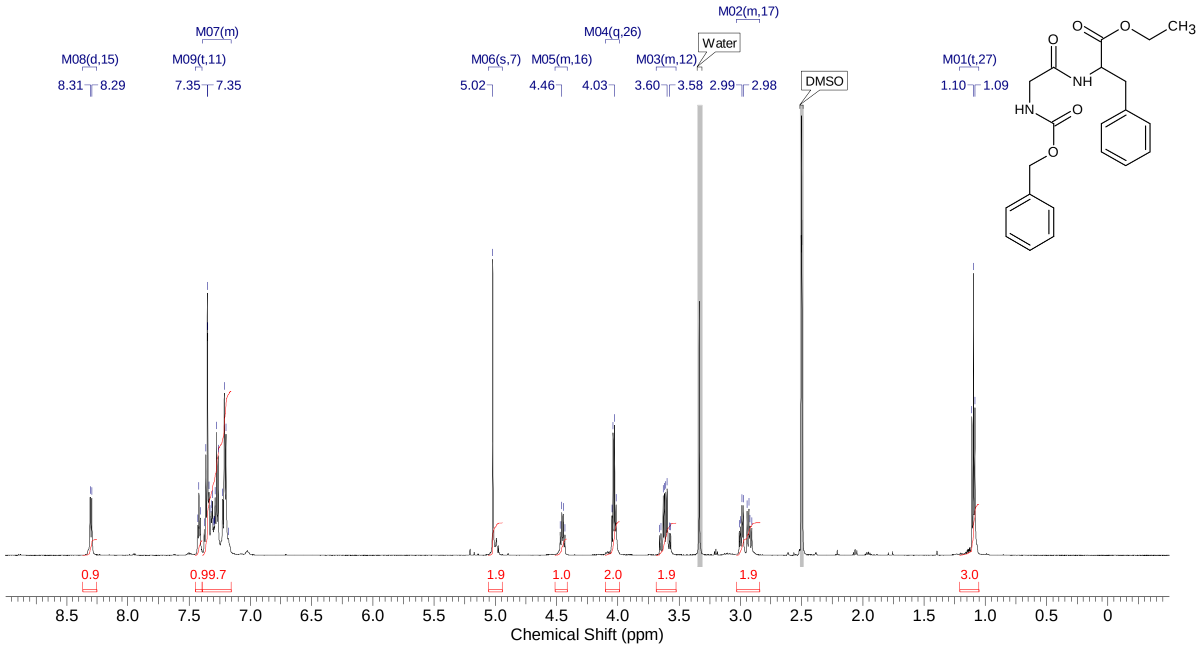

Supplementary Figure 108. ^{1}H NMR of *N*-(*N*-carboxybenzoyl-L-glycinyl)-L-phenylalanine ethyl ester (**5afl**) (DMSO-d_6, 600 MHz).

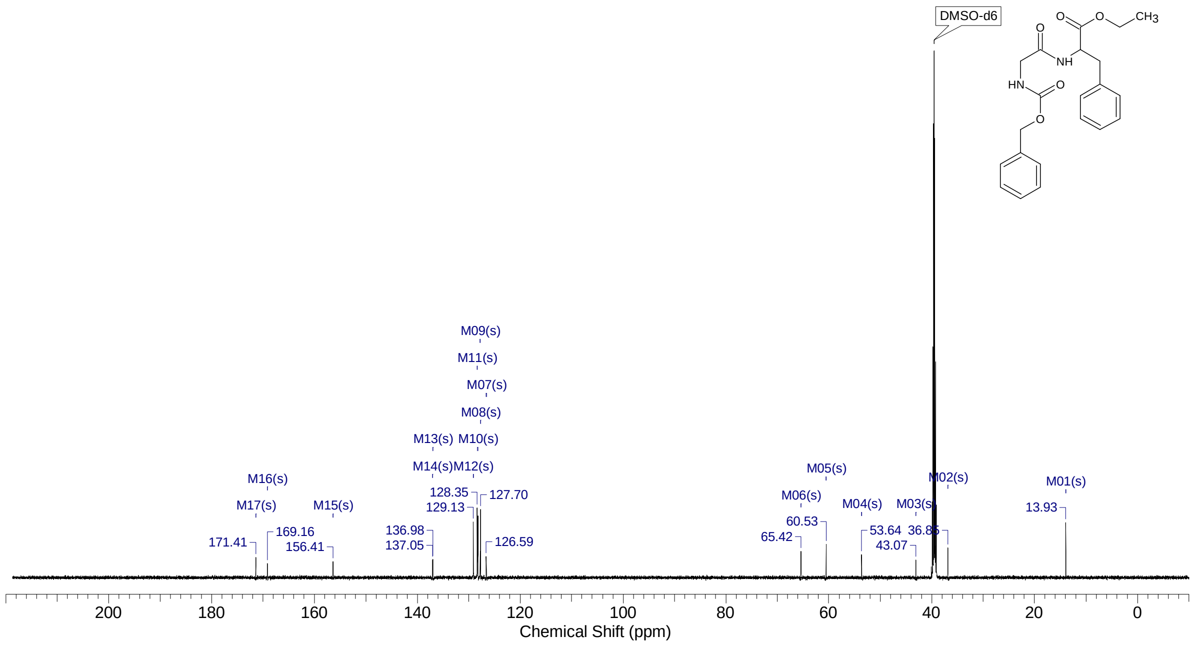

Supplementary Figure 109.^{13}C{^{1}H} NMR of *N*-(*N*-carboxybenzoyl-L-glycinyl)-L-phenylalanine ethyl ester (**5afl**) (DMSO-d_6. 400 MHz).

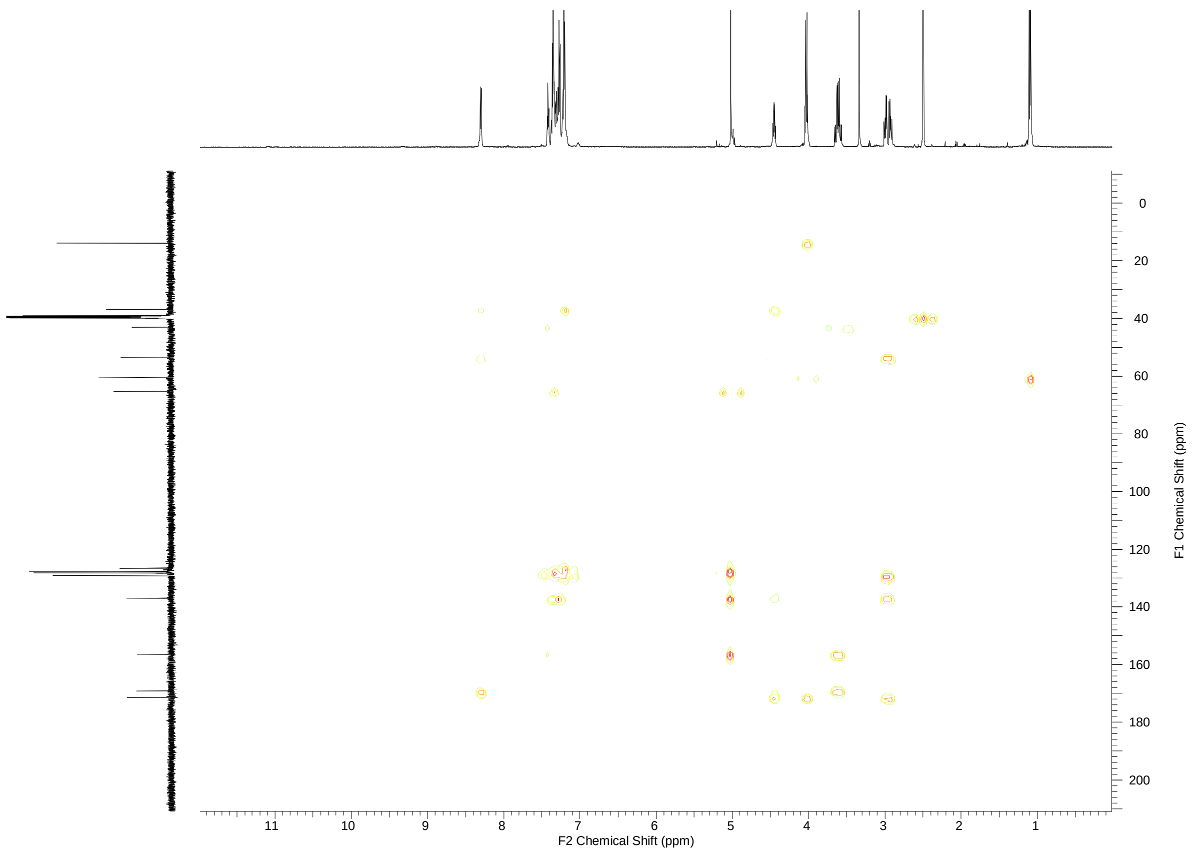

Supplementary Figure 110. $^{1}H/^{13}C$ HMBC NMR of *N*-(*N*-carboxybenzoyl-L-glycinyl)-L-phenylalanine ethyl ester (**5afl**) (DMSO-d_6, 600 MHz).

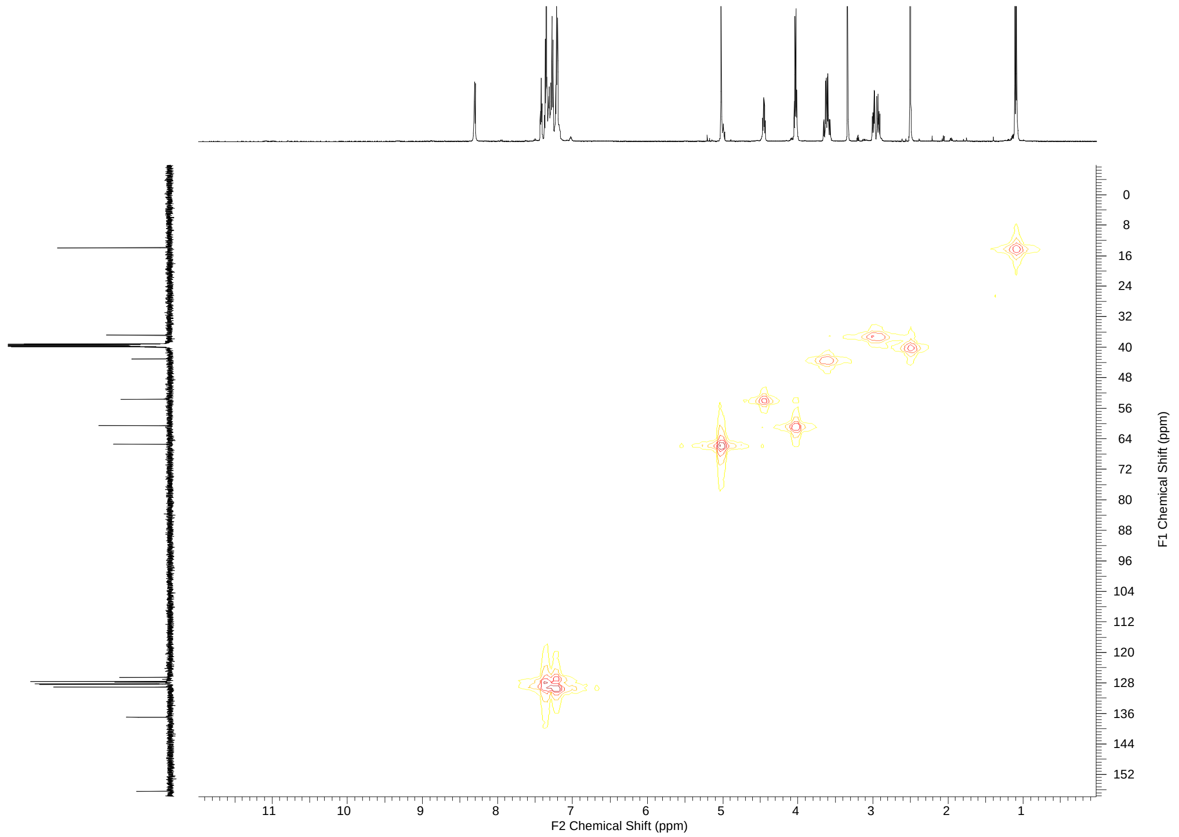

Supplementary Figure 111. $^{1}H/^{13}C$ HSQC NMR of *N*-(*N*-carboxybenzoyl-L-glycinyl)-L-phenylalanine ethyl ester (**5afl**) (DMSO-d_6, 600 MHz).

Supplementary Tables

Supplementary Table 1. Acetylene based optimization experiments[a]

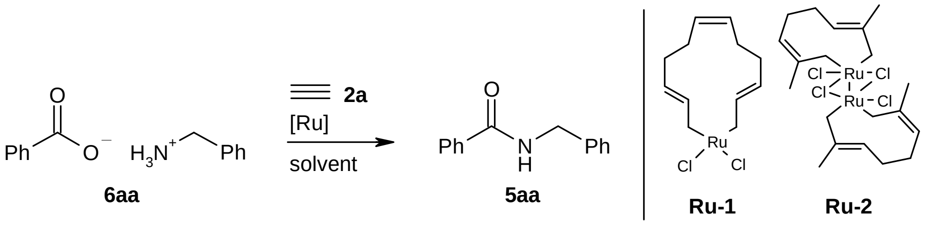

Entry	Solvent	[Ru] (mol%)	Changed Condition	Yield (%)
1	1,4-dioxane	$RuCl_3$•$3H_2O$ (1)	-	75
2	"	**Ru-2** (0.5)	-	73
3	"	**Ru-1** (1)	-	81
4	"	[{$RuCl_2$(*p*-cymene)}$_2$] (0.5)	Sequential addition of amine	trace[b]
5	"	"	Solvent exchange prior to amine addition	trace[c]
6	"	**Ru-1** (1)	Sequential addition of amine	trace[b]
7	"	"	Solvent exchange prior to amine addition	trace[c]
8	"	"	DMAP (1 mol%)	67
9	"	"	PPh_3 (1 mol%)	54
10	"	"	NEt_3 (1 mol%)	64
11	"	"	P(2-furyl)$_3$ (1 mol%)	42
12	"	RuO_2 (1)	-	0
13	"	[{$RuCl_2$(*p*-cymene)}$_2$] (0.5)	-	11
14	"	[$Ru_3(CO)_{12}$] (0.3)	-	trace
15	"	[$RuCl_2(PPh_3)_3$] (1)	-	24
16	"	[Ru(methallyl)$_2$(PPh_3)$_2$] (1)	-	32
17	"	[Ru(CO)HCl(PPh_3)$_3$] (1)	-	trace
18	"	[$AuClPPh_3$] (1)	-	0
19	"	$PdCl_2$ (1)	-	9
20	"	$FeCl_3$ (1)	-	0
21	ethyl acetate	**Ru-1** (1)	-	66
22	toluene	"	-	64
23	tetrahydrofuran	"	-	62
24	acetonitrile	"	-	25
25	water	"	-	0
26	NMP	"	-	25
27	1,4-dioxane/NMP 1:1	"	-	8
28	1,4-dioxane	"	+0.1 equiv water	79
29	"	"	+1 equiv. water	72
30	"	"	+10 equiv.water	15
31	"	"	no inert atmosphere was used	74
32	"	**Ru-1** (2)	-	94 (93)
33	"	$RuCl_3$•$3H_2O$ (2)	-	89
34	"	**Ru-1** (3)	-	84
35	"	**Ru-1** (5)	-	71
31	"	**Ru-1** (2)	0.4 ml solvent	90
32	"	"	0.2 ml solvent	77
33	"	"	0.1 ml solvent	78
34	"	"	neat	0
35	"	-	no catalyst	0
36	"	**Ru-1** (2)	1.5 equiv. 1-hexyne as alkyne	trace
37	"	"	1.5 equiv. TMS-acetylene as alkyne	trace
38	"	"	1.5 equiv. phenylacetylene as alkyne	trace
39	"	"	1.5 equiv. ethoxyacetylene as alkyne	87
40[d]	NMP	**Ru-1** (1.5)	1.5 equiv. ethoxyacetylene as alkyne	99 (99)

[a]Reaction conditions: 0.5 mL solvent, 0.5 mmol **6aa**, 0.25 mmol benzylamine, 1.7 atm. acetylene, [Ru], 80 °C, 6 h; [b]after the standard reaction time, amine was added and the reaction was stirred at 80 °C for 6h; [c]after the standard reaction time, the solvent was removed in vacuo, then amine in dichloromethane was added and the reaction was stirred at 80 °C for 6h; [d]4 h, 40 °C, 1 mL NMP. Yields were determined by GC analysis using *n*-tetradecane as internal standard; isolated yields in parenthesis.

Supplementary Table 2. Ethoxyacetylene based optimization experiments[a]

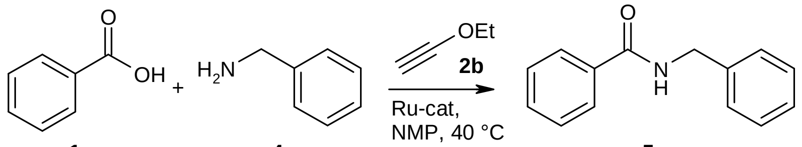

Entry	Solvent	[Ru] (mol%)	Changed Condition	Yield (%)
1	toluene	**Ru-1** (1)	PPh_3 (1mol%)	65
2	1,4-dioxane	"	"	68
3	ethyl acetate	"	"	52
4	NMP	"	"	71
5	"	**Ru-2** (0.5)	"	69
6	"	RuO_2 (1)	"	2
7	"	**Ru-1** (1)	P(*p*-tol)$_3$ (1mol%)	70
8	"	"	P(2-fur)$_3$ (1mol%)	54
9	"	"	P(OPh)$_3$(1mol%)	52
10	"	"	PCy_3(1mol%)	52
11	"	"	DMAP (1mol%)	81
12	"	"	2,2'-bipyridine (1mol%)	82
13	"	"	-	86
14	"	"	1 mmol **2b**	81
15	"	"	1 mmol **4a**	86
16	"	"	25 °C	44
17	"	"	60 °C	93
18	"	**Ru-1** (1.5)	-	99 (99)
19	"	$RuCl_3 \cdot 3H_2O$ (1.5)	-	33
20	toluene	**Ru-1** (1.5)	-	67
21	"	"	Amine added sequentially after 1h	65[b]
22	"	"	Amine added sequentially after solvent exchange	13[c]
23	ethyl acetate	"	-	75
24	"	"	Amine added sequentially after solvent exchange	17[c]
25	1,4-dioxane	"	-	77
26	"	"	Amine added sequentially after solvent exchange	21[c]
27	toluene	[RuCl$_2$(*p*-cymene)]$_2$ (0.75)	Amine added sequentially after 15min	78[b,d]
28	"	"	Amine added sequentially after solvent exchange	28[c,d]

[a]Reaction conditions: 1 mL solvent, 0.5 mmol **1a**, 0.75 mmol **4a**, 0.75 mmol **2b**, [Ru], 40 °C, 4 h; [b]the amine was added sequentially via syringe; [c]after 4 h the solvent was removed in vacuo and the amine in dichloromethane was added and the resulting mixture was stirred for another 4 h at 40 °C; [d]ethoxyacetylene in toluene was added dropwise at 0°C to a solution of **1a** and Ru-catalyst in toluene, then heating at 40°C for 1h[1]. Yields were determined by GC analysis using *n*-tetradecane as internal standard; isolated yields in parenthesis.

Supplementary Table 3. Optimization experiments for other amines[a]

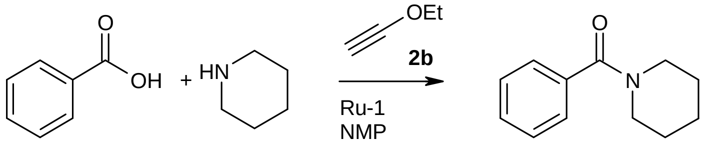

Entry	t (h)	T (°C)	Yield (%)
1	4	40	67
2[b]	"	40	62
3[c]	"	40	64
4	"	60	75
5	"	80	85
6	6	80	95 (92)
7	6	100	84
8	20	80	93

[a]Reaction conditions: 1 mL solvent, 0.5 mmol **1a**, 0.75 mmol **4i**, 0.75 mmol **2b**, 1.5mol% **Ru-1**; [b]1 mmol **2b**; [c]1 mmol **4i** Yields were determined by GC analysis using *n*-tetradecane as internal standard; isolated yields in parenthesis.

Supplementary Table 4. Catalyst screening for acetylene[a].

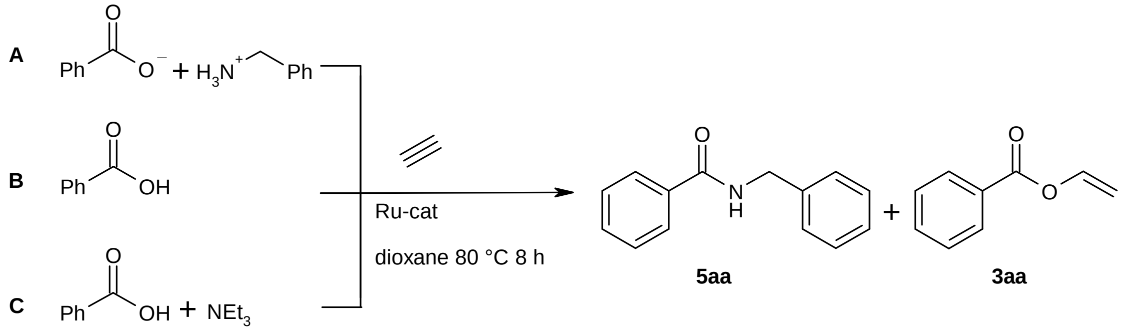

Entry	Starting Material	Ru-cat	Yield (%)	
			**5aa**	**3aa**
1	A	**Ru-1**	81	-
2		$RuCl_3$	75	-
3		**Ru-2**	73	
4		$[RuCl_2(p\text{-cymene})]_2$	11	-
5		$[Ru_3(CO)_{12}]$	1	-
6		$[RuCl_2(PPh_3)_3]$	24	-
7		$[Ru(methallyl)_2(PPh_3)_2]$	32	-
8		$[Ru(CO)HCl(PPh_3)_3]$	1	-
9		$[AuClPPh_3]$	0	-
10	B	**Ru-1**	-	1
11		$RuCl_3$	-	6
13		$[RuCl_2(p\text{-cymene})]_2$	-	16
14		$[Ru_3(CO)_{12}]$	-	52
15		$[RuCl_2(PPh_3)_3]$	-	0
18		$[Ru(methallyl)_2(PPh_3)_2]$	-	18
19	C	**Ru-1**	-	16
20		$RuCl_3$	-	14
21		$[RuCl_2(p\text{-cymene})]_2$	-	12
22		$[Ru_3(CO)_{12}]$	-	16
23		$[RuCl_2(PPh_3)_3]$	-	4
24		$[Ru(methallyl)_2(PPh_3)_2]$	-	7

[a]Reaction conditions: **A**: 0.5 mmol benzylammonium benzoate, 0.25 mmol benzylamine; **B**: 0.5 mmol benzoic acid; **C**: 0.5 mmol benzoic acid, 0.75 mmol triethylamine; **A,B,C**: 1.7 bar acetylene, 1mol% Ru-cat, 0.5 mL dioxane, 80 °C, 16 h; Yields were determined by GC analysis using *n*-tetradecane as internal standard.

Supplementary Table 5. Hexyne-based, state-of-the-art systems in presence of benzylamine[a]

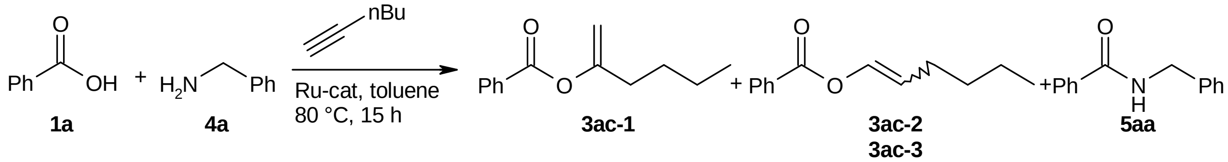

Entry	Ru-cat	Yield (%)	
		**3ac**	**5aa**
1	[Ru(methallyl)$_2$(dppb)]	0	4
2	[RuCl$_2$PPh$_3$(*p*-cymene)]	0	2
3	**Ru-1**	0	2

[a]Reaction conditions: 0.5 mmol **1a**, 0.75 mmol **4a**, 1 mol% Ru-cat, 0.5 mL toluene, 80 °C, 16 h. Yields were determined by GC analysis using *n*-tetradecane as internal standard.

Supplementary Table 6. Time dependent consumption of enol ester 3ab and influence of Ru-1 catalyst onto it[a]. An oven-dried headspace vial with Teflon-coated stirring bar was charged with 1-ethoxyvinyl benzoate[2] (96.1 mg, 0.5 mmol) or 1-hexen-2-ol benzoate[3] (102 mg, 0.5 mmol), sealed and the atmosphere was changed three times with nitrogen. Then *N*-methylpyrrolidone (1 ml), tetradecane (as internal standard for GC analysis; 50 µl, 0.193 mmol) and benzylamine (81.2 mg, 0.75 mmol, 82.8 µl) were added via syringe. Every 30 minutes, a 30 µl sample was taken, diluted with ethyl acetate (5 ml) and quenched with hydrochloric acid (0.25 M, 5 ml). The organic phase was filtered over a $MgSO_4$ pad and analyzed by GC analysis

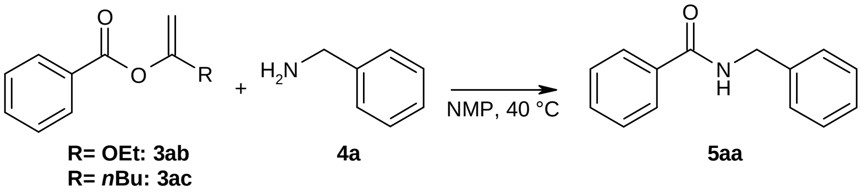

Entry	Reaction Time (min)	Yield (%)		
		With **Ru-1**[b]	Without **Ru-1**	Without **Ru-1**
		R= OEt	R= OEt	R= *n*Bu
1	30	83	91	<1
2	60	99	98	<1
3	90	98	97	<1
4	120	98	99	<1
5	960	-	-	3

[a]Reaction conditions: 0.5 mmol **3ab**, 0.75 mmol **4a**, 0.5 mL NMP, 40 °C. [b]1.5 mol% **Ru-1**. Yields were determined by GC analysis using *n*-tetradecane as internal standard.

Supplementary Methods.

General procedure A: acetylene mediated amidation.

An oven-dried headspace vial with Teflon-coated stirring bar was charged with the corresponding ammonium carboxylate (1 mmol) and dichloro[(2,6,10-dodecatriene)-1,12-diyl]ruthenium (5.06 mg, 20 µmol). The atmosphere was changed three times with nitrogen, then *N*-methylpyrrolidone (1 ml) and the corresponding amine (0.5 mmol) were added. The vial was placed in an autoclave reactor, the atmosphere was changed twice with acetylene, and a pressure of 1.7 bar was set. The mixture was then heated to 80 °C for 6 hours. After cooling down to r.t., the mixture was diluted with 20 mL of ethyl acetate and washed with each 20 mL of sat. $NaHCO_3$ solution, water and brine. The organic layer was dried with $MgSO_4$, the solvent removed under reduced pressure and the residue purified by column chromatography (SiOH, ethyl acetate/cyclohexane gradient).

General procedure B: ethoxyacetylene mediated amidation of various carboxylic acids.

An oven-dried headspace vial with Teflon-coated stirring bar was charged with the corresponding carboxylic acid (1 mmol) and dichloro[(2,6,10-dodecatriene)-1,12-diyl]ruthenium (5.06 mg, 15.0 µmol). The atmosphere was changed three times with nitrogen, then *N*-methylpyrrolidone (2 ml), benzylamine (164 mg, 167 µl, 1.5 mmol) and ethoxyacetylene (40 wt.%-solution in hexane) (210 mg, 299 µl, 1.5 mmol) were added in this order. The mixture was then heated to 40 °C for 4 hours. After cooling down to r.t., the mixture was diluted with 20 mL of ethyl acetate and washed with each 20 mL of sat. $NaHCO_3$ solution, water and brine. The organic layer was dried with $MgSO_4$, the solvent removed under reduced pressure and the residue purified by column chromatography (SiOH, ethyl acetate/cyclohexane gradient).

General procedure C: ethoxyacetylene mediated amidation of various amines.

An oven-dried headspace vial with Teflon-coated stirring bar was charged with benzoic acid (123 mg, 1 mmol) and dichloro[(2,6,10-dodecatriene)-1,12-diyl]ruthenium (5.06 mg, 15.0 µmol). The atmosphere was changed three times with nitrogen, then *N*-methylpyrrolidone (2 ml), the corresponding amine (1.5 mmol) and ethoxyacetylene (40 wt.%-solution in hexane) (210 mg, 299 µl, 1.5 mmol) were

added in this order. The mixture was then heated to 80 °C for 6 hours. After cooling down to r.t., the mixture was diluted with 20 mL of ethyl acetate and washed with each 20 mL of sat. $NaHCO_3$ solution, water and brine. The organic layer was dried with $MgSO_4$, the solvent removed under reduced pressure and the residue purified by column chromatography (SiOH, ethyl acetate/cyclohexane gradient).

General procedure D: ethoxyacetylene mediated dipeptide synthesis.

An oven-dried headspace vial with Teflon-coated stirring bar was charged with the corresponding *N*-protected amino acid (1 mmol), *C*-protected amino acid (1.5 mmol) and dichloro[(2,6,10-dodecatriene)-1,12-diyl]ruthenium (5.06 mg, 15.0 µmol). The atmosphere was changed three times with nitrogen, then toluene (2 ml), trimethylamine (304 mg, 415 µl, 3 mmol) and ethoxyacetylene (40 wt.%-solution in hexane) (210 mg, 299 µl, 1.5 mmol) were added in this order. The mixture was then heated to 80 °C for 6 hours. After cooling down to r.t., the mixture was diluted with 20 mL of ethyl acetate and washed with each 20 mL of sat. $NaHCO_3$ solution, water and brine. The organic layer was dried with $MgSO_4$, the solvent removed under reduced pressure and the residue purified by column chromatography (SiOH, ethyl acetate /cyclohexane gradient).

Synthesis and characterization of the corresponding products.

Synthesis of 1-ethoxyvinyl benzoate (3ab) [CAS: 38425-59-1]. Compound **3ab** was prepared following the literature procedure[4]. After purification **3ab** was isolated as a colorless oil (577 mg, 30 %). ^{1}H-NMR (400 MHz, Tol-*d*$_8$) δ = 7.99-8.04 (m, 2 H), 6.93-7.13 (m, 5 H), 3.97 (d, *J*=3.5 Hz, 1 H), 3.67 (d, *J*=3.3 Hz, 1 H), 3.50 (q, *J*=7.0 Hz, 2 H), 1.01 (t, *J*=7.0 Hz, 3 H) ppm; ^{13}C-NMR (101 MHz, Tol-*d*$_8$) δ = 163.5, 158.4, 137.5, 133.3, 130.4, 129.7, 128.5, 71.9, 64.8, 14.1 ppm. The analytical data matched those reported in the literature for 1-ethoxyvinyl benzoate[4].

Synthesis of *N*-benzylbenzamide (5aa) [CAS: 1485-70-7]. Compound **5aa** was prepared following the general procedure A or general procedure B, starting from benzoic acid (**1a**) (122 mg, 1 mmol). After purification **5aa** was isolated as a white solid (general procedure A: 181 mg, 86 %; general procedure B: 211 mg, 99 %). ^{1}H-NMR (400 MHz, $CDCl_3$) δ = 7.73-7.75 (m, 1 H), 7.71-7.73 (m, 1 H), 7.40-7.45 (m, 1

H), 7.31-7.37 (m, 2 H), 7.19-7.29 (m, 5 H), 6.59 (br. s., 1 H), 4.56 (d, *J* = 5.8 Hz, 2 H) ppm; ^{13}C-NMR (101 MHz, $CDCl_3$) δ = 167.4, 138.2, 134.3, 131.5, 128.7, 128.5, 127.8, 127.5, 126.9, 44.0 ppm; IR (ATR): $\tilde{\nu}$ = 3279 (w), 3062 (w), 3030 (w), 1738 (w), 1637 (m), 1601 (w), 1546 (s), 1490 (m), 1452 (m), 1417 (m), 1363 (m), 1315 (m), 1261 (m), 1217 (w), 1151 (w), 1075 (w), 1055 (w), 1027 (w), 986 (w), 929 (w), 905 (w), 805 (w), 726 (m), 692 (vs) cm^{-1}; MS (Ion trap, EI): m/z (%) = 211 (25), 210 (100), 105 (71), 77 (47), 51 (19), 50 (14), 44 (7); CHN elemental analysis calcd. for $C_{14}H_{13}NO$: C, 79.59; H, 6.20; N, 6.63; found: C, 79.49; H, 6.21; N, 6.66; melting point: 105.5 °C. The analytical data matched those reported in the literature for *N*-benzylbenzamide[5].

Synthesis of *N*-benzyl-4-methoxybenzamide (5ba) [CAS: 7465-87-4]. Compound **5ba** was prepared following the general procedure A or general procedure B, starting from 4-methoxybenzoic acid (**1b**) (152 mg, 1 mmol). After purification **5ba** was isolated as a light brown solid (general procedure A: 180 mg, 75 %; general procedure B: 201 mg, 83 %). ^{1}H-NMR (400 MHz, $CDCl_3$) δ = 7.66-7.73 (m, 2 H), 7.18-7.31 (m, 5 H), 6.79-6.90 (m, 2 H), 6.49 (br. s., 1 H), 4.55 (d, *J* = 5.8 Hz, 2 H), 3.77 (s, 3 H) ppm; ^{13}C-NMR (101 MHz, $CDCl_3$) δ = 166.9, 162.1, 138.4, 128.7, 128.7, 127.8, 127.4, 126.6, 113.7, 55.3, 44.0 ppm; IR (ATR): $\tilde{\nu}$ = 3252 (w), 3058 (w), 3023 (w), 2956 (w), 2931 (w), 2837 (w), 1738 (w), 1631 (m), 1605 (m), 1560 (m), 1507 (s), 1450 (m), 1420 (m), 1364 (m), 1326 (m), 1249 (s), 1177 (s), 1120 (w), 1054 (w), 1029 (m), 990 (m), 842 (s), 722 (vs), 686 (vs) cm^{-1}; MS (Ion trap, EI): m/z (%) = 241 (100), 135 (10), 134 (91), 106 (18), 91 (10), 76 (35), 43 (72); CHN elemental analysis calcd. for $C_{15}H_{15}NO_2$: C, 74.67; H, 6.27; N, 5.80. found: C, 74.70; H, 6.32; N, 5.79. melting point: 130.7 °C. The analytical data matched those reported in the literature for *N*-benzyl-4-methoxybenzamide[6].

Synthesis of *N*-benzyl-cyclohexanecarboxamide (5ca) [CAS: 35665-26-0]. Compound **5ca** was prepared following the general procedure A or general procedure B, starting from cyclohexanecarboxylic acid (**1c**) (128 mg, 1 mmol). After purification **5ca** was isolated as a white solid (general procedure A: 193 mg, 89 %; general procedure B: 206 mg, 95 %). ^{1}H-NMR (400 MHz, $CDCl_3$) δ= 7.16-7.33 (m, 5 H), 5.74 (br. s., 1 H), 4.37 (d, *J* = 5.5 Hz, 2 H), 2.06 (tt, *J* = 11.8, 3.5 Hz, 1 H), 1.83 (dd, *J* = 12.5, 2.5 Hz, 2 H), 1.67-1.78 (m, 2 H), 1.57-1.67 (m, 1 H), 1.41 (qd, *J* = 12.2, 2.8 Hz, 2 H), 1.11-1.28 (m, 3 H) ppm; ^{13}C-NMR (101 MHz, $CDCl_3$) δ = 175.9, 138.5,

128.6, 127.7, 127.4, 45.5, 43.3, 29.7, 25.7 (s, 2C) ppm; IR (ATR): $\tilde{\nu}$ = 3280 (w), 3085 (w), 3030 (w), 2924 (w), 2850 (w), 1738 (w), 1641 (m), 1619 (m), 1551 (m), 1489 (m), 1446 (m), 1377 (m), 1327 (w), 1257 (m), 1218 (s), 1104 (w), 1081 (w), 1029 (m), 972 (w), 926 (w), 897 (w), 848 (w), 810 (m), 742 (m), 693 (vs) cm^{-1}; MS (Ion trap, EI): m/z (%) = 218.0 (48), 217.0 (93), 202.1 (25), 162.0 (98), 106.0 (30), 91.1 (100), 55.0 (45); CHN elemental analysis calcd. for $C_{14}H_{19}NO$: N, 6.45; C, 77.38; H, 8.81; found N, 6.24; C, 76.98; H, 8.87; melting point: 111.0 °C. The analytical data matched those reported in the literature *N*-benzyl-cyclohexanecarboxamide[7].

Synthesis of thiophene-2-carboxylic acid benzylamide (5da) [CAS: 10354-43-5]. Compound **5da** was prepared following the general procedure A or general procedure B, starting from 2-thiophenecarboxylic acid (**1d**) (129 mg, 1 mmol). After purification **5da** was isolated as a white solid (general procedure A: 96 mg, 44 %; general procedure B: 117.0 mg, 99 %). ^{1}H-NMR (400 MHz, $CDCl_3$) δ = 7.46-7.56 (m, 2 H), 7.28-7.40 (m, 5 H), 7.05-7.11 (m, 1 H), 6.32 (br. s., 1 H), 4.60-4.67 (m, 2 H) ppm; ^{13}C-NMR (101 MHz, $CDCl_3$) δ = 161.7, 138.7, 138.0, 130.0, 128.8, 128.1, 127.9, 127.7, 127.6, 44.0 ppm; IR (ATR): $\tilde{\nu}$ = 3349 (m), 3089 (w), 3059 (w), 3029 (w), 2931 (w), 1738 (w), 1619 (m), 1542 (vs), 1510 (m), 1454 (w), 1421 (s), 1354 (m), 1300 (s), 1246 (m), 1143 (w), 1078 (w), 1023 (w), 962 (w), 861 (m), 715 (vs), 650 cm-1; MS (Ion trap, EI): m/z (%) = 218.0 (41), 217.0 (100), 111.0 (95), 106.2 (41), 91.1 (17), 77.1 (14), 51.0 (10); CHN elemental analysis calcd. for $C_{12}H_{11}NOS$: N, 6.45; C, 66.33; H, 5.10; S, 14.76; found N, 6.46; C, 66.14; H, 5.10; S,14.59; melting point: 120.5 °C. The analytical data matched those reported in the literature for thiophene-2-carboxylic acid benzylamide[6].

Synthesis of 3-methyl-*N*-benzylbutyramide (5ea) [CAS: 98379-78-3]. Compound **5ea** was prepared following the general procedure B, starting from 3-methylbutyric acid (**1e**) (102 mg, 1 mmol). After purification **5ea** was isolated as a white solid (191 mg, 99 %). ^{1}H-NMR (400 MHz, $CDCl_3$) δ = 7.16-7.33 (m, 5 H), 5.78 (br. s., 1 H), 4.38 (d, J = 5.8 Hz, 2 H), 1.96-2.19 (m, 3 H), 0.90 (d, J = 6.5 Hz, 6 H) ppm; ^{13}C-NMR (101 MHz, $CDCl_3$) δ = 172.3, 138.4, 128.7, 127.8, 127.4, 46.1, 43.5, 26.1, 22.5 ppm; IR (ATR): $\tilde{\nu}$ = 3067 (w), 2956 (w), 2868 (w), 1634 (m), 1544 (s), 1498 (w), 1453 (m), 1378 (w), 1352 (w), 1298 (w), 1251 (w), 1215 (w), 1169 (w), 1131 (w), 1064 (w), 1032 (w), 887 (w), 733 (s), 694 (vs) cm^{-1}; MS (Ion trap, EI): m/z (%) = 192.2 (100), 191.2

(30), 149.2 (28), 148.2 (14), 107.2 (15), 106.3 (30), 91.3 (20); HRMS-EI (TOF) calcd. for $C_{12}H_{17}NO$: 191.1310; found: 191.1301. The analytical data matched those reported in the literature 3-methyl-*N*-benzylbutyramide[8].

Synthesis of *N*-benzyl-2,2-dimethylpropanamide (5fa) [CAS: 26209-45-0]. Compound **5fa** was prepared following the general procedure B, starting from pivalic acid (**1f**) (103 mg, 1 mmol). After purification **5fa** was isolated as a white solid (95.0 mg, 50 %). ^{1}H-NMR (400 MHz, $CDCl_3$) δ = 7.15-7.32 (m, 5 H), 5.87 (br. s., 1 H), 4.38 (d, *J* = 5.5 Hz, 2 H), 1.11-1.21 (m, 9 H) ppm; ^{13}C-NMR (101 MHz, $CDCl_3$) δ = 178.3, 138.6, 128.7, 127.6, 127.4, 43.6, 38.7, 27.6 ppm; IR (ATR): $\tilde{\nu}$ = 3297 (w), 2969 (w), 2923 (w), 1738 (w), 1633 (m), 1542 (s), 1480 (m), 1453 (m), 1428 (w), 1365 (m), 1313 (w), 1218 (s), 1068 (w), 1028 (w), 999 (m), 906 (w), 734 (vs), 692 (vs) cm^{-1}; MS (Ion trap, EI): m/z (%) = 192.1 (26), 191.1 (71), 149.1 (20), 91.1 (100), 65.1 (17), 57.0 (34), 41.1 (21); HRMS-EI (TOF) calcd. for $C_{12}H_{17}NO$: 191.1310; found: 191.1326 [M^+]; melting point: 91.6 °C. The analytical data matched those reported in the literature for *N*-benzyl-2,2-dimethylpropanamide[9].

Synthesis of *N*-benzyl-4-nitrobenzamide (5ga) [CAS: 2585-26-4]. Compound **5ga** was prepared following the general procedure B, starting from 4-nitrobenzoic acid (**1g**) (167 mg, 1 mmol). After purification **5ga** was isolated as a white solid (239 mg, 93 %). ^{1}H-NMR (400 MHz, $CDCl_3$) δ = 8.11-8.22 (m, 2 H), 7.77-7.90 (m, 2 H), 7.14-7.36 (m, 5 H), 6.75 (br. s., 1 H), 4.56 (d, *J* = 5.5 Hz, 2 H) ppm; ^{13}C-NMR (151 MHz, $CDCl_3$) δ = 165.4, 149.5, 139.8, 137.4, 128.9, 128.2, 127.9, 127.9, 123.7, 44.4 ppm; IR (ATR): $\tilde{\nu}$ = 3277 (w), 3033 (w), 1739 (w), 1629 (m), 1596 (m), 1534 (s), 1509 (vs), 1484 (m), 1453 (m), 1345 (vs), 1280 (m), 1218 (m), 1104 (w), 1060 (w), 1031 (w), 1011 (w), 871 (m), 852 (m), 796 (w), 752 (m), 725 (m), 697 (s), 661 (s) cm^{-1}; MS (Ion trap, EI): m/z (%) = 105 (34), 78 (17), 77 (100), 74 (8), 52 (9), 51 (40), 50 (42); CHN elemental analysis calcd. for $C_{14}H_{12}N_2O_3$: C, 65.62; H, 4.72; N, 10.93; found: C, 65.85; H, 4.97; N, 10.92; melting point: 142.3 °C. The analytical data matched those reported in the literature for *N*-benzyl-4-nitrobenzamide[10].

Synthesis of *N*-benzyl-4-chlorobenzamide (5ha) [CAS-Nr: 7461-34-9]. Compound **5ha** was prepared following the general procedure B, starting from 4-chlorobenzoic acid (**1h**) (158 mg, 1 mmol). After purification **5ha** was isolated as a light brown solid

(235 mg, 96 %). ^{1}H-NMR (400 MHz, $CDCl_3$) δ = 7.60-7.71 (m, 2 H), 7.17-7.38 (m, 7 H), 6.50 (br. s., 1 H), 4.54 (d, *J* = 5.8 Hz, 2 H) ppm; ^{13}C-NMR (101 MHz, $CDCl_3$) δ = 166.3, 137.9, 137.7, 132.7, 128.8, 128.4, 127.9, 127.7, 44.2 ppm; IR (ATR): $\tilde{\nu}$ = 3309 (w), 3029 (w), 1739 (w), 1638 (s), 1593 (m), 1546 (s), 1486 (m), 1450 (m), 1421 (m), 1361 (w), 1318 (m), 1278 (m), 1256 (m), 1232 (w), 1152 (w), 1091 (m), 1054 (w), 1013 (m), 990 (w), 903 (w), 848 (s), 761 (m), 736 (m), 711 (vs), 669 (s) cm^{-1}; MS (Ion trap, EI): m/z (%) = 246 (64), 245 (58), 244 (42), 140 (35), 139 (100), 110 (37), 44 (28); CHN elemental analysis calcd. for $C_{14}H_{12}NOCl$: C, 68.44; H, 4.92; N, 5.70; found: C, 68.64; H, 5.09; N, 5.80; melting point: 164.8 °C. The analytical data matched those reported in the literature for *N*-benzyl-4-chloro-benzamide[11].

Synthesis of *N*-benzyl-4-cyanobenzamide (5ia) [CAS: 17922-99-5]. Compound **5ia** was prepared following the general procedure B, starting from 4-cyanobenzoic acid (**1i**) (149 mg, 1 mmol). After purification **5ia** was isolated as a light brown solid (191 mg, 81 %). ^{1}H-NMR (400 MHz, $CDCl_3$) δ = 7.76-7.85 (m, 2 H), 7.58-7.66 (m, 2 H), 7.20-7.33 (m, 5 H), 6.79 (br. s., 1 H), 4.55 (d, *J* = 5.8 Hz, 2 H) ppm; ^{13}C-NMR (101 MHz, $CDCl_3$) δ = 165.6, 138.2, 137.5, 132.3, 128.8, 127.8, 127.8, 127.7, 117.9, 115.0, 44.2 ppm; IR (ATR): $\tilde{\nu}$ = 3311 (m), 3087 (w), 3029 (w), 2970 (w), 2233 (w), 1738 (m), 1643 (vs), 1550 (s), 1495 (m), 1450 (m), 1423 (m), 1360 (m), 1311 (m), 1286 (m), 1253 (w), 1217 (m), 1055 (w), 1019 (w), 990 (w), 864 (s), 770 (w), 720 (vs), 673 cm^{-1}; MS (Ion trap, EI): m/z (%) = 237 (18), 236 (100), 235 (49), 130 (49), 106 (23), 102 (34), 44 (26); CHN elemental analysis calcd. for $C_{15}H_{12}N_2O$: C, 76.25; H, 5.12; N, 11.71; found: C, 75.90; H, 5.33; N, 11.71; melting point: 151.4 °C. The analytical data matched those reported in the literature for *N*-benzyl-4-cyanobenzamide[6].

Synthesis of *N*-benzyl-2-methoxybenzamide (5ja) [CAS: 183198-63-2]. Compound **5ja** was prepared following the general procedure B, starting from 2-methoxybenzoic acid (**1j**) (155 mg, 1 mmol). After purification **5ja** was isolated as a brown oil (221 mg, 92 %). ^{1}H-NMR (400 MHz, $CDCl_3$) δ = 8.16 (dd, *J* = 7.8, 1.8 Hz, 1 H), 8.12 (br. s., 1 H), 7.36 (ddd, *J* = 8.3, 7.4, 1.9 Hz, 1 H), 7.22-7.30 (m, 4 H), 7.15-7.21 (m, 1 H), 6.97-7.02 (m, 1 H), 6.88 (d, *J* = 8.3 Hz, 1 H), 4.60 (d, *J* = 5.8 Hz, 2 H), 3.81 (s, 3 H) ppm; ^{13}C-NMR (101 MHz, $CDCl_3$) δ = 165.3, 157.4, 138.7, 132.8, 132.3, 128.6, 127.4, 127.2, 121.3, 121.3, 111.2, 55.9, 43.7 ppm; IR (ATR): $\tilde{\nu}$ = 3395 (w), 3028 (w), 2942 (w), 1738 (w), 1643 (vs), 1599 (m), 1525 (vs), 1482 (vs), 1454 (s), 1293 (s), 1235 (vs),

1181 (m), 1105 (m), 1019 (s), 753 (vs), 697 (vs) cm^{-1}; MS (Ion trap, EI): m/z (%) = 248 (32), 247 (26), 246 (100), 245 (30), 139 (50), 111 (14), 106 (14); CHN elemental analysis calcd. for $C_{15}H_{15}NO_2$: C, 74.67; H, 6.27; N, 5.80; found: C, 74.59; H, 6.54; N, 6.05;. The analytical data matched those reported in the literature for *N*-benzyl-2-methoxybenzamide[6].

Synthesis of *N*-benzyl-3-methoxybenzamide (5ka) [CAS: 82082-48-2]. Compound **5ka** was prepared following the general procedure B, starting from 3-methoxybenzoic acid (**1k**) (155 mg, 1 mmol). After purification **5ka** was isolated as a white solid (238 mg, 99 %). ^{1}H-NMR (400 MHz, $CDCl_3$) δ = 7.16-7.45 (m, 7 H), 6.96 (td, *J*=4.6, 2.8 Hz, 1 H), 6.58 (br. s., 1 H), 4.55 (d, *J*=5.8 Hz, 2 H), 3.75 (s, 3 H) ppm; ^{13}C-NMR (101 MHz, $CDCl_3$) δ = 167.2, 159.8, 138.1, 135.8, 129.5, 128.7, 127.8, 127.5, 118.7, 117.7, 112.3, 55.4, 44.1 ppm; IR (ATR): $\tilde{\nu}$ = 3305 (w), 3029 (w), 2938 (w), 2835 (w), 1738 (w), 1636 (m), 1581 (s), 1533 (s), 1485 (s), 1453 (m), 1427 (m), 1286 (s), 1238 (s), 1131 (w), 1038 (m), 995 (w), 875 (w), 777 (w), 750 (m), 725 (m), 689 (vs) cm^{-1}; MS (Ion trap, EI): m/z (%) = 242.0 (25), 241.0 (100), 135.8 (54), 134.9 (74), 108.0 (36), 107.0 (33), 77.1 (41); HRMS-EI (TOF) calcd. for $C_{15}H_{15}NO_2$: 241.1107; found 241.1103 [M^+]. The analytical data matched those reported in the literature *N*-benzyl-3-methoxybenzamide[12].

Synthesis of 3-dimethylamino-*N*-benzylbenzamide (5la) [CAS: 793730-46-8]. Compound **5la** was prepared following the general procedure B, starting from 3-(dimethylamino)benzoic acid (**1l**) (169 mg, 1 mmol). After purification **5la** was isolated as pale brown solid (222 mg, 87 %). ^{1}H-NMR (400 MHz, $CDCl_3$) δ = 7.22-7.32 (m, 7 H), 6.88-6.95 (m, 1 H), 6.76 (dd, *J* = 8.0, 2.5 Hz, 1 H), 6.40 (br. s., 1 H), 4.56 (d, *J* = 5.5 Hz, 2 H), 2.91 (s, 6 H) ppm; ^{13}C-NMR (101 MHz, $CDCl_3$) δ = 168.1, 150.7, 138.3, 135.2, 129.1, 128.7, 127.8, 127.5, 115.3, 113.9, 111.4, 44.0, 40.5 ppm; IR (ATR): $\tilde{\nu}$ = 3333 (w), 2919 (w), 2807 (w), 1637 (m), 1600 (m), 1572 (m), 1534 (vs), 1495 (vs), 1438 (m), 1364 (m), 1314 (m), 1291 (m), 1233 (m), 1187 (w), 1083 (w), 1063 (w), 1029 (w), 986 (w), 954 (w), 882 (w), 858 (w), 797 (w), 737 (vs), 689 (vs) cm^{-1}; MS (Ion trap, EI): m/z (%) = 254.2 (96), 149.0 (58), 148.2 (21), 121.2 (70), 120.2 (100), 91.1 (22), 77.1 (25); CHN elemental analysis calcd. for $C_{16}H_{18}N_2O$: N, 11.01; C, 75.56; H, 7.13; found N, 10.95; C, 75.57; H, 7.09; melting point: 135.8 °C. The

analytical data matched those reported in the literature for 3-dimethylamine-*N*-benzylbenzamide[13].

Synthesis of *N*-benzyl-4-bromobenzamide (5ma) [CAS: 80311-89-3]. Compound **5ma** was prepared following the general procedure B, starting from 4-bromobenzoic acid (**1m**) (205 mg, 1.0 mmol). After purification **5ma** was isolated as a white solid (270 mg, 93 %). ^{1}H-NMR (400 MHz, DMSO-d_6) δ = 9.18 (t, J = 5.9 Hz, 1 H), 7.85-7.94 (m, 2 H), 7.62-7.72 (m, 2 H), 7.20-7.38 (m, 5 H), 4.53 (d, J = 5.8 Hz, 2 H) ppm; ^{13}C-NMR (101 MHz, DMSO-d_6) δ = 165.3, 139.4, 133.5, 131.3, 129.4, 128.2, 127.2, 126.7, 125.0, 42.8 ppm; IR (ATR): $\tilde{\nu}$ = 3307 (w), 3085 (w), 3059 (w), 3028 (w), 1739 (m), 1637 (s), 1588 (m), 1546 (vs), 1482 (m), 1450 (m), 1420 (m), 1361 (m), 1319 (s), 1256 (m), 1217 (m), 1151 (w), 1071 (m), 1010 (m), 902 (w), 846 (s), 820 (w), 758 (m), 730 (vs), 700 (vs), 669 (vs) cm^{-1}; MS (Ion trap, EI): m/z (%) = 291.1 (100), 290.2 (53), 289.3 (82), 288.5 (27), 185.2 (50), 183.2 (45), 106.3 (27); CHN elemental analysis calcd. for $C_{14}H_{12}BrNO$: N, 4.83; C, 57.95; H, 4.71; found N, 4.83; C, 57.87; H, 4.46; melting point: 169.9 °C. The analytical data matched those reported in the literature for *N*-benzyl-4-bromobenzamide[10].

Synthesis of *N*-benzyl-terephthalamic acid methyl ester (5na) [CAS: 349491-81-2]. Compound **5na** was prepared following the general procedure B, starting from monomethyl terephthalate (**1n**) (180 mg, 1.0 mmol). After purification **5na** was isolated as a white solid (218 mg, 81 %). ^{1}H-NMR (400 MHz, $CDCl_3$) δ = 8.05-8.11 (m, 2 H), 7.82-7.87 (m, 2 H), 7.28-7.39 (m, 4 H), 6.60 (br. s., 1 H), 4.65 (d, J = 5.8 Hz, 2 H), 3.94 (s, 3 H) ppm; ^{13}C-NMR (101 MHz, $CDCl_3$) δ = 166.4, 166.3, 138.2, 137.8, 132.7, 129.8, 128.8, 128.0, 127.8, 127.0, 52.4, 44.3 ppm; IR (ATR): $\tilde{\nu}$ = 3298 (w), 2950 (w), 1713 (vs), 1640 (s), 1552 (s), 1495 (m), 1441 (m), 1362 (m), 1281 (vs), 1193 (m), 1151 (w), 1110 (s), 1054 (w), 1017 (m), 991 (w), 963 (w), 871 (m), 810 (w), 730 (m), 701 (vs), 666 (m) cm^{-1}; MS (Ion trap, EI): m/z (%) = 270.1 (17), 269.2 (100), 268.3 (38), 254.3 (13), 163.1 (58), 104.1 (15), 77.1 (15); CHN elemental analysis calcd. for $C_{16}H_{15}NO_3$: N, 5.20; C, 71.36; H, 5.61; found N, 5.08; C, 71.41; H, 5.58; melting point: 152.1°C. The analytical data matched those reported in the literature for *N*-benzyl-terephthalamic acid methyl ester[14].

Synthesis of *N*-benzyl-4-formylbenzamide (5oa) [CAS: 129242-08-6]. Compound **5oa** was prepared following the general procedure B, starting from 4-formylbenzoic acid (**1o**) (155 mg, 1.0 mmol) with 2.5 mmol of benzylamine. After purification **5oa** was isolated as a white solid (245 mg, 99 %). ^{1}H-NMR (400 MHz, $CDCl_3$) δ = 10.01 (d, *J* = 2.3 Hz, 1 H), 7.80-7.96 (m, 4 H), 7.23-7.37 (m, 5 H), 6.54 (br. s., 1 H), 4.54-4.66 (m, 2 H) ppm; ^{13}C-NMR (101 MHz, $CDCl_3$) δ = 191.5, 166.2, 139.5, 138.2, 137.7, 129.8, 128.9, 128.0, 127.8, 127.7, 44.3 ppm; IR (ATR): $\tilde{\nu}$ = 3308 (w), 3016 (w), 2970 (w), 2946 (w), 1739 (vs), 1638 (w), 1547 (w), 1454 (w), 1366 (s), 1217 (s), 990 (w), 847 (w), 801 (w), 758 (w), 727 (w), 696 (w), 667 (w) cm^{-1}; MS (Ion trap, EI): m/z (%) = 240.1 (19), 239.1 (100), 238.2 (38), 133.0 (63), 105.0 (25), 76.9 (34), 51.0 (18); HRMS-EI (TOF) calcd. for $C_{15}H_{13}NO_2$: 239.0946; found: 239.0945; melting point: 109.1 °C. The analytical data matched those reported in the literature for *N*-benzyl-4-formylbenzamide[15].

Synthesis of *N*-benzyl-4-(trifluoromethyl)benzamide (5pa) [CAS: 365274-70-0]. Compound **5pa** was prepared following the general procedure B, starting from 4-(trifluoromethyl)benzoic acid (**1p**) (194 mg, 1.0 mmol). After purification **5pa** was isolated as white solid (246 mg, 88 %). ^{1}H-NMR (400 MHz, $CDCl_3$) δ = 7.81 (m, *J* = 8.0 Hz, 2 H), 7.59 (m, *J* = 8.3 Hz, 2 H), 7.27 (s, 5 H), 6.56 (br. s., 1 H), 4.56 (d, *J* = 5.5 Hz, 2 H) ppm; ^{13}C-NMR (101 MHz, $CDCl_3$) δ = 166.1, 137.7, 137.6, 133.3 (q, *J* = 32.7 Hz), 128.9, 127.9, 127.8, 127.4, 125.6 (q, *J* = 3.6 Hz), 123.6 (q, *J* = 272.5 Hz), 44.3 ppm; IR (ATR): $\tilde{\nu}$ = 3323 (w), 3033 (w), 2970 (w), 1739 (m), 1642 (m), 1547 (s), 1497 (w), 1454 (w), 1421 (w), 1364 (m), 1309 (m), 1256 (m), 1230 (m), 1156 (s), 1121 (vs), 1068 (s), 1015 (m), 989 (m), 861 (s), 775 (m), 728 (s), 670 (vs) cm^{-1}; MS (Ion trap, EI): m/z (%) = 280.1 (22), 279.2 (100), 278.3 (42), 173.1 (61), 145.1 (27), 106.2 (15), 77.1 (12); CHN elemental analysis calcd. for $C_{15}H_{12}F_3NO$: N, 5.02; C, 64.51; H, 4.33; found N, 4.99; C, 64.40; H, 4,36; melting point: 170.8 °C. The analytical data matched those reported in the literature for *N*-benzyl-4-(trifluoromethyl)benzamide[16].

Synthesis of 6-oxo-6-(benzylamino)-hexanoic acid methyl ester (5qa) [CAS: 1094692-14-4]. Compound **5qa** was prepared following the general procedure B, starting from monomethyl adipate (**1q**) (163 mg, 1.0 mmol). After purification **5qa** was isolated as a white solid (245 mg, 98 %). ^{1}H-NMR (400 MHz, $CDCl_3$) δ = 7.24-7.38

(m, 5 H), 7.03 (t, *J* = 5.1 Hz, 1 H), 4.38 (d, *J* = 5.8 Hz, 2 H), 3.66 (s, 3 H), 2.31 (t, *J* = 7.0 Hz, 2 H), 2.22 (t, *J* = 7.0 Hz, 2 H), 1.59-1.70 (m, 4 H) ppm; ^{13}C-NMR (101 MHz, $CDCl_3$) δ = 173.6, 172.5, 138.3, 128.2, 127.2, 126.9, 51.2, 42.9, 35.6, 33.3, 24.8, 24.1 ppm; IR (ATR): $\tilde{\nu}$ = 3290 (m), 3067 (w), 2953 (w), 1729 (vs), 1634 (vs), 1546 (s), 1464 (m), 1436 (m), 1379 (m), 1357 (m), 1268 (m), 1231 (m), 1176 (s), 1082 (w), 1034 (w), 1003 (w), 978 (w), 908 (w), 884 (w), 734 (m), 691 (vs) cm^{-1}; MS (Ion trap, EI): m/z (%) = 250.0 (16), 218.1 (12), 161.0 (11), 160.0 (10), 107.1 (11), 106.0 (100), 91.1 (31); CHN elemental analysis calcd. for $C_{14}H_{19}NO_3$: N, 5.62; C, 67.45; H, 7.68; found N, 5.80; C, 67.31; H, 7.47; melting point: 47.1 °C. The analytical data matched those reported in the literature for 6-oxo-6-(benzylamino)-hexanoic acid methyl ester[17].

Synthesis of ethyl *N*-benzylmalonamate (5ra) [CAS: 29689-63-2]. Compound **5ra** was prepared following the general procedure B, starting from monoethylmalonate (**1r**) (136 mg, 1 mmol). After purification **5ra** was isolated as a white solid (92 mg, 42 %). ^{1}H-NMR (400 MHz, $CDCl_3$) δ = 7.37 (br. s., 1 H), 7.16-7.28 (m, 5 H), 4.40 (d, *J* = 5.6 Hz, 2 H), 4.10 (q, *J* = 7.2 Hz, 2 H), 3.27 (s, 2 H), 1.19 (t, *J* = 7.2 Hz, 3 H) ppm; ^{13}C-NMR (101 MHz, $CDCl_3$) δ = 169.5, 164.9, 137.8, 128.7, 127.7, 127.5, 61.6, 43.5, 41.0, 14.0 ppm; IR (ATR): $\tilde{\nu}$ = 3290 (w), 2970 (w), 1737 (vs), 1651 (vs), 1541 (m), 1455 (m), 1368 (m), 1334 (m), 1217 (m), 1188 (m), 1154 (s), 1029 (m), 847 (w), 732 (m), 698 (vs) cm^{-1}; MS (Ion trap, EI): m/z (%) = 221.0 (13), 118.1 (15), 107.1 (13), 106.0 (100), 91.1 (33), 79.1 (14), 77.1 (11); HRMS-EI (TOF) calcd. for $C_{12}H_{15}NO_3$: 221.1052; found 221.1054. The analytical data matched those reported in the literature for ethyl *N*-benzylmalonamate[18].

Synthesis of *N*-benzylpyridine-4-carboxamide (5sa) [CAS: 6320-63-4]. Compound **5sa** was prepared following the general procedure B, starting from isonicotinic acid (**1s**) (123 mg, 1 mmol). After purification **5sa** was isolated as a white solid (105 mg, 50 %). ^{1}H-NMR (400 MHz, $CDCl_3$) δ = 9.36 (br. s., 1 H), 8.67-8.82 (m, 2 H), 7.75-7.90 (m, 2 H), 7.30-7.43 (m, 4 H), 7.22-7.29 (m, 1 H), 4.51 (d, *J* = 6.0 Hz, 2 H) ppm; ^{13}C-NMR (101 MHz, $CDCl_3$) δ = 164.7, 150.3, 141.2, 139.1, 128.4, 127.3, 126.9, 121.3, 42.7 ppm; IR (ATR): $\tilde{\nu}$ = 3320 (w), 3029 (w), 1737 (w), 1645 (m), 1542 (s), 1489 (m), 1451 (m), 1422 (w), 1362 (w), 1315 (m), 1251 (m), 1217 (w), 1156 (w), 1066 (w), 1030 (w), 989 (w), 850 (m), 757 (m), 728 (s), 695 (s), 661 (vs) cm^{-1}; MS (Ion trap, EI):

m/z (%) = 212.2 (100), 211.2 (33), 106.0 (49), 91.1 (22), 79.0 (24), 78.0 (40), 51.0 (32); HRMS-EI (TOF) calcd. for $C_{13}H_{12}N_2O$: 212.0950; found: 212.0852 [M^+]; melting point: 91.6 °C. The analytical data matched those reported in the literature for *N*-benzylpyridine-4-carboxamide[19].

Synthesis of 3-(1,3-benzodioxol-5-yl)-*N*-benzyl-2-propenamide (5ta) [CAS: 73080-05-4]. Compound **5ta** was prepared following the general procedure B, starting from (E)-3-(benzo[1,3]dioxol-5-yl)acrylic acid (**1t**) (194 mg, 1 mmol). After purification **5ta** was isolated as a white solid (221 mg, 79 %). ^{1}H-NMR (400 MHz, $CDCl_3$) δ = 7.53 (d, *J* = 15.6 Hz, 1 H), 7.20-7.32 (m, 5 H), 6.89-6.94 (m, 2 H), 6.72-6.76 (m, 1 H), 6.19 (d, *J* = 15.6 Hz, 1 H), 5.93 (s, 2 H), 5.86 (br. s., 1 H), 4.51 (d, *J* = 5.8 Hz, 2 H) ppm; ^{13}C-NMR (101 MHz, $CDCl_3$) δ = 165.9, 149.1, 148.2, 141.1, 138.2, 129.1, 128.7, 127.9, 127.6, 123.9, 118.4, 108.5, 106.3, 101.4, 43.8 ppm; IR (ATR): $\tilde{\nu}$ = 3279 (w), 3082 (w), 2970 (w), 2897 (w), 2784 (w), 1738 (m), 1655 (m), 1619 (s), 1560 (m), 1488 (s), 1445 (vs), 1375 (m), 1327 (m), 1249 (vs), 1218 (vs), 1103 (m), 1037 (s), 972 (s), 926 (m), 848 (m), 810 (s), 741 (vs), 681 (m) cm^{-1}; MS (Ion trap, EI): m/z (%) = 281.2 (24), 214.3 (20), 147.1 (17), 146.1 (69), 91.1 (100), 73.2 (31); CHN elemental analysis calcd. for $C_{17}H_{15}NO_3$: N, 4.98; C, 72.58; H, 5.37; found N, 4.89; C, 72.38; H, 5.58; melting point: 136.6 °C. The analytical data matched those reported in the literature 3-(1,3-benzodioxol-5-yl)-*N*-benzyl-2-propenamide[20].

Synthesis of *N*-benzyl-4-phenylbutanamide (5ua) [CAS: 179923-27-4]. Compound **5ua** was prepared following the general procedure B, starting from 4-phenylbutyric acid (**1u**) (166 mg, 1 mmol). After purification **5ua** was isolated as a white solid (253 mg, 99 %). ^{1}H-NMR (400 MHz, $CDCl_3$) δ = 7.19-7.35 (m, 7 H), 7.08-7.18 (m, 3 H), 5.62 (br. s., 1 H), 4.38 (d, *J* = 5.5 Hz, 2 H), 2.62 (t, *J* = 7.5 Hz, 2 H), 2.16 (t, *J* = 7.5 Hz, 2 H), 1.85-2.02 (m, 2 H) ppm; ^{13}C-NMR (101 MHz, $CDCl_3$) δ = 172.4, 141.4, 138.3, 128.7, 128.5, 128.4, 127.9, 127.5, 126.0, 43.6, 35.9, 35.2, 27.1 ppm; IR (ATR): $\tilde{\nu}$ = 3285 (w), 3062 (w), 3023 (w), 2950 (w), 2921 (w), 2873 (w), 1642 (m), 1543 (m), 1495 (w), 1452 (m), 1414 (w), 1384 (w), 1268 (w), 1214 (m), 1077 (w), 1029 (w), 1002 (w), 904 (w), 740 (m), 694 (vs) cm^{-1}; MS (Ion trap, EI): m/z (%) = 253.1 (18), 149.1 (100), 148.3 (24), 106.1 (34), 91.1 (58), 65.1 (19), 40.0 (13); CHN elemental analysis calcd. for $C_{17}H_{19}NO$: N, 5.39; C, 80.60; H, 7.64; found N, 5.39; C, 80.40; H,

7.64; melting point: 79.0 °C. The analytical data matched those reported in the literature for *N*-benzyl-4-phenylbutanamide[21].

Synthesis of ethyl *N*-benzyl-3-phenylpropiolamide (5va) [CAS: 55330-55-7]. Compound **5va** was prepared following the general procedure B, starting from phenylpropiolicacid (**1v**) (149 mg, 1 mmol). After purification **5va** was isolated as a white solid (170 mg, 72 %) as a 9:1 mixture of rotameres A and B. ^{1}H-NMR (400 MHz, $CDCl_3$) δ = 7.49-7.65 (m, 2 H, rotamere A), 7.28-7.48 (m, 8 H, rotamere B), 6.20-6.48 (m, 1 H, rotamere A+B), 4.70 (d, J = 5.5 Hz, 1 H, rotamere A), 4.54 (d, J = 5.5 Hz, 2 H, rotamere B) ppm; ^{13}C-NMR (101 MHz, $CDCl_3$) δ = 153.3, 137.3, 132.6, 132.5, 130.4, 130.1, 128.9, 128.8, 128.5, 128.5, 128.0, 127.8, 127.2, 120.1, 85.1, 82.9, 47.4, 43.9 ppm; IR (ATR): $\tilde{\nu}$ = 3270 (m), 3064 (w), 2218 (w), 1633 (vs), 1546 (s), 1488 (m), 1454 (w), 1423 (w), 1299 (m), 1226 (m), 1081 (w), 1028 (w), 996 (w), 919 (w), 881 (w), 761 (s), 723 (m), 679 (vs) cm^{-1}; MS (Ion trap, EI): m/z (%) = 235.1 (67), 234.2 (66), 129.1 (91), 73.0 (84), 40.0 (100); CHN elemental analysis calcd. for $C_{16}H_{13}NO$: N, 5.95; C, 81.68; H, 5.57; found N, 5.94; C, 81.38; H, 5.71; melting point: 136.6 °C. The analytical data matched those reported in the literature for *N*-benzyl-3-phenylpropiolamide[7].

Synthesis of *N*-benzylfuran-2-carboxamide (5wa) [CAS: 10354-48-0]. Compound **5wa** was prepared following the general procedure B, starting from 2-furancarboxylic acid (**1w**) (114 mg, 1 mmol). After purification **5wa** was isolated as a white solid (201.0 mg, 99 %). ^{1}H-NMR (400 MHz, $CDCl_3$) δ = 7.42 (dd, J = 1.7, 0.7 Hz, 10 H), 7.28-7.39 (m, 5 H), 7.16 (dd, J = 3.5, 0.7 Hz, 1 H), 6.68 (br. s., 1 H), 6.51 (dd, J = 3.5, 1.8 Hz, 1 H), 4.63 (d, J = 5.9 Hz, 2 H) ppm; ^{13}C-NMR (101 MHz, $CDCl_3$) δ = 158.2, 147.9, 143.9, 138.0, 128.7, 127.9, 127.6, 114.4, 112.2, 43.1 ppm; IR (ATR): $\tilde{\nu}$ = 3283 (m), 3125 (w), 3062 (w), 3029 (w), 1737 (w), 1638 (s), 1571 (s), 1542 (vs), 1475 (m), 1455 (w), 1431 (m), 1372 (w), 1316 (s), 1249 (m), 1191 (m), 1144 (w), 1083 (w), 1037 (w), 991 (m), 924 (w), 885 (w), 838 (w), 735 (s), 698 (vs) cm^{-1}; MS (Ion trap, EI): m/z (%) = 202.1 (30), 201.1 (100), 106.1 (49), 95.0 (47), 91.1 (14), 79.1 (11), 77.1 (13); CHN elemental analysis calcd. for $C_{12}H_{11}NO_2$: N, 6.96; C, 71.63; H, 5.51; found N, 6.86; C, 71.44; H, 5.58; melting point: 111.6 °C. The analytical data matched those reported in the literature for *N*-benzylfuran-2-carboxamide[22].

Synthesis of (9Z,12Z)-*N*-benzyloctadeca-9,12-dienamide (5xa) [CAS: 18286-71-0]. Compound **5xa** was prepared following the general procedure B, starting from linoleic acid (**1x**) (312 mg, 1 mmol). After purification **5xa** was isolated as a yellow oil (370 mg, 99 %). ^{1}H-NMR (400 MHz, $CDCl_3$) δ = 7.14-7.38 (m, 5 H), 5.65 (br. s., 1 H), 5.19-5.41 (m, 4 H), 4.39 (d, J = 5.8 Hz, 2 H), 2.72 (t, J = 6.5 Hz, 2 H), 2.05-2.22 (m, 2 H), 2.00 (q, J = 7.3 Hz, 4 H), 1.58-1.64 (m, 2 H), 1.19-1.37 (m, 14 H), 0.70-0.95 (m, 3 H) ppm; ^{13}C-NMR (101 MHz, $CDCl_3$) δ = 172.9, 160.2, 138.4, 130.2, 130.0, 128.7, 128.0, 127.9, 127.8, 127.5, 43.6, 36.8, 31.5, 29.6, 29.3, 29.3, 29.2, 29.1, 27.2, 25.7, 25.6, 22.6, 14.1 ppm; IR (ATR): $\tilde{\nu}$ = 2925 (m), 2855 (w), 1737 (m), 1644 (m), 1544 (m), 1455 (m), 1365 (m), 1217 (m), 1080 (w), 1029 (w), 973 (w), 727 (m), 697 (vs) cm^{-1}; MS (Ion trap, EI): m/z (%) = 370 (1), 396 (4), 341 (98), 281 (52), 267 (23), 231 (18), 209 (23), 149 (22), 106 (8), 91 (23), 73 (100), 44 (55); HRMS-EI (TOF) calcd. for $C_{25}H_{39}NO$: 396.3032; found: 396.3047 [M^+]. The analytical data matched those reported in the literature for (9Z,12Z)-*N*-benzyloctadeca-9,12-dienamide[23].

Synthesis of (S,S)-*N,N'*-dibenzyltartramide (5ya) [CAS: 108321-43-3]. Compound **5ya** was prepared following the general procedure B, starting from L-(+)-tartaric acid (**1y**) (152 mg, 1.0 mmol) and 3 mmol of ethoxyacetylene and benzylamine. After purification **5ya** was isolated as a white solid (270 mg, 82 %). ^{1}H-NMR (400 MHz, $CDCl_3$) δ = 8.26 (t, J = 6.2 Hz, 2 H), 7.26-7.32 (m, 8 H), 7.22 (dq, J = 8.4, 4.3 Hz, 2 H), 5.74 (d, J = 7.0 Hz, 2 H), 4.27-4.42 (m, 6 H) ppm; ^{13}C-NMR (101 MHz, $CDCl_3$) δ = 172.2, 139.5, 128.1, 127.1, 126.6, 72.8, 41.9 ppm; IR (ATR): $\tilde{\nu}$ = 3349 (m), 3310 (m), 3064 (w), 3034 (w), 2926 (w), 1738 (w), 1625 (m), 1541 (m), 1493 (m), 1455 (m), 1425 (m), 1361 (m), 1320 (m), 1279 (m), 1243 (m), 1217 (m), 1094 (m), 1066 (s), 1026 (m), 922 (w), 816 (w), 739 (vs), 693 (vs) cm^{-1}; MS (Ion trap, EI): m/z (%) = 240.1 (19), 239.1 (100), 238.2 (38), 133.0 (63), 105.0 (25), 76.9 (34), 51.0 (18); CHN elemental analysis calcd. for $C_{18}H_{20}N_2O_4$: N, 8.53; C, 65.84; H, 6.14; found N, 8.53; C, 65.66; H, 6.15; melting point: 202.1 °C. The analytical data matched those reported in the literature for (S,S)-*N,N'*-dibenzyltartramide[24].

Synthesis of ethyl *N*-(2-benzylamino-2-oxoethyl)benzamide (5za) [CAS: 3392-91-4]. Compound **5za** was prepared following the general procedure B, starting from hippuric acid (**1z**) (183 mg, 1 mmol). After purification **5za** was isolated as a white solid (237 mg, 88 %). ^{1}H-NMR (400 MHz, DMSO-d_6) δ = 8.81 (t, J = 5.9 Hz, 1 H),

8.46 (t, *J* = 6.0 Hz, 1 H), 7.88-7.92 (m, 2 H), 7.45-7.56 (m, 3 H), 7.21-7.34 (m, 4 H), 4.30 (d, *J* = 6.0 Hz, 2 H), 3.92 (d, *J* = 6.0 Hz, 2 H) ppm; ^{13}C-NMR (101 MHz, DMSO-d_6) δ = 169.0, 166.5, 139.5, 134.0, 131.3, 128.3, 128.2, 127.4, 127.2, 126.7, 42.8, 42.0 ppm; IR (ATR): $\tilde{\nu}$ = 3286 (w), 3067 (w), 3032 (w), 2911 (w), 1737 (w), 1665 (m), 1637 (s), 1546 (vs), 1492 (m), 1454 (m), 1398 (w), 1366 (m), 1315 (m), 1243 (m), 1080 (w), 1010 (m), 987 (w), 870 (w), 802 (w), 745 (w), 688 cm^{-1}; MS (Ion trap, EI): m/z (%) = 269 (7), 268 (13), 162 (59), 134 (57), 106 (54), 105 (100), 91 (38), 77 (68), 65 (11), 44 (17), 40 (27); CHN elemental analysis calcd. for $C_{16}H_{16}N_2O_2$: N, 10.44; C, 71.62; H, 6.01; found N, 10.43; C, 71.47; H, 6.01; melting point: 162.1 °C. The analytical data matched those reported in the literature for *N*-(2-benzylamino-2-oxoethyl)benzamide[25].

Synthesis of (S)-(+)-*N*-benzylmandelamide (5aaa) [CAS: 82270-62-0]. Compound **5aaa** was prepared following the general procedure B, starting from (S)-α-hydroxybenzeneacetic acid (**1aa**) (155 mg, 1 mmol). After purification **5aaa** was isolated as a white solid (241 mg, 99 %). ^{1}H-NMR (400 MHz, $CDCl_3$) δ = 7.22-7.42 (m, 7 H), 7.14-7.21 (m, 2 H), 6.52 (br. s., 1 H), 5.04 (d, *J* = 3.6 Hz, 1 H), 4.44 (dd, *J* = 14.9, 5.9 Hz, 1 H), 4.39 (dd, *J* = 14.8, 5.8 Hz, 1 H), 3.67 (d, *J* = 3.5 Hz, 1 H) ppm; ^{13}C-NMR (101 MHz, $CDCl_3$) δ = 172.0, 139.3, 137.6, 128.9, 128.7, 127.6, 127.6, 126.8, 74.2, 43.5 ppm; IR (ATR): $\tilde{\nu}$ = 3405 (w), 3176 (w), 1738 (w), 1648 (s), 1535 (m), 1493 (w), 1441 (w), 1340 (w), 1288 (w), 1238 (w), 1207 (w), 1095 (w), 1066 (m), 1029 (w), 923 (w), 852 (w), 757 (s), 736 (m), 704 (vs) cm^{-1}; MS (Ion trap, EI): m/z (%) = 241 (1), 207 (8), 107 (16), 106 (10), 91 (32), 79 (34), 65 (9), 51 (10), 40 (99); CHN elemental analysis calcd. for $C_{15}H_{15}NO_2$: N, 5.80; C, 74.67; H, 6.27; found N, 5.74; C, 74.45; H, 6.33; melting point: 135.2 °C. The analytical data matched those reported in the literature for (S)-(+)-*N*-benzylmandelamide[26].

Synthesis of *N*,*N'*-dibenzyl-L-malamide (5aba) [CAS: 173654-52-9]. Compound **5aba** was prepared following the general procedure B, starting from L-(-)-malic acid (**1ab**) (134 mg, 1.0 mmol). After purification **5aba** was isolated as a white solid (189 mg, 61 %). ^{1}H-NMR (400 MHz, DMSO-d_6) δ = 8.27 (t, *J* = 6.2 Hz, 2 H), 7.19-7.38 (m, 10 H), 5.76 (d, *J* = 7.2 Hz, 2 H), 4.23-4.44 (m, 6 H) ppm; ^{13}C-NMR (101 MHz, DMSO-d_6) δ = 172.2, 139.5, 128.3, 127.1, 126.6, 72.8, 41.8 ppm; IR (ATR): $\tilde{\nu}$ = 3310 (w), 3065 (w), 3034 (w), 2970 (w), 2929 (w), 1738 (m), 1649 (w), 1619 (m), 1528 (s), 1492

(m), 1455 (m), 1425 (m), 1362 (m), 1303 (m), 1277 (m), 1232 (m), 1079 (m), 1056 (m), 1020 (w), 884 (w), 738 (s), 695 (vs) cm^{-1}; HRMS-EI (TOF) calcd. for $C_{18}H_{20}N_2O_3$: 312.1474; found: 312.1489 [M^+]; CHN elemental analysis calcd. for $C_{18}H_{20}N_2O_3$: N, 8.97; C, 69.21; H, 6.45; found N, 8.77; C, 68.87; H, 6.65; melting point: 155.7°C. The analytical data matched those reported in the literature for *N*,*N*'-dibenzyl-L-malamide[27].

Synthesis of *N*-cyclohexylbenzamide (5ab) [CAS: 1759-68-8]. Compound **5ab** was prepared following the general procedure A or C, starting from cyclohexylamine (**4b**) (150 mg, 133 µl, 1.5 mmol). After purification **5ab** was isolated as a white solid (A: 21 mg, 10 %; C: 176 mg, 87 %). ^{1}H-NMR (400 MHz, $CDCl_3$) δ = 7.73-7.79 (m, 2 H), 7.39-7.51 (m, 3 H), 5.93-6.17 (m, 1 H), 3.94-4.07 (m, 1 H), 1.98-2.08 (m, 2 H), 1.71-1.81 (m, 2 H), 1.66 (dt, *J* = 12.9, 3.7 Hz, 1 H), 1.35-1.49 (m, 2 H), 1.14-1.31 (m, 3 H) ppm; ^{13}C-NMR (101 MHz, $CDCl_3$) δ = 166.6, 135.1, 131.2, 128.4, 126.8, 48.6, 33.2, 25.5, 24.9 ppm; IR (ATR): $\tilde{\nu}$ = 3324 (w), 3072 (w), 2928 (m), 2851 (m), 1812 (w), 1627 (vs), 1577 (m), 1530 (vs), 1489 (m), 1446 (m), 1328 (s), 1291 (m), 1255 (m), 1152 (m), 1084 (m), 1030 (w), 971 (w), 924 (w), 891 (m), 846 (w), 804 (w), 721 (m), 692 (vs), 656 (s) cm^{-1}; MS (Ion trap, EI): m/z (%) = 204.0 (24), 203.0 (40), 122.0 (67), 105.0 (100), 79.1 (11), 77.1 (39), 51.0 (13); CHN elemental analysis calcd. for $C_{13}H_{17}NO$: N, 6.89; C, 67.81; H, 8.43; found N, 6.78; C, 76.63; H, 8.40; melting point: 149.2 °C. The analytical data matched those reported in the literature for *N*-cyclohexylbenzamide[28].

Synthesis of *N*-ethylbenzamide (5ac) [CAS: 614-17-5]. Compound **5ac** was prepared following the general procedure A or C, starting from ethylamine (**4c**) (97 mg, 112 µl, 1.5 mmol). After purification **5ac** was isolated as a white solid (A: 51 mg, 34 %; C: 130 mg, 87 %). ^{1}H-NMR (400 MHz, $CDCl_3$) δ = 7.75-7.79 (m, 2 H), 7.41-7.53 (m, 2 H), 6.12 (br. s., 1 H), 3.52 (qd, *J* = 7.2, 5.6 Hz, 2 H), 1.27 (t, *J* = 7.3 Hz, 3 H) ppm; ^{13}C-NMR (101 MHz, $CDCl_3$) δ = 134.8, 131.3, 128.5, 126.8, 34.9, 14.9 ppm; IR (ATR): $\tilde{\nu}$ = 3315 (w), 3081 (w), 2978 (w), 2935 (w), 1737 (w), 1634 (m), 1602 (w), 1545 (vs), 1487 (w), 1432 (w), 1310 (s), 1145 (m), 1088 (w), 1040 (w), 929 (w), 868 (m), 804 (w), 719 (m), 692 (vs), 658 (s) cm^{-1}; MS (Ion trap, EI): m/z (%) = 150.0 (16), 148.9 (19), 148.1 (44), 105.0 (100), 77.1 (47), 51.0 (20), 50.0 (12); HRMS-EI (TOF)

calcd. for $C_9H_{11}NO$: 148.0762; found: 148.0757 [M^+]; melting point: 69.6 °C. The analytical data matched those reported in the literature for *N*-ethylbenzamide[29])

Synthesis of *N*-(*n*-butyl)benzamide (5ad) [CAS: 2782-40-3]. Compound **5ad** was prepared following the general procedure A or C, starting from *n*-butylamine (**4d**) (111 mg, 150 µl, 1.5 mmol). After purification **5ad** was isolated as a pale yellow oil (A: 48 mg, 27 %; C: 138 mg, 78 %). ^{1}H-NMR (400 MHz, $CDCl_3$) δ = 7.74-7.79 (m, 2 H), 7.39-7.53 (m, 3 H), 6.15 (br. s., 1 H), 3.41-3.51 (m, 2 H), 1.56-1.68 (m, 2 H), 1.36-1.48 (m, 2 H), 0.97 (td, *J* = 7.3, 1.8 Hz, 3 H) ppm; ^{13}C-NMR (101 MHz, $CDCl_3$) δ = 167.5, 134.8, 131.3, 128.5, 126.8, 39.8, 31.7, 20.1, 13.8 ppm; IR (ATR): $\tilde{\nu}$ = 3304 (w), 2957 (w), 2930 (w), 2871 (w), 1635 (s), 1538 (s), 1490 (m), 1435 (w), 1306 (m), 1153 (w), 1075 (w), 1026 (w), 946 (w), 851 (w), 803 (w), 692 (vs) cm^{-1}; MS (Ion trap, EI): m/z (%) = 178.0 (13), 134.9 (12), 134.0 (17), 106.0 (8), 105.0 (100), 77.1 (31), 51.0 (13); HRMS-EI (TOF) calcd. for $C_{12}H_{15}NO$: 177.1154; found: 177.1152 [M^+]. The analytical data matched those reported in the literature for *N*-(*n*-butyl)benzamide[30].

Synthesis of *N,N*-diethylbenzamide (5ae) [CAS: 1696-17-9]. Compound **5ae** was prepared following the general procedure C, starting from diethylamine (**4e**) (111 mg, 156 µl, 1.5 mmol). After purification **5ae** was isolated as a brown oil (53 mg, 30 %). ^{1}H-NMR (400 MHz, $CDCl_3$) δ = 7.35-7.42 (m, 5 H), 3.55 (br. s., 2 H), 3.27 (br. s., 2 H), 1.26 (br. s., 3 H), 1.12 (br. s., 3 H) ppm; ^{13}C-NMR (101 MHz, $CDCl_3$) δ = 171.2, 137.1, 128.9, 128.2, 126.1, 43.1, 39.1, 14.1, 12.8 ppm; IR (ATR): $\tilde{\nu}$ = 2970 (w), 2935 (w), 1717 (w), 1626 (s), 1426 (m), 1365 (m), 1286 (m), 1218 (m), 1096 (m), 1070 (w), 1027 (w), 943 (w), 872 (w), 784 (m), 704 (vs) cm^{-1}; MS (Ion trap, EI): m/z (%) = 177.0 (10), 176.1 (48), 106.0 (8), 105.0 (100), 77.1 (38), 51.0 (13), 50.0 (7); CHN elemental analysis calcd. for $C_{11}H_{15}NO$: N, 7.90; C, 74.54; H, 8.53; found N, 7.65; C, 74.24; H, 8.56; HRMS-EI (TOF) calcd. for $C_{11}H_{15}NO$: 177.1154; found: 177.1159 [M^+]. The analytical data matched those reported in the literature for *N,N*-diethylbenzamide[31].

Synthesis of *N*-phenylbenzamide (5af) [CAS: 93-98-1]. Compound **5af** was prepared following the general procedure C, starting from aniline (**4f**) (140 mg, 137 µl, 1.5 mmol). After purification **5af** was isolated as a white solid (35 mg, 18 %). ^{1}H-NMR (400 MHz, DMSO-d_6) δ = 10.25 (s, 1 H), 7.90-8.00 (m, 2 H), 7.79 (dd, *J* = 8.7, 1.1 Hz, 2 H), 7.49-7.63 (m, 3 H), 7.35 (dd, *J* = 8.5, 7.5 Hz, 2 H), 7.06-7.15 (m, 1 H) ppm; ^{13}C-

NMR (101 MHz, $CDCl_3$) δ = 165.6, 139.2, 135.0, 131.5, 128.6, 128.4, 127.6, 123.7, 120.4 ppm; IR (ATR): $\tilde{\nu}$ = 3342 (w), 3052 (w), 2921 (w), 2851 (w), 1747 (w), 1654 (m), 1599 (m), 1525 (s), 1490 (m), 1436 (s), 1320 (m), 1257 (m), 1178 (w), 1113 (w), 1075 (w), 1027 (m), 1001 (w), 978 (w), 909 (w), 885 (w), 791 (w), 747 (vs), 714 (s), 688 (vs), 650 (s) cm^{-1}; MS (Ion trap, EI): m/z (%) = 197.8 (12), 196.8 (83), 104.9 (100), 77.0 (56), 64.9 (10), 51.0 (17), 49.9 (11); HRMS-EI (TOF) calcd. for $C_{13}H_{11}NO$: 197.0841; found: 197.0843 [M^+]; melting point: 160.0 °C. The analytical data matched those reported in the literature for *N*-phenylbenzamide[32].

Synthesis of benzamide (5ag) [CAS: 55-21-0]**.** Compound **5ag** was prepared following the general procedure C, starting from aqueous ammonia (**4g**) (25wt%, 210 mg, 232 µl, 1.5 mmol). After purification **5ag** was isolated as a white solid (111 mg, 92 %). ^{1}H-NMR (400 MHz, $CDCl_3$) δ = 7.79-7.86 (m, 2 H), 7.50-7.57 (m, 1 H), 7.40-7.49 (m, 2 H), 6.25 (br. s., 2 H) ppm; ^{13}C-NMR (101 MHz, $CDCl_3$) δ = 169.6, 133.3, 132.0, 128.6, 127.3 ppm; IR (ATR): $\tilde{\nu}$ = 3363 (m), 3164 (m), 3065 (w), 1739 (w), 1655 (s), 1623 (s), 1577 (s), 1448 (m), 1396 (s), 1298 (m), 1217 (m), 1180 (w), 1143 (m), 1122 (m), 1025 (w), 1001 (w), 925 (w), 769 (s), 683 (vs), 650 (s) cm^{-1}; MS (Ion trap, EI): m/z (%) = 100.0 (100), 99.0 (24), 98.0 (64), 70.1 (18), 44.1 (39), 42.1 (36), 41.1 (21); CHN elemental analysis calcd. for C_7H_7NO: N, 11.56; C, 69.41; H, 5.82; found N, 11.23; C, 69.50; H, 6.02; melting point: 124.2 °C. The analytical data matched those reported in the literature for benzamide[33].

Synthesis of *N*-*tert*-butylbenzamide (5ah) [CAS: 5894-65-5]. Compound **5ah** was prepared following the general procedure C, starting from *tert*-butylamine (**4h**) (110 mg, 157 µl, 1.5 mmol). After purification **5ah** was isolated as a white solid (72 mg, 41 %). ^{1}H-NMR (400 MHz, $CDCl_3$) δ = 7.68-7.76 (m, 2 H), 7.37-7.51 (m, 3 H), 5.95 (br. s., 1 H), 1.48 (s, 9 H) ppm; ^{13}C-NMR (101 MHz, $CDCl_3$) δ = 166.9, 135.9, 131.1, 128.5, 126.7, 51.6, 28.8 ppm; IR (ATR): $\tilde{\nu}$ = 3319 (w), 3064 (w), 2966 (w), 1739 (w), 1634 (s), 1530 (vs), 1491 (m), 1451 (m), 1392 (w), 1360 (m), 1311 (s), 1217 (s), 1079 (w), 1028 (w), 937 (w), 876 (w), 805 (w), 716 (s), 693 (vs), 668 (m), 650 (m) cm^{-1}; MS (Ion trap, EI): m/z (%) = 176.8 (10), 161.8 (28), 122.0 (10), 106.0 (8), 105.0 (100), 77.1 (30), 51.0 (12); CHN elemental analysis calcd. for $C_{11}H_{15}NO$: N, 7.90; C, 74.54; H, 8.53; found N, 7.65; C, 74.24; H, 8.56; melting point: 132.9°C. The analytical data

matched those reported in the literature for *N-tert*-butylbenzamide (Z. Xia, Q. Zhu, *Org. Lett.* **2013**, *15*, 4110–4113).

Synthesis of 4-benzoylpiperidine (5ai) [CAS: 776-75-0]. Compound **5ai** was prepared following the general procedure C, starting from piperidine (**4i**) (129 mg, 1.5 mmol). After purification **5ai** was isolated as a white solid (173 mg, 92 %). ^{1}H-NMR (400 MHz, $CDCl_3$) δ = 7.39 (s, 5 H), 3.71 (br. s., 2 H), 3.34 (br. s., 2 H), 1.68 (br. s., 4 H), 1.52 (br. s., 2 H) ppm; ^{13}C-NMR (101 MHz, $CDCl_3$) δ = 170.3, 136.5, 129.3, 128.4, 126.7, 48.7, 43.1, 26.5, 25.6, 24.6 ppm; IR (ATR): $\tilde{\nu}$ = 2935 (w), 2854 (w), 1623 (vs), 1428 (vs), 1273 (vs), 1109 (w), 1027 (w), 1002 (m), 955 (w), 883 (w), 853 (w), 786 (m), 730 (m), 706 (vs) cm^{-1}; MS (Ion trap, EI): m/z (%) = 190 (12), 188 (29), 105 (100), 77 (65), 51 (26), 50 (12), 44 (28); CHN elemental analysis calcd. for $C_{12}H_{15}NO$: N, 7.40; C, 76.16; H, 7.99; found N, 7.50; C, 75.80; H, 7.99; HRMS-EI (TOF) calcd. for $C_{12}H_{15}NO$: 189.1153; found: 189.1150 [M$^+$]; melting point: 50.0 °C. The analytical data matched those reported in the literature for 4-benzoylpiperidine[34].

Synthesis of 4-benzoylmorpholine (5aj) [CAS: 1468-28-6]. Compound **5aj** was prepared following the general procedure C, starting from morpholine (**4j**) (132 mg, 133 µl, 1.5 mmol). After purification **5aj** was isolated as a white solid (133 mg, 70 %). ^{1}H-NMR (400 MHz, $CDCl_3$) δ = 7.38-7.48 (m, 5 H), 3.36-3.93 (m, 8 H) ppm; ^{13}C-NMR (101 MHz, $CDCl_3$) δ = 170.4, 135.3, 129.9, 128.5, 127.0, 66.9, 48.2, 42.6 ppm; IR (ATR): $\tilde{\nu}$ = 2911 (w), 2858 (w), 1624 (s), 1577 (w), 1424 (s), 1298 (w), 1270 (s), 1147 (w), 1110 (s), 1064 (w), 1019 (m), 933 (m), 889 (w), 839 (m), 796 (m), 735 (m), 710 (vs) cm^{-1}; MS (Ion trap, EI): m/z (%) = 192.0 (20), 191.0 (12), 190.0 (43), 105.0 (100), 77.1 (45), 56.1 (12), 51.0 (21); HRMS-EI (TOF) calcd. for $C_{10}H_{12}NO_2$: 191.0946; found: 191.0934 [M$^+$]; melting point: 73.5 °C. The analytical data matched those reported in the literature for 4-benzoylmorpholine[35].

Synthesis of benzoylpyrrolidin-2-one (5ak) [CAS: 2399-66-8]. Compound **5ak** was prepared following the general procedure C, starting from 2-pyrrolidone (**4k**) (128 mg, 116 µl, 1.5 mmol). After purification **5ak** was isolated as a brown solid (24 mg, 13 %). ^{1}H-NMR (400 MHz, $CDCl_3$) δ = 7.58-7.66 (m, 2 H), 7.38-7.56 (m, 3 H), 3.97 (t, J = 7.1 Hz, 2 H), 2.62 (t, J = 8.0 Hz, 2 H), 2.08-2.19 (m, 2 H) ppm; ^{13}C-NMR (101 MHz, $CDCl_3$) δ = 174.6, 170.7, 134.3, 133.5, 131.9, 130.1, 128.9, 128.4, 127.7, 46.5, 33.3,

17.6 ppm; IR (ATR): $\tilde{\nu}$ = 2931 (w), 2900 (w), 1740 (s), 1661 (vs), 1600 (w), 1447 (w), 1363 (m), 1307 (vs), 1232 (s), 1190 (s), 1027 (m), 931 (w), 889 (w), 827 (w), 792 (m), 734 (s), 701 (vs), 655 (s) cm^{-1}; MS (Ion trap, EI): m/z (%) = 188.9 (24), 188.1 (27), 106.0 (10), 105.0 (100), 77.1 (52), 51.0 (21), 50.0 (12); HRMS-EI (TOF) calcd. for $C_{11}H_{11}NO_2$: 189.0789; found: 189.0803 [M^+]; melting point: 85.2 °C. The analytical data matched those reported in the literature for benzoylpyrrolidin-2-one[36].

Synthesis of *N*-carboxybenzoyl-L-phenylalanyl-L-phenylalanine ethyl ester (5acl) [CAS: 5276-63-1]. Compound **5acl** was prepared following the general procedure D, starting from *N*-carboxybenzoyl-L-phenylalanine (**4l**) (299 mg, 1.0 mmol) and L-phenylalanine ethyl ester hydrochloride (347 mg, 1.5 mmol). After purification **5acl** was isolated as a white solid (344 mg, 73 %). ^{1}H-NMR (600 MHz, DMSO-*d₆*) δ = 8.48 (d, *J* = 7.6 Hz, 1 H), 7.47 (d, *J* = 8.8 Hz, 2 H), 7.16-7.36 (m, 45 H), 4.93 (s, 5 H), 4.44-4.51 (m, 3 H), 4.23-4.33 (m, 3 H), 4.03 (q, *J* = 7.2 Hz, 5 H), 2.91-3.08 (m, 8 H), 2.70 (dd, *J* = 13.6, 11.0 Hz, 3 H), 1.06-1.11 (m, 3 H); ^{13}C-NMR (151 MHz, DMSO-d_6) δ = 171.8, 171.3, 155.8, 138.0, 137.0, 129.3, 129.2, 129.1, 129.1, 128.3, 128.3, 128.2, 128.0, 128.0, 127.7, 127.4, 127.4, 126.8, 126.6, 126.3, 65.2, 60.5, 55.8, 53.7, 37.4, 36.7, 26.4, 14.0, 13.9 ppm; IR (ATR): $\tilde{\nu}$ = 3296 (w), 3062 (w), 3030 (w), 2927 (w), 1728 (m), 1695 (m), 1649 (s), 1533 (m), 1495 (w), 1452 (w), 1370 (w), 1346 (w), 1285 (s), 1242 (s), 1183 (m), 1039 (m), 745 (m), 695 (vs), 669 (m) cm^{-1}; CHN elemental analysis calcd. for $C_{22}H_{26}N_2O_6$: N, 5.90; C, 70.87; H, 6.37; found N, 5.86; C, 70.78; H, 6.26; HRMS-EI (TOF) calcd. for $C_{28}H_{30}N_2O_5$: 474.2154; found: 366.1660 [M^+-C_7H_7O]; melting point: 162.4 °C. The analytical data matched those reported in the literature for *N*-carboxybenzoyl-L-phenylalanyl-L-phenylalanine ethyl ester[37].

Synthesis of *N*-(*N*-carboxybenzoxyl-L-phenylalanyl)-L-serin ethyl ester (5acm). Compound **5acm** was prepared following the general procedure D, starting from *N*-carboxybenzoyl-L-phenylalanine (**1ac**) (299 mg, 1.0 mmol) and L-serine ethyl ester hydrochloride (**4m**) (257 mg, 1.5 mmol). After purification **5acm** was isolated as a white solid (223 mg, 54 %).^{1}H-NMR (600 MHz, DMSO-d_6) δ = 8.47 (d, *J* = 7.5 Hz, 1 H), 7.52 (d, *J* = 8.8 Hz, 1 H), 7.06-7.39 (m, 10 H), 5.15 (t, *J* = 5.5 Hz, 1 H), 4.89-4.95 (m, 2 H), 4.31-4.44 (m, 2 H), 4.09 (q, *J* = 7.0 Hz, 2 H), 3.71-3.81 (m, 1 H), 3.62-3.71 (m, 1 H), 3.05 (dd, *J* = 13.8, 3.3 Hz, 1 H), 2.72 (dd, *J* = 13.7, 11.4 Hz, 1 H), 1.18 (t, *J* = 7.0 Hz, 3 H) ppm; ^{13}C-NMR (151 MHz, DMSO-d_6) δ = 172.0, 170.5, 155.9, 138.2,

137.0, 129.3, 128.3, 128.0, 127.7, 127.4, 126.8, 126.2, 65.2, 61.2, 60.6, 55.9, 54.8, 37.5, 14.1 ppm; IR (ATR): $\tilde{\nu}$ = 3297 (w), 2970 (w), 2940 (w), 1740 (s), 1695 (m), 1649 (s), 1535 (s), 1455 (w), 1375 (m), 1260 (s), 1206 (vs), 1125 (m), 1042 (s), 911 (w), 865 (w), 746 (s), 696 (vs) cm^{-1}; CHN elemental analysis calcd. for $C_{22}H_{26}N_2O_6$: N, 6.76; C, 63.76; H, 6.32; found N, 6.68; C, 63.82; H, 6.32; HRMS-EI (TOF) calcd. for $C_{22}H_{26}N_2O_6$: 396.1685; found: 369.1680 [M^+-H_2O]; melting point: 121.4 °C.

Synthesis of *N*-(*N*-carboxybenzoyl-L-phenylalanyl)-glycine methyl ester (5cn) [CAS: 4818-07-9]. Compound **5cn** was prepared following the general procedure D, starting from *N*-carboxybenzoyl-L-phenylalanine (**1c**) (299 mg, 1.0 mmol) and glycine methyl ester hydrochloride (**4n**) (188 mg, 1.5 mmol). After purification **5cn** was isolated as a white solid (267 mg, 72 %).^{1}H-NMR (600 MHz, DMSO-*d*$_6$) δ = 8.52 (t, *J* = 5.9 Hz, 1 H), 7.56 (d, *J* = 8.8 Hz, 1 H), 7.19-7.34 (m, 9 H), 4.89-4.97 (m, 2 H), 4.30 (ddd, *J* = 10.9, 8.8, 3.8 Hz, 1 H), 3.92 (dd, *J* = 17.3, 5.9 Hz, 1 H), 3.86 (dd, *J* = 17.3, 5.9 Hz, 1 H), 3.64 (s, 3 H), 3.03 (dd, *J* = 13.9, 3.7 Hz, 1 H), 2.75 (dd, *J* = 13.8, 11.2 Hz, 1 H); ^{13}C-NMR (151 MHz, DMSO-*d*$_6$) δ = 172.2, 170.3, 155.9, 138.2, 137.0, 129.2, 128.3, 128.1, 127.7, 127.4, 126.3, 65.2, 56.0, 51.7, 40.6, 37.4 ppm; IR (ATR): $\tilde{\nu}$ = 3297 (w), 3029 (w), 2970 (w), 2949 (w), 1740 (vs), 1691 (m), 1645 (s), 1539 (s), 1435 (w), 1373 (m), 1351 (m), 1311 (m), 1262 (s), 1218 (vs), 1142 (w), 1048 (m), 1007 (w), 909 (w), 847 (w), 741 (m), 700 (s), 677 (s) cm^{-1}; CHN elemental analysis calcd. for $C_{20}H_{22}N_2O_5$: N, 7.56; C, 64.85; H, 5.99; found N, 7.50; C, 64.55; H, 6.06; HRMS-EI (TOF) calcd. for $C_{20}H_{22}N_2O_5$: 370.1529; found: 262.0957 [M+-C_7H_8O]; melting point: 116.6 °C. The analytical data matched those reported in the literature for *N*-(*N*-carboxybenzoyl-L-phenylalanyl)-glycine ethyl ester[38].

Synthesis of *N*-(*N*-carboxybenzoyl-L-phenylalanyl)-L-leucine ethyl ester (5aco) [CAS: 2953-42-6]. Compound **5aco** was prepared following the general procedure D, starting from *N*-carboxybenzoyl-L-phenylalanine (**1ac**) (299 mg, 1.0 mmol) and L-leucine ethyl ester hydrochloride (**4o**) (297 mg, 1.5 mmol). After purification **5aco** was isolated as a white solid (424 mg, 96 %).^{1}H-NMR (600 MHz, DMSO-*d*$_6$) δ = 8.40 (d, *J* = 7.6 Hz, 1 H), 7.50 (d, *J* = 8.8 Hz, 1 H), 7.15-7.38 (m, 10 H), 4.94 (d, *J* = 2.1 Hz, 2 H), 4.25-4.35 (m, 2 H), 4.03-4.14 (m, 2 H), 3.01 (dd, *J* = 13.9, 3.7 Hz, 1 H), 2.74 (dd, *J* = 13.8, 10.9 Hz, 1 H), 1.63-1.73 (m, 1 H), 1.48-1.63 (m, 2 H), 1.14-1.21 (m, 3 H), 0.93 (d, *J* = 6.7 Hz, 3 H), 0.87 (d, *J* = 6.5 Hz, 3 H); ^{13}C-NMR (151 MHz, DMSO-*d*$_6$) δ

= 172.4, 171.9, 155.8, 138.1, 137.0, 129.2, 128.3, 128.0, 127.7, 127.4, 126.3, 65.1, 60.4, 55.8, 50.5, 40.0, 39.9, 39.8, 39.6, 39.4, 39.2, 39.1, 37.4, 24.2, 22.8, 21.3, 14.0 ppm; IR (ATR): $\tilde{\nu}$ = 3306 (w), 2953 (w), 1728 (s), 1692 (s), 1652 (s), 1524 (s), 1454 (m), 1368 (m), 1282 (s), 1230 (s), 1207 (s), 1132 (m), 1038 (s), 1001 (m), 910 (w), 747 (s), 696 (vs), 656 (s) cm^{-1}; CHN elemental analysis calcd. for $C_{25}H_{32}N_2O_5$: N, 6.36; C, 68.16; H, 7.32; found N, 6.32; C, 67.71; H, 6.97; HRMS-EI (TOF) calcd. for $C_{25}H_{32}N_2O_5$: 440.2311; found: 440.2322 [M^+]; melting point: 111.0 °C. The analytical data matched those reported in the literature for *N*-(*N*-carboxybenzoyl-L-phenylalanyl)-L-leucine ethyl ester[39].

Synthesis of *N*-carboxybenzoyl-L-prolyl-L-phenylalanine ethyl ester (5adl) [CAS: 18532-06-4]. Compound **5adl** was prepared following the general procedure D, starting from *N*-carboxybenzoyl-L-proline (**1ad**) (252 mg, 1.0 mmol) and L-phenylalanine ethyl ester hydrochloride (**4l**) (347 mg, 1.5 mmol). After purification **5adl** was isolated as a colorless oil (345 mg, 81 %). ^{1}H-NMR (600 MHz, DMSO-d_6) δ = 8.28-8.42 (m, 1 H), 7.10-7.41 (m, 10 H), 5.06 (dd, *J* = 31.7, 12.6 Hz, 1 H), 4.94 (dd, *J* = 70.1, 13.5 Hz, 2 H), 4.38-4.54 (m, 1 H), 4.19-4.27 (m, 1 H), 3.94-4.05 (m, 2 H), 3.36-3.49 (m, 2 H), 3.26-3.32 (m, 32 H), 2.84-3.07 (m, 2 H), 1.98-2.14 (m, 1 H), 1.65-1.79 (m, 2 H), 1.07-1.07 (m, 1 H), 1.05-1.12 (m, 3 H) ppm; ^{13}C-NMR (151 MHz, DMSO-d_6) δ = 172.2, 171.4, 154.0, 137.2, 137.0, 129.2, 129.0, 128.4, 128.3, 128.2, 128.1, 127.8, 127.5, 127.5, 127.0, 127.0, 126.8, 126.5, 126.4, 65.9, 65.7, 60.5, 60.4, 59.6, 59.2, 53.6, 53.3, 47.0, 46.4, 36.5, 31.0, 29.8, 26.3, 23.6, 22.8, 13.9 ppm; IR (ATR): $\tilde{\nu}$ = 2978 (w), 1738 (m), 1675 (s), 1526 (w), 1498 (w), 1412 (s), 1353 (s), 1182 (s), 1115 (s), 1028 (m), 984 (w), 918 (w), 861 (w), 741 (m), 697 cm^{-1}; CHN elemental analysis calcd. for $C_{24}H_{28}N_2O_5$: N, 6.60; C, 67.91; H, 6.65; found N, 6.50; C, 67.56; H, 6.84; HRMS-EI (TOF) calcd. for $C_{24}H_{28}N_2O_5$: 424.1998; found: 424.1991 [M^+].

Synthesis of *N*-(*N*-carboxybenzoyl-L-methionyl)-L-phenylalanine ethyl ester (5ael). Compound **5ael** was prepared following the general procedure D, starting from *N*-carboxybenzoyl-L-methionine (**1ae**) (289 mg, 1.0 mmol) and L-phenylalanine ethyl ester hydrochloride (**4l**) (347 mg, 1.5 mmol). After purification **5ael** was isolated as a white solid (453 mg, 99 %). ^{1}H-NMR (600 MHz, DMSO-d_6) δ = 8.34 (d, *J* = 7.3 Hz, 1 H), 7.45 (d, *J* = 8.2 Hz, 1 H), 7.22-7.23 (m, 1 H), 7.14-7.39 (m, 11 H), 4.97-5.05 (m, 2 H), 4.39-4.50 (m, 1 H), 4.05-4.15 (m, 1 H), 4.02 (q, *J* = 7.0 Hz, 2 H), 2.84-3.09

(m, 2 H), 2.36-2.47 (m, 2 H), 2.01 (s, 3 H), 1.70-1.88 (m, 2 H), 1.40 (s, 1 H), 1.09 (t, *J* = 7.3 Hz, 3 H) ppm; ^{13}C-NMR (151 MHz, DMSO-*d*$_6$) δ = 171.6, 171.3, 155.8, 137.0, 137.0, 129.2, 129.1, 128.3, 128.2, 128.2, 127.8, 127.7, 127.0, 126.5, 65.4, 60.6, 60.5, 53.6, 36.5, 31.8, 29.5, 26.4, 14.6, 13.9, ppm; IR (ATR): $\tilde{\nu}$ = 3313 (w), 3033 (w), 2971 (w), 2917 (w), 1736 (s), 1688 (m), 1656 (s), 1528 (s), 1455 (w), 1387 (w), 1365 (w), 1283 (m), 1226 (s), 1183 (m), 1019 (m), 906 (w), 869 (w), 843 (w), 753 (s), 695 (vs) cm^{-1}; CHN elemental analysis calcd. for $C_{24}H_{30}N_2O_5S$: N, 6.11; C, 62.86; H, 6.59; found N, 6.05; C, 62.92, H, 6.52; HRMS-EI (TOF) calcd. for $C_{24}H_{30}N_2O_5S$: 458.1875; found: 458.1879 [M$^+$]; melting point: 81.3 °C.

Synthesis of *N*-(*N*-carboxybenzoyl-L-glycinyl)-L-phenylalanine ethyl ester (5afl) [CAS: 3956-78-3]**.** Compound **5afl** was prepared following the general procedure D, starting from *N*-carboxybenzoyl-L-glycin (**1af**) (211 mg, 1.0 mmol) and L-phenylalanine ethyl ester hydrochloride (**4l**) (347 mg, 1.5 mmol). After purification **5afl** was isolated as a brown slurry (301 mg, 82 %). ^{1}H-NMR (600 MHz, DMSO-*d*$_6$) δ = 8.30 (d, *J* = 7.6 Hz, 1 H), 7.42 (t, *J* = 6.2 Hz, 1 H), 7.16-7.39 (m, 10 H), 5.02 (s, 2 H), 4.41-4.51 (m, 1 H), 4.03 (q, *J* = 7.0 Hz, 9 H), 3.52-3.69 (m, 2 H), 2.84-3.03 (m, 2 H), 1.10 (t, *J* = 7.0 Hz, 3 H) ppm; ^{13}C-NMR (151 MHz, DMSO-*d*$_6$) δ = 171.4, 169.2, 156.4, 137.0, 137.0, 129.1, 128.3, 128.3, 127.8, 127.7, 126.6, 65.4, 60.5, 53.6, 43.1, 36.9, 13.9 ppm; IR (ATR): $\tilde{\nu}$ = 3291 (w), 3063 (w), 1734 (m), 1693 (m), 1662 (s), 1532 (m), 1455 (m), 1375 (w), 1212 (s), 1157 (m), 1042 (m), 909 (w), 740 (s), 697 (vs), 650(m) cm^{-1}; HRMS-EI (TOF) calcd. for $C_{21}H_{24}N_2O_5$: 384.1685; found: 384.1714 [M$^+$]; melting point: 87.0 °C. The analytical data matched those reported in the literature for *N*-(*N*-carboxybenzoyl-L-glycinyl)-L-phenylalanine ethyl ester[40].

Supplementary References

1. Kita, Y., Maeda, H., Omori, K., Okuno, T. & Tamura, Y. A Novel Efficient Synthesis of 1-Ethoxyvinyl Esters and Their Use in Acylation of Amines and Alcohols: Synthesis of Water-Soluble Oxaunomycin Derivatives. *Synlett* **1993**, 273–274 (1993).
2. 1-Ethoxyvinyl benzoate was isolated according to literature procedure, but purificated with column chromatography (SiOH; pentane:diethylether:NEt_3 90:5:5). Yin, J., Bai, Y., Mao, M. & Zhu, G. Silver-Catalyzed Regio- and Stereoselective Addition of Carboxylic Acids to Ynol Ethers. *J. Org. Chem*. **79,** 9179–9185 (2014).
3. 1-Hexen-2-ol benzoate was isolated according to literature procedure. Chary, B. C. & Kim, S. Gold(I)-Catalyzed Addition of Carboxylic Acids to Alkynes. *J. Org. Chem.* **75,** 7928–7931 (2010).

4. Yin, J., Bai, Y., Mao, M. & Zhu, G. Silver-Catalyzed Regio- and Stereoselective Addition of Carboxylic Acids to Ynol Ethers. *J. Org. Chem.* **79,** 9179–9185 (2014).
5. Jones, R. C. F. & Nichols, J. R. Coenzyme-inspired chemistry 2: 4,5-dihydroimidazolium ylides (NHCs) and the reactions of 2-(1-hydroxyalkyl)-4,5-dihydroimidazoles. *Org. Biomol. Chem.* **11,** 5926 (2013).
6. Ueda, T., Konishi, H. & Manabe, K. Palladium-Catalyzed Fluorocarbonylation Using *N*-Formylsaccharin as CO Source: General Access to Carboxylic Acid Derivatives. *Org. Lett.* **15,** 5370–5373 (2013).
7. Morimoto, H., Fujiwara, R., Shimizu, Y., Morisaki, K. & Ohshima, T. Lanthanum(III) Triflate Catalyzed Direct Amidation of Esters. *Org. Lett.* **16,** 2018–2021 (2014).
8. Starkov, P. & Sheppard, T. D. Borate esters as convenient reagents for direct amidation of carboxylic acids and transamidation of primary amides. *Org. Biomol. Chem.* **9,** 1320 (2011).
9. Zhou, L. & Lu, W. Palladium-Catalyzed β-Acyloxylation of Simple Amide *via* sp^3 C–H Activation. *Org. Lett.* **16,** 508–511 (2014).
10. Kawagoe, Y., Moriyama, K. & Togo, H. Facile preparation of amides from carboxylic acids and amines with ion-supported Ph3P. *Tetrahedron* **69,** 3971–3977 (2013).
11. Bian, Y.-J., Chen, C.-Y. & Huang, Z.-Z. Synthesis of Imides by Palladium-Catalyzed C□H Functionalization of Aldehydes with Secondary Amides. *Chem. Eur. J.* **19,** 1129–1133 (2013).
12. Roberts, B., Liptrot, D., Alcaraz, L., Luker, T. & Stocks, M. J. Molybdenum-Mediated Carbonylation of Aryl Halides with Nucleophiles Using Microwave Irradiation. *Org. Lett.* **12,** 4280–4283 (2010).
13. Rombouts, F. *et al.* Microwave-assisted *N*-debenzylation of amides with triflic acid. *Tetrahedron Lett.* **51,** 4815–4818 (2010).
14. Oda, Y., Sato, T. & Chida, N. Direct Chemoselective Allylation of Inert Amide Carbonyls. *Org. Lett.* **14,** 950–953 (2012).
15. Molander, G. A. & Hiebel, M.-A. Synthesis of Amidomethyltrifluoroborates and Their Use in Cross-Coupling Reactions. *Org. Lett.* **12,** 4876–4879 (2010).
16. Lanigan, R. M., Starkov, P. & Sheppard, T. D. Direct Synthesis of Amides from Carboxylic Acids and Amines Using $B(OCH_2CF_3)_3$. *J. Org. Chem.* **78,** 4512–4523 (2013).
17. Saito, Y., Ouchi, H. & Takahata, H. Carboxamidation of carboxylic acids with 1-tert-butoxy-2-tert-butoxycarbonyl-1,2-dihydroisoquinoline (BBDI) without bases. *Tetrahedron* **64,** 11129–11135 (2008).
18. Moyer, M. P., Feldman, P. L. & Rapoport, H. Intramolecular nitrogen-hydrogen, oxygen-hydrogen and sulfur-hydrogen insertion reactions. Synthesis of heterocycles from .alpha.-diazo .beta.-keto esters. *J. Org. Chem.* **50,** 5223–5230 (1985).
19. Katritzky, A. R. & Monro, A. M. 31. *N*-oxides and related compounds. Part VII. Per-acid oxidation of some conjugated pyridines. *J. Chem.Soc. (Resumed)* 150, (1958).
20. Chang, Y. H. *et al.* Study of AMP-Activated Protein Kinase Agonists by Structure-Based Drug Designing. *Adv. Mat. Res.* **79,** 2187–2190 (2009).
21. Štefane, B. & Polanc, S. Hydrogenation of BF2 complexes with 1,3-dicarbonyl ligands. *Tetrahedron* **65,** 2339–2343 (2009).
22. Gernigon, N., Al-Zoubi, R. M. & Hall, D. G. Direct Amidation of Carboxylic Acids Catalyzed by ortho -Iodo Arylboronic Acids: Catalyst Optimization, Scope, and Preliminary Mechanistic Study Supporting a Peculiar Halogen Acceleration Effect. *J. Org. Chem.* **77,** 8386–8400 (2012).
23. Wu, H., Kelley, C. J., Pino-Figueroa, A., Vu, H. D. & Maher, T. J. Macamides and their synthetic analogs: Evaluation of in vitro FAAH inhibition. *Bioorg. Med. Chem.* **21,** 5188–5197 (2013).
24. Chen, W., Liu, Y. & Chen, Z. A Highly Efficient and Practical New Allylboronate Tartramide for the Asymmetric Allylboration of Achiral Aldehydes. *Eur. J. Org. Chem.* **2005,** 1665–1668 (2005).

25. Nadimpally, K. C., Thalluri, K., Palakurthy, N. B., Saha, A. & Mandal, B. Catalyst and solvent-free amidation of inactive esters of N-protected amino acids. *Tetrahedron Lett.* **52,** 2579–2582 (2011).
26. Blay, G., Fernández, I., Marco-Aleixandre, A. & Pedro, J. Enantioselective Addition of Dimethylzinc to α-Keto Esters. *Synthesis* **2007,** 3754–3757 (2007).
27. Cousins, R. P. C., Curtis, A. D. M., Ding, W. C. & Stoodley, R. J. 1,5-asymmetric inductions in the reactions of 2-(2′,3′,4′,6′-tetra-O-acetyl-β-d-glucopyranosyloxy)benzaldehyde with Danishefsky's diene. *Tetrahedron Lett.* **36,** 8689–8692 (1995).
28. Correa, A. & Martin, R. Ni-Catalyzed Direct Reductive Amidation via C–O Bond Cleavage. *J. Am. Chem. Soc.* **136,** 7253–7256 (2014).
29. Harvill, E. K., Herbst, R. M., Schreiner, E. C. & Roberts, C. W. The Synthesis of 1,5-Disubstituted Tetrazoles. *J. Org. Chem.* **15,** 662–670 (1950).
30. K. Cermak, J., Kolar, K. & Cirkva, V. Rapid and Efficient Synthesis of N-alkylbenzamides Under Microwave Irradiation. *Lett. Org. Chem.* **10,** 126–130 (2013).
31. de Silva, S. O. *et al.* Directed ortho metalation of n,n-diethyl benzamides. Methodology and regiospecific synthesis of useful contiguously tri- and tetra-substituted oxygenated aromatics, phthalides and phthalic anhydrides. *Tetrahedron* **48,** 4863–4878 (1992).
32. Iranpoor, N., Firouzabadi, H., Nowrouzi, N. & Khalili, D. Selective mono- and di-N-alkylation of aromatic amines with alcohols and acylation of aromatic amines using Ph_3P/DDQ. *Tetrahedron* **65,** 3893–3899 (2009).
33. Song, Q., Feng, Q. & Yang, K. Synthesis of Primary Amides via Copper-Catalyzed Aerobic Decarboxylative Ammoxidation of Phenylacetic Acids and α-Hydroxyphenylacetic Acids with Ammonia in Water. *Org. Lett.* **16,** 624–627 (2014).
34. Kokare, N., Nagawade, R., Rane, V. & Shinde, D. Organophosphorus Esters of 1-Hydroxy-2-phenylbenzimidazole: Synthesis and Utilization as Novel Peptide Coupling Reagents. *Synthesis* **2007,** 766–772 (2007).
35. Tamaru, Y., Yamada, Y. & Yoshida, Z. Direct Oxidative Transformation of Aldehydes to Amides by Palladium Catalysis. *Synthesis* **1983,** 474–476 (1983).
36. Huang, W., Wang, M. & Yue, H. Conversion of *N* -Acyl Amino Acids into Imides via Oxidative Decarboxylation Induced by $Ag^+/Cu^{2+}/S_2O_8^{2-}$ in Water. *Synthesis* **2008,** 1342–1344 (2008).
37. Losse, G. & Demuth, E. Diphenylketen als Reagens zur Knüpfung von Peptidbindungen. *Chem. Ber.* **94,** 1762–1766 (1961).
38. Scott-Burden, T. & Hawtrey, A. O. Use of 2, 4, 6-tribromophenyl, aminoacid esters in peptide synthesis. *Tetrahedron. Lett.*, ***8*****,** 4831-4832. **(**1967).
39. Nomura, R., Yamada, Y. & Matsuda, H. Triphenylstibine oxide-phosphorus(V) sulfide as a novel condensation catalyst system: application to the synthesis of dipeptides. *App. Organomet.Chem.* **3,** 355–357 (1989).
40. Schüssler, H. & Zahn, H. Beitrag zur Reaktionsweise von Carbobenzoxy-aminosäuren mit Dicyclohexylcarbodiimid. *Chem. Ber.* **95,** 1076–1080 (1962).
